# Cross‐cohort NULISAseq identifies tau‐anchored CSF and plasma‐specific signatures across the amyloid‐positive Alzheimer's disease

**DOI:** 10.1002/alz.71915

**Published:** 2026-10-12

**Authors:** Lan Ding, Menghan Zheng, Simiao Wang, Wenying Liu, Yumei Liang, Siqi Xie, Ting Yang, Dandan Sheng, Shuheng Wang, Jianpeng Sheng, Jianping Jia, the Alzheimer's Disease Neuroimaging Initiative

**Affiliations:** ^1^ Chinese Institutes for Medical Research, Beijing (CIMR) Capital Medical University Beijing P. R. China; ^2^ Innovation Center for Neurological Disorders and Department of Neurology, Xuanwu Hospital Capital Medical University, National Clinical Research Center for Geriatric Diseases Beijing P. R. China; ^3^ Beijing Key Laboratory of Geriatric Cognitive Disorders Beijing P. R. China; ^4^ Clinical Center for Neurodegenerative Disease and Memory Impairment Capital Medical University Beijing P. R. China; ^5^ Center of Alzheimer's Disease Beijing Institute of Brain Disorders Collaborative Innovation Center for Brain Disorders Capital Medical University Beijing P. R. China; ^6^ Key Laboratory of Neurodegenerative Diseases Ministry of Education Beijing P. R. China

**Keywords:** Alzheimer's disease, ATN framework, cerebrospinal fluid proteomics, NULISAseq, plasma biomarkers, tau

## Abstract

**INTRODUCTION:**

ATN‐stratified cerebrospinal fluid (CSF) proteomic signals may partly reflect classifier tau immunoassays; reproducible non‐tau signals and CSF–plasma differences remain unclear.

**METHODS:**

We profiled CSF from 60 A^+^ Beijing patients with Alzheimer's disease (AD) and 875 A^+^ Alzheimer's Disease Neuroimaging Initiative (ADNI) participants using the NULISAseq CNS Disease Panel. Differential expression was assessed using a false discovery rate (FDR) < 0.05 and |log_2_FC|≥0.5 across three ATN contrasts. We also analyzed ADNI plasma, protein–cognition correlations, tau‐adjusted associations, and nested models comparing non‐tau proteins with tau biomarkers.

**RESULTS:**

Eight CSF proteins met the cross‐cohort criterion within at least one matched contrast: pTau‐217, pTau‐181, pTau‐231, MAPT, SMOC1, MDH1, VSNL1, and FABP3. NULISA tau correlated with INNOTEST (ρ = 0.75–0.85) and Elecsys (ρ = 0.65–0.68). T and N classifications agreed for 94.6% of ADNI participants (κ = 0.880); after adjustment for CSF pTau‐217, all 12 associations involving the four non‐tau proteins retained their direction and FDR support. In CSF, Elecsys p‐tau181 and NULISA tau‐family analytes had the strongest cognitive correlations (mean |ρ| = 0.35 and 0.29–0.34, respectively), followed by FABP3 (0.24). Adding the four‐protein block after tau increased cross‐validated *R* ^2^ across all three cross‐sectional outcomes in models without diagnosis (+0.0101 to +0.0265), most clearly for the Alzheimer's Disease Assessment Scale–Cognitive Subscale, 13‐item version (ADAS‐Cog13); diagnosis‐conditioned models showed no stable increment beyond tau and clinical characteristics. In plasma, pTau‐217 was the only analyte that met both thresholds in all three contrasts.

**DISCUSSION:**

CSF NULISAseq identified a reproducible tau‐associated signature across A^+^ cohorts that extended beyond the tau family. The four non‐tau proteins extend the molecular profile associated with tau‐defined disease state and merit further evaluation for biological stratification in independent cohorts.

## Highlights

- Eight cerebrospinal fluid (CSF) proteins replicated across cohorts in matched ATN contrasts.
- The non‐tau CSF proteins were SMOC1, MDH1, FABP3, and VSNL1.
- Non‐tau signals retained direction and false discovery rate (FDR) support after adjusting for CSF pTau‐217.
- Four‐protein Δ*R* ^2^ was positive before diagnosis adjustment but smaller afterward.
- pTau‐217 was the only plasma analyte to meet both thresholds in all three contrasts.

## 1 BACKGROUND

Alzheimer's disease (AD), the leading cause of dementia worldwide, is now characterized as a biologically defined disorder. The National Institute on Aging–Alzheimer's Association (NIA‐AA) research framework organizes AD biomarkers along three axes: amyloid (A), tau pathology (T), and neurodegeneration (N), providing a system for biological classification and staging.^1^, ^2^ Within this framework, individuals who share amyloid positivity may nonetheless differ in tau burden and neurodegeneration severity, and understanding the molecular heterogeneity across ATN‐defined states remains an active area of investigation.^3^, ^4^

A key methodological challenge in ATN‐stratified cerebrospinal fluid (CSF) proteomic studies is the close biochemical relationship between the analytes used for biomarker classification and those measured by multiplex proteomic platforms. In CSF‐based studies, T and N status are typically operationalized using phosphorylated tau (p‐tau) and total tau (t‐tau), respectively—both members of the tau protein family.^5^, ^6^ This shared biochemical origin raises the possibility that proteomic associations attributed to either axis may partly reflect cross‐platform concordance between the classifying immunoassay and the multiplex proteomic readout, rather than broader non‐tau molecular variation. A second challenge is generalizability: many published CSF proteomic studies of ATN‐stratified AD are based on single cohorts, and findings from small discovery cohorts do not always reproduce at the same effect‐size threshold in larger independent cohorts using different ATN‐cutoff systems and clinical compositions.^7^, ^8^

NULISAseq is a nucleic acid–linked immuno‐sandwich proteomic platform with attomolar sensitivity and high multiplexing capacity.^9^ Its CNS Disease panel covers tau‐family, amyloid‐related, and broader central nervous system (CNS) ‐related analytes, making it well suited to study ATN‐stratified CSF proteomic variation. Importantly, by measuring tau‐family analytes and broader CNS proteins within the same multiplex CSF assay, NULISAseq enables direct comparison of classifier‐proximal tau signals with residual non‐tau proteomic signals in the same biological samples. Alzheimer's Disease Neuroimaging Initiative (ADNI) has released NULISAseq CNS panel data for both CSF and plasma, together with assay documentation,^10^ allowing cross‐cohort evaluation using a common proteomic panel and participant‐matched measurements in the two fluids.

In the present study, we used NULISAseq to profile CSF from a Beijing discovery cohort of 60 biologically defined amyloid‐positive AD patients recruited at Xuanwu Hospital, Capital Medical University, and integrated ADNI NULISAseq CNS panel data from 875 amyloid‐positive participants as an independent replication cohort. Because ATN status is biologically rather than clinically defined, the ADNI replication analysis tested whether signals discovered in clinically diagnosed A^+^ AD generalize across the broader A^+^ biological continuum. Beijing candidates meeting false discovery rate (FDR) < 0.05 and |log_2_FC| ≥ 0.5 were assessed for cross‐cohort consistency in ADNI under the same threshold and direction requirement. We also examined cross‐platform tau concordance, attenuation after adjustment for tau measures, diagnostic composition, CSF–plasma differences, and protein–cognition correlations. Together, this discovery–replication design was intended to identify reproducible CSF biomarkers associated with ATN‐defined disease severity in amyloid‐positive AD.

## 2 METHODS

### 2.1 Cohorts and study design

Data used in the preparation of this article were obtained from the Alzheimer's Disease Neuroimaging Initiative (ADNI) database (adni.loni.usc.edu). The ADNI was launched in 2003 as a public‐private partnership, led by Principal Investigator Michael W. Weiner, MD. The primary goal of ADNI has been to test whether serial magnetic resonance imaging (MRI), positron emission tomography (PET), other biological markers, and clinical and neuropsychological assessment can be combined to measure the progression of mild cognitive impairment (MCI) and early Alzheimer's disease (AD). For up‐to‐date information, see www.adni‐info.org.

The discovery cohort included 60 amyloid‐positive (A^+^) patients with clinically diagnosed AD recruited from Xuanwu Hospital, Capital Medical University, Beijing, China. This cohort is referred to as the Beijing cohort throughout the manuscript. AD diagnosis followed the NIA‐AA 2011 criteria for probable AD dementia,^11^ and amyloid positivity was confirmed by ^1^ ^8^F‐florbetaben amyloid positron emission tomography (PET) with visual interpretation by qualified nuclear medicine physicians.^12^ Demographic and clinical variables collected at baseline included age, sex, education, Mini‐Mental State Examination (MMSE), Montreal Cognitive Assessment (MoCA), comorbidities, and lifestyle factors. Apolipoprotein E (APOE) genotype was determined by standard polymerase chain reaction (PCR) ‐based methods and converted to a binary ε4‐carrier variable. The study was approved by the Ethics Committee of Xuanwu Hospital, Capital Medical University (Approval No. 2019‐110); written informed consent was obtained from all participants or their legally authorized representatives, in accordance with the Declaration of Helsinki.

**RESEARCH IN CONTEXT**

1. **Systematic review**: The authors reviewed PubMed and Web of Science through April 2026. Multiplex cerebrospinal fluid (CSF) and plasma proteomic studies have reported tau‐family and non‐tau signals across ATN‐defined Alzheimer's disease, but cross‐cohort replication under a common threshold using different classifier assays remains limited.
2. **Interpretation**: Eight CSF proteins met the cross‐cohort criterion in Beijing and Alzheimer's Disease Neuroimaging Initiative (ADNI), including four non‐tau proteins. All 12 associations involving the non‐tau proteins retained direction and false discovery rate (FDR) support after CSF pTau‐217 adjustment, although every individual 95% CI upper limit fell below 0.5. Highly concordant T and N classifications indicated that the matched contrasts captured overlapping tau‐associated burden. Stable incremental value beyond Elecsys tau and clinical characteristics was not demonstrated. Plasma findings were more focused and led by pTau‐217.
3. **Future directions**: Independent prospective cohorts using tau‐positron emission tomography (PET) or staging independent of tau immunoassays should test whether these proteins improve biological stratification or prediction beyond established tau and clinical measures.

ADNI contributed 875 amyloid‐positive participants with a NULISA CSF index record. Diagnosis was matched to this record by exact VISCODE2 match when available and otherwise by the nearest dated assessment; the resulting groups were cognitively normal (CN) (*n* = 212), mild cognitive impairment (MCI) (*n* = 438), and AD (*n* = 225). The primary replication analysis included all participants and did not use diagnosis as a grouping variable. After excluding 10 CSF samples with a SampleQC status of “warning,” 865 participants remained for differential‐abundance and cross‐platform analyses. QC‐passed plasma NULISA data were available for 793 participants, and 785 had QC‐passed measurements in both fluids. Diagnosis‐based sensitivity analyses used the CSF‐index diagnosis; the AD‐only sensitivity sample comprised 221 QC‐passed participants. Cohort characteristics and ATN classification are summarized in Table 1.

**TABLE 1:** Cohort characteristics and ATN classification.

Characteristic	Beijing discovery cohort (*n* = 60)	ADNI A^+^ replication cohort (*n* = 875)
Age, years (mean ± SD)	65.6 ± 8.5	73.4 ± 7.4
Age range, years	53–83	55–91
Female, *n* (%)	45 (75.0%)	385 (44.0%)
Education, years (mean ± SD)	10.3 ± 4.2	16.1 ± 2.8
MMSE, mean ± SD	14.8 ± 6.2	26.7 ± 2.9
ADAS‐Cog13, mean ± SD	—	19.0 ± 10.2
CDR‐SB, mean ± SD	—	2.0 ± 2.0
APOE ε4 carrier, *n* (%)	31 (51.7%)	560 (64.0%)
Clinical diagnosis	All AD (*n* = 60)	CN 212 / MCI 438 / AD 225
T^+^, *n* (%)	44 (73.3%)	589 (67.3%)
N^+^, *n* (%)	40 (66.7%)	552 (63.1%)
A^+^T^+^N^+^, *n* (%)	36 (60.0%)	547 (62.5%)
A^+^T^−^N^−^, *n* (%)	12 (20.0%)	281 (32.1%)
Other ATN profiles, *n* (%)	12 (20.0%)	47 (5.4%)
ATN classification platform	Fujirebio INNOTEST ELISA	Roche Elecsys ECLIA

*Note*: Values are mean ± SD or *n* (%) as indicated. ADNI diagnosis was anchored to the NULISA CSF index record by exact VISCODE2 match when available and otherwise by nearest date. ADNI cognition was linked to that CSF index by exact VISCODE2 match when available and otherwise by the nearest dated assessment within ± 90 days; Table 1 sample sizes were MMSE *n* = 791, ADAS‐Cog13 *n* = 849, and CDR‐SB *n* = 788, and other ADNI denominators were *n* = 875. ATN classification in Beijing used Fujirebio INNOTEST ELISA with age‐stratified upper‐reference thresholds for T and N. ADNI used Roche Elecsys research cut points: Aβ42 < 976.6 pg/ml for A^+^ when PET visual read was unavailable, p‐tau181 > 21.8 pg/ml for T^+^, and total tau > 245 pg/ml for N^+^. ADAS‐Cog13 and CDR‐SB were not routinely administered in the Beijing cohort and are shown as “—”. Ten ADNI CSF samples had SampleQC = “warning” rather than “passed” and were excluded from the primary CSF differential‐abundance and cross‐platform analyses, yielding *n* = 865. The CSF‐index‐anchored AD‐only sensitivity subset included *n* = 221 among SampleQC‐passed participants for the T and N contrasts and *n* = 210 for the concordant‐extreme contrast.

Abbreviations: AD, Alzheimer's disease; ADAS‐Cog13, Alzheimer's Disease Assessment Scale–Cognitive Subscale (13‐item); ADNI, Alzheimer's Disease Neuroimaging Initiative; ATN, amyloid, tau pathology, and neurodegeneration; CDR‐SB, Clinical Dementia Rating ‐ Sum of Boxes; CN, cognitively normal; CSF, cerebrospinal fluid; MCI, mild cognitive impairment; MMSE, Mini‐Mental State Examination; PET, positron emission tomography.

### 2.2 CSF and plasma collection and core biomarker measurement

Beijing‐cohort CSF was collected via lumbar puncture into sterile polypropylene tubes, centrifuged at 1000 × *g* for 10 minutes at 4°C, aliquoted, and stored at −80°C; samples underwent a single freeze‐thaw cycle before proteomic profiling. Core CSF biomarkers—Aβ42, Aβ40, p‐tau181, and total tau—were measured using the Fujirebio INNOTEST ELISA platform.^13^ ADNI CSF and plasma collection followed the standard ADNI biospecimen protocol. ADNI core CSF biomarkers (Aβ42, p‐tau181, and total tau) were measured using the Roche Elecsys electrochemiluminescence immunoassay (Roche Diagnostics, Penzberg, Germany).

### 2.3 ATN classification

In Beijing, A^+^ status was confirmed by ^1^ ^8^F‐florbetaben amyloid‐PET visual positivity.^12^ In ADNI, A^+^ status was assigned by amyloid‐PET visual read when available and otherwise by Roche Elecsys CSF Aβ42 < 976.6 pg/ml.^14^ T and N status in Beijing were assigned using age‐stratified Fujirebio INNOTEST upper reference limits (T^+^ if p‐tau181 > 45.67 pg/ml [age ≤44] or > 66.26 pg/ml [age > 44]; N^+^ if total tau > 225 pg/ml [age ≤50], > 370 pg/ml [age 51–70], or > 512 pg/ml [age > 70]). In ADNI, previously applied ADNI Elecsys research cut points were used for T and N (T^+^ if p‐tau181 > 21.8 pg/ml; N^+^ if total tau > 245 pg/ml).^6^ These cohort‐established classifier assays were independent of the NULISA panel. NULISA pTau‐217 was evaluated as an analyte and continuous tau‐adjustment measure rather than used to define the comparison groups.

### 2.4 NULISAseq proteomic profiling and quality control

Beijing and ADNI CSF samples and ADNI plasma samples were profiled using the NULISAseq targeted proximity extension assay (Alamar Biosciences) on the CNS Disease Panel (127 protein targets). Protein expression was reported as Normalized Protein Quantification (NPQ), defined as log_2_‐transformed normalized counts after internal‐control and inter‐plate‐control normalization with a 10^4^ rescaling factor. Per‐target detectability was the proportion of samples with NPQ above the assay‐level limit of detection (LoD), and intra‐plate coefficients of variation were calculated from AQSC replicates after conversion to the linear 2^NPQ scale.

### 2.5 Differential expression analysis

Differential protein expression was assessed using limma with empirical Bayes moderation in Beijing CSF, ADNI CSF, and ADNI plasma.^15^ Models adjusted for age, sex, APOE ε4 carrier status, and technical batch, defined as the sample‐information group in the single‐plate Beijing data and as NULISA plate in ADNI. The same three prespecified ATN contrasts were tested in each cohort: T^+^ versus T^−^, N^+^ versus N^−^, and T^+^N^+^ versus T^−^N^−^ (Beijing: *n* = 44 vs. 16, 40 vs. 20, and 36 vs. 12; ADNI: *n* = 585 vs. 280, 548 vs. 317, and 543 vs. 275). *P*‐values were corrected using the Benjamini–Hochberg procedure within each contrast and panel. Proteins meeting both FDR < 0.05 and |log_2_FC| ≥ 0.5 were considered threshold‐passing proteins for the primary analyses. The harmonized CSF panel for testing comprised 124 analytes; APOE and C‐reactive protein (CRP) were excluded because quantitative values were unavailable from the ADNI CSF export. The plasma panel comprised 126 quantitative analytes. The APOE4 panel readout was excluded from every testing family because it is a binary genotype proxy rather than a quantitative analyte, and because genotype‐derived APOE ε4 carrier status was included as a model covariate.

The cross‐cohort consistent set comprised proteins meeting both thresholds with concordant log_2_FC direction in the same contrast in Beijing and ADNI; a protein was included in unique‐protein summaries if this occurred in at least one contrast. To match the Beijing clinical phenotype, the models were repeated in a CSF‐index‐anchored ADNI A^+^ AD subset (*n* = 221 for T and N; *n* = 210 for the concordant‐extreme contrast). The AD‐only analysis reported two‐sided moderated‐t 95% CIs, with the prespecified |log_2_FC| ≥ 0.5 threshold applied to point estimates.

### 2.6 Cross‐platform concordance and plasma analyses

Cross‐platform concordance between NULISAseq NPQ and matched core CSF immunoassay measurements was computed in both cohorts. In Beijing (*n* = 60 A^+^ AD), Spearman correlations were computed between NULISAseq pTau‐181, pTau‐217, pTau‐231, and MAPT and the corresponding INNOTEST p‐tau181 or total tau measurements. In ADNI (*n* = 865 A^+^ baseline subjects with paired NULISA CSF and Roche Elecsys CSF measurements), Pearson and Spearman correlations were computed between NULISA pTau‐181 and Elecsys p‐tau181, NULISA MAPT and Elecsys total tau, and NULISA pTau‐217 / pTau‐231 and Elecsys p‐tau181. Among these participant‐matched assay pairs, 854 had the same recorded collection date.

For plasma ATN‐stratified differential expression, limma models with the same covariate adjustment as CSF were applied to the ADNI A^+^ plasma subset: *n* = 793 for T^+^ versus T^−^ and N^+^ versus N^−^ contrasts, and *n* = 749 for the T^+^N^+^ versus T^−^N^−^ contrast after excluding 44 discordant T/N participants. Plasma volcano thresholds were |log_2_FC| ≥ 0.5 and FDR < 0.05, identical to CSF, and consistent with recent NULISA AD plasma studies.^16^, ^17^

### 2.7 Cross‐sectional cognitive correlations

Cognitive assessments were linked to the CSF draw by exact VISCODE2 match when available; otherwise, the nearest valid assessment within ± 90 days was selected. Cross‐sectional associations between CSF biomarkers and cognition were assessed using Spearman correlations in ADNI A^+^ participants who passed CSF NULISA QC. Outcome‐specific sample sizes were 781 for MMSE, 839 for the Alzheimer's Disease Assessment Scale–Cognitive Subscale, 13‐item version (ADAS‐Cog13), and 778 for Clinical Dementia Rating – Sum of Boxes (CDR‐SB). The analyte set comprised the eight strict cross‐cohort consistent proteins, Elecsys p‐tau181 as a reference biomarker, NRGN as a reference marker of synaptic injury, and eight contextual CSF analytes; NRGN was not part of the strict cross‐cohort consistent protein set. Plasma protein–cognition associations were assessed in the same way using baseline plasma NPQ for 16 prespecified analytes. To assess the contribution of diagnostic composition to the pooled correlations, nine focal analytes, comprising the eight strict cross‐cohort consistent proteins and NRGN, were examined within the CN, MCI, and AD groups. Diagnosis‐specific correlations were combined using Fisher z transformations with n−3 weights. Diagnosis‐adjusted correlations were also calculated after ranked protein and cognitive values had been residualized on diagnosis indicators. Finally, the total cross‐product of globally ranked protein and cognitive values was partitioned exactly into within‐ and between‐diagnosis components. The between/total fraction described the contribution of diagnosis‐group differences to the pooled rank covariance; it was not an R^2^ measure or a causal attributable fraction.

CSF and plasma pTau‐217 correlations with cognition were compared among participants with QC‐passed measurements in both fluids. Comparisons were conducted in all eligible participant‐matched pairs and repeated in pairs collected on the same day or within 30, 90, or 365 days. Because the CSF and plasma correlations shared participants and the same cognitive outcome, they were compared using Steiger's modification of the Dunn–Clark z test. The covariance term was evaluated at the mean of the two correlations, and the test was applied to Pearson correlations of midranks.^18^ Holm correction was applied jointly across the 15 comparisons defined by three cognitive outcomes and five sampling‐interval groups.

For exploratory longitudinal analyses, individual CDR‐SB and MMSE slopes were estimated by regressing each cognitive score on time in years relative to the CSF draw. Participants required at least three assessments from 0.25 years before the CSF draw onward, spanning at least one year (CDR‐SB *n* = 737; MMSE *n* = 724). Spearman correlations were then calculated between baseline NULISA NPQ and each cognitive slope for the eight strict cross‐cohort consistent proteins. Mean NPQ for these proteins was also calculated within each of the four A^+^ ATN strata.

### 2.8 Tau‐related sensitivity and incremental‐prediction analyses

Overlap between the operational T and N classifications was assessed by cross‐tabulation and Cohen's κ. Pearson correlation was calculated between the log‐transformed Elecsys p‐tau181 and total tau measurements used to define these classifications.

Tau‐adjustment sensitivity analyses refitted the original ATN contrast models separately with continuous same‐panel CSF NULISA pTau‐217 or separately measured plasma NULISA pTau‐217. Tau‐PET analyses used the UC Berkeley flortaucipir (FTP/AV‐1451) meta‐temporal composite standardized uptake value ratio (SUVR) from the scan nearest the CSF draw within ± 365 days. Tau‐PET was examined both as a sample‐derived lower‐versus‐upper‐tertile contrast (*n* = 128; tertile cut‐points, 1.1983 and 1.3517) and as a continuous covariate (*n* = 192; median absolute CSF‐to‐PET interval, 9 days).

Incremental prediction was evaluated for MMSE, ADAS‐Cog13, CDR‐SB, MMSE slope, and CDR‐SB slope. The clinical/technical model included age, sex, education, APOE ε4 carrier status, NULISA plate, and diagnosis; slope models also included the corresponding baseline cognitive score. The tau model added log‐transformed Elecsys p‐tau181 and total tau. The four‐protein model instead added SMOC1, MDH1, FABP3, and VSNL1, whereas the full model included both biomarker blocks.

The primary comparison assessed the change in out‐of‐fold *R* ^2^ after the four‐protein block was added to the tau model. For direct comparison of the two biomarker blocks, the tau and four‐protein models were also evaluated relative to the same clinical/technical model. Within each outcome, all models used the same complete‐case participants and identical folds. Participants required a recorded diagnosis and the corresponding cognitive assessment within ± 90 days of the CSF draw. Models were evaluated using five‐fold cross‐validation repeated 10 times.

Paired out‐of‐fold Δ*R* ^2^ was the primary performance measure. In‐sample ΔR^2^, partial F tests, and HC3 block‐Wald tests were secondary. Conditional 95% percentile intervals were obtained from 5000 participant resamples while the existing out‐of‐fold predictions were held fixed. Holm correction was applied across the five outcome‐specific block tests. A sensitivity analysis omitted diagnosis while retaining the same participants, folds, and interval procedure.

Analyses were performed in R 4.4.3 and 4.5.3. Within each ATN contrast, differential‐abundance *p*‐values were corrected using the Benjamini–Hochberg procedure as described in Section 2.5. A sensitivity analysis applied global Benjamini–Hochberg correction across all 372 ADNI CSF tests.

## 3 RESULTS

### 3.1 Cohort, ATN classification, and platform performance

We profiled CSF from 60 A^+^ Beijing participants with AD dementia and 875 A^+^ ADNI participants (Table 1). At the NULISA CSF index, ADNI diagnoses were CN (*n* = 212), MCI (*n* = 438), and AD (*n* = 225). ATN status was assigned using Fujirebio INNOTEST in Beijing and Roche Elecsys in ADNI, with amyloid‐PET visual reads prioritized for A status when available.

Among 127 NULISA targets, 92 (72.4%) were detected in at least 90% of Beijing samples; the median intra‐plate coefficient of variation for AQSC replicates was 5.50% on the linear 2^NPQ scale, and 59 of 60 samples passed all Alamar criteria (Figure S1). Excluding the single sample with a marginal internal‐control warning did not materially alter the differential‐abundance results.

### 3.2 Beijing ATN‐stratified differential expression

Using the threshold of FDR < 0.05 and |log_2_FC| ≥ 0.5, the Beijing cohort had five threshold‐passing proteins for T^+^ versus T^−^ (pTau‐181, pTau‐217, pTau‐231, MDH1, and SMOC1), four for N^+^ versus N^−^ (pTau‐181, pTau‐217, pTau‐231, and MAPT), and six for T^+^N^+^ versus T^−^N^−^ (pTau‐181, pTau‐217, pTau‐231, MAPT, FABP3, and VSNL1; Figure 1A–C). Across the three contrasts, these findings comprised four tau‐family analytes and four non‐tau proteins. Representative effects were pTau‐217 in the T contrast (log_2_FC = 1.09, FDR = 6.5×10^−^^4^), MAPT in the N contrast (0.63, 3.6×10^−^^3^), and FABP3 in the concordant‐extreme contrast (0.57, 2.9×10^−^^3^; Figure 1D–F). Cross‐platform tau correlations are shown in Figure S2.

**FIGURE 1:**
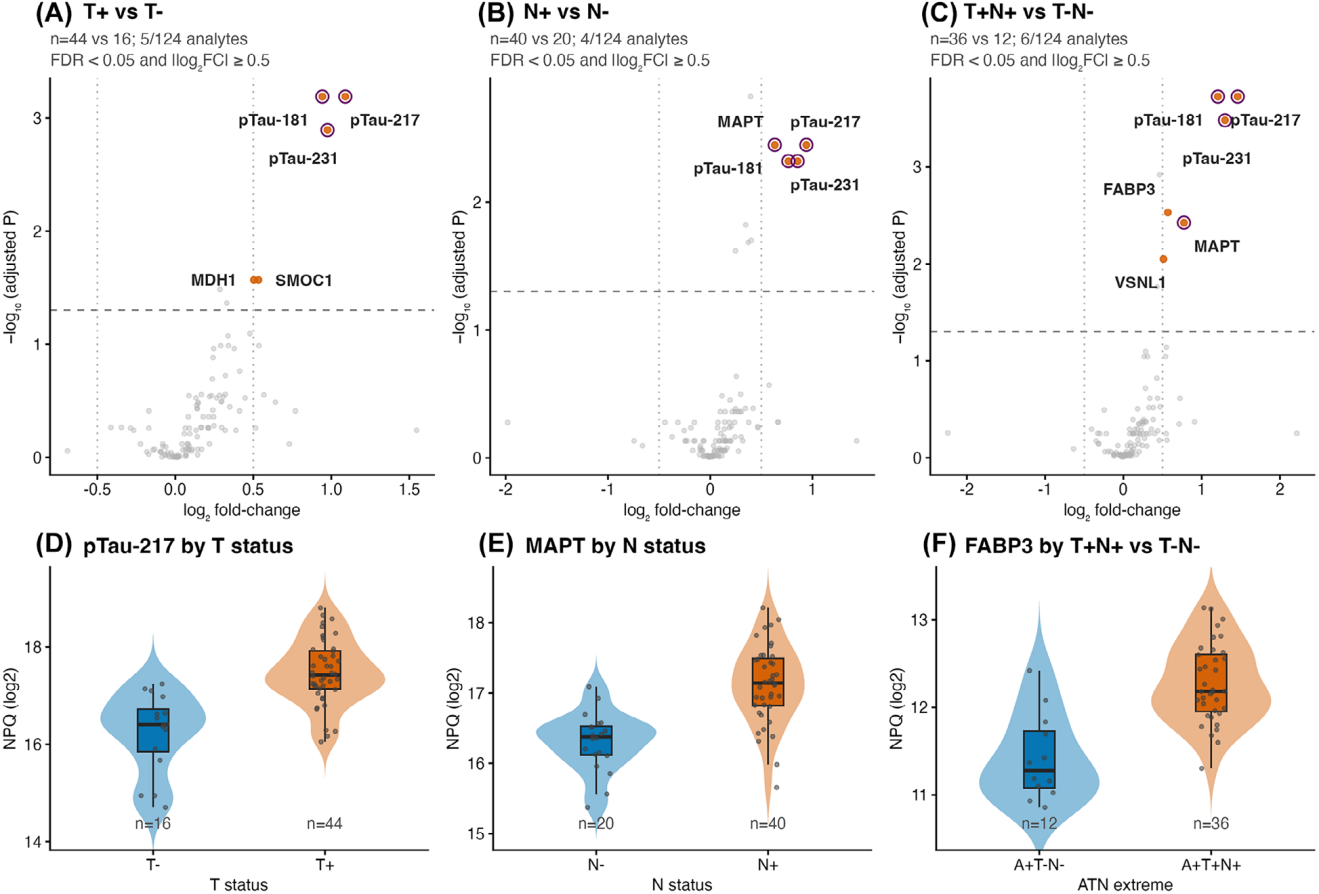
Beijing discovery cohort CSF ATN‐stratified differential abundance and representative between‐group distributions (*n* = 60 A^+^ AD). (A–C) Volcano plots for (A) T^+^ vs T^−^ (44 vs. 16), (B) N^+^ versus N^−^ (40 vs. 20), and (C) T^+^N^+^ versus T^−^N^−^ (concordant extremes; *n* = 48, 36 vs. 12). Limma models tested 124 CSF analytes (Methods Section 2.5). Orange points met both FDR < 0.05 and |log_2_FC| ≥ 0.5; gray points did not, and threshold‐passing tau‐family analytes are outlined in purple. Horizontal dashed and vertical dotted lines mark the two thresholds. (D–F) Distributions of one threshold‐passing analyte per contrast: pTau‐217 by T status, MAPT by N status, and FABP3 in the concordant‐extreme comparison. Violin and box plots show jittered observations and group sizes, medians, and IQRs. AD, Alzheimer's disease; ATN, amyloid, tau pathology, and neurodegeneration; CSF, cerebrospinal fluid; IQR, interquartile range.

### 3.3 Cross‐cohort consistency in ADNI

Applying the same framework to 865 ADNI A^+^ participants identified 15, 14, and 17 threshold‐passing analytes in the T, N, and concordant‐extreme contrasts, respectively (Figure 2A–C). All 15 Beijing protein–contrast findings met the cross‐cohort criterion in the corresponding ADNI contrast, comprising eight proteins: pTau‐181, pTau‐217, and pTau‐231 in all three contrasts; MAPT in the N and concordant‐extreme contrasts; MDH1 and SMOC1 in the T contrast; and FABP3 and VSNL1 in the concordant‐extreme contrast (Figure 3). SNCB, UCHL1, and PSEN1 were FDR‐supported in both cohorts but did not reach |log_2_FC| ≥ 0.5 in either, and NRGN met both thresholds in ADNI but not the Beijing effect‐size threshold (Figure S3).

**FIGURE 2:**
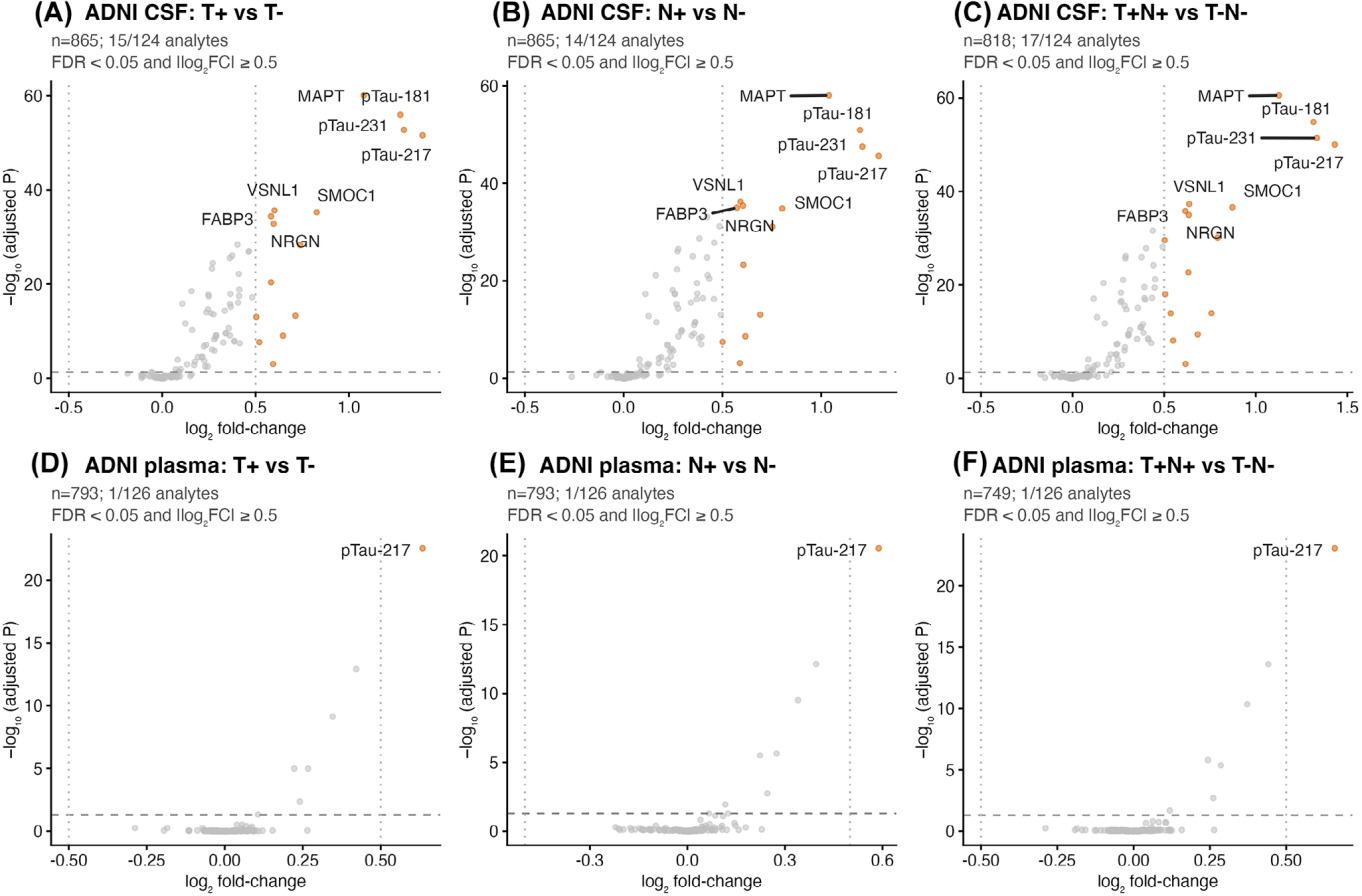
ADNI A^+^ ATN‐stratified differential abundance in CSF and plasma. (A–C) CSF contrasts: (A) T^+^ versus T^−^ (*n* = 865), (B) N^+^ versus N^−^ (*n* = 865), and (C) T^+^N^+^ versus T^−^N^−^ (*n* = 818); (D–F) corresponding plasma contrasts (*n* = 793, *n* = 793, and *n* = 749). Models tested 124 CSF and 126 plasma analytes. In both fluids, colored points met FDR < 0.05 and |log_2_FC| ≥ 0.5; gray points did not. Horizontal dashed and vertical dotted lines mark the two thresholds. ADNI, Alzheimer's Disease Neuroimaging Initiative; ATN, amyloid, tau pathology, and neurodegeneration; CSF, cerebrospinal fluid; FDR, false discovery rate.

**FIGURE 3:**
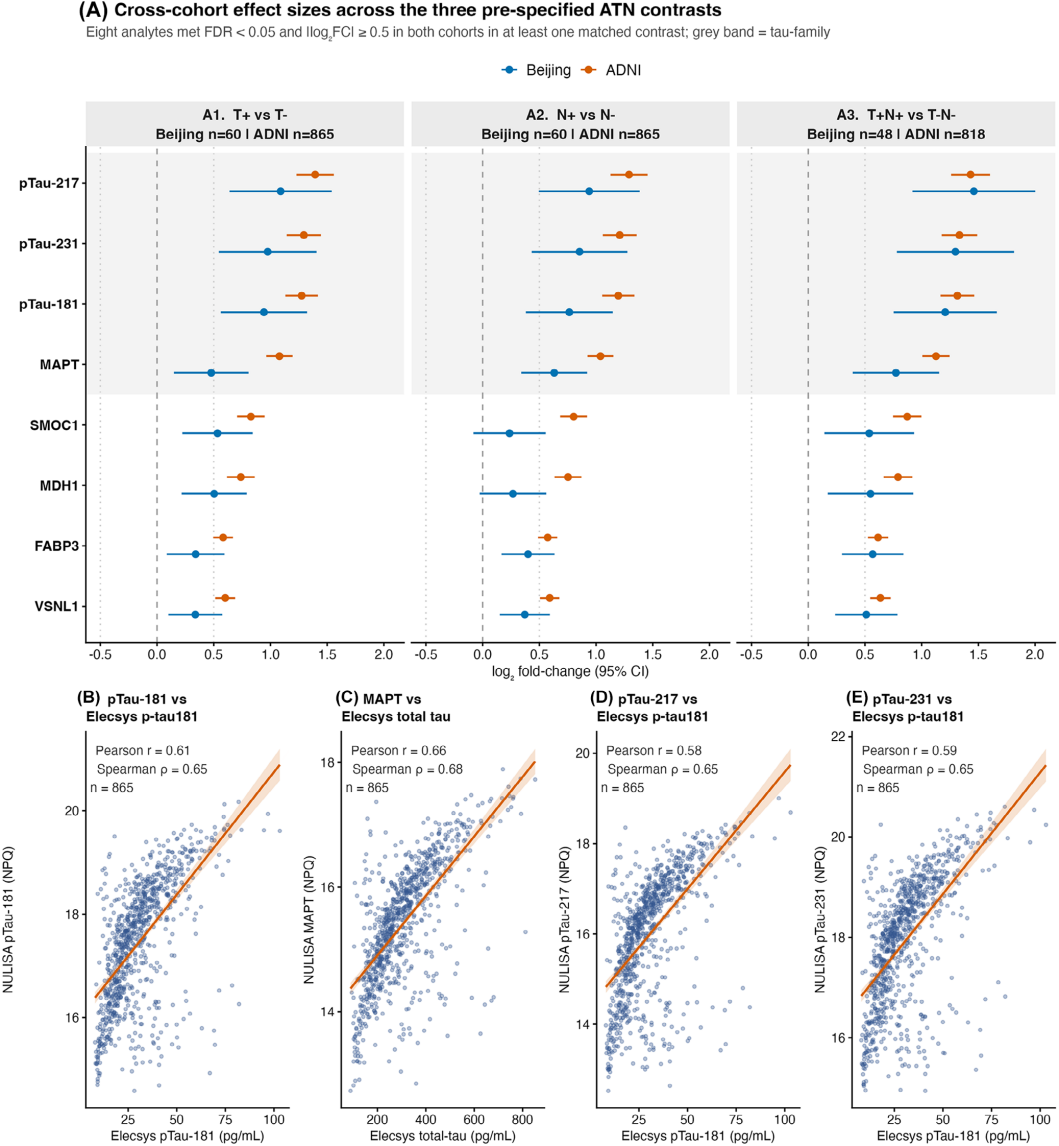
Cross‐cohort effect‐size comparison and cross‐platform tau correlations. (A) Beijing and ADNI estimates for eight proteins that met FDR < 0.05 and |log_2_FC| ≥ 0.5 in both cohorts in at least one matched contrast; all three contrast‐specific estimates are shown. Beijing/ADNI sample sizes were *n* = 60/865 for the T and N contrasts and *n* = 48/818 for concordant extremes. Points and bars show log_2_FC and model‐based 95% CIs; vertical lines mark zero and |log_2_FC| = 0.5. (B–E) correlations between participant‐matched NULISA and Elecsys CSF measurements in 865 ADNI A^+^ participants; 854 pairs had the same recorded collection date. Pearson r (raw Elecsys pg/ml scale) and Spearman ρ are shown with linear fits and 95% CIs. ADNI, Alzheimer's Disease Neuroimaging Initiative; CI, confidence interval; CSF, cerebrospinal fluid; FDR, false discovery rate.

For the four replicated non‐tau proteins, all estimates in the clinically matched AD‐only sensitivity analysis remained directionally concordant, but the 95% confidence interval (CIs) for all four non‐tau effects included the prespecified 0.5 magnitude cutoff (Table S1).

Among the 865 ADNI A^+^ participants, T and N classifications agreed in 94.6% (κ = 0.880; 42 T^+^N^−^ and 5 T^−^N^+^), and log‐transformed Elecsys p‐tau181 and total tau correlated at *r* = 0.986. After adjustment for same‐panel CSF NULISA pTau‐217, all 12 associations involving the four non‐tau proteins remained directionally positive and FDR‐supported but were attenuated (log_2_FC = 0.141–0.308); every individual model‐based 95% CI had an upper limit below 0.5 (maximum, 0.427). In matched subsets, adjustment for separately measured plasma NULISA pTau‐217 changed these estimates by −2.6% to +2.2%, whereas adjustment for continuous tau‐PET SUVR changed them by −11.4% to +1.5%. In the exploratory tau‐PET extreme‐tertile analysis (*n* = 128), pTau‐181 was the only analyte to meet both full‐panel thresholds. The four non‐tau estimates were 0.196–0.303, with wide 95% CIs (upper limits, 0.452–0.656), so this analysis did not resolve whether their effects reached the magnitude threshold (Table S2, Panel C). Full T–N agreement and tau‐adjustment results are provided in Table S2, Panels A, B, and G.

### 3.4 Tau‐family proteins as platform‐pathology anchors

In the concordant‐extreme contrast, pTau‐217, pTau‐231, pTau‐181, and MAPT had log_2_FC values of 1.43, 1.33, 1.31, and 1.13, respectively, with FDR < 5×10^−^^4^ ^8^ for each (Figure 3A). In paired ADNI CSF measurements, NULISA pTau‐181 and MAPT correlated with Elecsys p‐tau181 and total tau at Spearman ρ = 0.65 and 0.68, while NULISA pTau‐217 and pTau‐231 each correlated with Elecsys p‐tau181 at ρ = 0.65 (Figure 3B–E). In Beijing, the four NULISA tau‐family analytes correlated with their prespecified INNOTEST tau comparators at ρ = 0.75–0.85; together, the Beijing and ADNI findings supported cross‐platform concordance of the NULISA tau measurements.

### 3.5 Cognitive associations and incremental prediction in ADNI A^+^

In the time‐anchored CSF samples (MMSE *n* = 781, ADAS‐Cog13 *n* = 839, CDR‐SB *n* = 778), the mean absolute cognitive correlation was 0.35 for Elecsys p‐tau181 and 0.29–0.34 for the four NULISA tau‐family analytes. FABP3 had the largest estimate among the four replicated non‐tau proteins (0.24); NRGN was included as a reference analyte and was not part of the cross‐cohort consistent set (Figure 4).^19^, ^20^, ^21^, ^22^, ^23^

**FIGURE 4:**
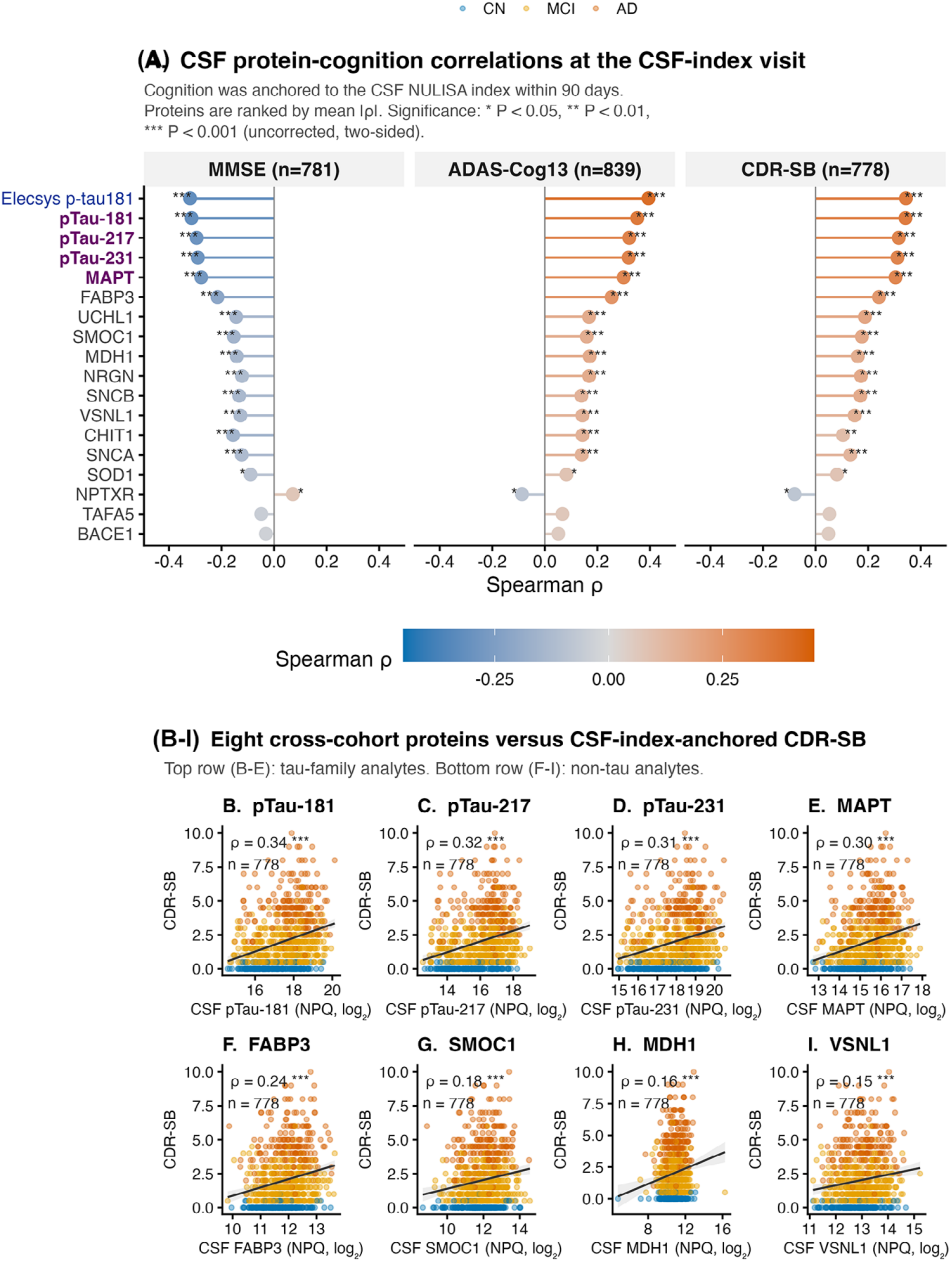
Time‐anchored CSF protein–cognition correlations in ADNI A^+^ participants. (A) Lollipop heatmap of Spearman correlations for 17 CSF NULISA analytes and Elecsys p‐tau181 with MMSE (*n* = 781), ADAS‐Cog13 (*n* = 839), and CDR‐SB (*n* = 778), each assessed within ± 90 days of the CSF draw. Proteins are ordered by mean |ρ|; tau‐family analytes are shown in purple, color encodes signed ρ, and asterisks denote nominal two‐sided *p*‐values (*p < 0.05, **p < 0.01, ***p < 0.001). (B–I) CDR‐SB scatter plots for the eight cross‐cohort proteins (*n* = 778). Points are colored by diagnosis at the CSF index; gray lines show overall linear fits with 95% CIs, and each panel reports Spearman ρ. ADAS‐Cog13, Alzheimer's Disease Assessment Scale–Cognitive Subscale, 13‐item version; ADNI, Alzheimer's Disease Neuroimaging Initiative; CDR‐SB, Clinical Dementia Rating – Sum of Boxes; CI, confidence interval; CSF, cerebrospinal fluid; MMSE, Mini‐Mental State Examination.

Within‐diagnosis correlations were smaller than pooled correlations, and approximately three quarters of the pooled rank covariance lay between the CN, MCI, and AD groups (median fractions, 72.5%, 73.7%, and 71.8% for the tau‐family, four‐protein, and NRGN sets, respectively; Table S2E). Ordered ATN‐stratum summaries and cognitive‐slope correlations are reported in Tables S3 and S4.

In the sensitivity analysis without adjustment for diagnosis, adding the four‐protein block after tau increased cross‐validated *R* ^2^ across all three cross‐sectional cognitive outcomes (+0.0101 to +0.0265), most clearly for ADAS‐Cog13. These increments attenuated in the diagnosis‐conditioned models. Across the five cognitive outcomes in these models, adding the tau block to the clinical/technical model increased cross‐validated *R* ^2^ by +0.0029 to +0.0250, whereas adding the four‐protein block to the same baseline changed *R* ^2^ by −0.0067 to +0.0029. After tau was included, the four‐protein Δ*R* ^2^ ranged from −0.0027 to +0.0032 for the cross‐sectional outcomes and was negative for both cognitive slopes; only the conditional percentile interval for CDR‐SB slope excluded zero, in the direction of lower out‐of‐fold performance. Complete estimates and conditional intervals are reported in Table S2, Panel D.

### 3.6 Plasma ATN‐stratified differential expression and cognitive correlations

In the ADNI A^+^ plasma subset, pTau‐217 was the only analyte that met both FDR < 0.05 and |log_2_FC| ≥ 0.5 in all three contrasts (Figure 2D–F). pTau‐231 and pTau‐181 remained FDR‐supported and directionally concordant but did not reach the magnitude threshold (log_2_FC = 0.396–0.442 and 0.340–0.372, respectively). pTau‐217 also ranked among the strongest plasma cognitive correlates, together with pTau‐231 and ACHE (Figure 5).

**FIGURE 5:**
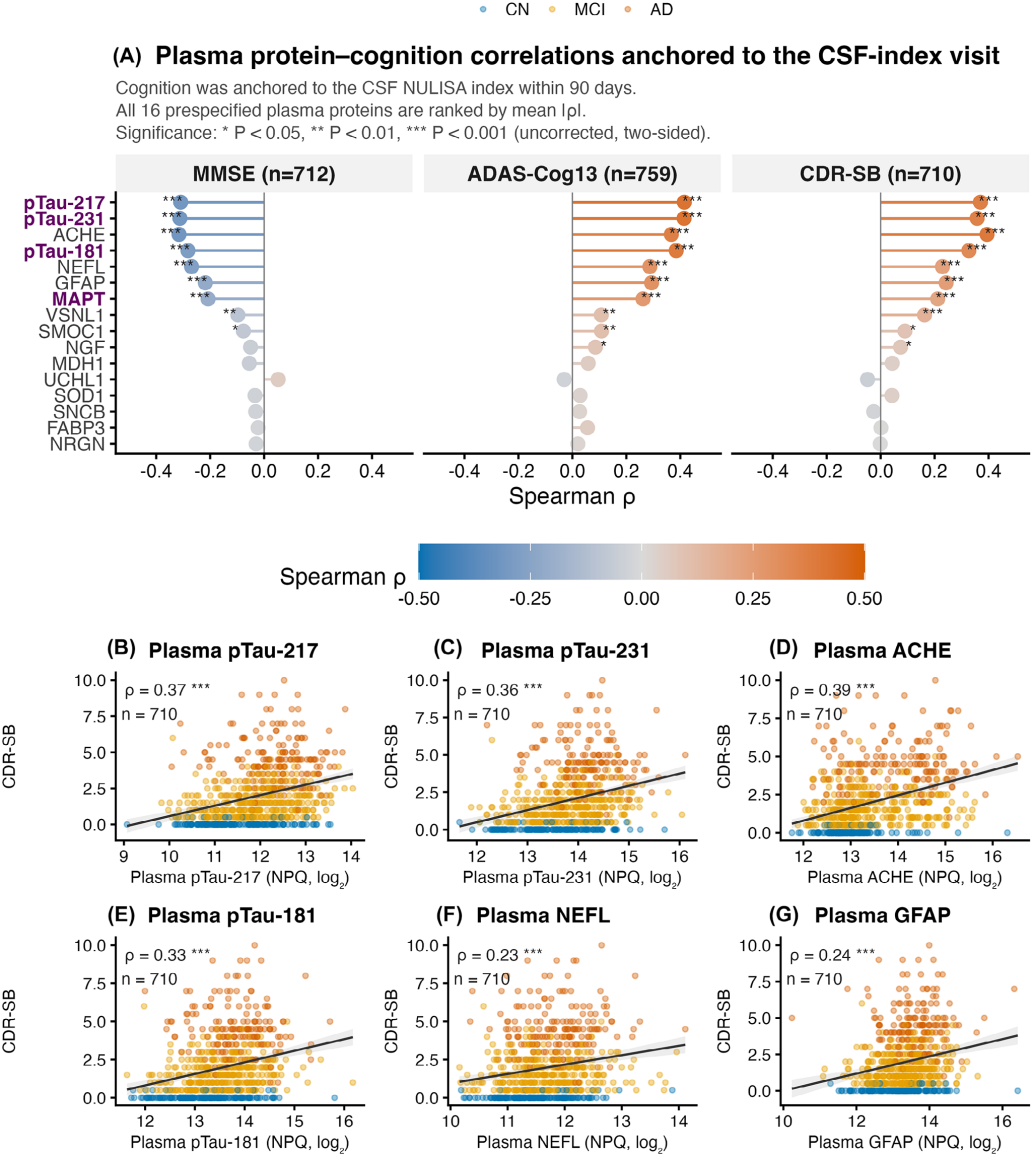
Time‐anchored plasma protein–cognition correlations in ADNI A^+^ participants. (A) Lollipop heatmap of Spearman correlations for 16 prespecified plasma NULISA proteins with MMSE (*n* = 712), ADAS‐Cog13 (*n* = 759), and CDR‐SB (*n* = 710), each assessed within ± 90 days of the CSF index; plasma sampling was not required on that date. Proteins are ordered by mean |ρ|; tau‐family analytes are shown in purple, color encodes signed ρ, and asterisks denote nominal two‐sided *p* values (*p < 0.05, **p < 0.01, ***p < 0.001). (B–G) CDR‐SB scatter plots for pTau‐217, pTau‐231, ACHE, pTau‐181, NEFL, and GFAP (*n* = 710). Points are colored by diagnosis at the CSF index, and gray lines show overall linear fits with 95% CIs. NEFL was selected by cognitive‐correlation rank, not the differential‐abundance threshold. ADAS‐Cog13, Alzheimer's Disease Assessment Scale–Cognitive Subscale, 13‐item version; ADNI, Alzheimer's Disease Neuroimaging Initiative; CDR‐SB, Clinical Dementia Rating – Sum of Boxes; CI, confidence interval; CSF, cerebrospinal fluid; GFAP, glial fibrillary acidic protein; MMSE, Mini‐Mental State Examination.

Among 785 participants with QC‐passed measurements in both fluids, the median plasma–CSF collection interval was 0 days; 512 pairs (65.2%) were collected on the same day and 715 (91.1%) within 30 days. Plasma pTau‐217 showed numerically stronger cognitive correlations than CSF pTau‐217 across the three outcomes. After Holm correction across the 15 interval‐window comparisons, only ADAS‐Cog13 showed a supported cross‐fluid difference in each of the five sampling‐window analyses, including the all‐pairs analysis (adjusted *p* = 0.0269). Results for the 30‐, 90‐, and 365‐day windows are reported in Table S2, Panel F. Table S5 reports the protein–cognition correlations in the full anchored samples and Table S6 the all‐pairs cross‐fluid comparisons; full differential‐abundance results are provided in Table S7.

## 4 DISCUSSION

This cross‐cohort NULISAseq study identified eight CSF proteins that met the cross‐cohort criterion in at least one matched contrast in Beijing and ADNI: four tau‐family analytes and four non‐tau proteins (MDH1, SMOC1, FABP3, and VSNL1). Each non‐tau protein met the criterion in one matched contrast, extending the replicated CSF profile beyond the tau family.

NULISA tau measurements showed cross‐platform concordance with the prespecified INNOTEST and Elecsys comparators. Using cohort‐established classifier assays kept the stratifying measurements separate from the NULISA discovery panel, and NULISA pTau‐217 was evaluated as an analyte and tau‐adjustment measure rather than used to define the groups. The marked concordance between T and N indicated that the three contrasts captured a common tau‐associated biomarker burden rather than independent biological axes; cross‐cohort replication therefore refers to recurrence from Beijing to ADNI within the same matched contrast.

FABP3 has been linked to AD pathology, tau burden, and cognitive decline,^19^, ^20^ and a large multi‐cohort CSF proteomic study also identified SMOC1, FABP3, and VSNL1.^21^ Tau‐adjustment results varied by measure. After adjustment for same‐panel CSF pTau‐217, the non‐tau associations retained their direction and FDR support, although the estimates fell below the prespecified magnitude threshold; separately measured plasma pTau‐217 and continuous tau‐PET SUVR produced smaller changes. Because CSF pTau‐217 and the candidate proteins were measured on the same panel, this analysis could not separate biological from assay covariance, and the sensitivity analyses did not establish tau‐independent effects.

Cross‐sectional correlations for the four‐protein set largely reflected differences between diagnostic groups. In sensitivity models that omitted diagnosis, adding the block after tau produced positive cross‐sectional Δ*R* ^2^ estimates, whereas the diagnosis‐conditioned models showed no stable out‐of‐sample increment beyond diagnosis and Elecsys tau. This pattern is consistent with stage‐related information that overlaps with established clinical and tau measures; its value for biological stratification requires external validation.

Plasma findings were dominated by pTau‐217, consistent with recent NULISA plasma studies^16^, ^17^ and broader work on plasma pTau‐217.^24^, ^25^ Although its cognitive correlations were numerically stronger than those of CSF pTau‐217, adjusted support for a cross‐fluid difference was limited to ADAS‐Cog13. The broader CSF profile and focused plasma signal therefore represented distinct fluid patterns. These observational comparisons cannot determine whether the observed differences arose from biological compartment, sampling time, assay performance, or participant characteristics.

Interpretation is limited by the small, single‐center Beijing cohort (*n* = 60), its Han Chinese composition, and the differing clinical spectra of the two cohorts. The clinically aligned AD‐only analysis retained directional concordance, but confidence intervals for the four non‐tau effects included the 0.5 cutoff, leaving their magnitude unresolved. Recurrence across different ATN assays and cutoffs supports reproducibility across classifier systems but precludes direct numerical comparison of T and N thresholds. The cognitive models used a fixed four‐protein set selected partly in ADNI and were evaluated using internal cross‐validation; diagnosis‐conditioned and diagnosis‐omitted models address different estimands, while the rank‐covariance decomposition was descriptive and the slope analyses were exploratory. Additional ADNI‐only or subthreshold findings included SFRP1 across all three ADNI CSF contrasts and associations involving glial fibrillary acidic protein (GFAP), NEFL, CHIT1, and ACHE; all require independent validation.^26^, ^27^, ^28^, ^29^

Across two amyloid‐positive cohorts, NULISAseq identified a tau‐anchored CSF signature that included four non‐tau proteins, whereas plasma findings were dominated by pTau‐217. External validation should determine whether these patterns improve biological stratification or prediction.

## AUTHOR CONTRIBUTIONS

Jianpeng Sheng and Jianping Jia conceived and supervised the study. Lan Ding performed the main data analyses, interpreted the results, and drafted the manuscript. Menghan Zheng contributed to data processing, statistical analysis, figure preparation, and manuscript revision. Simiao Wang, Wenying Liu, Yumei Liang, Siqi Xie, Ting Yang, Dandan Sheng, and Shuheng Wang contributed to participant recruitment, clinical data collection, sample organization, and data curation. Jianpeng Sheng and Jianping Jia provided critical scientific input and revised the manuscript. All authors reviewed and approved the final manuscript.

## CONFLICT OF INTEREST STATEMENT

The authors declare no competing interests. Author disclosures are available in the Supporting Information.

## CONSENT STATEMENT

The Beijing cohort study was approved by the Ethics Committee of Xuanwu Hospital, Capital Medical University (Approval No. 2019‐110). All Beijing participants or their legally authorized representatives provided written informed consent before enrollment, in accordance with the Declaration of Helsinki. ADNI was approved by the institutional review boards of all participating institutions, and written informed consent was obtained from all ADNI participants or their legally authorized representatives. The present analysis used de‐identified ADNI data under the ADNI Data Use Agreement. This manuscript does not contain identifiable individual‐level participant information.

## Supporting information




Supporting Information

Supporting Information

Supporting Information

Supporting Information

## Data Availability

De‐identified individual‐level Beijing NULISAseq NPQ values and ATN classification data will be made available to qualified researchers upon reasonable request to the corresponding authors, subject to institutional and ethical approvals. ADNI data are available to qualified investigators through the ADNI/LONI database under the ADNI Data Use Agreement.

## References

[1] Jack CR , Bennett DA , Blennow K , et al. NIA‐AA research framework: toward a biological definition of Alzheimer's disease. Alzheimers Dement. 2018;14:535‐562. doi: 10.1016/j.jalz.2018.02.018 PMC5958625 29653606

[2] Knopman DS , Amieva H , Petersen RC , et al. Alzheimer disease. Nat Rev, Dis Primers. 2021;7:33. doi: 10.1038/s41572-021-00269-y PMC8574196 33986301

[3] Tijms BM , Vromen EM , Mjaavatten O , et al. Cerebrospinal fluid proteomics in patients with Alzheimer's disease reveals five molecular subtypes with distinct genetic risk profiles. Nat Aging. 2024;4:33‐47. doi: 10.1038/s43587-023-00550-7 PMC10798889 38195725

[4] Western D , Timsina J , Wang L , Wang C , Yang C , Phillips B , et al. Proteogenomic analysis of human cerebrospinal fluid identifies neurologically relevant regulation and implicates causal proteins for Alzheimer's disease. Nat Genet. 2024;56:2672‐2684. doi: 10.1038/s41588-024-01972-8 PMC11831731 39528825

[5] Blennow K , Hampel H , Weiner M , Zetterberg H . Cerebrospinal fluid and plasma biomarkers in Alzheimer disease. Nat Rev, Neurol. 2010;6:131‐144. doi: 10.1038/nrneurol.2010.4 20157306

[6] Suárez‐Calvet M , Capell A , Morenas‐Rodríguez E , et al. CSF progranulin increases in the course of Alzheimer's disease and is associated with sTREM2, neurodegeneration and cognitive decline. EMBO Mol Med. 2018;10:e9712. doi: 10.15252/emmm.201809712 PMC6284390 30482868

[7] Vromen EM , de Leeuw DM , van Harten AC , et al. CSF proteomic profiles related to cognitive decline in MCI A+ depend on tau levels. Brain. 2025;148:4389‐4399. doi: 10.1093/brain/awaf251 PMC12677021 40676952

[8] Oh HS‐H , Urey DY , Karlsson L , et al. A cerebrospinal fluid synaptic protein biomarker for prediction of cognitive resilience versus decline in Alzheimer's disease. Nat Med. 2025;31:1592‐1603. doi: 10.1038/s41591-025-03565-2 PMC12092275 40164724

[9] Feng W , Beer JC , Hao Q , et al. NULISA: a proteomic liquid biopsy platform with attomolar sensitivity and high multiplexing. Nat Commun. 2023;14:7238. doi: 10.1038/s41467-023-42834-x PMC10636041 37945559

[10] Ashton NJ , Denkinger MN . NULISAseq 120‐plex CNS Disease Panel in ADNI plasma and CSF. Study Report P056 ADNI. Banner Sun Health Research Institute; 6 January 2026. ADNI/LONI Image and Data Archive.

[11] McKhann GM , Knopman DS , Chertkow H , et al. The diagnosis of dementia due to Alzheimer's disease: recommendations from the national institute on aging‐Alzheimer's association workgroups on diagnostic guidelines for Alzheimer's disease. Alzheimers Dement. 2011;7:263‐269. doi: 10.1016/j.jalz.2011.03.005 PMC3312024 21514250

[12] Sabri O , Sabbagh MN , Seibyl J , et al. Florbetaben PET imaging to detect amyloid beta plaques in Alzheimer's disease: phase 3 study. Alzheimers Dement. 2015;11:964‐974. doi: 10.1016/j.jalz.2015.02.004 25824567

[13] Vanderstichele H , De Vreese K , Blennow K , et al. Analytical performance and clinical utility of the INNOTEST PHOSPHO‐TAU181P assay for discrimination between Alzheimer's disease and dementia with lewy bodies. Clin Chem Lab Med. 2006;44:1472‐1480. doi: 10.1515/CCLM.2006.258 17163825

[14] Hansson O , Seibyl J , Stomrud E , et al. CSF biomarkers of Alzheimer's disease concord with amyloid‐β PET and predict clinical progression: a study of fully automated immunoassays in BioFINDER and ADNI cohorts. Alzheimer's Dement. 2018;14:1470‐1481. doi: 10.1016/j.jalz.2018.01.010 PMC6119541 29499171

[15] Ritchie ME , Phipson B , Wu D , et al. limma powers differential expression analyses for RNA‐sequencing and microarray studies. Nucleic Acids Res. 2015;43:e47. doi: 10.1093/nar/gkv007 PMC4402510 25605792

[16] Rea Reyes RE , Wilson RE , Langhough RE , et al. Targeted proteomic biomarker profiling using NULISA in a cohort enriched with risk for Alzheimer's disease and related dementias. Alzheimers Dement. 2025;21:e70166. doi: 10.1002/alz.70166 PMC12046973 40318118

[17] Zeng X , Sehrawat A , Lafferty TK , et al. Novel plasma biomarkers of amyloid plaque pathology and cortical thickness: evaluation of the NULISA targeted proteomic platform in an ethnically diverse cohort. Alzheimers Dement. 2025;21:e14535. doi: 10.1002/alz.14535 PMC11848535 39989429

[18] Steiger JH . Tests for comparing elements of a correlation matrix. Psychol Bull. 1980;87:245‐251. doi: 10.1037/0033-2909.87.2.245

[19] Chiasserini D , Biscetti L , Eusebi P , et al. Differential role of CSF fatty acid binding protein 3, α‐synuclein, and Alzheimer's disease core biomarkers in lewy body disorders and Alzheimer's dementia. Alzheimer's Res Ther. 2017;9:52. doi: 10.1186/s13195-017-0276-4 PMC5532764 28750675

[20] Olsson B , Lautner R , Andreasson U , et al. CSF and blood biomarkers for the diagnosis of Alzheimer's disease: a systematic review and meta‐analysis. Lancet, Neurol. 2016;15:673‐684. doi: 10.1016/S1474-4422(16)00070-3 27068280

[21] Ali M , Timsina J , Western D , et al. Multi‐cohort cerebrospinal fluid proteomics identifies robust molecular signatures across the Alzheimer disease continuum. Neuron. 2025;113:1363‐1379.e9. doi: 10.1016/j.neuron.2025.02.014 PMC12067247 40088886

[22] Kvartsberg H , Duits FH , Ingelsson M , et al. Cerebrospinal fluid levels of the synaptic protein neurogranin correlates with cognitive decline in prodromal Alzheimer's disease. Alzheimers Dement. 2015;11:1180‐1190. doi: 10.1016/j.jalz.2014.10.009 25533203

[23] Kvartsberg H , Portelius E , Andreasson U , et al. Characterization of the postsynaptic protein neurogranin in paired cerebrospinal fluid and plasma samples from Alzheimer's disease patients and healthy controls. Alzheimer's Res Ther. 2015;7:40. doi: 10.1186/s13195-015-0124-3 PMC4487851 26136856

[24] Schindler SE , Petersen KK , Saef B , et al. Head‐to‐head comparison of leading blood tests for Alzheimer's disease pathology. Alzheimers Dement. 2024;20:8074‐8096. doi: 10.1002/alz.14315 PMC11567821 39394841

[25] Brum WS , Cullen NC , Janelidze S , et al. A two‐step workflow based on plasma p‐tau217 to screen for amyloid β positivity with further confirmatory testing only in uncertain cases. Nat Aging. 2023;3:1079‐1090. doi: 10.1038/s43587-023-00471-5 PMC10501903 37653254

[26] Pereira JB , Janelidze S , Smith R , et al. Plasma GFAP is an early marker of amyloid‐β but not tau pathology in Alzheimer's disease. Brain: J Neurol. 2021;144:3505‐3516. doi: 10.1093/brain/awab223 PMC8677538 34259835

[27] Mattsson N , Andreasson U , Zetterberg H , Blennow K , Alzheimer's Disease Neuroimaging Initiative . Association of plasma neurofilament light with neurodegeneration in patients with Alzheimer disease. JAMA Neurol. 2017;74:557‐566. doi: 10.1001/jamaneurol.2016.6117 PMC5822204 28346578

[28] Watabe‐Rudolph M , Song Z , Lausser L , et al. Chitinase enzyme activity in CSF is a powerful biomarker of Alzheimer disease. Neurology. 2012;78:569‐577. doi: 10.1212/WNL.0b013e318247caa1 22323746

[29] García‐Ayllón M‐S , Small DH , Avila J , Sáez‐Valero J . Revisiting the role of acetylcholinesterase in Alzheimer's disease: cross‐talk with P‐tau and β‐amyloid. Front Mol Neurosci. 2011;4:22. doi: 10.3389/fnmol.2011.00022 PMC3171929 21949503

