# Supplementary material for: Cross‐cohort NULISAseq identifies tau‐anchored CSF and plasma‐specific signatures across the amyloid‐positive Alzheimer's disease
Source: Alzheimers Dement. 2026 Oct 12;22(10):e71915. doi: 10.1002/alz.71915 (PMC13656197; doi:10.1002/alz.71915)
Supplement: Supplementary file 2 [file ALZ-22-e71915-s002.docx]

**Supplementary Table S2. Tau-adjustment sensitivity analyses, diagnosis-conditioned and diagnosis-omitted model performance, diagnosis-stratified protein–cognition correlations and rank-covariance decomposition, and CSF–plasma sampling intervals.**

**Legend**

| **Item** | **Description** |
| --- | --- |
| Supplementary Table S2. | Supplementary Table S2 reports the analyses summarized in Panels A–G. Panels A-C and G examine the close relation between operational ATN classification and tau measures. Panel D reports the primary nested analysis, same-sample four-model comparisons, and diagnosis-omitted sensitivity analyses. Panel E reports pooled and diagnosis-specific cognitive correlations plus an exact descriptive rank-covariance decomposition. Panel F reports sampling intervals and dependent CSF-plasma correlation comparisons. Panels B-G use the ADNI A+ cohort; Panel A additionally reports the corresponding Beijing classification statistics. |
| Panel A | Overlap between T and N status in the classifying immunoassays, and the correlation between the two classifying Elecsys analytes (Pearson r on the log scale). Beijing values, classified with Fujirebio INNOTEST, are shown for contrast. |
| Panel B | The three ATN contrasts were refitted with NULISA pTau-217 as a continuous covariate using the covariates of Methods 2.5. An Elecsys analyte was not used because those analytes define the groups. Panel B reports two-sided moderated-t 95% confidence intervals from limma for every estimable unadjusted and adjusted log2 fold-change. The prespecified 0.5 threshold is a descriptive classification rule, not a hypothesis test about effect magnitude. Because pTau-217 and the candidate proteins were measured on the same panel, adjustment may absorb shared assay-platform or other correlated measurement variation as well as biological covariation. The attenuation therefore cannot be attributed solely to biological tau burden and establishes neither tau independence nor full tau dependence. |
| Panel C | T was redefined using the UC Berkeley flortaucipir (FTP/AV-1451) meta-temporal composite SUVR nearest the CSF draw within ±365 days. The continuous-SUVR adjustment is reported in Panel G. The extreme-tertile contrast included 128 participants (64 per group; SUVR cut points 1.1983 and 1.3517) and used the covariates of Methods 2.5. Benjamini-Hochberg correction across the 124-analyte CSF family was primary; the candidate-focused q was secondary and not preregistered. Only pTau-181 met both thresholds. The limited positive-control pattern and wide non-tau intervals made this analysis non-identifying rather than evidence for or against tau independence. |
| Panel D | Panel D1 is the primary nested comparison: the four-protein block was added after age, sex, education, APOE e4, NULISA plate, diagnosis, and log-transformed Elecsys p-tau181 and total tau; slope models also included baseline cognition. Panels D2-D3 give exploratory diagnosis-conditioned four-model performance and direct non-nested four-protein-versus-tau comparisons. Panels D4-D5 repeat the same-sample, same-fold framework after diagnosis was removed from every model, thereby changing the estimand. Panel D6 reports the corresponding full-sample in-sample increments without cross-validation. Cross-validation used 10 repeats of balanced five-fold splits. For Panel D1, the q/FDR column contains Holm-adjusted p values across the five outcome-specific primary block tests. Percentile intervals use 5,000 participant resamples with fixed out-of-fold predictions and are descriptive conditional intervals; they do not include refitting or feature-selection uncertainty. No equivalence or non-inferiority margin was prespecified, and feature selection was not independently validated. |
| Panel E | Panel E1 reports pooled, diagnosis-specific Fisher-z-combined, and diagnosis-adjusted protein-cognition correlations using cognition within ±90 days of the CSF draw. Panel E2 writes the pooled global-rank cross-product exactly as the sum of within- and between-diagnosis components. The between/total fraction is a descriptive decomposition of observed Spearman rank covariance; it is neither a causal attributable fraction nor a decomposition of R2. Panel E results are interpreted descriptively. |
| Panel F | Panel F reports plasma-minus-CSF sampling intervals, same-analyte cross-fluid correlations by interval window, and Steiger (1980) tests comparing dependent CSF and plasma pTau-217 correlations with cognition. Correlations are Pearson correlations of midranks, equivalent to Spearman rho. The q/FDR column contains Holm-adjusted p values across all 15 outcome-by-window Steiger comparisons. Same-day subsets are nested sensitivity analyses, not independent validation, and the findings are not attributed solely to fluid biology or sampling time. |
| Panel G | Panel G repeats Panel B using plasma NULISA pTau-217 (n=785 for the T and N contrasts; n=741 for the concordant-extreme contrast) and tau-PET meta-temporal SUVR (n=192 and n=183, respectively), neither measured on the CSF assay plate. Because these covariates differ in matrix, biological target, and correlation with the classifying Elecsys measures, differences in attenuation cannot isolate biological tau burden from correlated measurement structure. |

*Numeric values are displayed to eight significant digits; analyses used unrounded values.*

**A. T/N overlap in the classifying immunoassays**

| **Panel** | **Analysis** | **Item** | **Stratum / contrast / outcome** | **Class** | **Estimate** | **SE** | **CI_low** | **CI_high** | **P** | **q or FDR** | **n** | **Note** |
| --- | --- | --- | --- | --- | --- | --- | --- | --- | --- | --- | --- | --- |
| A. T/N overlap in the classifying immunoassays | Descriptive | ADNI A+ cross-tabulation |  |  | T-N- n=275; T+N- n=42; T-N+ n=5; T+N+ n=543 |  |  |  |  |  |  |  |
| A. T/N overlap in the classifying immunoassays | Descriptive | Cohen's kappa (ADNI, cutpoints as used) |  |  | 0.880 |  |  |  |  |  |  |  |
| A. T/N overlap in the classifying immunoassays | Descriptive | Raw agreement (ADNI) |  |  | 94.6% |  |  |  |  |  |  |  |
| A. T/N overlap in the classifying immunoassays | Descriptive | Elecsys p-tau181 vs total tau, Pearson r (log scale) |  |  | 0.986 |  |  |  |  |  |  |  |
| A. T/N overlap in the classifying immunoassays | Descriptive | Beijing: INNOTEST p-tau vs t-tau, Spearman rho |  |  | 0.704 |  |  |  |  |  |  |  |
| A. T/N overlap in the classifying immunoassays | Descriptive | Beijing: Cohen's kappa / agreement |  |  | 0.53 / 80.0% |  |  |  |  |  |  |  |

**B. ATN contrasts adjusted for continuous NULISA pTau-217**

| **Panel** | **Analysis** | **Item** | **Stratum / contrast / outcome** | **Class** | **Estimate** | **SE** | **CI_low** | **CI_high** | **P** | **q or FDR** | **n** | **Note** |
| --- | --- | --- | --- | --- | --- | --- | --- | --- | --- | --- | --- | --- |
| B. ATN contrasts adjusted for continuous NULISA pTau-217 | unadjusted | pTau-217 | T+ vs T- | tau-family | 1.3941459 | 0.083948245 | 1.2293742 | 1.5589177 | 8.294615e-54 | 2.5713306e-52 | 865 | log2 fold-change with two-sided moderated-t 95% CI from limma |
| B. ATN contrasts adjusted for continuous NULISA pTau-217 | adjusted for NULISA pTau-217 | pTau-217 | T+ vs T- | tau-family |  |  |  |  |  |  | 865 | N/A: this analyte is the adjustment covariate, so the model is a self-adjustment and yields no interpretable estimate |
| B. ATN contrasts adjusted for continuous NULISA pTau-217 | unadjusted | pTau-231 | T+ vs T- | tau-family | 1.294179 | 0.076860015 | 1.1433198 | 1.4450381 | 4.5138335e-55 | 1.8657179e-53 | 865 | log2 fold-change with two-sided moderated-t 95% CI from limma |
| B. ATN contrasts adjusted for continuous NULISA pTau-217 | adjusted for NULISA pTau-217 | pTau-231 | T+ vs T- | tau-family | 0.029172874 | 0.011868217 | 0.0058781313 | 0.052467617 | 0.014169021 | 0.049865491 | 865 | Adjusted log2 fold-change with two-sided moderated-t 95% CI from limma. Because pTau-217 was measured on the same panel, attenuation may reflect biological covariation and shared assay-platform or other correlated measurement variation. |
| B. ATN contrasts adjusted for continuous NULISA pTau-217 | unadjusted | pTau-181 | T+ vs T- | tau-family | 1.2750754 | 0.073069118 | 1.1316569 | 1.4184938 | 1.8306921e-58 | 1.1350291e-56 | 865 | log2 fold-change with two-sided moderated-t 95% CI from limma |
| B. ATN contrasts adjusted for continuous NULISA pTau-217 | adjusted for NULISA pTau-217 | pTau-181 | T+ vs T- | tau-family | 0.083440602 | 0.015928551 | 0.0521763 | 0.1147049 | 2.047495e-07 | 1.2694469e-05 | 865 | Adjusted log2 fold-change with two-sided moderated-t 95% CI from limma. Because pTau-217 was measured on the same panel, attenuation may reflect biological covariation and shared assay-platform or other correlated measurement variation. |
| B. ATN contrasts adjusted for continuous NULISA pTau-217 | unadjusted | MAPT | T+ vs T- | tau-family | 1.0791669 | 0.059184823 | 0.96300019 | 1.1953335 | 6.9196652e-63 | 8.5803848e-61 | 865 | log2 fold-change with two-sided moderated-t 95% CI from limma |
| B. ATN contrasts adjusted for continuous NULISA pTau-217 | adjusted for NULISA pTau-217 | MAPT | T+ vs T- | tau-family | 0.16704641 | 0.025412568 | 0.11716703 | 0.21692578 | 8.6155935e-11 | 1.0683336e-08 | 865 | Adjusted log2 fold-change with two-sided moderated-t 95% CI from limma. Because pTau-217 was measured on the same panel, attenuation may reflect biological covariation and shared assay-platform or other correlated measurement variation. |
| B. ATN contrasts adjusted for continuous NULISA pTau-217 | unadjusted | SMOC1 | T+ vs T- | non-tau | 0.82749358 | 0.061726628 | 0.70633792 | 0.94864924 | 2.7170666e-37 | 5.6152711e-36 | 865 | log2 fold-change with two-sided moderated-t 95% CI from limma |
| B. ATN contrasts adjusted for continuous NULISA pTau-217 | adjusted for NULISA pTau-217 | SMOC1 | T+ vs T- | non-tau | 0.16951568 | 0.054563905 | 0.062418536 | 0.27661283 | 0.001955062 | 0.0098456935 | 865 | Adjusted log2 fold-change with two-sided moderated-t 95% CI from limma. Because pTau-217 was measured on the same panel, attenuation may reflect biological covariation and shared assay-platform or other correlated measurement variation. |
| B. ATN contrasts adjusted for continuous NULISA pTau-217 | unadjusted | MDH1 | T+ vs T- | non-tau | 0.73870623 | 0.062284434 | 0.61645572 | 0.86095674 | 4.04045e-30 | 5.010158e-29 | 865 | log2 fold-change with two-sided moderated-t 95% CI from limma |
| B. ATN contrasts adjusted for continuous NULISA pTau-217 | adjusted for NULISA pTau-217 | MDH1 | T+ vs T- | non-tau | 0.2457142 | 0.063120769 | 0.12182178 | 0.36960662 | 0.00010694085 | 0.0015730124 | 865 | Adjusted log2 fold-change with two-sided moderated-t 95% CI from limma. Because pTau-217 was measured on the same panel, attenuation may reflect biological covariation and shared assay-platform or other correlated measurement variation. |
| B. ATN contrasts adjusted for continuous NULISA pTau-217 | unadjusted | FABP3 | T+ vs T- | non-tau | 0.58270671 | 0.044095922 | 0.49615621 | 0.66925721 | 2.2541098e-36 | 3.9929944e-35 | 865 | log2 fold-change with two-sided moderated-t 95% CI from limma |
| B. ATN contrasts adjusted for continuous NULISA pTau-217 | adjusted for NULISA pTau-217 | FABP3 | T+ vs T- | non-tau | 0.14118059 | 0.040538594 | 0.061612102 | 0.22074909 | 0.00052209575 | 0.0033449078 | 865 | Adjusted log2 fold-change with two-sided moderated-t 95% CI from limma. Because pTau-217 was measured on the same panel, attenuation may reflect biological covariation and shared assay-platform or other correlated measurement variation. |
| B. ATN contrasts adjusted for continuous NULISA pTau-217 | unadjusted | VSNL1 | T+ vs T- | non-tau | 0.60097716 | 0.044514138 | 0.51360579 | 0.68834852 | 9.4268818e-38 | 2.3378667e-36 | 865 | log2 fold-change with two-sided moderated-t 95% CI from limma |
| B. ATN contrasts adjusted for continuous NULISA pTau-217 | adjusted for NULISA pTau-217 | VSNL1 | T+ vs T- | non-tau | 0.19872472 | 0.043038733 | 0.11424899 | 0.28320045 | 4.4929554e-06 | 0.00013928162 | 865 | Adjusted log2 fold-change with two-sided moderated-t 95% CI from limma. Because pTau-217 was measured on the same panel, attenuation may reflect biological covariation and shared assay-platform or other correlated measurement variation. |
| B. ATN contrasts adjusted for continuous NULISA pTau-217 | unadjusted | pTau-217 | N+ vs N- | tau-family | 1.290792 | 0.083293917 | 1.1273045 | 1.4542794 | 7.4329679e-48 | 2.30422e-46 | 865 | log2 fold-change with two-sided moderated-t 95% CI from limma |
| B. ATN contrasts adjusted for continuous NULISA pTau-217 | adjusted for NULISA pTau-217 | pTau-217 | N+ vs N- | tau-family |  |  |  |  |  |  | 865 | N/A: this analyte is the adjustment covariate, so the model is a self-adjustment and yields no interpretable estimate |
| B. ATN contrasts adjusted for continuous NULISA pTau-217 | unadjusted | pTau-231 | N+ vs N- | tau-family | 1.2085819 | 0.076101351 | 1.0592118 | 1.3579519 | 6.8429867e-50 | 2.8284345e-48 | 865 | log2 fold-change with two-sided moderated-t 95% CI from limma |
| B. ATN contrasts adjusted for continuous NULISA pTau-217 | adjusted for NULISA pTau-217 | pTau-231 | N+ vs N- | tau-family | 0.0394403 | 0.011360384 | 0.017142322 | 0.061738278 | 0.00054340802 | 0.0021736321 | 865 | Adjusted log2 fold-change with two-sided moderated-t 95% CI from limma. Because pTau-217 was measured on the same panel, attenuation may reflect biological covariation and shared assay-platform or other correlated measurement variation. |
| B. ATN contrasts adjusted for continuous NULISA pTau-217 | unadjusted | pTau-181 | N+ vs N- | tau-family | 1.1960905 | 0.072317691 | 1.0541469 | 1.3380341 | 1.9429771e-53 | 1.2046458e-51 | 865 | log2 fold-change with two-sided moderated-t 95% CI from limma |
| B. ATN contrasts adjusted for continuous NULISA pTau-217 | adjusted for NULISA pTau-217 | pTau-181 | N+ vs N- | tau-family | 0.094759825 | 0.015201649 | 0.064922275 | 0.12459737 | 7.2028149e-10 | 2.9771635e-08 | 865 | Adjusted log2 fold-change with two-sided moderated-t 95% CI from limma. Because pTau-217 was measured on the same panel, attenuation may reflect biological covariation and shared assay-platform or other correlated measurement variation. |
| B. ATN contrasts adjusted for continuous NULISA pTau-217 | unadjusted | MAPT | N+ vs N- | tau-family | 1.0387359 | 0.058098802 | 0.92470082 | 1.1527709 | 7.15861e-61 | 8.8766764e-59 | 865 | log2 fold-change with two-sided moderated-t 95% CI from limma |
| B. ATN contrasts adjusted for continuous NULISA pTau-217 | adjusted for NULISA pTau-217 | MAPT | N+ vs N- | tau-family | 0.20057192 | 0.024057089 | 0.15335306 | 0.24779078 | 3.0779096e-16 | 3.8166079e-14 | 865 | Adjusted log2 fold-change with two-sided moderated-t 95% CI from limma. Because pTau-217 was measured on the same panel, attenuation may reflect biological covariation and shared assay-platform or other correlated measurement variation. |
| B. ATN contrasts adjusted for continuous NULISA pTau-217 | unadjusted | SMOC1 | N+ vs N- | non-tau | 0.80237672 | 0.060349834 | 0.6839234 | 0.92083004 | 9.2331667e-37 | 1.4311408e-35 | 865 | log2 fold-change with two-sided moderated-t 95% CI from limma |
| B. ATN contrasts adjusted for continuous NULISA pTau-217 | adjusted for NULISA pTau-217 | SMOC1 | N+ vs N- | non-tau | 0.19849037 | 0.052269025 | 0.095897575 | 0.30108316 | 0.00015666824 | 0.00074718701 | 865 | Adjusted log2 fold-change with two-sided moderated-t 95% CI from limma. Because pTau-217 was measured on the same panel, attenuation may reflect biological covariation and shared assay-platform or other correlated measurement variation. |
| B. ATN contrasts adjusted for continuous NULISA pTau-217 | unadjusted | MDH1 | N+ vs N- | non-tau | 0.75263473 | 0.060354447 | 0.63417236 | 0.87109711 | 7.0162736e-33 | 7.9092538e-32 | 865 | log2 fold-change with two-sided moderated-t 95% CI from limma |
| B. ATN contrasts adjusted for continuous NULISA pTau-217 | adjusted for NULISA pTau-217 | MDH1 | N+ vs N- | non-tau | 0.30849534 | 0.060247562 | 0.1902424 | 0.42674827 | 3.7787758e-07 | 5.2063134e-06 | 865 | Adjusted log2 fold-change with two-sided moderated-t 95% CI from limma. Because pTau-217 was measured on the same panel, attenuation may reflect biological covariation and shared assay-platform or other correlated measurement variation. |
| B. ATN contrasts adjusted for continuous NULISA pTau-217 | unadjusted | FABP3 | N+ vs N- | non-tau | 0.57341557 | 0.042979006 | 0.48905733 | 0.65777382 | 5.5289897e-37 | 9.7942103e-36 | 865 | log2 fold-change with two-sided moderated-t 95% CI from limma |
| B. ATN contrasts adjusted for continuous NULISA pTau-217 | adjusted for NULISA pTau-217 | FABP3 | N+ vs N- | non-tau | 0.1701092 | 0.038781048 | 0.093990391 | 0.24622801 | 1.2984272e-05 | 9.4708808e-05 | 865 | Adjusted log2 fold-change with two-sided moderated-t 95% CI from limma. Because pTau-217 was measured on the same panel, attenuation may reflect biological covariation and shared assay-platform or other correlated measurement variation. |
| B. ATN contrasts adjusted for continuous NULISA pTau-217 | unadjusted | VSNL1 | N+ vs N- | non-tau | 0.59125703 | 0.043385705 | 0.50610053 | 0.67641354 | 2.2714298e-38 | 5.6331458e-37 | 865 | log2 fold-change with two-sided moderated-t 95% CI from limma |
| B. ATN contrasts adjusted for continuous NULISA pTau-217 | adjusted for NULISA pTau-217 | VSNL1 | N+ vs N- | non-tau | 0.22287982 | 0.041153366 | 0.14210466 | 0.30365497 | 7.9624978e-08 | 2.2039707e-06 | 865 | Adjusted log2 fold-change with two-sided moderated-t 95% CI from limma. Because pTau-217 was measured on the same panel, attenuation may reflect biological covariation and shared assay-platform or other correlated measurement variation. |
| B. ATN contrasts adjusted for continuous NULISA pTau-217 | unadjusted | pTau-217 | T+N+ vs T-N- | tau-family | 1.4311194 | 0.087328236 | 1.259699 | 1.6025399 | 3.0761225e-52 | 9.5359798e-51 | 818 | log2 fold-change with two-sided moderated-t 95% CI from limma |
| B. ATN contrasts adjusted for continuous NULISA pTau-217 | adjusted for NULISA pTau-217 | pTau-217 | T+N+ vs T-N- | tau-family |  |  |  |  |  |  | 818 | N/A: this analyte is the adjustment covariate, so the model is a self-adjustment and yields no interpretable estimate |
| B. ATN contrasts adjusted for continuous NULISA pTau-217 | unadjusted | pTau-231 | T+N+ vs T-N- | tau-family | 1.333167 | 0.07993769 | 1.1762538 | 1.4900802 | 8.5724961e-54 | 3.5432984e-52 | 818 | log2 fold-change with two-sided moderated-t 95% CI from limma |
| B. ATN contrasts adjusted for continuous NULISA pTau-217 | adjusted for NULISA pTau-217 | pTau-231 | T+N+ vs T-N- | tau-family | 0.034704193 | 0.012296533 | 0.010566706 | 0.05884168 | 0.0048873868 | 0.017315313 | 818 | Adjusted log2 fold-change with two-sided moderated-t 95% CI from limma. Because pTau-217 was measured on the same panel, attenuation may reflect biological covariation and shared assay-platform or other correlated measurement variation. |
| B. ATN contrasts adjusted for continuous NULISA pTau-217 | unadjusted | pTau-181 | T+N+ vs T-N- | tau-family | 1.314871 | 0.075826191 | 1.1660285 | 1.4637136 | 2.1254556e-57 | 1.3177825e-55 | 818 | log2 fold-change with two-sided moderated-t 95% CI from limma |
| B. ATN contrasts adjusted for continuous NULISA pTau-217 | adjusted for NULISA pTau-217 | pTau-181 | T+N+ vs T-N- | tau-family | 0.093688432 | 0.016252669 | 0.061785247 | 0.12559162 | 1.1714183e-08 | 7.2627932e-07 | 818 | Adjusted log2 fold-change with two-sided moderated-t 95% CI from limma. Because pTau-217 was measured on the same panel, attenuation may reflect biological covariation and shared assay-platform or other correlated measurement variation. |
| B. ATN contrasts adjusted for continuous NULISA pTau-217 | unadjusted | MAPT | T+N+ vs T-N- | tau-family | 1.1262239 | 0.061150523 | 1.0061888 | 1.2462589 | 2.2010591e-63 | 2.7293133e-61 | 818 | log2 fold-change with two-sided moderated-t 95% CI from limma |
| B. ATN contrasts adjusted for continuous NULISA pTau-217 | adjusted for NULISA pTau-217 | MAPT | T+N+ vs T-N- | tau-family | 0.19473229 | 0.026087316 | 0.14352418 | 0.24594041 | 2.1947699e-13 | 2.7215147e-11 | 818 | Adjusted log2 fold-change with two-sided moderated-t 95% CI from limma. Because pTau-217 was measured on the same panel, attenuation may reflect biological covariation and shared assay-platform or other correlated measurement variation. |
| B. ATN contrasts adjusted for continuous NULISA pTau-217 | unadjusted | SMOC1 | T+N+ vs T-N- | non-tau | 0.87267234 | 0.063567427 | 0.74789304 | 0.99745164 | 1.1799311e-38 | 2.4385243e-37 | 818 | log2 fold-change with two-sided moderated-t 95% CI from limma |
| B. ATN contrasts adjusted for continuous NULISA pTau-217 | adjusted for NULISA pTau-217 | SMOC1 | T+N+ vs T-N- | non-tau | 0.19306875 | 0.055752101 | 0.083630125 | 0.30250737 | 0.00056275557 | 0.0027912676 | 818 | Adjusted log2 fold-change with two-sided moderated-t 95% CI from limma. Because pTau-217 was measured on the same panel, attenuation may reflect biological covariation and shared assay-platform or other correlated measurement variation. |
| B. ATN contrasts adjusted for continuous NULISA pTau-217 | unadjusted | MDH1 | T+N+ vs T-N- | non-tau | 0.79145752 | 0.064351619 | 0.66513889 | 0.91777615 | 5.9006334e-32 | 7.3167854e-31 | 818 | log2 fold-change with two-sided moderated-t 95% CI from limma |
| B. ATN contrasts adjusted for continuous NULISA pTau-217 | adjusted for NULISA pTau-217 | MDH1 | T+N+ vs T-N- | non-tau | 0.28718883 | 0.0654057 | 0.15880067 | 0.41557699 | 1.2814207e-05 | 0.00018311578 | 818 | Adjusted log2 fold-change with two-sided moderated-t 95% CI from limma. Because pTau-217 was measured on the same panel, attenuation may reflect biological covariation and shared assay-platform or other correlated measurement variation. |
| B. ATN contrasts adjusted for continuous NULISA pTau-217 | unadjusted | FABP3 | T+N+ vs T-N- | non-tau | 0.61552486 | 0.045430708 | 0.52634693 | 0.7047028 | 8.6931434e-38 | 1.5399283e-36 | 818 | log2 fold-change with two-sided moderated-t 95% CI from limma |
| B. ATN contrasts adjusted for continuous NULISA pTau-217 | adjusted for NULISA pTau-217 | FABP3 | T+N+ vs T-N- | non-tau | 0.16085767 | 0.041614721 | 0.079170033 | 0.24254532 | 0.00011995022 | 0.00082632371 | 818 | Adjusted log2 fold-change with two-sided moderated-t 95% CI from limma. Because pTau-217 was measured on the same panel, attenuation may reflect biological covariation and shared assay-platform or other correlated measurement variation. |
| B. ATN contrasts adjusted for continuous NULISA pTau-217 | unadjusted | VSNL1 | T+N+ vs T-N- | non-tau | 0.6364948 | 0.045826515 | 0.54653991 | 0.72644968 | 1.9453284e-39 | 4.8244144e-38 | 818 | log2 fold-change with two-sided moderated-t 95% CI from limma |
| B. ATN contrasts adjusted for continuous NULISA pTau-217 | adjusted for NULISA pTau-217 | VSNL1 | T+N+ vs T-N- | non-tau | 0.22477056 | 0.044336574 | 0.13774005 | 0.31180106 | 4.9609043e-07 | 1.5378803e-05 | 818 | Adjusted log2 fold-change with two-sided moderated-t 95% CI from limma. Because pTau-217 was measured on the same panel, attenuation may reflect biological covariation and shared assay-platform or other correlated measurement variation. |

**C. T redefined by tau-PET (extreme tertiles of meta-temporal SUVR)**

| **Panel** | **Analysis** | **Item** | **Stratum / contrast / outcome** | **Class** | **Estimate** | **SE** | **CI_low** | **CI_high** | **P** | **q or FDR** | **n** | **Note** |
| --- | --- | --- | --- | --- | --- | --- | --- | --- | --- | --- | --- | --- |
| C. T redefined by tau-PET (extreme tertiles of meta-temporal SUVR) | tau-PET-defined T contrast | pTau-217 | upper vs lower SUVR tertile | tau-family | 0.828 | 0.25 | 0.339 | 1.317 | 0.00121 | 0.0502 | 128 | secondary q restricted to the eight candidates: 0.00324 |
| C. T redefined by tau-PET (extreme tertiles of meta-temporal SUVR) | tau-PET-defined T contrast | pTau-231 | upper vs lower SUVR tertile | tau-family | 0.772 | 0.231 | 0.319 | 1.225 | 0.00114 | 0.0502 | 128 | secondary q restricted to the eight candidates: 0.00324 |
| C. T redefined by tau-PET (extreme tertiles of meta-temporal SUVR) | tau-PET-defined T contrast | pTau-181 | upper vs lower SUVR tertile | tau-family | 0.805 | 0.221 | 0.373 | 1.238 | 0.000396 | 0.0491 | 128 | secondary q restricted to the eight candidates: 0.00317 |
| C. T redefined by tau-PET (extreme tertiles of meta-temporal SUVR) | tau-PET-defined T contrast | MAPT | upper vs lower SUVR tertile | tau-family | 0.506 | 0.174 | 0.164 | 0.847 | 0.00448 | 0.139 | 128 | secondary q restricted to the eight candidates: 0.00896 |
| C. T redefined by tau-PET (extreme tertiles of meta-temporal SUVR) | tau-PET-defined T contrast | SMOC1 | upper vs lower SUVR tertile | non-tau | 0.237 | 0.172 | -0.1 | 0.574 | 0.17 | 0.681 | 128 | secondary q restricted to the eight candidates: 0.17 |
| C. T redefined by tau-PET (extreme tertiles of meta-temporal SUVR) | tau-PET-defined T contrast | MDH1 | upper vs lower SUVR tertile | non-tau | 0.303 | 0.18 | -0.05 | 0.656 | 0.0954 | 0.62 | 128 | secondary q restricted to the eight candidates: 0.127 |
| C. T redefined by tau-PET (extreme tertiles of meta-temporal SUVR) | tau-PET-defined T contrast | FABP3 | upper vs lower SUVR tertile | non-tau | 0.282 | 0.13 | 0.028 | 0.537 | 0.0319 | 0.44 | 128 | secondary q restricted to the eight candidates: 0.0511 |
| C. T redefined by tau-PET (extreme tertiles of meta-temporal SUVR) | tau-PET-defined T contrast | VSNL1 | upper vs lower SUVR tertile | non-tau | 0.196 | 0.131 | -0.06 | 0.452 | 0.137 | 0.62 | 128 | secondary q restricted to the eight candidates: 0.157 |
| C. T redefined by tau-PET (extreme tertiles of meta-temporal SUVR) | Sample flow | ADNI A+ with QC-passed CSF NULISA = 865; + tau-PET within ±365 d of CSF draw = 192; + extreme tertiles (tau-PET-defined T) = 128; + plate with >=2 participants (plate-adjusted model) = 128 |  |  |  |  |  |  |  |  |  |  |

**D1. Incremental predictive value: strict nested models**

| **Panel** | **Analysis** | **Item** | **Stratum / contrast / outcome** | **Class** | **Estimate** | **SE** | **CI_low** | **CI_high** | **P** | **q or FDR** | **n** | **Note** |
| --- | --- | --- | --- | --- | --- | --- | --- | --- | --- | --- | --- | --- |
| D1. Incremental predictive value: strict nested models | primary: clinical + diagnosis + CSF tau; added block: four non-tau proteins | ADAS-Cog13 anchored to the CSF draw | diagnosis and corresponding cognition within ±90 d of the CSF draw | non-tau | 0.0031660552 | 0.002314649 | -0.0043988801 | 0.010306476 | 0.0065053294 | 0.032526647 | 812 | Mean cross-validated delta R2 from 10x5-fold paired CV; SE is across-repeat SD (split stability, not a confidence interval). The 95% percentile interval used 5,000 participant resamples with repeated out-of-fold predictions fixed; it is descriptive and conditional and excludes model-refitting and feature-selection uncertainty. In-sample delta R2 0.00699; partial F(4,781)=3.5935, raw p=0.006505, Holm p=0.03253. HC3 block-Wald chi-square(4)=14.763894271075801, p=0.0052168219050095404, Holm p=0.026084109525047701. The four-protein block was selected partly using ADNI; the analysis does not independently validate feature selection. |
| D1. Incremental predictive value: strict nested models | primary: clinical + diagnosis + CSF tau; added block: four non-tau proteins | CDR-SB anchored to the CSF draw | diagnosis and corresponding cognition within ±90 d of the CSF draw | non-tau | -0.00021199778 | 0.0012767841 | -0.0041919258 | 0.0036099593 | 0.22348969 | 0.89395875 | 753 | Mean cross-validated delta R2 from 10x5-fold paired CV; SE is across-repeat SD (split stability, not a confidence interval). The 95% percentile interval used 5,000 participant resamples with repeated out-of-fold predictions fixed; it is descriptive and conditional and excludes model-refitting and feature-selection uncertainty. In-sample delta R2 0.00240; partial F(4,722)=1.4263, raw p=0.2235, Holm p=0.8940. HC3 block-Wald chi-square(4)=5.7615999414499104, p=0.21767358379969801, Holm p=0.87069433519879003. The four-protein block was selected partly using ADNI; the analysis does not independently validate feature selection. |
| D1. Incremental predictive value: strict nested models | primary: clinical + diagnosis + CSF tau; added block: four non-tau proteins | MMSE anchored to the CSF draw | diagnosis and corresponding cognition within ±90 d of the CSF draw | non-tau | -0.0027054775 | 0.0014648155 | -0.0069296866 | 0.0013512906 | 0.5105267 | 1 | 756 | Mean cross-validated delta R2 from 10x5-fold paired CV; SE is across-repeat SD (split stability, not a confidence interval). The 95% percentile interval used 5,000 participant resamples with repeated out-of-fold predictions fixed; it is descriptive and conditional and excludes model-refitting and feature-selection uncertainty. In-sample delta R2 0.00162; partial F(4,725)=0.8232, raw p=0.5105, Holm p=1.000. HC3 block-Wald chi-square(4)=2.9871806953624702, p=0.55997296732357105, Holm p=1. The four-protein block was selected partly using ADNI; the analysis does not independently validate feature selection. |
| D1. Incremental predictive value: strict nested models | primary: clinical + diagnosis + CSF tau; added block: four non-tau proteins | CDR-SB annual slope | diagnosis and corresponding cognition within ±90 d of the CSF draw | non-tau | -0.0089374274 | 0.0047171818 | -0.01716518 | -0.00016198824 | 0.59281796 | 1 | 668 | Mean cross-validated delta R2 from 10x5-fold paired CV; SE is across-repeat SD (split stability, not a confidence interval). The 95% percentile interval used 5,000 participant resamples with repeated out-of-fold predictions fixed; it is descriptive and conditional and excludes model-refitting and feature-selection uncertainty. In-sample delta R2 0.00319; partial F(4,636)=0.6990, raw p=0.5928, Holm p=1.000. HC3 block-Wald chi-square(4)=3.39573814488661, p=0.49390759029685599, Holm p=1. The four-protein block was selected partly using ADNI; the analysis does not independently validate feature selection. |
| D1. Incremental predictive value: strict nested models | primary: clinical + diagnosis + CSF tau; added block: four non-tau proteins | MMSE annual slope | diagnosis and corresponding cognition within ±90 d of the CSF draw | non-tau | -0.005957281 | 0.0033278393 | -0.015636188 | 0.0029631228 | 0.46702282 | 1 | 662 | Mean cross-validated delta R2 from 10x5-fold paired CV; SE is across-repeat SD (split stability, not a confidence interval). The 95% percentile interval used 5,000 participant resamples with repeated out-of-fold predictions fixed; it is descriptive and conditional and excludes model-refitting and feature-selection uncertainty. In-sample delta R2 0.00447; partial F(4,630)=0.8941, raw p=0.4670, Holm p=1.000. HC3 block-Wald chi-square(4)=3.54105816810981, p=0.471662884175563, Holm p=1. The four-protein block was selected partly using ADNI; the analysis does not independently validate feature selection. |

**D2. Symmetric four-model performance (exploratory)**

| **Panel** | **Analysis** | **Item** | **Stratum / contrast / outcome** | **Class** | **Estimate** | **SE** | **CI_low** | **CI_high** | **P** | **q or FDR** | **n** | **Note** |
| --- | --- | --- | --- | --- | --- | --- | --- | --- | --- | --- | --- | --- |
| D2. Symmetric four-model performance (exploratory) | mean out-of-fold performance, same participants and folds | Clinical/technical | ADAS-Cog13 | clinical/technical | 0.57151495 | 0.0047525085 |  |  |  |  | 812 | Estimate is mean cross-validated R2; SE column is across-repeat SD (split stability, not a confidence interval). Apparent R2 0.6015; cross-validated RMSE 6.5594 in the original outcome units; cross-validated MAE 0.5199 SD units. Clinical/technical covariates were age, sex, education, APOE e4 carrier status, NULISA plate, and diagnosis; slope models also included the matching baseline cognitive score. |
| D2. Symmetric four-model performance (exploratory) | mean out-of-fold performance, same participants and folds | Tau | ADAS-Cog13 | tau-family | 0.58228728 | 0.0049566575 |  |  |  |  | 812 | Estimate is mean cross-validated R2; SE column is across-repeat SD (split stability, not a confidence interval). Apparent R2 0.6134; cross-validated RMSE 6.4764 in the original outcome units; cross-validated MAE 0.5090 SD units. Clinical/technical covariates were age, sex, education, APOE e4 carrier status, NULISA plate, and diagnosis; slope models also included the matching baseline cognitive score. |
| D2. Symmetric four-model performance (exploratory) | mean out-of-fold performance, same participants and folds | Four-protein | ADAS-Cog13 | non-tau | 0.57444764 | 0.0043060597 |  |  |  |  | 812 | Estimate is mean cross-validated R2; SE column is across-repeat SD (split stability, not a confidence interval). Apparent R2 0.6087; cross-validated RMSE 6.5369 in the original outcome units; cross-validated MAE 0.5173 SD units. Clinical/technical covariates were age, sex, education, APOE e4 carrier status, NULISA plate, and diagnosis; slope models also included the matching baseline cognitive score. |
| D2. Symmetric four-model performance (exploratory) | mean out-of-fold performance, same participants and folds | Full | ADAS-Cog13 | combined | 0.58545333 | 0.0041811961 |  |  |  |  | 812 | Estimate is mean cross-validated R2; SE column is across-repeat SD (split stability, not a confidence interval). Apparent R2 0.6203; cross-validated RMSE 6.4518 in the original outcome units; cross-validated MAE 0.5070 SD units. Clinical/technical covariates were age, sex, education, APOE e4 carrier status, NULISA plate, and diagnosis; slope models also included the matching baseline cognitive score. |
| D2. Symmetric four-model performance (exploratory) | mean out-of-fold performance, same participants and folds | Clinical/technical | CDR-SB | clinical/technical | 0.66458237 | 0.0045303522 |  |  |  |  | 753 | Estimate is mean cross-validated R2; SE column is across-repeat SD (split stability, not a confidence interval). Apparent R2 0.6892; cross-validated RMSE 1.1240 in the original outcome units; cross-validated MAE 0.4099 SD units. Clinical/technical covariates were age, sex, education, APOE e4 carrier status, NULISA plate, and diagnosis; slope models also included the matching baseline cognitive score. |
| D2. Symmetric four-model performance (exploratory) | mean out-of-fold performance, same participants and folds | Tau | CDR-SB | tau-family | 0.66752187 | 0.0043924913 |  |  |  |  | 753 | Estimate is mean cross-validated R2; SE column is across-repeat SD (split stability, not a confidence interval). Apparent R2 0.6939; cross-validated RMSE 1.1190 in the original outcome units; cross-validated MAE 0.4087 SD units. Clinical/technical covariates were age, sex, education, APOE e4 carrier status, NULISA plate, and diagnosis; slope models also included the matching baseline cognitive score. |
| D2. Symmetric four-model performance (exploratory) | mean out-of-fold performance, same participants and folds | Four-protein | CDR-SB | non-tau | 0.66548688 | 0.0047303329 |  |  |  |  | 753 | Estimate is mean cross-validated R2; SE column is across-repeat SD (split stability, not a confidence interval). Apparent R2 0.6927; cross-validated RMSE 1.1224 in the original outcome units; cross-validated MAE 0.4092 SD units. Clinical/technical covariates were age, sex, education, APOE e4 carrier status, NULISA plate, and diagnosis; slope models also included the matching baseline cognitive score. |
| D2. Symmetric four-model performance (exploratory) | mean out-of-fold performance, same participants and folds | Full | CDR-SB | combined | 0.66730987 | 0.0045526725 |  |  |  |  | 753 | Estimate is mean cross-validated R2; SE column is across-repeat SD (split stability, not a confidence interval). Apparent R2 0.6963; cross-validated RMSE 1.1194 in the original outcome units; cross-validated MAE 0.4088 SD units. Clinical/technical covariates were age, sex, education, APOE e4 carrier status, NULISA plate, and diagnosis; slope models also included the matching baseline cognitive score. |
| D2. Symmetric four-model performance (exploratory) | mean out-of-fold performance, same participants and folds | Clinical/technical | MMSE | clinical/technical | 0.60835821 | 0.0041514171 |  |  |  |  | 756 | Estimate is mean cross-validated R2; SE column is across-repeat SD (split stability, not a confidence interval). Apparent R2 0.6366; cross-validated RMSE 1.7674 in the original outcome units; cross-validated MAE 0.5069 SD units. Clinical/technical covariates were age, sex, education, APOE e4 carrier status, NULISA plate, and diagnosis; slope models also included the matching baseline cognitive score. |
| D2. Symmetric four-model performance (exploratory) | mean out-of-fold performance, same participants and folds | Tau | MMSE | tau-family | 0.61122898 | 0.0038757983 |  |  |  |  | 756 | Estimate is mean cross-validated R2; SE column is across-repeat SD (split stability, not a confidence interval). Apparent R2 0.6410; cross-validated RMSE 1.7609 in the original outcome units; cross-validated MAE 0.5042 SD units. Clinical/technical covariates were age, sex, education, APOE e4 carrier status, NULISA plate, and diagnosis; slope models also included the matching baseline cognitive score. |
| D2. Symmetric four-model performance (exploratory) | mean out-of-fold performance, same participants and folds | Four-protein | MMSE | non-tau | 0.60735088 | 0.0048546712 |  |  |  |  | 756 | Estimate is mean cross-validated R2; SE column is across-repeat SD (split stability, not a confidence interval). Apparent R2 0.6395; cross-validated RMSE 1.7697 in the original outcome units; cross-validated MAE 0.5072 SD units. Clinical/technical covariates were age, sex, education, APOE e4 carrier status, NULISA plate, and diagnosis; slope models also included the matching baseline cognitive score. |
| D2. Symmetric four-model performance (exploratory) | mean out-of-fold performance, same participants and folds | Full | MMSE | combined | 0.6085235 | 0.0046162589 |  |  |  |  | 756 | Estimate is mean cross-validated R2; SE column is across-repeat SD (split stability, not a confidence interval). Apparent R2 0.6426; cross-validated RMSE 1.7670 in the original outcome units; cross-validated MAE 0.5059 SD units. Clinical/technical covariates were age, sex, education, APOE e4 carrier status, NULISA plate, and diagnosis; slope models also included the matching baseline cognitive score. |
| D2. Symmetric four-model performance (exploratory) | mean out-of-fold performance, same participants and folds | Clinical/technical | CDR-SB annual slope | clinical/technical | 0.1758704 | 0.011457647 |  |  |  |  | 668 | Estimate is mean cross-validated R2; SE column is across-repeat SD (split stability, not a confidence interval). Apparent R2 0.2502; cross-validated RMSE 1.0852 in the original outcome units; cross-validated MAE 0.6143 SD units. Clinical/technical covariates were age, sex, education, APOE e4 carrier status, NULISA plate, and diagnosis; slope models also included the matching baseline cognitive score. |
| D2. Symmetric four-model performance (exploratory) | mean out-of-fold performance, same participants and folds | Tau | CDR-SB annual slope | tau-family | 0.19325109 | 0.01146711 |  |  |  |  | 668 | Estimate is mean cross-validated R2; SE column is across-repeat SD (split stability, not a confidence interval). Apparent R2 0.2713; cross-validated RMSE 1.0737 in the original outcome units; cross-validated MAE 0.6128 SD units. Clinical/technical covariates were age, sex, education, APOE e4 carrier status, NULISA plate, and diagnosis; slope models also included the matching baseline cognitive score. |
| D2. Symmetric four-model performance (exploratory) | mean out-of-fold performance, same participants and folds | Four-protein | CDR-SB annual slope | non-tau | 0.17400436 | 0.012539679 |  |  |  |  | 668 | Estimate is mean cross-validated R2; SE column is across-repeat SD (split stability, not a confidence interval). Apparent R2 0.2588; cross-validated RMSE 1.0865 in the original outcome units; cross-validated MAE 0.6157 SD units. Clinical/technical covariates were age, sex, education, APOE e4 carrier status, NULISA plate, and diagnosis; slope models also included the matching baseline cognitive score. |
| D2. Symmetric four-model performance (exploratory) | mean out-of-fold performance, same participants and folds | Full | CDR-SB annual slope | combined | 0.18431366 | 0.01313449 |  |  |  |  | 668 | Estimate is mean cross-validated R2; SE column is across-repeat SD (split stability, not a confidence interval). Apparent R2 0.2745; cross-validated RMSE 1.0796 in the original outcome units; cross-validated MAE 0.6173 SD units. Clinical/technical covariates were age, sex, education, APOE e4 carrier status, NULISA plate, and diagnosis; slope models also included the matching baseline cognitive score. |
| D2. Symmetric four-model performance (exploratory) | mean out-of-fold performance, same participants and folds | Clinical/technical | MMSE annual slope | clinical/technical | 0.10042448 | 0.010562341 |  |  |  |  | 662 | Estimate is mean cross-validated R2; SE column is across-repeat SD (split stability, not a confidence interval). Apparent R2 0.1704; cross-validated RMSE 1.8821 in the original outcome units; cross-validated MAE 0.6311 SD units. Clinical/technical covariates were age, sex, education, APOE e4 carrier status, NULISA plate, and diagnosis; slope models also included the matching baseline cognitive score. |
| D2. Symmetric four-model performance (exploratory) | mean out-of-fold performance, same participants and folds | Tau | MMSE annual slope | tau-family | 0.12541529 | 0.010966006 |  |  |  |  | 662 | Estimate is mean cross-validated R2; SE column is across-repeat SD (split stability, not a confidence interval). Apparent R2 0.2039; cross-validated RMSE 1.8529 in the original outcome units; cross-validated MAE 0.6211 SD units. Clinical/technical covariates were age, sex, education, APOE e4 carrier status, NULISA plate, and diagnosis; slope models also included the matching baseline cognitive score. |
| D2. Symmetric four-model performance (exploratory) | mean out-of-fold performance, same participants and folds | Four-protein | MMSE annual slope | non-tau | 0.093737903 | 0.011319313 |  |  |  |  | 662 | Estimate is mean cross-validated R2; SE column is across-repeat SD (split stability, not a confidence interval). Apparent R2 0.1767; cross-validated RMSE 1.8882 in the original outcome units; cross-validated MAE 0.6341 SD units. Clinical/technical covariates were age, sex, education, APOE e4 carrier status, NULISA plate, and diagnosis; slope models also included the matching baseline cognitive score. |
| D2. Symmetric four-model performance (exploratory) | mean out-of-fold performance, same participants and folds | Full | MMSE annual slope | combined | 0.11945801 | 0.0099322158 |  |  |  |  | 662 | Estimate is mean cross-validated R2; SE column is across-repeat SD (split stability, not a confidence interval). Apparent R2 0.2077; cross-validated RMSE 1.8599 in the original outcome units; cross-validated MAE 0.6271 SD units. Clinical/technical covariates were age, sex, education, APOE e4 carrier status, NULISA plate, and diagnosis; slope models also included the matching baseline cognitive score. |

**D3. Four-protein versus tau model (non-nested, exploratory)**

| **Panel** | **Analysis** | **Item** | **Stratum / contrast / outcome** | **Class** | **Estimate** | **SE** | **CI_low** | **CI_high** | **P** | **q or FDR** | **n** | **Note** |
| --- | --- | --- | --- | --- | --- | --- | --- | --- | --- | --- | --- | --- |
| D3. Four-protein versus tau model (non-nested, exploratory) | paired out-of-fold R2: four-protein minus tau | ADAS-Cog13 | same participants and folds | non-nested comparison | -0.0078396387 | 0.0033597294 | -0.017805937 | 0.0020763431 |  |  | 812 | Negative estimates favor the tau model. The percentile interval resampled participants while holding the repeated out-of-fold predictions fixed; it is conditional and descriptive, not a formal equivalence interval. No equivalence or non-inferiority margin was prespecified. Feature selection was not repeated within training folds. |
| D3. Four-protein versus tau model (non-nested, exploratory) | paired out-of-fold R2: four-protein minus tau | CDR-SB | same participants and folds | non-nested comparison | -0.0020349857 | 0.0018056514 | -0.0085047928 | 0.0047453494 |  |  | 753 | Negative estimates favor the tau model. The percentile interval resampled participants while holding the repeated out-of-fold predictions fixed; it is conditional and descriptive, not a formal equivalence interval. No equivalence or non-inferiority margin was prespecified. Feature selection was not repeated within training folds. |
| D3. Four-protein versus tau model (non-nested, exploratory) | paired out-of-fold R2: four-protein minus tau | MMSE | same participants and folds | non-nested comparison | -0.0038780935 | 0.0018902813 | -0.010158186 | 0.0024541321 |  |  | 756 | Negative estimates favor the tau model. The percentile interval resampled participants while holding the repeated out-of-fold predictions fixed; it is conditional and descriptive, not a formal equivalence interval. No equivalence or non-inferiority margin was prespecified. Feature selection was not repeated within training folds. |
| D3. Four-protein versus tau model (non-nested, exploratory) | paired out-of-fold R2: four-protein minus tau | CDR-SB annual slope | same participants and folds | non-nested comparison | -0.019246727 | 0.0066142323 | -0.036764701 | -0.0020870449 |  |  | 668 | Negative estimates favor the tau model. The percentile interval resampled participants while holding the repeated out-of-fold predictions fixed; it is conditional and descriptive, not a formal equivalence interval. No equivalence or non-inferiority margin was prespecified. Feature selection was not repeated within training folds. |
| D3. Four-protein versus tau model (non-nested, exploratory) | paired out-of-fold R2: four-protein minus tau | MMSE annual slope | same participants and folds | non-nested comparison | -0.031677391 | 0.0056324804 | -0.054755052 | -0.010376301 |  |  | 662 | Negative estimates favor the tau model. The percentile interval resampled participants while holding the repeated out-of-fold predictions fixed; it is conditional and descriptive, not a formal equivalence interval. No equivalence or non-inferiority margin was prespecified. Feature selection was not repeated within training folds. |

**D4. Diagnosis-omitted four-model performance (exploratory)**

| **Panel** | **Analysis** | **Item** | **Stratum / contrast / outcome** | **Class** | **Estimate** | **SE** | **CI_low** | **CI_high** | **P** | **q or FDR** | **n** | **Note** |
| --- | --- | --- | --- | --- | --- | --- | --- | --- | --- | --- | --- | --- |
| D4. Diagnosis-omitted four-model performance (exploratory) | mean out-of-fold performance; diagnosis omitted; same participants and folds | Clinical/technical baseline without diagnosis | ADAS-Cog13 | clinical/technical | 0.032532062 | 0.011688737 |  |  |  |  | 812 | Estimate is mean cross-validated R2; SE is across-repeat SD (split stability, not a confidence interval). Apparent R2 0.0946. The baseline retained age, sex, education, APOE e4 carrier status, and NULISA plate but omitted diagnosis; slope models also retained the matching baseline cognitive score. Participants and cross-validation folds were identical to the diagnosis-conditioned analysis. |
| D4. Diagnosis-omitted four-model performance (exploratory) | mean out-of-fold performance; diagnosis omitted; same participants and folds | Tau model without diagnosis | ADAS-Cog13 | tau-family | 0.13966197 | 0.012665675 |  |  |  |  | 812 | Estimate is mean cross-validated R2; SE is across-repeat SD (split stability, not a confidence interval). Apparent R2 0.2005. The baseline retained age, sex, education, APOE e4 carrier status, and NULISA plate but omitted diagnosis; slope models also retained the matching baseline cognitive score. Participants and cross-validation folds were identical to the diagnosis-conditioned analysis. |
| D4. Diagnosis-omitted four-model performance (exploratory) | mean out-of-fold performance; diagnosis omitted; same participants and folds | Four-protein model without diagnosis | ADAS-Cog13 | non-tau | 0.070166407 | 0.012005814 |  |  |  |  | 812 | Estimate is mean cross-validated R2; SE is across-repeat SD (split stability, not a confidence interval). Apparent R2 0.1368. The baseline retained age, sex, education, APOE e4 carrier status, and NULISA plate but omitted diagnosis; slope models also retained the matching baseline cognitive score. Participants and cross-validation folds were identical to the diagnosis-conditioned analysis. |
| D4. Diagnosis-omitted four-model performance (exploratory) | mean out-of-fold performance; diagnosis omitted; same participants and folds | Full model without diagnosis | ADAS-Cog13 | combined | 0.16615972 | 0.01193664 |  |  |  |  | 812 | Estimate is mean cross-validated R2; SE is across-repeat SD (split stability, not a confidence interval). Apparent R2 0.2318. The baseline retained age, sex, education, APOE e4 carrier status, and NULISA plate but omitted diagnosis; slope models also retained the matching baseline cognitive score. Participants and cross-validation folds were identical to the diagnosis-conditioned analysis. |
| D4. Diagnosis-omitted four-model performance (exploratory) | mean out-of-fold performance; diagnosis omitted; same participants and folds | Clinical/technical baseline without diagnosis | CDR-SB | clinical/technical | 0.013610328 | 0.007237538 |  |  |  |  | 753 | Estimate is mean cross-validated R2; SE is across-repeat SD (split stability, not a confidence interval). Apparent R2 0.0719. The baseline retained age, sex, education, APOE e4 carrier status, and NULISA plate but omitted diagnosis; slope models also retained the matching baseline cognitive score. Participants and cross-validation folds were identical to the diagnosis-conditioned analysis. |
| D4. Diagnosis-omitted four-model performance (exploratory) | mean out-of-fold performance; diagnosis omitted; same participants and folds | Tau model without diagnosis | CDR-SB | tau-family | 0.079625273 | 0.0083629475 |  |  |  |  | 753 | Estimate is mean cross-validated R2; SE is across-repeat SD (split stability, not a confidence interval). Apparent R2 0.1404. The baseline retained age, sex, education, APOE e4 carrier status, and NULISA plate but omitted diagnosis; slope models also retained the matching baseline cognitive score. Participants and cross-validation folds were identical to the diagnosis-conditioned analysis. |
| D4. Diagnosis-omitted four-model performance (exploratory) | mean out-of-fold performance; diagnosis omitted; same participants and folds | Four-protein model without diagnosis | CDR-SB | non-tau | 0.047230816 | 0.0052593828 |  |  |  |  | 753 | Estimate is mean cross-validated R2; SE is across-repeat SD (split stability, not a confidence interval). Apparent R2 0.1145. The baseline retained age, sex, education, APOE e4 carrier status, and NULISA plate but omitted diagnosis; slope models also retained the matching baseline cognitive score. Participants and cross-validation folds were identical to the diagnosis-conditioned analysis. |
| D4. Diagnosis-omitted four-model performance (exploratory) | mean out-of-fold performance; diagnosis omitted; same participants and folds | Full model without diagnosis | CDR-SB | combined | 0.092037189 | 0.0087503376 |  |  |  |  | 753 | Estimate is mean cross-validated R2; SE is across-repeat SD (split stability, not a confidence interval). Apparent R2 0.1626. The baseline retained age, sex, education, APOE e4 carrier status, and NULISA plate but omitted diagnosis; slope models also retained the matching baseline cognitive score. Participants and cross-validation folds were identical to the diagnosis-conditioned analysis. |
| D4. Diagnosis-omitted four-model performance (exploratory) | mean out-of-fold performance; diagnosis omitted; same participants and folds | Clinical/technical baseline without diagnosis | MMSE | clinical/technical | 0.040167405 | 0.009087016 |  |  |  |  | 756 | Estimate is mean cross-validated R2; SE is across-repeat SD (split stability, not a confidence interval). Apparent R2 0.1056. The baseline retained age, sex, education, APOE e4 carrier status, and NULISA plate but omitted diagnosis; slope models also retained the matching baseline cognitive score. Participants and cross-validation folds were identical to the diagnosis-conditioned analysis. |
| D4. Diagnosis-omitted four-model performance (exploratory) | mean out-of-fold performance; diagnosis omitted; same participants and folds | Tau model without diagnosis | MMSE | tau-family | 0.10904741 | 0.008142502 |  |  |  |  | 756 | Estimate is mean cross-validated R2; SE is across-repeat SD (split stability, not a confidence interval). Apparent R2 0.1740. The baseline retained age, sex, education, APOE e4 carrier status, and NULISA plate but omitted diagnosis; slope models also retained the matching baseline cognitive score. Participants and cross-validation folds were identical to the diagnosis-conditioned analysis. |
| D4. Diagnosis-omitted four-model performance (exploratory) | mean out-of-fold performance; diagnosis omitted; same participants and folds | Four-protein model without diagnosis | MMSE | non-tau | 0.067372755 | 0.0089862115 |  |  |  |  | 756 | Estimate is mean cross-validated R2; SE is across-repeat SD (split stability, not a confidence interval). Apparent R2 0.1403. The baseline retained age, sex, education, APOE e4 carrier status, and NULISA plate but omitted diagnosis; slope models also retained the matching baseline cognitive score. Participants and cross-validation folds were identical to the diagnosis-conditioned analysis. |
| D4. Diagnosis-omitted four-model performance (exploratory) | mean out-of-fold performance; diagnosis omitted; same participants and folds | Full model without diagnosis | MMSE | combined | 0.11910489 | 0.0078855863 |  |  |  |  | 756 | Estimate is mean cross-validated R2; SE is across-repeat SD (split stability, not a confidence interval). Apparent R2 0.1942. The baseline retained age, sex, education, APOE e4 carrier status, and NULISA plate but omitted diagnosis; slope models also retained the matching baseline cognitive score. Participants and cross-validation folds were identical to the diagnosis-conditioned analysis. |
| D4. Diagnosis-omitted four-model performance (exploratory) | mean out-of-fold performance; diagnosis omitted; same participants and folds | Clinical/technical baseline without diagnosis | CDR-SB annual slope | clinical/technical | 0.1631168 | 0.012046454 |  |  |  |  | 668 | Estimate is mean cross-validated R2; SE is across-repeat SD (split stability, not a confidence interval). Apparent R2 0.2338. The baseline retained age, sex, education, APOE e4 carrier status, and NULISA plate but omitted diagnosis; slope models also retained the matching baseline cognitive score. Participants and cross-validation folds were identical to the diagnosis-conditioned analysis. |
| D4. Diagnosis-omitted four-model performance (exploratory) | mean out-of-fold performance; diagnosis omitted; same participants and folds | Tau model without diagnosis | CDR-SB annual slope | tau-family | 0.18583501 | 0.01296284 |  |  |  |  | 668 | Estimate is mean cross-validated R2; SE is across-repeat SD (split stability, not a confidence interval). Apparent R2 0.2601. The baseline retained age, sex, education, APOE e4 carrier status, and NULISA plate but omitted diagnosis; slope models also retained the matching baseline cognitive score. Participants and cross-validation folds were identical to the diagnosis-conditioned analysis. |
| D4. Diagnosis-omitted four-model performance (exploratory) | mean out-of-fold performance; diagnosis omitted; same participants and folds | Four-protein model without diagnosis | CDR-SB annual slope | non-tau | 0.16186692 | 0.014671169 |  |  |  |  | 668 | Estimate is mean cross-validated R2; SE is across-repeat SD (split stability, not a confidence interval). Apparent R2 0.2437. The baseline retained age, sex, education, APOE e4 carrier status, and NULISA plate but omitted diagnosis; slope models also retained the matching baseline cognitive score. Participants and cross-validation folds were identical to the diagnosis-conditioned analysis. |
| D4. Diagnosis-omitted four-model performance (exploratory) | mean out-of-fold performance; diagnosis omitted; same participants and folds | Full model without diagnosis | CDR-SB annual slope | combined | 0.17725979 | 0.015249313 |  |  |  |  | 668 | Estimate is mean cross-validated R2; SE is across-repeat SD (split stability, not a confidence interval). Apparent R2 0.2642. The baseline retained age, sex, education, APOE e4 carrier status, and NULISA plate but omitted diagnosis; slope models also retained the matching baseline cognitive score. Participants and cross-validation folds were identical to the diagnosis-conditioned analysis. |
| D4. Diagnosis-omitted four-model performance (exploratory) | mean out-of-fold performance; diagnosis omitted; same participants and folds | Clinical/technical baseline without diagnosis | MMSE annual slope | clinical/technical | 0.062139714 | 0.010114994 |  |  |  |  | 662 | Estimate is mean cross-validated R2; SE is across-repeat SD (split stability, not a confidence interval). Apparent R2 0.1297. The baseline retained age, sex, education, APOE e4 carrier status, and NULISA plate but omitted diagnosis; slope models also retained the matching baseline cognitive score. Participants and cross-validation folds were identical to the diagnosis-conditioned analysis. |
| D4. Diagnosis-omitted four-model performance (exploratory) | mean out-of-fold performance; diagnosis omitted; same participants and folds | Tau model without diagnosis | MMSE annual slope | tau-family | 0.097315 | 0.010397837 |  |  |  |  | 662 | Estimate is mean cross-validated R2; SE is across-repeat SD (split stability, not a confidence interval). Apparent R2 0.1727. The baseline retained age, sex, education, APOE e4 carrier status, and NULISA plate but omitted diagnosis; slope models also retained the matching baseline cognitive score. Participants and cross-validation folds were identical to the diagnosis-conditioned analysis. |
| D4. Diagnosis-omitted four-model performance (exploratory) | mean out-of-fold performance; diagnosis omitted; same participants and folds | Four-protein model without diagnosis | MMSE annual slope | non-tau | 0.05694131 | 0.012026556 |  |  |  |  | 662 | Estimate is mean cross-validated R2; SE is across-repeat SD (split stability, not a confidence interval). Apparent R2 0.1381. The baseline retained age, sex, education, APOE e4 carrier status, and NULISA plate but omitted diagnosis; slope models also retained the matching baseline cognitive score. Participants and cross-validation folds were identical to the diagnosis-conditioned analysis. |
| D4. Diagnosis-omitted four-model performance (exploratory) | mean out-of-fold performance; diagnosis omitted; same participants and folds | Full model without diagnosis | MMSE annual slope | combined | 0.093129712 | 0.010244004 |  |  |  |  | 662 | Estimate is mean cross-validated R2; SE is across-repeat SD (split stability, not a confidence interval). Apparent R2 0.1782. The baseline retained age, sex, education, APOE e4 carrier status, and NULISA plate but omitted diagnosis; slope models also retained the matching baseline cognitive score. Participants and cross-validation folds were identical to the diagnosis-conditioned analysis. |

**D5. Diagnosis-omitted incremental value after tau (exploratory)**

| **Panel** | **Analysis** | **Item** | **Stratum / contrast / outcome** | **Class** | **Estimate** | **SE** | **CI_low** | **CI_high** | **P** | **q or FDR** | **n** | **Note** |
| --- | --- | --- | --- | --- | --- | --- | --- | --- | --- | --- | --- | --- |
| D5. Diagnosis-omitted incremental value after tau (exploratory) | paired out-of-fold R2: full minus tau; diagnosis omitted | ADAS-Cog13 | same participants and folds | nested comparison | 0.026497752 |  | 0.0039421859 | 0.049826889 | 2.7130022e-06 |  | 812 | In-sample delta R2 0.03125; partial F(4,783)=7.9620, raw p=0.000002713. The 95% percentile interval used 5,000 participant resamples while holding repeated out-of-fold predictions fixed; it is descriptive and conditional, and does not include model-refitting or feature-selection uncertainty. No equivalence or non-inferiority margin was prespecified. |
| D5. Diagnosis-omitted incremental value after tau (exploratory) | paired out-of-fold R2: full minus tau; diagnosis omitted | CDR-SB | same participants and folds | nested comparison | 0.012411916 |  | -0.0084124015 | 0.033201451 | 0.00077354739 |  | 753 | In-sample delta R2 0.02228; partial F(4,724)=4.8152, raw p=0.0007735. The 95% percentile interval used 5,000 participant resamples while holding repeated out-of-fold predictions fixed; it is descriptive and conditional, and does not include model-refitting or feature-selection uncertainty. No equivalence or non-inferiority margin was prespecified. |
| D5. Diagnosis-omitted incremental value after tau (exploratory) | paired out-of-fold R2: full minus tau; diagnosis omitted | MMSE | same participants and folds | nested comparison | 0.01005748 |  | -0.01071517 | 0.030878559 | 0.0012285048 |  | 756 | In-sample delta R2 0.02018; partial F(4,727)=4.5524, raw p=0.001229. The 95% percentile interval used 5,000 participant resamples while holding repeated out-of-fold predictions fixed; it is descriptive and conditional, and does not include model-refitting or feature-selection uncertainty. No equivalence or non-inferiority margin was prespecified. |
| D5. Diagnosis-omitted incremental value after tau (exploratory) | paired out-of-fold R2: full minus tau; diagnosis omitted | CDR-SB annual slope | same participants and folds | nested comparison | -0.0085752177 |  | -0.017788956 | 0.0008801172 | 0.46819302 |  | 668 | In-sample delta R2 0.00412; partial F(4,638)=0.8921, raw p=0.4682. The 95% percentile interval used 5,000 participant resamples while holding repeated out-of-fold predictions fixed; it is descriptive and conditional, and does not include model-refitting or feature-selection uncertainty. No equivalence or non-inferiority margin was prespecified. |
| D5. Diagnosis-omitted incremental value after tau (exploratory) | paired out-of-fold R2: full minus tau; diagnosis omitted | MMSE annual slope | same participants and folds | nested comparison | -0.0041852879 |  | -0.015654767 | 0.0064972542 | 0.29789733 |  | 662 | In-sample delta R2 0.00552; partial F(4,632)=1.0617, raw p=0.3746. The 95% percentile interval used 5,000 participant resamples while holding repeated out-of-fold predictions fixed; it is descriptive and conditional, and does not include model-refitting or feature-selection uncertainty. No equivalence or non-inferiority margin was prespecified. |

**D6. Nested-model in-sample increments (full analysis sample, no cross-validation)**

| **Panel** | **Analysis** | **Item** | **Stratum / contrast / outcome** | **Class** | **Estimate** | **SE** | **CI_low** | **CI_high** | **P** | **q or FDR** | **n** | **Note** |
| --- | --- | --- | --- | --- | --- | --- | --- | --- | --- | --- | --- | --- |
| D6. Nested-model in-sample increments (full analysis sample, no cross-validation) | primary diagnosis-conditioned nested model: full versus tau | Four-protein block added to tau model | ADAS-Cog13 | in-sample | 0.0069875566 |  |  |  | 0.0065053294 | 0.032526647 | 812 | In-sample increment from the same primary diagnosis-conditioned nested models as Panel D1 (full versus tau), computed on the full analysis sample without cross-validation; reported for completeness. |
| D6. Nested-model in-sample increments (full analysis sample, no cross-validation) | primary diagnosis-conditioned nested model: full versus tau | Four-protein block added to tau model | CDR-SB | in-sample | 0.0023999611 |  |  |  | 0.22348969 | 0.89395875 | 753 | In-sample increment from the same primary diagnosis-conditioned nested models as Panel D1 (full versus tau), computed on the full analysis sample without cross-validation; reported for completeness. |
| D6. Nested-model in-sample increments (full analysis sample, no cross-validation) | primary diagnosis-conditioned nested model: full versus tau | Four-protein block added to tau model | MMSE | in-sample | 0.0016232166 |  |  |  | 0.5105267 | 1 | 756 | In-sample increment from the same primary diagnosis-conditioned nested models as Panel D1 (full versus tau), computed on the full analysis sample without cross-validation; reported for completeness. |
| D6. Nested-model in-sample increments (full analysis sample, no cross-validation) | primary diagnosis-conditioned nested model: full versus tau | Four-protein block added to tau model | CDR-SB annual slope | in-sample | 0.0031896185 |  |  |  | 0.59281796 | 1 | 668 | In-sample increment from the same primary diagnosis-conditioned nested models as Panel D1 (full versus tau), computed on the full analysis sample without cross-validation; reported for completeness. |
| D6. Nested-model in-sample increments (full analysis sample, no cross-validation) | primary diagnosis-conditioned nested model: full versus tau | Four-protein block added to tau model | MMSE annual slope | in-sample | 0.004466754 |  |  |  | 0.46702282 | 1 | 662 | In-sample increment from the same primary diagnosis-conditioned nested models as Panel D1 (full versus tau), computed on the full analysis sample without cross-validation; reported for completeness. |

**E1. Protein-cognition correlations, pooled versus within diagnosis**

| **Panel** | **Analysis** | **Item** | **Stratum / contrast / outcome** | **Class** | **Estimate** | **SE** | **CI_low** | **CI_high** | **P** | **q or FDR** | **n** | **Note** |
| --- | --- | --- | --- | --- | --- | --- | --- | --- | --- | --- | --- | --- |
| E1. Protein-cognition correlations, pooled versus within diagnosis | pooled across anchored diagnoses | pTau-181 | MMSE | tau-family | -0.3140807 |  | -0.37594986 | -0.24942372 | 2.430388e-19 |  | 781 | Diagnosis and cognition were anchored to the CSF draw; cognition was required within ±90 days. |
| E1. Protein-cognition correlations, pooled versus within diagnosis | within diagnosis, Fisher-z weighted by n-3 | pTau-181 | MMSE | tau-family | -0.112855 |  | -0.18183372 | -0.042771056 | 0.0016378622 |  | 781 | CN rho -0.035 (-0.183 to 0.114, n=175); MCI rho -0.191 (-0.284 to -0.094, n=400); AD rho -0.023 (-0.159 to 0.114, n=206); partial rho adjusted for diagnosis -0.133 (-0.202 to -0.064, p=0.000188). |
| E1. Protein-cognition correlations, pooled versus within diagnosis | pooled across anchored diagnoses | pTau-181 | ADAS-Cog13 | tau-family | 0.35162282 |  | 0.29086189 | 0.40955889 | 8.0114386e-26 |  | 839 | Diagnosis and cognition were anchored to the CSF draw; cognition was required within ±90 days. |
| E1. Protein-cognition correlations, pooled versus within diagnosis | within diagnosis, Fisher-z weighted by n-3 | pTau-181 | ADAS-Cog13 | tau-family | 0.17455282 |  | 0.10790565 | 0.23963807 | 3.7575696e-07 |  | 839 | CN rho 0.150 (0.012 to 0.283, n=201); MCI rho 0.218 (0.125 to 0.307, n=421); AD rho 0.111 (-0.023 to 0.241, n=217); partial rho adjusted for diagnosis 0.174 (0.108 to 0.239, p=4.08e-7). |
| E1. Protein-cognition correlations, pooled versus within diagnosis | pooled across anchored diagnoses | pTau-181 | CDR-SB | tau-family | 0.34210332 |  | 0.27851246 | 0.40270781 | 8.8050668e-23 |  | 778 | Diagnosis and cognition were anchored to the CSF draw; cognition was required within ±90 days. |
| E1. Protein-cognition correlations, pooled versus within diagnosis | within diagnosis, Fisher-z weighted by n-3 | pTau-181 | CDR-SB | tau-family | 0.11047047 |  | 0.040223346 | 0.17963092 | 0.0020980229 |  | 778 | CN rho 0.078 (-0.072 to 0.224, n=174); MCI rho 0.168 (0.071 to 0.262, n=399); AD rho 0.023 (-0.114 to 0.160, n=205); partial rho adjusted for diagnosis 0.136 (0.066 to 0.204, p=0.000145). |
| E1. Protein-cognition correlations, pooled versus within diagnosis | pooled across anchored diagnoses | pTau-217 | MMSE | tau-family | -0.29479559 |  | -0.35755382 | -0.22938678 | 3.9987476e-17 |  | 781 | Diagnosis and cognition were anchored to the CSF draw; cognition was required within ±90 days. |
| E1. Protein-cognition correlations, pooled versus within diagnosis | within diagnosis, Fisher-z weighted by n-3 | pTau-217 | MMSE | tau-family | -0.11540617 |  | -0.18433192 | -0.045350877 | 0.0012778964 |  | 781 | CN rho -0.035 (-0.182 to 0.114, n=175); MCI rho -0.186 (-0.279 to -0.090, n=400); AD rho -0.043 (-0.178 to 0.095, n=206); partial rho adjusted for diagnosis -0.131 (-0.199 to -0.061, p=0.000248). |
| E1. Protein-cognition correlations, pooled versus within diagnosis | pooled across anchored diagnoses | pTau-217 | ADAS-Cog13 | tau-family | 0.32125435 |  | 0.25920729 | 0.38066059 | 1.3534313e-21 |  | 839 | Diagnosis and cognition were anchored to the CSF draw; cognition was required within ±90 days. |
| E1. Protein-cognition correlations, pooled versus within diagnosis | within diagnosis, Fisher-z weighted by n-3 | pTau-217 | ADAS-Cog13 | tau-family | 0.15875386 |  | 0.091817404 | 0.22426209 | 3.9753099e-06 |  | 839 | CN rho 0.157 (0.019 to 0.289, n=201); MCI rho 0.186 (0.092 to 0.276, n=421); AD rho 0.107 (-0.026 to 0.237, n=217); partial rho adjusted for diagnosis 0.153 (0.086 to 0.219, p=0.00000865). |
| E1. Protein-cognition correlations, pooled versus within diagnosis | pooled across anchored diagnoses | pTau-217 | CDR-SB | tau-family | 0.31638468 |  | 0.25169396 | 0.3782608 | 1.5110521e-19 |  | 778 | Diagnosis and cognition were anchored to the CSF draw; cognition was required within ±90 days. |
| E1. Protein-cognition correlations, pooled versus within diagnosis | within diagnosis, Fisher-z weighted by n-3 | pTau-217 | CDR-SB | tau-family | 0.097957913 |  | 0.027587945 | 0.16736177 | 0.0064263461 |  | 778 | CN rho 0.070 (-0.079 to 0.217, n=174); MCI rho 0.156 (0.058 to 0.250, n=399); AD rho 0.007 (-0.131 to 0.144, n=205); partial rho adjusted for diagnosis 0.122 (0.052 to 0.191, p=0.000661). |
| E1. Protein-cognition correlations, pooled versus within diagnosis | pooled across anchored diagnoses | pTau-231 | MMSE | tau-family | -0.29013649 |  | -0.35310218 | -0.22455434 | 1.2964966e-16 |  | 781 | Diagnosis and cognition were anchored to the CSF draw; cognition was required within ±90 days. |
| E1. Protein-cognition correlations, pooled versus within diagnosis | within diagnosis, Fisher-z weighted by n-3 | pTau-231 | MMSE | tau-family | -0.10971873 |  | -0.17876135 | -0.039600837 | 0.0022071366 |  | 781 | CN rho -0.035 (-0.182 to 0.114, n=175); MCI rho -0.182 (-0.275 to -0.086, n=400); AD rho -0.029 (-0.165 to 0.108, n=206); partial rho adjusted for diagnosis -0.128 (-0.196 to -0.058, p=0.000342). |
| E1. Protein-cognition correlations, pooled versus within diagnosis | pooled across anchored diagnoses | pTau-231 | ADAS-Cog13 | tau-family | 0.31889963 |  | 0.25675841 | 0.37841501 | 2.7529795e-21 |  | 839 | Diagnosis and cognition were anchored to the CSF draw; cognition was required within ±90 days. |
| E1. Protein-cognition correlations, pooled versus within diagnosis | within diagnosis, Fisher-z weighted by n-3 | pTau-231 | ADAS-Cog13 | tau-family | 0.15945712 |  | 0.092532793 | 0.22494721 | 3.5961152e-06 |  | 839 | CN rho 0.147 (0.009 to 0.280, n=201); MCI rho 0.191 (0.097 to 0.281, n=421); AD rho 0.109 (-0.024 to 0.239, n=217); partial rho adjusted for diagnosis 0.155 (0.088 to 0.220, p=0.00000660). |
| E1. Protein-cognition correlations, pooled versus within diagnosis | pooled across anchored diagnoses | pTau-231 | CDR-SB | tau-family | 0.31156775 |  | 0.24668206 | 0.37367243 | 5.6392569e-19 |  | 778 | Diagnosis and cognition were anchored to the CSF draw; cognition was required within ±90 days. |
| E1. Protein-cognition correlations, pooled versus within diagnosis | within diagnosis, Fisher-z weighted by n-3 | pTau-231 | CDR-SB | tau-family | 0.096449699 |  | 0.02606644 | 0.16588144 | 0.0072971035 |  | 778 | CN rho 0.063 (-0.086 to 0.210, n=174); MCI rho 0.154 (0.057 to 0.248, n=399); AD rho 0.011 (-0.127 to 0.147, n=205); partial rho adjusted for diagnosis 0.120 (0.050 to 0.189, p=0.000785). |
| E1. Protein-cognition correlations, pooled versus within diagnosis | pooled across anchored diagnoses | MAPT | MMSE | tau-family | -0.27725556 |  | -0.34078 | -0.21121097 | 2.9955727e-15 |  | 781 | Diagnosis and cognition were anchored to the CSF draw; cognition was required within ±90 days. |
| E1. Protein-cognition correlations, pooled versus within diagnosis | within diagnosis, Fisher-z weighted by n-3 | MAPT | MMSE | tau-family | -0.11570444 |  | -0.18462394 | -0.045652556 | 0.0012409475 |  | 781 | CN rho -0.049 (-0.196 to 0.100, n=175); MCI rho -0.159 (-0.253 to -0.062, n=400); AD rho -0.086 (-0.220 to 0.051, n=206); partial rho adjusted for diagnosis -0.124 (-0.192 to -0.054, p=0.000530). |
| E1. Protein-cognition correlations, pooled versus within diagnosis | pooled across anchored diagnoses | MAPT | ADAS-Cog13 | tau-family | 0.29977323 |  | 0.23689663 | 0.36014908 | 6.9911314e-19 |  | 839 | Diagnosis and cognition were anchored to the CSF draw; cognition was required within ±90 days. |
| E1. Protein-cognition correlations, pooled versus within diagnosis | within diagnosis, Fisher-z weighted by n-3 | MAPT | ADAS-Cog13 | tau-family | 0.1465476 |  | 0.079411521 | 0.21236025 | 2.1128851e-05 |  | 839 | CN rho 0.091 (-0.048 to 0.227, n=201); MCI rho 0.166 (0.072 to 0.258, n=421); AD rho 0.159 (0.026 to 0.286, n=217); partial rho adjusted for diagnosis 0.145 (0.078 to 0.211, p=0.0000260). |
| E1. Protein-cognition correlations, pooled versus within diagnosis | pooled across anchored diagnoses | MAPT | CDR-SB | tau-family | 0.30422478 |  | 0.23904854 | 0.366672 | 4.0093058e-18 |  | 778 | Diagnosis and cognition were anchored to the CSF draw; cognition was required within ±90 days. |
| E1. Protein-cognition correlations, pooled versus within diagnosis | within diagnosis, Fisher-z weighted by n-3 | MAPT | CDR-SB | tau-family | 0.10964766 |  | 0.039391768 | 0.17882478 | 0.0022664393 |  | 778 | CN rho 0.050 (-0.099 to 0.198, n=174); MCI rho 0.159 (0.062 to 0.253, n=399); AD rho 0.062 (-0.075 to 0.198, n=205); partial rho adjusted for diagnosis 0.132 (0.062 to 0.201, p=0.000221). |
| E1. Protein-cognition correlations, pooled versus within diagnosis | pooled across anchored diagnoses | SMOC1 | MMSE | non-tau | -0.15322535 |  | -0.22100245 | -0.083975327 | 1.7029374e-05 |  | 781 | Diagnosis and cognition were anchored to the CSF draw; cognition was required within ±90 days. |
| E1. Protein-cognition correlations, pooled versus within diagnosis | within diagnosis, Fisher-z weighted by n-3 | SMOC1 | MMSE | non-tau | -0.071357125 |  | -0.14107208 | -0.0009379618 | 0.047030531 |  | 781 | CN rho 0.003 (-0.146 to 0.151, n=175); MCI rho -0.106 (-0.202 to -0.008, n=400); AD rho -0.067 (-0.202 to 0.071, n=206); partial rho adjusted for diagnosis -0.080 (-0.149 to -0.010, p=0.0259). |
| E1. Protein-cognition correlations, pooled versus within diagnosis | pooled across anchored diagnoses | SMOC1 | ADAS-Cog13 | non-tau | 0.1594177 |  | 0.092735169 | 0.2246766 | 3.4767472e-06 |  | 839 | Diagnosis and cognition were anchored to the CSF draw; cognition was required within ±90 days. |
| E1. Protein-cognition correlations, pooled versus within diagnosis | within diagnosis, Fisher-z weighted by n-3 | SMOC1 | ADAS-Cog13 | non-tau | 0.08665017 |  | 0.018834445 | 0.15367226 | 0.012326972 |  | 839 | CN rho 0.115 (-0.024 to 0.249, n=201); MCI rho 0.068 (-0.027 to 0.163, n=421); AD rho 0.096 (-0.037 to 0.227, n=217); partial rho adjusted for diagnosis 0.079 (0.012 to 0.146, p=0.0218). |
| E1. Protein-cognition correlations, pooled versus within diagnosis | pooled across anchored diagnoses | SMOC1 | CDR-SB | non-tau | 0.17604411 |  | 0.10708122 | 0.2433212 | 7.7690694e-07 |  | 778 | Diagnosis and cognition were anchored to the CSF draw; cognition was required within ±90 days. |
| E1. Protein-cognition correlations, pooled versus within diagnosis | within diagnosis, Fisher-z weighted by n-3 | SMOC1 | CDR-SB | non-tau | 0.069340128 |  | -0.0012265283 | 0.13921963 | 0.054110455 |  | 778 | CN rho -0.049 (-0.196 to 0.101, n=174); MCI rho 0.088 (-0.010 to 0.185, n=399); AD rho 0.131 (-0.006 to 0.263, n=205); partial rho adjusted for diagnosis 0.093 (0.023 to 0.162, p=0.00960). |
| E1. Protein-cognition correlations, pooled versus within diagnosis | pooled across anchored diagnoses | MDH1 | MMSE | non-tau | -0.14203755 |  | -0.21009677 | -0.072608351 | 6.7967984e-05 |  | 781 | Diagnosis and cognition were anchored to the CSF draw; cognition was required within ±90 days. |
| E1. Protein-cognition correlations, pooled versus within diagnosis | within diagnosis, Fisher-z weighted by n-3 | MDH1 | MMSE | non-tau | -0.035853253 |  | -0.10600946 | 0.034658133 | 0.3189559 |  | 781 | CN rho -0.022 (-0.170 to 0.126, n=175); MCI rho -0.051 (-0.148 to 0.048, n=400); AD rho -0.018 (-0.154 to 0.119, n=206); partial rho adjusted for diagnosis -0.044 (-0.114 to 0.026, p=0.215). |
| E1. Protein-cognition correlations, pooled versus within diagnosis | pooled across anchored diagnoses | MDH1 | ADAS-Cog13 | non-tau | 0.17057074 |  | 0.10408929 | 0.23553468 | 6.7034639e-07 |  | 839 | Diagnosis and cognition were anchored to the CSF draw; cognition was required within ±90 days. |
| E1. Protein-cognition correlations, pooled versus within diagnosis | within diagnosis, Fisher-z weighted by n-3 | MDH1 | ADAS-Cog13 | non-tau | 0.087803984 |  | 0.019996667 | 0.15480726 | 0.011208218 |  | 839 | CN rho 0.060 (-0.079 to 0.197, n=201); MCI rho 0.053 (-0.043 to 0.148, n=421); AD rho 0.181 (0.049 to 0.307, n=217); partial rho adjusted for diagnosis 0.080 (0.012 to 0.147, p=0.0211). |
| E1. Protein-cognition correlations, pooled versus within diagnosis | pooled across anchored diagnoses | MDH1 | CDR-SB | non-tau | 0.1605854 |  | 0.091328355 | 0.22829646 | 6.7572778e-06 |  | 778 | Diagnosis and cognition were anchored to the CSF draw; cognition was required within ±90 days. |
| E1. Protein-cognition correlations, pooled versus within diagnosis | within diagnosis, Fisher-z weighted by n-3 | MDH1 | CDR-SB | non-tau | 0.031126192 |  | -0.039521263 | 0.101464 | 0.38789853 |  | 778 | CN rho -0.038 (-0.186 to 0.111, n=174); MCI rho 0.016 (-0.082 to 0.114, n=399); AD rho 0.119 (-0.019 to 0.252, n=205); partial rho adjusted for diagnosis 0.037 (-0.033 to 0.108, p=0.297). |
| E1. Protein-cognition correlations, pooled versus within diagnosis | pooled across anchored diagnoses | FABP3 | MMSE | non-tau | -0.21525821 |  | -0.28116505 | -0.14733035 | 1.2211895e-09 |  | 781 | Diagnosis and cognition were anchored to the CSF draw; cognition was required within ±90 days. |
| E1. Protein-cognition correlations, pooled versus within diagnosis | within diagnosis, Fisher-z weighted by n-3 | FABP3 | MMSE | non-tau | -0.085257777 |  | -0.15475249 | -0.014923503 | 0.017565948 |  | 781 | CN rho -0.086 (-0.231 to 0.063, n=175); MCI rho -0.099 (-0.196 to -0.001, n=400); AD rho -0.057 (-0.192 to 0.081, n=206); partial rho adjusted for diagnosis -0.092 (-0.161 to -0.022, p=0.0101). |
| E1. Protein-cognition correlations, pooled versus within diagnosis | pooled across anchored diagnoses | FABP3 | ADAS-Cog13 | non-tau | 0.25425055 |  | 0.18983417 | 0.31648741 | 7.6202573e-14 |  | 839 | Diagnosis and cognition were anchored to the CSF draw; cognition was required within ±90 days. |
| E1. Protein-cognition correlations, pooled versus within diagnosis | within diagnosis, Fisher-z weighted by n-3 | FABP3 | ADAS-Cog13 | non-tau | 0.14003366 |  | 0.072799553 | 0.20600076 | 4.8859681e-05 |  | 839 | CN rho 0.130 (-0.009 to 0.263, n=201); MCI rho 0.149 (0.054 to 0.241, n=421); AD rho 0.132 (-0.001 to 0.261, n=217); partial rho adjusted for diagnosis 0.147 (0.080 to 0.213, p=0.0000200). |
| E1. Protein-cognition correlations, pooled versus within diagnosis | pooled across anchored diagnoses | FABP3 | CDR-SB | non-tau | 0.24112491 |  | 0.17378231 | 0.30622289 | 9.3863714e-12 |  | 778 | Diagnosis and cognition were anchored to the CSF draw; cognition was required within ±90 days. |
| E1. Protein-cognition correlations, pooled versus within diagnosis | within diagnosis, Fisher-z weighted by n-3 | FABP3 | CDR-SB | non-tau | 0.092549129 |  | 0.022133009 | 0.16205153 | 0.010057928 |  | 778 | CN rho 0.026 (-0.123 to 0.174, n=174); MCI rho 0.109 (0.011 to 0.205, n=399); AD rho 0.116 (-0.021 to 0.249, n=205); partial rho adjusted for diagnosis 0.101 (0.030 to 0.170, p=0.00505). |
| E1. Protein-cognition correlations, pooled versus within diagnosis | pooled across anchored diagnoses | VSNL1 | MMSE | non-tau | -0.12818152 |  | -0.19656663 | -0.058555375 | 0.00032903758 |  | 781 | Diagnosis and cognition were anchored to the CSF draw; cognition was required within ±90 days. |
| E1. Protein-cognition correlations, pooled versus within diagnosis | within diagnosis, Fisher-z weighted by n-3 | VSNL1 | MMSE | non-tau | -0.050265222 |  | -0.12026338 | 0.020230267 | 0.16217566 |  | 781 | CN rho -0.046 (-0.193 to 0.103, n=175); MCI rho -0.048 (-0.145 to 0.051, n=400); AD rho -0.059 (-0.194 to 0.078, n=206); partial rho adjusted for diagnosis -0.056 (-0.126 to 0.014, p=0.116). |
| E1. Protein-cognition correlations, pooled versus within diagnosis | pooled across anchored diagnoses | VSNL1 | ADAS-Cog13 | non-tau | 0.14297146 |  | 0.076023991 | 0.20863568 | 3.2325118e-05 |  | 839 | Diagnosis and cognition were anchored to the CSF draw; cognition was required within ±90 days. |
| E1. Protein-cognition correlations, pooled versus within diagnosis | within diagnosis, Fisher-z weighted by n-3 | VSNL1 | ADAS-Cog13 | non-tau | 0.060735771 |  | -0.0072205992 | 0.12813373 | 0.079784655 |  | 839 | CN rho 0.021 (-0.118 to 0.158, n=201); MCI rho 0.052 (-0.043 to 0.147, n=421); AD rho 0.114 (-0.019 to 0.243, n=217); partial rho adjusted for diagnosis 0.056 (-0.012 to 0.123, p=0.105). |
| E1. Protein-cognition correlations, pooled versus within diagnosis | pooled across anchored diagnoses | VSNL1 | CDR-SB | non-tau | 0.14878406 |  | 0.079325733 | 0.21680467 | 3.0928394e-05 |  | 778 | Diagnosis and cognition were anchored to the CSF draw; cognition was required within ±90 days. |
| E1. Protein-cognition correlations, pooled versus within diagnosis | within diagnosis, Fisher-z weighted by n-3 | VSNL1 | CDR-SB | non-tau | 0.059763268 |  | -0.010843112 | 0.12977666 | 0.09706271 |  | 778 | CN rho 0.020 (-0.129 to 0.168, n=174); MCI rho 0.053 (-0.045 to 0.150, n=399); AD rho 0.106 (-0.031 to 0.240, n=205); partial rho adjusted for diagnosis 0.061 (-0.009 to 0.131, p=0.0869). |
| E1. Protein-cognition correlations, pooled versus within diagnosis | pooled across anchored diagnoses | NRGN | MMSE | reference | -0.1220309 |  | -0.19055231 | -0.052326169 | 0.00063166606 |  | 781 | Diagnosis and cognition were anchored to the CSF draw; cognition was required within ±90 days. |
| E1. Protein-cognition correlations, pooled versus within diagnosis | within diagnosis, Fisher-z weighted by n-3 | NRGN | MMSE | reference | -0.028875734 |  | -0.09909809 | 0.041632805 | 0.42224521 |  | 781 | CN rho 0.007 (-0.141 to 0.156, n=175); MCI rho -0.030 (-0.128 to 0.068, n=400); AD rho -0.057 (-0.192 to 0.081, n=206); partial rho adjusted for diagnosis -0.027 (-0.097 to 0.043, p=0.445). |
| E1. Protein-cognition correlations, pooled versus within diagnosis | pooled across anchored diagnoses | NRGN | ADAS-Cog13 | reference | 0.16850651 |  | 0.10198653 | 0.23352626 | 9.1663613e-07 |  | 839 | Diagnosis and cognition were anchored to the CSF draw; cognition was required within ±90 days. |
| E1. Protein-cognition correlations, pooled versus within diagnosis | within diagnosis, Fisher-z weighted by n-3 | NRGN | ADAS-Cog13 | reference | 0.090215786 |  | 0.022426636 | 0.15717917 | 0.0091559012 |  | 839 | CN rho 0.019 (-0.120 to 0.157, n=201); MCI rho 0.095 (-0.001 to 0.189, n=421); AD rho 0.146 (0.014 to 0.274, n=217); partial rho adjusted for diagnosis 0.088 (0.020 to 0.155, p=0.0111). |
| E1. Protein-cognition correlations, pooled versus within diagnosis | pooled across anchored diagnoses | NRGN | CDR-SB | reference | 0.17261526 |  | 0.10358413 | 0.23999139 | 1.2766396e-06 |  | 778 | Diagnosis and cognition were anchored to the CSF draw; cognition was required within ±90 days. |
| E1. Protein-cognition correlations, pooled versus within diagnosis | within diagnosis, Fisher-z weighted by n-3 | NRGN | CDR-SB | reference | 0.089840669 |  | 0.019403014 | 0.15939091 | 0.012486323 |  | 778 | CN rho 0.043 (-0.106 to 0.191, n=174); MCI rho 0.098 (-0.001 to 0.194, n=399); AD rho 0.114 (-0.023 to 0.247, n=205); partial rho adjusted for diagnosis 0.101 (0.031 to 0.170, p=0.00483). |
| E1. Protein-cognition correlations, pooled versus within diagnosis | class summary | tau-family | mean absolute rho | tau-family | 0.31184158 |  |  |  |  |  | 12 | Pooled 0.312; within diagnosis 0.126; adjusted for diagnosis 0.138; 40% of pooled magnitude retained within diagnosis and 44% after diagnosis adjustment. |
| E1. Protein-cognition correlations, pooled versus within diagnosis | class summary | non-tau | mean absolute rho | non-tau | 0.17437096 |  |  |  |  |  | 12 | Pooled 0.174; within diagnosis 0.073; adjusted for diagnosis 0.077; 42% of pooled magnitude retained within diagnosis and 44% after diagnosis adjustment. |
| E1. Protein-cognition correlations, pooled versus within diagnosis | class summary | reference | mean absolute rho | reference | 0.15438422 |  |  |  |  |  | 3 | Pooled 0.154; within diagnosis 0.070; adjusted for diagnosis 0.072; 45% of pooled magnitude retained within diagnosis and 47% after diagnosis adjustment. |

**E2. Exact rank-covariance decomposition by diagnosis**

| **Panel** | **Analysis** | **Item** | **Stratum / contrast / outcome** | **Class** | **Estimate** | **SE** | **CI_low** | **CI_high** | **P** | **q or FDR** | **n** | **Note** |
| --- | --- | --- | --- | --- | --- | --- | --- | --- | --- | --- | --- | --- |
| E2. Exact rank-covariance decomposition by diagnosis | pair-level global-rank cross-product decomposition | pTau-217 | MMSE | tau-family | 0.72412736 |  |  |  |  |  | 781 | Pooled Spearman rho -0.294796; total global-rank cross-product -11603722.5000 = within-diagnosis -3201149.5925 + between-diagnosis -8402572.9075. The estimate column is the between/total fraction. This is an exact descriptive algebraic decomposition of observed rank covariance, not a causal attributable fraction and not a decomposition of R2. |
| E2. Exact rank-covariance decomposition by diagnosis | pair-level global-rank cross-product decomposition | pTau-231 | MMSE | tau-family | 0.72545937 |  |  |  |  |  | 781 | Pooled Spearman rho -0.290136; total global-rank cross-product -11420331.0000 = within-diagnosis -3135344.8272 + between-diagnosis -8284986.1728. The estimate column is the between/total fraction. This is an exact descriptive algebraic decomposition of observed rank covariance, not a causal attributable fraction and not a decomposition of R2. |
| E2. Exact rank-covariance decomposition by diagnosis | pair-level global-rank cross-product decomposition | pTau-181 | MMSE | tau-family | 0.73806447 |  |  |  |  |  | 781 | Pooled Spearman rho -0.314081; total global-rank cross-product -12362821.5000 = within-diagnosis -3238262.1452 + between-diagnosis -9124559.3548. The estimate column is the between/total fraction. This is an exact descriptive algebraic decomposition of observed rank covariance, not a causal attributable fraction and not a decomposition of R2. |
| E2. Exact rank-covariance decomposition by diagnosis | pair-level global-rank cross-product decomposition | MAPT | MMSE | tau-family | 0.7203971 |  |  |  |  |  | 781 | Pooled Spearman rho -0.277256; total global-rank cross-product -10913313.0000 = within-diagnosis -3051393.9528 + between-diagnosis -7861919.0472. The estimate column is the between/total fraction. This is an exact descriptive algebraic decomposition of observed rank covariance, not a causal attributable fraction and not a decomposition of R2. |
| E2. Exact rank-covariance decomposition by diagnosis | pair-level global-rank cross-product decomposition | SMOC1 | MMSE | non-tau | 0.66445252 |  |  |  |  |  | 781 | Pooled Spearman rho -0.153225; total global-rank cross-product -6031245.0000 = within-diagnosis -2023769.0849 + between-diagnosis -4007475.9151. The estimate column is the between/total fraction. This is an exact descriptive algebraic decomposition of observed rank covariance, not a causal attributable fraction and not a decomposition of R2. |
| E2. Exact rank-covariance decomposition by diagnosis | pair-level global-rank cross-product decomposition | MDH1 | MMSE | non-tau | 0.79868379 |  |  |  |  |  | 781 | Pooled Spearman rho -0.142038; total global-rank cross-product -5590871.5000 = within-diagnosis -1125533.0494 + between-diagnosis -4465338.4506. The estimate column is the between/total fraction. This is an exact descriptive algebraic decomposition of observed rank covariance, not a causal attributable fraction and not a decomposition of R2. |
| E2. Exact rank-covariance decomposition by diagnosis | pair-level global-rank cross-product decomposition | FABP3 | MMSE | non-tau | 0.72774151 |  |  |  |  |  | 781 | Pooled Spearman rho -0.215258; total global-rank cross-product -8472978.0000 = within-diagnosis -2306840.1825 + between-diagnosis -6166137.8175. The estimate column is the between/total fraction. This is an exact descriptive algebraic decomposition of observed rank covariance, not a causal attributable fraction and not a decomposition of R2. |
| E2. Exact rank-covariance decomposition by diagnosis | pair-level global-rank cross-product decomposition | VSNL1 | MMSE | non-tau | 0.71595027 |  |  |  |  |  | 781 | Pooled Spearman rho -0.128182; total global-rank cross-product -5045471.5000 = within-diagnosis -1433164.8047 + between-diagnosis -3612306.6953. The estimate column is the between/total fraction. This is an exact descriptive algebraic decomposition of observed rank covariance, not a causal attributable fraction and not a decomposition of R2. |
| E2. Exact rank-covariance decomposition by diagnosis | pair-level global-rank cross-product decomposition | NRGN | MMSE | NRGN reference | 0.85524878 |  |  |  |  |  | 781 | Pooled Spearman rho -0.122031; total global-rank cross-product -4803371.5000 = within-diagnosis -695293.8786 + between-diagnosis -4108077.6214. The estimate column is the between/total fraction. This is an exact descriptive algebraic decomposition of observed rank covariance, not a causal attributable fraction and not a decomposition of R2. |
| E2. Exact rank-covariance decomposition by diagnosis | pair-level global-rank cross-product decomposition | pTau-217 | ADAS_Cog13 | tau-family | 0.70757908 |  |  |  |  |  | 839 | Pooled Spearman rho 0.321254; total global-rank cross-product 15809649.0000 = within-diagnosis 4623072.0464 + between-diagnosis 11186576.9536. The estimate column is the between/total fraction. This is an exact descriptive algebraic decomposition of observed rank covariance, not a causal attributable fraction and not a decomposition of R2. |
| E2. Exact rank-covariance decomposition by diagnosis | pair-level global-rank cross-product decomposition | pTau-231 | ADAS_Cog13 | tau-family | 0.70108599 |  |  |  |  |  | 839 | Pooled Spearman rho 0.318900; total global-rank cross-product 15693768.0000 = within-diagnosis 4691087.1243 + between-diagnosis 11002680.8757. The estimate column is the between/total fraction. This is an exact descriptive algebraic decomposition of observed rank covariance, not a causal attributable fraction and not a decomposition of R2. |
| E2. Exact rank-covariance decomposition by diagnosis | pair-level global-rank cross-product decomposition | pTau-181 | ADAS_Cog13 | tau-family | 0.69864539 |  |  |  |  |  | 839 | Pooled Spearman rho 0.351623; total global-rank cross-product 17304149.5000 = within-diagnosis 5214685.2820 + between-diagnosis 12089464.2180. The estimate column is the between/total fraction. This is an exact descriptive algebraic decomposition of observed rank covariance, not a causal attributable fraction and not a decomposition of R2. |
| E2. Exact rank-covariance decomposition by diagnosis | pair-level global-rank cross-product decomposition | MAPT | ADAS_Cog13 | tau-family | 0.70119788 |  |  |  |  |  | 839 | Pooled Spearman rho 0.299773; total global-rank cross-product 14752514.5000 = within-diagnosis 4408082.6154 + between-diagnosis 10344431.8846. The estimate column is the between/total fraction. This is an exact descriptive algebraic decomposition of observed rank covariance, not a causal attributable fraction and not a decomposition of R2. |
| E2. Exact rank-covariance decomposition by diagnosis | pair-level global-rank cross-product decomposition | SMOC1 | ADAS_Cog13 | non-tau | 0.68351249 |  |  |  |  |  | 839 | Pooled Spearman rho 0.159418; total global-rank cross-product 7845303.5000 = within-diagnosis 2482940.5961 + between-diagnosis 5362362.9039. The estimate column is the between/total fraction. This is an exact descriptive algebraic decomposition of observed rank covariance, not a causal attributable fraction and not a decomposition of R2. |
| E2. Exact rank-covariance decomposition by diagnosis | pair-level global-rank cross-product decomposition | MDH1 | ADAS_Cog13 | non-tau | 0.70317675 |  |  |  |  |  | 839 | Pooled Spearman rho 0.170571; total global-rank cross-product 8394169.5000 = within-diagnosis 2491584.6737 + between-diagnosis 5902584.8263. The estimate column is the between/total fraction. This is an exact descriptive algebraic decomposition of observed rank covariance, not a causal attributable fraction and not a decomposition of R2. |
| E2. Exact rank-covariance decomposition by diagnosis | pair-level global-rank cross-product decomposition | FABP3 | ADAS_Cog13 | non-tau | 0.63681789 |  |  |  |  |  | 839 | Pooled Spearman rho 0.254251; total global-rank cross-product 12512241.0000 = within-diagnosis 4544222.0631 + between-diagnosis 7968018.9369. The estimate column is the between/total fraction. This is an exact descriptive algebraic decomposition of observed rank covariance, not a causal attributable fraction and not a decomposition of R2. |
| E2. Exact rank-covariance decomposition by diagnosis | pair-level global-rank cross-product decomposition | VSNL1 | ADAS_Cog13 | non-tau | 0.75035107 |  |  |  |  |  | 839 | Pooled Spearman rho 0.142971; total global-rank cross-product 7035947.0000 = within-diagnosis 1756516.6083 + between-diagnosis 5279430.3917. The estimate column is the between/total fraction. This is an exact descriptive algebraic decomposition of observed rank covariance, not a causal attributable fraction and not a decomposition of R2. |
| E2. Exact rank-covariance decomposition by diagnosis | pair-level global-rank cross-product decomposition | NRGN | ADAS_Cog13 | NRGN reference | 0.66866071 |  |  |  |  |  | 839 | Pooled Spearman rho 0.168507; total global-rank cross-product 8292584.0000 = within-diagnosis 2747658.8893 + between-diagnosis 5544925.1107. The estimate column is the between/total fraction. This is an exact descriptive algebraic decomposition of observed rank covariance, not a causal attributable fraction and not a decomposition of R2. |
| E2. Exact rank-covariance decomposition by diagnosis | pair-level global-rank cross-product decomposition | pTau-217 | CDR_SB | tau-family | 0.82117941 |  |  |  |  |  | 778 | Pooled Spearman rho 0.316385; total global-rank cross-product 12325491.5000 = within-diagnosis 2204051.7180 + between-diagnosis 10121439.7820. The estimate column is the between/total fraction. This is an exact descriptive algebraic decomposition of observed rank covariance, not a causal attributable fraction and not a decomposition of R2. |
| E2. Exact rank-covariance decomposition by diagnosis | pair-level global-rank cross-product decomposition | pTau-231 | CDR_SB | tau-family | 0.82059182 |  |  |  |  |  | 778 | Pooled Spearman rho 0.311568; total global-rank cross-product 12137837.0000 = within-diagnosis 2177627.2019 + between-diagnosis 9960209.7981. The estimate column is the between/total fraction. This is an exact descriptive algebraic decomposition of observed rank covariance, not a causal attributable fraction and not a decomposition of R2. |
| E2. Exact rank-covariance decomposition by diagnosis | pair-level global-rank cross-product decomposition | pTau-181 | CDR_SB | tau-family | 0.81699133 |  |  |  |  |  | 778 | Pooled Spearman rho 0.342103; total global-rank cross-product 13327420.0000 = within-diagnosis 2439033.3758 + between-diagnosis 10888386.6242. The estimate column is the between/total fraction. This is an exact descriptive algebraic decomposition of observed rank covariance, not a causal attributable fraction and not a decomposition of R2. |
| E2. Exact rank-covariance decomposition by diagnosis | pair-level global-rank cross-product decomposition | MAPT | CDR_SB | tau-family | 0.79705645 |  |  |  |  |  | 778 | Pooled Spearman rho 0.304225; total global-rank cross-product 11851774.5000 = within-diagnosis 2405241.1950 + between-diagnosis 9446533.3050. The estimate column is the between/total fraction. This is an exact descriptive algebraic decomposition of observed rank covariance, not a causal attributable fraction and not a decomposition of R2. |
| E2. Exact rank-covariance decomposition by diagnosis | pair-level global-rank cross-product decomposition | SMOC1 | CDR_SB | non-tau | 0.74639139 |  |  |  |  |  | 778 | Pooled Spearman rho 0.176044; total global-rank cross-product 6858202.5000 = within-diagnosis 1739299.2169 + between-diagnosis 5118903.2831. The estimate column is the between/total fraction. This is an exact descriptive algebraic decomposition of observed rank covariance, not a causal attributable fraction and not a decomposition of R2. |
| E2. Exact rank-covariance decomposition by diagnosis | pair-level global-rank cross-product decomposition | MDH1 | CDR_SB | non-tau | 0.88800908 |  |  |  |  |  | 778 | Pooled Spearman rho 0.160585; total global-rank cross-product 6255973.0000 = within-diagnosis 700612.1987 + between-diagnosis 5555360.8013. The estimate column is the between/total fraction. This is an exact descriptive algebraic decomposition of observed rank covariance, not a causal attributable fraction and not a decomposition of R2. |
| E2. Exact rank-covariance decomposition by diagnosis | pair-level global-rank cross-product decomposition | FABP3 | CDR_SB | non-tau | 0.80223655 |  |  |  |  |  | 778 | Pooled Spearman rho 0.241125; total global-rank cross-product 9393574.5000 = within-diagnosis 1857705.6849 + between-diagnosis 7535868.8151. The estimate column is the between/total fraction. This is an exact descriptive algebraic decomposition of observed rank covariance, not a causal attributable fraction and not a decomposition of R2. |
| E2. Exact rank-covariance decomposition by diagnosis | pair-level global-rank cross-product decomposition | VSNL1 | CDR_SB | non-tau | 0.80097359 |  |  |  |  |  | 778 | Pooled Spearman rho 0.148784; total global-rank cross-product 5796224.5000 = within-diagnosis 1153601.7826 + between-diagnosis 4642622.7174. The estimate column is the between/total fraction. This is an exact descriptive algebraic decomposition of observed rank covariance, not a causal attributable fraction and not a decomposition of R2. |
| E2. Exact rank-covariance decomposition by diagnosis | pair-level global-rank cross-product decomposition | NRGN | CDR_SB | NRGN reference | 0.71812814 |  |  |  |  |  | 778 | Pooled Spearman rho 0.172615; total global-rank cross-product 6724623.5000 = within-diagnosis 1895482.1601 + between-diagnosis 4829141.3399. The estimate column is the between/total fraction. This is an exact descriptive algebraic decomposition of observed rank covariance, not a causal attributable fraction and not a decomposition of R2. |
| E2. Exact rank-covariance decomposition by diagnosis | analyte-class summary | tau-family | 12 protein-outcome pairs | tau-family | 0.72479337 |  | 0.69864539 | 0.82117941 |  |  |  | Estimate is the median between/total fraction; CI_low and CI_high contain the observed pair-level range, not a confidence interval. Mean between/total fraction 0.747698. Descriptive global-rank covariance decomposition only; no causal attribution is intended. |
| E2. Exact rank-covariance decomposition by diagnosis | analyte-class summary | non-tau | 12 protein-outcome pairs | non-tau | 0.73706645 |  | 0.63681789 | 0.88800908 |  |  |  | Estimate is the median between/total fraction; CI_low and CI_high contain the observed pair-level range, not a confidence interval. Mean between/total fraction 0.743191. Descriptive global-rank covariance decomposition only; no causal attribution is intended. |
| E2. Exact rank-covariance decomposition by diagnosis | analyte-class summary | NRGN reference | 3 protein-outcome pairs | NRGN reference | 0.71812814 |  | 0.66866071 | 0.85524878 |  |  |  | Estimate is the median between/total fraction; CI_low and CI_high contain the observed pair-level range, not a confidence interval. Mean between/total fraction 0.747346. Descriptive global-rank covariance decomposition only; no causal attribution is intended. |

**F. CSF-plasma sampling interval and interval-restricted comparisons**

| **Panel** | **Analysis** | **Item** | **Stratum / contrast / outcome** | **Class** | **Estimate** | **SE** | **CI_low** | **CI_high** | **P** | **q or FDR** | **n** | **Note** |
| --- | --- | --- | --- | --- | --- | --- | --- | --- | --- | --- | --- | --- |
| F. CSF-plasma sampling interval and interval-restricted comparisons | Interval distribution | paired participants |  |  | 785 |  |  |  |  |  |  |  |
| F. CSF-plasma sampling interval and interval-restricted comparisons | Interval distribution | median plasma - CSF interval (days) |  |  | 0 |  |  |  |  |  |  |  |
| F. CSF-plasma sampling interval and interval-restricted comparisons | Interval distribution | IQR (days) |  |  | -4 to 0 |  |  |  |  |  |  |  |
| F. CSF-plasma sampling interval and interval-restricted comparisons | Interval distribution | range (days) |  |  | -133 to 419 |  |  |  |  |  |  |  |
| F. CSF-plasma sampling interval and interval-restricted comparisons | Interval distribution | same day |  |  | 512 (65.2%) |  |  |  |  |  |  |  |
| F. CSF-plasma sampling interval and interval-restricted comparisons | Interval distribution | within 30 days |  |  | 715 (91.1%) |  |  |  |  |  |  |  |
| F. CSF-plasma sampling interval and interval-restricted comparisons | Interval distribution | within 90 days |  |  | 752 (95.8%) |  |  |  |  |  |  |  |
| F. CSF-plasma sampling interval and interval-restricted comparisons | Interval distribution | within 365 days |  |  | 784 (99.9%) |  |  |  |  |  |  |  |
| F. CSF-plasma sampling interval and interval-restricted comparisons | CSF-plasma correlation for the same analyte | pTau-181 | same day | tau-family | 0.295 |  |  |  |  |  | 512 |  |
| F. CSF-plasma sampling interval and interval-restricted comparisons | CSF-plasma correlation for the same analyte | pTau-217 | same day | tau-family | 0.366 |  |  |  |  |  | 512 |  |
| F. CSF-plasma sampling interval and interval-restricted comparisons | CSF-plasma correlation for the same analyte | pTau-231 | same day | tau-family | 0.309 |  |  |  |  |  | 512 |  |
| F. CSF-plasma sampling interval and interval-restricted comparisons | CSF-plasma correlation for the same analyte | MAPT | same day | tau-family | 0.206 |  |  |  |  |  | 512 |  |
| F. CSF-plasma sampling interval and interval-restricted comparisons | CSF-plasma correlation for the same analyte | SMOC1 | same day | non-tau | 0.059 |  |  |  |  |  | 512 |  |
| F. CSF-plasma sampling interval and interval-restricted comparisons | CSF-plasma correlation for the same analyte | MDH1 | same day | non-tau | -0.057 |  |  |  |  |  | 512 |  |
| F. CSF-plasma sampling interval and interval-restricted comparisons | CSF-plasma correlation for the same analyte | FABP3 | same day | non-tau | 0.055 |  |  |  |  |  | 512 |  |
| F. CSF-plasma sampling interval and interval-restricted comparisons | CSF-plasma correlation for the same analyte | VSNL1 | same day | non-tau | 0.031 |  |  |  |  |  | 512 |  |
| F. CSF-plasma sampling interval and interval-restricted comparisons | CSF-plasma correlation for the same analyte | NRGN | same day |  | 0.017 |  |  |  |  |  | 512 |  |
| F. CSF-plasma sampling interval and interval-restricted comparisons | CSF-plasma correlation for the same analyte | GFAP | same day |  | 0.175 |  |  |  |  |  | 512 |  |
| F. CSF-plasma sampling interval and interval-restricted comparisons | CSF-plasma correlation for the same analyte | NEFL | same day |  | 0.273 |  |  |  |  |  | 512 |  |
| F. CSF-plasma sampling interval and interval-restricted comparisons | CSF-plasma correlation for the same analyte | pTau-181 | within 30d | tau-family | 0.277 |  |  |  |  |  | 715 |  |
| F. CSF-plasma sampling interval and interval-restricted comparisons | CSF-plasma correlation for the same analyte | pTau-217 | within 30d | tau-family | 0.349 |  |  |  |  |  | 715 |  |
| F. CSF-plasma sampling interval and interval-restricted comparisons | CSF-plasma correlation for the same analyte | pTau-231 | within 30d | tau-family | 0.285 |  |  |  |  |  | 715 |  |
| F. CSF-plasma sampling interval and interval-restricted comparisons | CSF-plasma correlation for the same analyte | MAPT | within 30d | tau-family | 0.178 |  |  |  |  |  | 715 |  |
| F. CSF-plasma sampling interval and interval-restricted comparisons | CSF-plasma correlation for the same analyte | SMOC1 | within 30d | non-tau | 0.064 |  |  |  |  |  | 715 |  |
| F. CSF-plasma sampling interval and interval-restricted comparisons | CSF-plasma correlation for the same analyte | MDH1 | within 30d | non-tau | -0.03 |  |  |  |  |  | 715 |  |
| F. CSF-plasma sampling interval and interval-restricted comparisons | CSF-plasma correlation for the same analyte | FABP3 | within 30d | non-tau | 0.036 |  |  |  |  |  | 715 |  |
| F. CSF-plasma sampling interval and interval-restricted comparisons | CSF-plasma correlation for the same analyte | VSNL1 | within 30d | non-tau | 0.059 |  |  |  |  |  | 715 |  |
| F. CSF-plasma sampling interval and interval-restricted comparisons | CSF-plasma correlation for the same analyte | NRGN | within 30d |  | -0.014 |  |  |  |  |  | 715 |  |
| F. CSF-plasma sampling interval and interval-restricted comparisons | CSF-plasma correlation for the same analyte | GFAP | within 30d |  | 0.174 |  |  |  |  |  | 715 |  |
| F. CSF-plasma sampling interval and interval-restricted comparisons | CSF-plasma correlation for the same analyte | NEFL | within 30d |  | 0.226 |  |  |  |  |  | 715 |  |
| F. CSF-plasma sampling interval and interval-restricted comparisons | CSF-plasma correlation for the same analyte | pTau-181 | within 90d | tau-family | 0.269 |  |  |  |  |  | 752 |  |
| F. CSF-plasma sampling interval and interval-restricted comparisons | CSF-plasma correlation for the same analyte | pTau-217 | within 90d | tau-family | 0.346 |  |  |  |  |  | 752 |  |
| F. CSF-plasma sampling interval and interval-restricted comparisons | CSF-plasma correlation for the same analyte | pTau-231 | within 90d | tau-family | 0.28 |  |  |  |  |  | 752 |  |
| F. CSF-plasma sampling interval and interval-restricted comparisons | CSF-plasma correlation for the same analyte | MAPT | within 90d | tau-family | 0.161 |  |  |  |  |  | 752 |  |
| F. CSF-plasma sampling interval and interval-restricted comparisons | CSF-plasma correlation for the same analyte | SMOC1 | within 90d | non-tau | 0.06 |  |  |  |  |  | 752 |  |
| F. CSF-plasma sampling interval and interval-restricted comparisons | CSF-plasma correlation for the same analyte | MDH1 | within 90d | non-tau | -0.03 |  |  |  |  |  | 752 |  |
| F. CSF-plasma sampling interval and interval-restricted comparisons | CSF-plasma correlation for the same analyte | FABP3 | within 90d | non-tau | 0.03 |  |  |  |  |  | 752 |  |
| F. CSF-plasma sampling interval and interval-restricted comparisons | CSF-plasma correlation for the same analyte | VSNL1 | within 90d | non-tau | 0.05 |  |  |  |  |  | 752 |  |
| F. CSF-plasma sampling interval and interval-restricted comparisons | CSF-plasma correlation for the same analyte | NRGN | within 90d |  | -0.011 |  |  |  |  |  | 752 |  |
| F. CSF-plasma sampling interval and interval-restricted comparisons | CSF-plasma correlation for the same analyte | GFAP | within 90d |  | 0.175 |  |  |  |  |  | 752 |  |
| F. CSF-plasma sampling interval and interval-restricted comparisons | CSF-plasma correlation for the same analyte | NEFL | within 90d |  | 0.214 |  |  |  |  |  | 752 |  |
| F. CSF-plasma sampling interval and interval-restricted comparisons | CSF-plasma correlation for the same analyte | pTau-181 | within 365d | tau-family | 0.273 |  |  |  |  |  | 784 |  |
| F. CSF-plasma sampling interval and interval-restricted comparisons | CSF-plasma correlation for the same analyte | pTau-217 | within 365d | tau-family | 0.359 |  |  |  |  |  | 784 |  |
| F. CSF-plasma sampling interval and interval-restricted comparisons | CSF-plasma correlation for the same analyte | pTau-231 | within 365d | tau-family | 0.287 |  |  |  |  |  | 784 |  |
| F. CSF-plasma sampling interval and interval-restricted comparisons | CSF-plasma correlation for the same analyte | MAPT | within 365d | tau-family | 0.162 |  |  |  |  |  | 784 |  |
| F. CSF-plasma sampling interval and interval-restricted comparisons | CSF-plasma correlation for the same analyte | SMOC1 | within 365d | non-tau | 0.059 |  |  |  |  |  | 784 |  |
| F. CSF-plasma sampling interval and interval-restricted comparisons | CSF-plasma correlation for the same analyte | MDH1 | within 365d | non-tau | -0.027 |  |  |  |  |  | 784 |  |
| F. CSF-plasma sampling interval and interval-restricted comparisons | CSF-plasma correlation for the same analyte | FABP3 | within 365d | non-tau | 0.039 |  |  |  |  |  | 784 |  |
| F. CSF-plasma sampling interval and interval-restricted comparisons | CSF-plasma correlation for the same analyte | VSNL1 | within 365d | non-tau | 0.047 |  |  |  |  |  | 784 |  |
| F. CSF-plasma sampling interval and interval-restricted comparisons | CSF-plasma correlation for the same analyte | NRGN | within 365d |  | -0.012 |  |  |  |  |  | 784 |  |
| F. CSF-plasma sampling interval and interval-restricted comparisons | CSF-plasma correlation for the same analyte | GFAP | within 365d |  | 0.186 |  |  |  |  |  | 784 |  |
| F. CSF-plasma sampling interval and interval-restricted comparisons | CSF-plasma correlation for the same analyte | NEFL | within 365d |  | 0.216 |  |  |  |  |  | 784 |  |
| F. CSF-plasma sampling interval and interval-restricted comparisons | CSF-plasma correlation for the same analyte | pTau-181 | all | tau-family | 0.274 |  |  |  |  |  | 785 |  |
| F. CSF-plasma sampling interval and interval-restricted comparisons | CSF-plasma correlation for the same analyte | pTau-217 | all | tau-family | 0.359 |  |  |  |  |  | 785 |  |
| F. CSF-plasma sampling interval and interval-restricted comparisons | CSF-plasma correlation for the same analyte | pTau-231 | all | tau-family | 0.287 |  |  |  |  |  | 785 |  |
| F. CSF-plasma sampling interval and interval-restricted comparisons | CSF-plasma correlation for the same analyte | MAPT | all | tau-family | 0.162 |  |  |  |  |  | 785 |  |
| F. CSF-plasma sampling interval and interval-restricted comparisons | CSF-plasma correlation for the same analyte | SMOC1 | all | non-tau | 0.06 |  |  |  |  |  | 785 |  |
| F. CSF-plasma sampling interval and interval-restricted comparisons | CSF-plasma correlation for the same analyte | MDH1 | all | non-tau | -0.027 |  |  |  |  |  | 785 |  |
| F. CSF-plasma sampling interval and interval-restricted comparisons | CSF-plasma correlation for the same analyte | FABP3 | all | non-tau | 0.041 |  |  |  |  |  | 785 |  |
| F. CSF-plasma sampling interval and interval-restricted comparisons | CSF-plasma correlation for the same analyte | VSNL1 | all | non-tau | 0.049 |  |  |  |  |  | 785 |  |
| F. CSF-plasma sampling interval and interval-restricted comparisons | CSF-plasma correlation for the same analyte | NRGN | all |  | -0.011 |  |  |  |  |  | 785 |  |
| F. CSF-plasma sampling interval and interval-restricted comparisons | CSF-plasma correlation for the same analyte | GFAP | all |  | 0.189 |  |  |  |  |  | 785 |  |
| F. CSF-plasma sampling interval and interval-restricted comparisons | CSF-plasma correlation for the same analyte | NEFL | all |  | 0.218 |  |  |  |  |  | 785 |  |
| F. CSF-plasma sampling interval and interval-restricted comparisons | Steiger (1980) test on midranks, CSF vs plasma pTau-217 | MMSE | same day | tau-family | 1.5856432 |  |  |  | 0.11282028 | 0.67692165 | 465 | CSF-cognition rho -0.254; plasma-cognition rho -0.332; CSF-plasma rho 0.370. Cognition was required within ±90 days of the CSF draw. Holm-adjusted p in the q/FDR column controls all 15 interval-window Steiger comparisons. |
| F. CSF-plasma sampling interval and interval-restricted comparisons | Steiger (1980) test on midranks, CSF vs plasma pTau-217 | ADAS-Cog13 | same day | tau-family | -3.4658137 |  |  |  | 0.00052862939 | 0.0079294409 | 498 | CSF-cognition rho 0.285; plasma-cognition rho 0.443; CSF-plasma rho 0.375. Cognition was required within ±90 days of the CSF draw. Holm-adjusted p in the q/FDR column controls all 15 interval-window Steiger comparisons. |
| F. CSF-plasma sampling interval and interval-restricted comparisons | Steiger (1980) test on midranks, CSF vs plasma pTau-217 | CDR-SB | same day | tau-family | -1.3543559 |  |  |  | 0.17562285 | 0.87811425 | 463 | CSF-cognition rho 0.297; plasma-cognition rho 0.363; CSF-plasma rho 0.368. Cognition was required within ±90 days of the CSF draw. Holm-adjusted p in the q/FDR column controls all 15 interval-window Steiger comparisons. |
| F. CSF-plasma sampling interval and interval-restricted comparisons | Steiger (1980) test on midranks, CSF vs plasma pTau-217 | MMSE | absolute interval <=30 days | tau-family | 1.3140021 |  |  |  | 0.18884551 | 0.87811425 | 658 | CSF-cognition rho -0.268; plasma-cognition rho -0.323; CSF-plasma rho 0.350. Cognition was required within ±90 days of the CSF draw. Holm-adjusted p in the q/FDR column controls all 15 interval-window Steiger comparisons. |
| F. CSF-plasma sampling interval and interval-restricted comparisons | Steiger (1980) test on midranks, CSF vs plasma pTau-217 | ADAS-Cog13 | absolute interval <=30 days | tau-family | -3.1190894 |  |  |  | 0.0018141093 | 0.02539753 | 698 | CSF-cognition rho 0.305; plasma-cognition rho 0.427; CSF-plasma rho 0.356. Cognition was required within ±90 days of the CSF draw. Holm-adjusted p in the q/FDR column controls all 15 interval-window Steiger comparisons. |
| F. CSF-plasma sampling interval and interval-restricted comparisons | Steiger (1980) test on midranks, CSF vs plasma pTau-217 | CDR-SB | absolute interval <=30 days | tau-family | -1.8647085 |  |  |  | 0.062222275 | 0.62222275 | 656 | CSF-cognition rho 0.298; plasma-cognition rho 0.375; CSF-plasma rho 0.349. Cognition was required within ±90 days of the CSF draw. Holm-adjusted p in the q/FDR column controls all 15 interval-window Steiger comparisons. |
| F. CSF-plasma sampling interval and interval-restricted comparisons | Steiger (1980) test on midranks, CSF vs plasma pTau-217 | MMSE | absolute interval <=90 days | tau-family | 1.2143423 |  |  |  | 0.22461704 | 0.87811425 | 685 | CSF-cognition rho -0.269; plasma-cognition rho -0.319; CSF-plasma rho 0.361. Cognition was required within ±90 days of the CSF draw. Holm-adjusted p in the q/FDR column controls all 15 interval-window Steiger comparisons. |
| F. CSF-plasma sampling interval and interval-restricted comparisons | Steiger (1980) test on midranks, CSF vs plasma pTau-217 | ADAS-Cog13 | absolute interval <=90 days | tau-family | -3.0408757 |  |  |  | 0.0023589117 | 0.026913834 | 731 | CSF-cognition rho 0.303; plasma-cognition rho 0.420; CSF-plasma rho 0.350. Cognition was required within ±90 days of the CSF draw. Holm-adjusted p in the q/FDR column controls all 15 interval-window Steiger comparisons. |
| F. CSF-plasma sampling interval and interval-restricted comparisons | Steiger (1980) test on midranks, CSF vs plasma pTau-217 | CDR-SB | absolute interval <=90 days | tau-family | -1.8169853 |  |  |  | 0.069219367 | 0.62222275 | 683 | CSF-cognition rho 0.301; plasma-cognition rho 0.373; CSF-plasma rho 0.360. Cognition was required within ±90 days of the CSF draw. Holm-adjusted p in the q/FDR column controls all 15 interval-window Steiger comparisons. |
| F. CSF-plasma sampling interval and interval-restricted comparisons | Steiger (1980) test on midranks, CSF vs plasma pTau-217 | MMSE | absolute interval <=365 days | tau-family | 1.1900296 |  |  |  | 0.23403474 | 0.87811425 | 711 | CSF-cognition rho -0.262; plasma-cognition rho -0.309; CSF-plasma rho 0.373. Cognition was required within ±90 days of the CSF draw. Holm-adjusted p in the q/FDR column controls all 15 interval-window Steiger comparisons. |
| F. CSF-plasma sampling interval and interval-restricted comparisons | Steiger (1980) test on midranks, CSF vs plasma pTau-217 | ADAS-Cog13 | absolute interval <=365 days | tau-family | -3.0784333 |  |  |  | 0.0020809214 | 0.026913834 | 758 | CSF-cognition rho 0.300; plasma-cognition rho 0.415; CSF-plasma rho 0.362. Cognition was required within ±90 days of the CSF draw. Holm-adjusted p in the q/FDR column controls all 15 interval-window Steiger comparisons. |
| F. CSF-plasma sampling interval and interval-restricted comparisons | Steiger (1980) test on midranks, CSF vs plasma pTau-217 | CDR-SB | absolute interval <=365 days | tau-family | -1.8179596 |  |  |  | 0.069070309 | 0.62222275 | 709 | CSF-cognition rho 0.299; plasma-cognition rho 0.369; CSF-plasma rho 0.372. Cognition was required within ±90 days of the CSF draw. Holm-adjusted p in the q/FDR column controls all 15 interval-window Steiger comparisons. |
| F. CSF-plasma sampling interval and interval-restricted comparisons | Steiger (1980) test on midranks, CSF vs plasma pTau-217 | MMSE | all pairs | tau-family | 1.1923507 |  |  |  | 0.23312377 | 0.87811425 | 712 | CSF-cognition rho -0.262; plasma-cognition rho -0.309; CSF-plasma rho 0.373. Cognition was required within ±90 days of the CSF draw. Holm-adjusted p in the q/FDR column controls all 15 interval-window Steiger comparisons. |
| F. CSF-plasma sampling interval and interval-restricted comparisons | Steiger (1980) test on midranks, CSF vs plasma pTau-217 | ADAS-Cog13 | all pairs | tau-family | -3.0799584 |  |  |  | 0.0020702949 | 0.026913834 | 759 | CSF-cognition rho 0.300; plasma-cognition rho 0.415; CSF-plasma rho 0.362. Cognition was required within ±90 days of the CSF draw. Holm-adjusted p in the q/FDR column controls all 15 interval-window Steiger comparisons. |
| F. CSF-plasma sampling interval and interval-restricted comparisons | Steiger (1980) test on midranks, CSF vs plasma pTau-217 | CDR-SB | all pairs | tau-family | -1.8202004 |  |  |  | 0.068728485 | 0.62222275 | 710 | CSF-cognition rho 0.299; plasma-cognition rho 0.370; CSF-plasma rho 0.372. Cognition was required within ±90 days of the CSF draw. Holm-adjusted p in the q/FDR column controls all 15 interval-window Steiger comparisons. |

**G. Adjustment by tau measures that do not share the CSF assay plate**

| **Panel** | **Analysis** | **Item** | **Stratum / contrast / outcome** | **Class** | **Estimate** | **SE** | **CI_low** | **CI_high** | **P** | **q or FDR** | **n** | **Note** |
| --- | --- | --- | --- | --- | --- | --- | --- | --- | --- | --- | --- | --- |
| G. Adjustment by tau measures that do not share the CSF assay plate | matched subset for plasma pTau-217 (n=785) \| adjusted for CSF NULISA pTau-217 | pTau-217 | T+ vs T- | tau-family |  |  |  |  |  |  | 785 | N/A: this analyte is the adjustment covariate, so the model is a self-adjustment and yields no interpretable estimate |
| G. Adjustment by tau measures that do not share the CSF assay plate | matched subset for plasma pTau-217 (n=785) \| adjusted for plasma pTau-217 | pTau-217 | T+ vs T- | tau-family | 1.132 |  |  |  |  | 7.21e-29 | 785 | unadjusted log2FC 1.307; attenuation -13%; covariate correlation with Elecsys p-tau181 (log scale) r=0.43; shares the CSF assay plate with the candidate proteins: no |
| G. Adjustment by tau measures that do not share the CSF assay plate | matched subset for plasma pTau-217 (n=785) \| adjusted for CSF NULISA pTau-217 | pTau-231 | T+ vs T- | tau-family | 0.027 |  |  |  |  | 0.0933 | 785 | unadjusted log2FC 1.212; attenuation -98%; covariate correlation with Elecsys p-tau181 (log scale) r=0.60; shares the CSF assay plate with the candidate proteins: yes |
| G. Adjustment by tau measures that do not share the CSF assay plate | matched subset for plasma pTau-217 (n=785) \| adjusted for plasma pTau-217 | pTau-231 | T+ vs T- | tau-family | 1.05 |  |  |  |  | 1.63e-29 | 785 | unadjusted log2FC 1.212; attenuation -13%; covariate correlation with Elecsys p-tau181 (log scale) r=0.43; shares the CSF assay plate with the candidate proteins: no |
| G. Adjustment by tau measures that do not share the CSF assay plate | matched subset for plasma pTau-217 (n=785) \| adjusted for CSF NULISA pTau-217 | pTau-181 | T+ vs T- | tau-family | 0.077 |  |  |  |  | 0.000233 | 785 | unadjusted log2FC 1.192; attenuation -94%; covariate correlation with Elecsys p-tau181 (log scale) r=0.60; shares the CSF assay plate with the candidate proteins: yes |
| G. Adjustment by tau measures that do not share the CSF assay plate | matched subset for plasma pTau-217 (n=785) \| adjusted for plasma pTau-217 | pTau-181 | T+ vs T- | tau-family | 1.021 |  |  |  |  | 7.49e-31 | 785 | unadjusted log2FC 1.192; attenuation -14%; covariate correlation with Elecsys p-tau181 (log scale) r=0.43; shares the CSF assay plate with the candidate proteins: no |
| G. Adjustment by tau measures that do not share the CSF assay plate | matched subset for plasma pTau-217 (n=785) \| adjusted for CSF NULISA pTau-217 | MAPT | T+ vs T- | tau-family | 0.159 |  |  |  |  | 2.16e-07 | 785 | unadjusted log2FC 1.006; attenuation -84%; covariate correlation with Elecsys p-tau181 (log scale) r=0.60; shares the CSF assay plate with the candidate proteins: yes |
| G. Adjustment by tau measures that do not share the CSF assay plate | matched subset for plasma pTau-217 (n=785) \| adjusted for plasma pTau-217 | MAPT | T+ vs T- | tau-family | 0.921 |  |  |  |  | 5.84e-37 | 785 | unadjusted log2FC 1.006; attenuation -8%; covariate correlation with Elecsys p-tau181 (log scale) r=0.43; shares the CSF assay plate with the candidate proteins: no |
| G. Adjustment by tau measures that do not share the CSF assay plate | matched subset for plasma pTau-217 (n=785) \| adjusted for CSF NULISA pTau-217 | SMOC1 | T+ vs T- | non-tau | 0.165 |  |  |  |  | 0.0154 | 785 | unadjusted log2FC 0.783; attenuation -79%; covariate correlation with Elecsys p-tau181 (log scale) r=0.60; shares the CSF assay plate with the candidate proteins: yes |
| G. Adjustment by tau measures that do not share the CSF assay plate | matched subset for plasma pTau-217 (n=785) \| adjusted for plasma pTau-217 | SMOC1 | T+ vs T- | non-tau | 0.778 |  |  |  |  | 2.12e-25 | 785 | unadjusted log2FC 0.783; attenuation -1%; covariate correlation with Elecsys p-tau181 (log scale) r=0.43; shares the CSF assay plate with the candidate proteins: no |
| G. Adjustment by tau measures that do not share the CSF assay plate | matched subset for plasma pTau-217 (n=785) \| adjusted for CSF NULISA pTau-217 | MDH1 | T+ vs T- | non-tau | 0.228 |  |  |  |  | 0.00578 | 785 | unadjusted log2FC 0.684; attenuation -67%; covariate correlation with Elecsys p-tau181 (log scale) r=0.60; shares the CSF assay plate with the candidate proteins: yes |
| G. Adjustment by tau measures that do not share the CSF assay plate | matched subset for plasma pTau-217 (n=785) \| adjusted for plasma pTau-217 | MDH1 | T+ vs T- | non-tau | 0.7 |  |  |  |  | 1.35e-20 | 785 | unadjusted log2FC 0.684; attenuation +2%; covariate correlation with Elecsys p-tau181 (log scale) r=0.43; shares the CSF assay plate with the candidate proteins: no |
| G. Adjustment by tau measures that do not share the CSF assay plate | matched subset for plasma pTau-217 (n=785) \| adjusted for CSF NULISA pTau-217 | FABP3 | T+ vs T- | non-tau | 0.125 |  |  |  |  | 0.0146 | 785 | unadjusted log2FC 0.541; attenuation -77%; covariate correlation with Elecsys p-tau181 (log scale) r=0.60; shares the CSF assay plate with the candidate proteins: yes |
| G. Adjustment by tau measures that do not share the CSF assay plate | matched subset for plasma pTau-217 (n=785) \| adjusted for plasma pTau-217 | FABP3 | T+ vs T- | non-tau | 0.529 |  |  |  |  | 3.02e-23 | 785 | unadjusted log2FC 0.541; attenuation -2%; covariate correlation with Elecsys p-tau181 (log scale) r=0.43; shares the CSF assay plate with the candidate proteins: no |
| G. Adjustment by tau measures that do not share the CSF assay plate | matched subset for plasma pTau-217 (n=785) \| adjusted for CSF NULISA pTau-217 | VSNL1 | T+ vs T- | non-tau | 0.193 |  |  |  |  | 0.000448 | 785 | unadjusted log2FC 0.564; attenuation -66%; covariate correlation with Elecsys p-tau181 (log scale) r=0.60; shares the CSF assay plate with the candidate proteins: yes |
| G. Adjustment by tau measures that do not share the CSF assay plate | matched subset for plasma pTau-217 (n=785) \| adjusted for plasma pTau-217 | VSNL1 | T+ vs T- | non-tau | 0.573 |  |  |  |  | 1.25e-26 | 785 | unadjusted log2FC 0.564; attenuation +2%; covariate correlation with Elecsys p-tau181 (log scale) r=0.43; shares the CSF assay plate with the candidate proteins: no |
| G. Adjustment by tau measures that do not share the CSF assay plate | matched subset for plasma pTau-217 (n=785) \| adjusted for CSF NULISA pTau-217 | pTau-217 | N+ vs N- | tau-family |  |  |  |  |  |  | 785 | N/A: this analyte is the adjustment covariate, so the model is a self-adjustment and yields no interpretable estimate |
| G. Adjustment by tau measures that do not share the CSF assay plate | matched subset for plasma pTau-217 (n=785) \| adjusted for plasma pTau-217 | pTau-217 | N+ vs N- | tau-family | 1.032 |  |  |  |  | 1.4e-25 | 785 | unadjusted log2FC 1.213; attenuation -15%; covariate correlation with Elecsys p-tau181 (log scale) r=0.43; shares the CSF assay plate with the candidate proteins: no |
| G. Adjustment by tau measures that do not share the CSF assay plate | matched subset for plasma pTau-217 (n=785) \| adjusted for CSF NULISA pTau-217 | pTau-231 | N+ vs N- | tau-family | 0.038 |  |  |  |  | 0.00422 | 785 | unadjusted log2FC 1.136; attenuation -97%; covariate correlation with Elecsys p-tau181 (log scale) r=0.60; shares the CSF assay plate with the candidate proteins: yes |
| G. Adjustment by tau measures that do not share the CSF assay plate | matched subset for plasma pTau-217 (n=785) \| adjusted for plasma pTau-217 | pTau-231 | N+ vs N- | tau-family | 0.971 |  |  |  |  | 8.94e-27 | 785 | unadjusted log2FC 1.136; attenuation -14%; covariate correlation with Elecsys p-tau181 (log scale) r=0.43; shares the CSF assay plate with the candidate proteins: no |
| G. Adjustment by tau measures that do not share the CSF assay plate | matched subset for plasma pTau-217 (n=785) \| adjusted for CSF NULISA pTau-217 | pTau-181 | N+ vs N- | tau-family | 0.092 |  |  |  |  | 2.95e-07 | 785 | unadjusted log2FC 1.124; attenuation -92%; covariate correlation with Elecsys p-tau181 (log scale) r=0.60; shares the CSF assay plate with the candidate proteins: yes |
| G. Adjustment by tau measures that do not share the CSF assay plate | matched subset for plasma pTau-217 (n=785) \| adjusted for plasma pTau-217 | pTau-181 | N+ vs N- | tau-family | 0.952 |  |  |  |  | 1.96e-28 | 785 | unadjusted log2FC 1.124; attenuation -15%; covariate correlation with Elecsys p-tau181 (log scale) r=0.43; shares the CSF assay plate with the candidate proteins: no |
| G. Adjustment by tau measures that do not share the CSF assay plate | matched subset for plasma pTau-217 (n=785) \| adjusted for CSF NULISA pTau-217 | MAPT | N+ vs N- | tau-family | 0.195 |  |  |  |  | 1.37e-12 | 785 | unadjusted log2FC 0.975; attenuation -80%; covariate correlation with Elecsys p-tau181 (log scale) r=0.60; shares the CSF assay plate with the candidate proteins: yes |
| G. Adjustment by tau measures that do not share the CSF assay plate | matched subset for plasma pTau-217 (n=785) \| adjusted for plasma pTau-217 | MAPT | N+ vs N- | tau-family | 0.888 |  |  |  |  | 2.2e-36 | 785 | unadjusted log2FC 0.975; attenuation -9%; covariate correlation with Elecsys p-tau181 (log scale) r=0.43; shares the CSF assay plate with the candidate proteins: no |
| G. Adjustment by tau measures that do not share the CSF assay plate | matched subset for plasma pTau-217 (n=785) \| adjusted for CSF NULISA pTau-217 | SMOC1 | N+ vs N- | non-tau | 0.204 |  |  |  |  | 0.000855 | 785 | unadjusted log2FC 0.770; attenuation -73%; covariate correlation with Elecsys p-tau181 (log scale) r=0.60; shares the CSF assay plate with the candidate proteins: yes |
| G. Adjustment by tau measures that do not share the CSF assay plate | matched subset for plasma pTau-217 (n=785) \| adjusted for plasma pTau-217 | SMOC1 | N+ vs N- | non-tau | 0.761 |  |  |  |  | 5.93e-26 | 785 | unadjusted log2FC 0.770; attenuation -1%; covariate correlation with Elecsys p-tau181 (log scale) r=0.43; shares the CSF assay plate with the candidate proteins: no |
| G. Adjustment by tau measures that do not share the CSF assay plate | matched subset for plasma pTau-217 (n=785) \| adjusted for CSF NULISA pTau-217 | MDH1 | N+ vs N- | non-tau | 0.293 |  |  |  |  | 4.08e-05 | 785 | unadjusted log2FC 0.705; attenuation -58%; covariate correlation with Elecsys p-tau181 (log scale) r=0.60; shares the CSF assay plate with the candidate proteins: yes |
| G. Adjustment by tau measures that do not share the CSF assay plate | matched subset for plasma pTau-217 (n=785) \| adjusted for plasma pTau-217 | MDH1 | N+ vs N- | non-tau | 0.719 |  |  |  |  | 3.39e-23 | 785 | unadjusted log2FC 0.705; attenuation +2%; covariate correlation with Elecsys p-tau181 (log scale) r=0.43; shares the CSF assay plate with the candidate proteins: no |
| G. Adjustment by tau measures that do not share the CSF assay plate | matched subset for plasma pTau-217 (n=785) \| adjusted for CSF NULISA pTau-217 | FABP3 | N+ vs N- | non-tau | 0.163 |  |  |  |  | 0.000353 | 785 | unadjusted log2FC 0.542; attenuation -70%; covariate correlation with Elecsys p-tau181 (log scale) r=0.60; shares the CSF assay plate with the candidate proteins: yes |
| G. Adjustment by tau measures that do not share the CSF assay plate | matched subset for plasma pTau-217 (n=785) \| adjusted for plasma pTau-217 | FABP3 | N+ vs N- | non-tau | 0.53 |  |  |  |  | 8.33e-25 | 785 | unadjusted log2FC 0.542; attenuation -2%; covariate correlation with Elecsys p-tau181 (log scale) r=0.43; shares the CSF assay plate with the candidate proteins: no |
| G. Adjustment by tau measures that do not share the CSF assay plate | matched subset for plasma pTau-217 (n=785) \| adjusted for CSF NULISA pTau-217 | VSNL1 | N+ vs N- | non-tau | 0.223 |  |  |  |  | 4.32e-06 | 785 | unadjusted log2FC 0.562; attenuation -60%; covariate correlation with Elecsys p-tau181 (log scale) r=0.60; shares the CSF assay plate with the candidate proteins: yes |
| G. Adjustment by tau measures that do not share the CSF assay plate | matched subset for plasma pTau-217 (n=785) \| adjusted for plasma pTau-217 | VSNL1 | N+ vs N- | non-tau | 0.568 |  |  |  |  | 8.85e-28 | 785 | unadjusted log2FC 0.562; attenuation +1%; covariate correlation with Elecsys p-tau181 (log scale) r=0.43; shares the CSF assay plate with the candidate proteins: no |
| G. Adjustment by tau measures that do not share the CSF assay plate | matched subset for plasma pTau-217 (n=741) \| adjusted for CSF NULISA pTau-217 | pTau-217 | T+N+ vs T-N- | tau-family |  |  |  |  |  |  | 741 | N/A: this analyte is the adjustment covariate, so the model is a self-adjustment and yields no interpretable estimate |
| G. Adjustment by tau measures that do not share the CSF assay plate | matched subset for plasma pTau-217 (n=741) \| adjusted for plasma pTau-217 | pTau-217 | T+N+ vs T-N- | tau-family | 1.163 |  |  |  |  | 7.61e-28 | 741 | unadjusted log2FC 1.345; attenuation -14%; covariate correlation with Elecsys p-tau181 (log scale) r=0.45; shares the CSF assay plate with the candidate proteins: no |
| G. Adjustment by tau measures that do not share the CSF assay plate | matched subset for plasma pTau-217 (n=741) \| adjusted for CSF NULISA pTau-217 | pTau-231 | T+N+ vs T-N- | tau-family | 0.033 |  |  |  |  | 0.0344 | 741 | unadjusted log2FC 1.252; attenuation -97%; covariate correlation with Elecsys p-tau181 (log scale) r=0.61; shares the CSF assay plate with the candidate proteins: yes |
| G. Adjustment by tau measures that do not share the CSF assay plate | matched subset for plasma pTau-217 (n=741) \| adjusted for plasma pTau-217 | pTau-231 | T+N+ vs T-N- | tau-family | 1.085 |  |  |  |  | 1.18e-28 | 741 | unadjusted log2FC 1.252; attenuation -13%; covariate correlation with Elecsys p-tau181 (log scale) r=0.45; shares the CSF assay plate with the candidate proteins: no |
| G. Adjustment by tau measures that do not share the CSF assay plate | matched subset for plasma pTau-217 (n=741) \| adjusted for CSF NULISA pTau-217 | pTau-181 | T+N+ vs T-N- | tau-family | 0.089 |  |  |  |  | 1.04e-05 | 741 | unadjusted log2FC 1.234; attenuation -93%; covariate correlation with Elecsys p-tau181 (log scale) r=0.61; shares the CSF assay plate with the candidate proteins: yes |
| G. Adjustment by tau measures that do not share the CSF assay plate | matched subset for plasma pTau-217 (n=741) \| adjusted for plasma pTau-217 | pTau-181 | T+N+ vs T-N- | tau-family | 1.058 |  |  |  |  | 3.42e-30 | 741 | unadjusted log2FC 1.234; attenuation -14%; covariate correlation with Elecsys p-tau181 (log scale) r=0.45; shares the CSF assay plate with the candidate proteins: no |
| G. Adjustment by tau measures that do not share the CSF assay plate | matched subset for plasma pTau-217 (n=741) \| adjusted for CSF NULISA pTau-217 | MAPT | T+N+ vs T-N- | tau-family | 0.187 |  |  |  |  | 6.53e-10 | 741 | unadjusted log2FC 1.055; attenuation -82%; covariate correlation with Elecsys p-tau181 (log scale) r=0.61; shares the CSF assay plate with the candidate proteins: yes |
| G. Adjustment by tau measures that do not share the CSF assay plate | matched subset for plasma pTau-217 (n=741) \| adjusted for plasma pTau-217 | MAPT | T+N+ vs T-N- | tau-family | 0.968 |  |  |  |  | 2.17e-37 | 741 | unadjusted log2FC 1.055; attenuation -8%; covariate correlation with Elecsys p-tau181 (log scale) r=0.45; shares the CSF assay plate with the candidate proteins: no |
| G. Adjustment by tau measures that do not share the CSF assay plate | matched subset for plasma pTau-217 (n=741) \| adjusted for CSF NULISA pTau-217 | SMOC1 | T+N+ vs T-N- | non-tau | 0.195 |  |  |  |  | 0.00346 | 741 | unadjusted log2FC 0.834; attenuation -77%; covariate correlation with Elecsys p-tau181 (log scale) r=0.61; shares the CSF assay plate with the candidate proteins: yes |
| G. Adjustment by tau measures that do not share the CSF assay plate | matched subset for plasma pTau-217 (n=741) \| adjusted for plasma pTau-217 | SMOC1 | T+N+ vs T-N- | non-tau | 0.828 |  |  |  |  | 1.1e-26 | 741 | unadjusted log2FC 0.834; attenuation -1%; covariate correlation with Elecsys p-tau181 (log scale) r=0.45; shares the CSF assay plate with the candidate proteins: no |
| G. Adjustment by tau measures that do not share the CSF assay plate | matched subset for plasma pTau-217 (n=741) \| adjusted for CSF NULISA pTau-217 | MDH1 | T+N+ vs T-N- | non-tau | 0.271 |  |  |  |  | 0.000854 | 741 | unadjusted log2FC 0.738; attenuation -63%; covariate correlation with Elecsys p-tau181 (log scale) r=0.61; shares the CSF assay plate with the candidate proteins: yes |
| G. Adjustment by tau measures that do not share the CSF assay plate | matched subset for plasma pTau-217 (n=741) \| adjusted for plasma pTau-217 | MDH1 | T+N+ vs T-N- | non-tau | 0.752 |  |  |  |  | 8.37e-22 | 741 | unadjusted log2FC 0.738; attenuation +2%; covariate correlation with Elecsys p-tau181 (log scale) r=0.45; shares the CSF assay plate with the candidate proteins: no |
| G. Adjustment by tau measures that do not share the CSF assay plate | matched subset for plasma pTau-217 (n=741) \| adjusted for CSF NULISA pTau-217 | FABP3 | T+N+ vs T-N- | non-tau | 0.148 |  |  |  |  | 0.00307 | 741 | unadjusted log2FC 0.577; attenuation -74%; covariate correlation with Elecsys p-tau181 (log scale) r=0.61; shares the CSF assay plate with the candidate proteins: yes |
| G. Adjustment by tau measures that do not share the CSF assay plate | matched subset for plasma pTau-217 (n=741) \| adjusted for plasma pTau-217 | FABP3 | T+N+ vs T-N- | non-tau | 0.562 |  |  |  |  | 2.47e-24 | 741 | unadjusted log2FC 0.577; attenuation -3%; covariate correlation with Elecsys p-tau181 (log scale) r=0.45; shares the CSF assay plate with the candidate proteins: no |
| G. Adjustment by tau measures that do not share the CSF assay plate | matched subset for plasma pTau-217 (n=741) \| adjusted for CSF NULISA pTau-217 | VSNL1 | T+N+ vs T-N- | non-tau | 0.222 |  |  |  |  | 4.05e-05 | 741 | unadjusted log2FC 0.601; attenuation -63%; covariate correlation with Elecsys p-tau181 (log scale) r=0.61; shares the CSF assay plate with the candidate proteins: yes |
| G. Adjustment by tau measures that do not share the CSF assay plate | matched subset for plasma pTau-217 (n=741) \| adjusted for plasma pTau-217 | VSNL1 | T+N+ vs T-N- | non-tau | 0.609 |  |  |  |  | 7.61e-28 | 741 | unadjusted log2FC 0.601; attenuation +1%; covariate correlation with Elecsys p-tau181 (log scale) r=0.45; shares the CSF assay plate with the candidate proteins: no |
| G. Adjustment by tau measures that do not share the CSF assay plate | matched subset for tau-PET SUVR (n=192) \| adjusted for CSF NULISA pTau-217 | pTau-217 | T+ vs T- | tau-family |  |  |  |  |  |  | 192 | N/A: this analyte is the adjustment covariate, so the model is a self-adjustment and yields no interpretable estimate |
| G. Adjustment by tau measures that do not share the CSF assay plate | matched subset for tau-PET SUVR (n=192) \| adjusted for tau-PET SUVR | pTau-217 | T+ vs T- | tau-family | 0.594 |  |  |  |  | 0.0508 | 192 | unadjusted log2FC 0.725; attenuation -18%; covariate correlation with Elecsys p-tau181 (log scale) r=0.45; shares the CSF assay plate with the candidate proteins: no |
| G. Adjustment by tau measures that do not share the CSF assay plate | matched subset for tau-PET SUVR (n=192) \| adjusted for CSF NULISA pTau-217 | pTau-231 | T+ vs T- | tau-family | -0.02 |  |  |  |  | 0.867 | 192 | unadjusted log2FC 0.652; attenuation -103%; covariate correlation with Elecsys p-tau181 (log scale) r=0.47; shares the CSF assay plate with the candidate proteins: yes |
| G. Adjustment by tau measures that do not share the CSF assay plate | matched subset for tau-PET SUVR (n=192) \| adjusted for tau-PET SUVR | pTau-231 | T+ vs T- | tau-family | 0.526 |  |  |  |  | 0.0578 | 192 | unadjusted log2FC 0.652; attenuation -19%; covariate correlation with Elecsys p-tau181 (log scale) r=0.45; shares the CSF assay plate with the candidate proteins: no |
| G. Adjustment by tau measures that do not share the CSF assay plate | matched subset for tau-PET SUVR (n=192) \| adjusted for CSF NULISA pTau-217 | pTau-181 | T+ vs T- | tau-family | 0.021 |  |  |  |  | 0.895 | 192 | unadjusted log2FC 0.644; attenuation -97%; covariate correlation with Elecsys p-tau181 (log scale) r=0.47; shares the CSF assay plate with the candidate proteins: yes |
| G. Adjustment by tau measures that do not share the CSF assay plate | matched subset for tau-PET SUVR (n=192) \| adjusted for tau-PET SUVR | pTau-181 | T+ vs T- | tau-family | 0.496 |  |  |  |  | 0.0578 | 192 | unadjusted log2FC 0.644; attenuation -23%; covariate correlation with Elecsys p-tau181 (log scale) r=0.45; shares the CSF assay plate with the candidate proteins: no |
| G. Adjustment by tau measures that do not share the CSF assay plate | matched subset for tau-PET SUVR (n=192) \| adjusted for CSF NULISA pTau-217 | MAPT | T+ vs T- | tau-family | 0.073 |  |  |  |  | 0.867 | 192 | unadjusted log2FC 0.537; attenuation -86%; covariate correlation with Elecsys p-tau181 (log scale) r=0.47; shares the CSF assay plate with the candidate proteins: yes |
| G. Adjustment by tau measures that do not share the CSF assay plate | matched subset for tau-PET SUVR (n=192) \| adjusted for tau-PET SUVR | MAPT | T+ vs T- | tau-family | 0.454 |  |  |  |  | 0.0411 | 192 | unadjusted log2FC 0.537; attenuation -15%; covariate correlation with Elecsys p-tau181 (log scale) r=0.45; shares the CSF assay plate with the candidate proteins: no |
| G. Adjustment by tau measures that do not share the CSF assay plate | matched subset for tau-PET SUVR (n=192) \| adjusted for CSF NULISA pTau-217 | SMOC1 | T+ vs T- | non-tau | 0.119 |  |  |  |  | 0.867 | 192 | unadjusted log2FC 0.466; attenuation -75%; covariate correlation with Elecsys p-tau181 (log scale) r=0.47; shares the CSF assay plate with the candidate proteins: yes |
| G. Adjustment by tau measures that do not share the CSF assay plate | matched subset for tau-PET SUVR (n=192) \| adjusted for tau-PET SUVR | SMOC1 | T+ vs T- | non-tau | 0.472 |  |  |  |  | 0.0411 | 192 | unadjusted log2FC 0.466; attenuation +1%; covariate correlation with Elecsys p-tau181 (log scale) r=0.45; shares the CSF assay plate with the candidate proteins: no |
| G. Adjustment by tau measures that do not share the CSF assay plate | matched subset for tau-PET SUVR (n=192) \| adjusted for CSF NULISA pTau-217 | MDH1 | T+ vs T- | non-tau | 0.191 |  |  |  |  | 0.867 | 192 | unadjusted log2FC 0.440; attenuation -57%; covariate correlation with Elecsys p-tau181 (log scale) r=0.47; shares the CSF assay plate with the candidate proteins: yes |
| G. Adjustment by tau measures that do not share the CSF assay plate | matched subset for tau-PET SUVR (n=192) \| adjusted for tau-PET SUVR | MDH1 | T+ vs T- | non-tau | 0.435 |  |  |  |  | 0.0487 | 192 | unadjusted log2FC 0.440; attenuation -1%; covariate correlation with Elecsys p-tau181 (log scale) r=0.45; shares the CSF assay plate with the candidate proteins: no |
| G. Adjustment by tau measures that do not share the CSF assay plate | matched subset for tau-PET SUVR (n=192) \| adjusted for CSF NULISA pTau-217 | FABP3 | T+ vs T- | non-tau | 0.093 |  |  |  |  | 0.867 | 192 | unadjusted log2FC 0.339; attenuation -73%; covariate correlation with Elecsys p-tau181 (log scale) r=0.47; shares the CSF assay plate with the candidate proteins: yes |
| G. Adjustment by tau measures that do not share the CSF assay plate | matched subset for tau-PET SUVR (n=192) \| adjusted for tau-PET SUVR | FABP3 | T+ vs T- | non-tau | 0.301 |  |  |  |  | 0.0487 | 192 | unadjusted log2FC 0.339; attenuation -11%; covariate correlation with Elecsys p-tau181 (log scale) r=0.45; shares the CSF assay plate with the candidate proteins: no |
| G. Adjustment by tau measures that do not share the CSF assay plate | matched subset for tau-PET SUVR (n=192) \| adjusted for CSF NULISA pTau-217 | VSNL1 | T+ vs T- | non-tau | 0.08 |  |  |  |  | 0.867 | 192 | unadjusted log2FC 0.301; attenuation -73%; covariate correlation with Elecsys p-tau181 (log scale) r=0.47; shares the CSF assay plate with the candidate proteins: yes |
| G. Adjustment by tau measures that do not share the CSF assay plate | matched subset for tau-PET SUVR (n=192) \| adjusted for tau-PET SUVR | VSNL1 | T+ vs T- | non-tau | 0.305 |  |  |  |  | 0.0508 | 192 | unadjusted log2FC 0.301; attenuation +1%; covariate correlation with Elecsys p-tau181 (log scale) r=0.45; shares the CSF assay plate with the candidate proteins: no |
| G. Adjustment by tau measures that do not share the CSF assay plate | matched subset for tau-PET SUVR (n=192) \| adjusted for CSF NULISA pTau-217 | pTau-217 | N+ vs N- | tau-family |  |  |  |  |  |  | 192 | N/A: this analyte is the adjustment covariate, so the model is a self-adjustment and yields no interpretable estimate |
| G. Adjustment by tau measures that do not share the CSF assay plate | matched subset for tau-PET SUVR (n=192) \| adjusted for tau-PET SUVR | pTau-217 | N+ vs N- | tau-family | 0.555 |  |  |  |  | 0.0406 | 192 | unadjusted log2FC 0.680; attenuation -18%; covariate correlation with Elecsys p-tau181 (log scale) r=0.45; shares the CSF assay plate with the candidate proteins: no |
| G. Adjustment by tau measures that do not share the CSF assay plate | matched subset for tau-PET SUVR (n=192) \| adjusted for CSF NULISA pTau-217 | pTau-231 | N+ vs N- | tau-family | -0.02 |  |  |  |  | 0.718 | 192 | unadjusted log2FC 0.611; attenuation -103%; covariate correlation with Elecsys p-tau181 (log scale) r=0.47; shares the CSF assay plate with the candidate proteins: yes |
| G. Adjustment by tau measures that do not share the CSF assay plate | matched subset for tau-PET SUVR (n=192) \| adjusted for tau-PET SUVR | pTau-231 | N+ vs N- | tau-family | 0.49 |  |  |  |  | 0.05 | 192 | unadjusted log2FC 0.611; attenuation -20%; covariate correlation with Elecsys p-tau181 (log scale) r=0.45; shares the CSF assay plate with the candidate proteins: no |
| G. Adjustment by tau measures that do not share the CSF assay plate | matched subset for tau-PET SUVR (n=192) \| adjusted for CSF NULISA pTau-217 | pTau-181 | N+ vs N- | tau-family | 0.033 |  |  |  |  | 0.718 | 192 | unadjusted log2FC 0.618; attenuation -95%; covariate correlation with Elecsys p-tau181 (log scale) r=0.47; shares the CSF assay plate with the candidate proteins: yes |
| G. Adjustment by tau measures that do not share the CSF assay plate | matched subset for tau-PET SUVR (n=192) \| adjusted for tau-PET SUVR | pTau-181 | N+ vs N- | tau-family | 0.48 |  |  |  |  | 0.0406 | 192 | unadjusted log2FC 0.618; attenuation -22%; covariate correlation with Elecsys p-tau181 (log scale) r=0.45; shares the CSF assay plate with the candidate proteins: no |
| G. Adjustment by tau measures that do not share the CSF assay plate | matched subset for tau-PET SUVR (n=192) \| adjusted for CSF NULISA pTau-217 | MAPT | N+ vs N- | tau-family | 0.11 |  |  |  |  | 0.708 | 192 | unadjusted log2FC 0.543; attenuation -80%; covariate correlation with Elecsys p-tau181 (log scale) r=0.47; shares the CSF assay plate with the candidate proteins: yes |
| G. Adjustment by tau measures that do not share the CSF assay plate | matched subset for tau-PET SUVR (n=192) \| adjusted for tau-PET SUVR | MAPT | N+ vs N- | tau-family | 0.466 |  |  |  |  | 0.0177 | 192 | unadjusted log2FC 0.543; attenuation -14%; covariate correlation with Elecsys p-tau181 (log scale) r=0.45; shares the CSF assay plate with the candidate proteins: no |
| G. Adjustment by tau measures that do not share the CSF assay plate | matched subset for tau-PET SUVR (n=192) \| adjusted for CSF NULISA pTau-217 | SMOC1 | N+ vs N- | non-tau | 0.147 |  |  |  |  | 0.708 | 192 | unadjusted log2FC 0.472; attenuation -69%; covariate correlation with Elecsys p-tau181 (log scale) r=0.47; shares the CSF assay plate with the candidate proteins: yes |
| G. Adjustment by tau measures that do not share the CSF assay plate | matched subset for tau-PET SUVR (n=192) \| adjusted for tau-PET SUVR | SMOC1 | N+ vs N- | non-tau | 0.471 |  |  |  |  | 0.0177 | 192 | unadjusted log2FC 0.472; attenuation -0%; covariate correlation with Elecsys p-tau181 (log scale) r=0.45; shares the CSF assay plate with the candidate proteins: no |
| G. Adjustment by tau measures that do not share the CSF assay plate | matched subset for tau-PET SUVR (n=192) \| adjusted for CSF NULISA pTau-217 | MDH1 | N+ vs N- | non-tau | 0.222 |  |  |  |  | 0.708 | 192 | unadjusted log2FC 0.454; attenuation -51%; covariate correlation with Elecsys p-tau181 (log scale) r=0.47; shares the CSF assay plate with the candidate proteins: yes |
| G. Adjustment by tau measures that do not share the CSF assay plate | matched subset for tau-PET SUVR (n=192) \| adjusted for tau-PET SUVR | MDH1 | N+ vs N- | non-tau | 0.445 |  |  |  |  | 0.0223 | 192 | unadjusted log2FC 0.454; attenuation -2%; covariate correlation with Elecsys p-tau181 (log scale) r=0.45; shares the CSF assay plate with the candidate proteins: no |
| G. Adjustment by tau measures that do not share the CSF assay plate | matched subset for tau-PET SUVR (n=192) \| adjusted for CSF NULISA pTau-217 | FABP3 | N+ vs N- | non-tau | 0.112 |  |  |  |  | 0.708 | 192 | unadjusted log2FC 0.342; attenuation -67%; covariate correlation with Elecsys p-tau181 (log scale) r=0.47; shares the CSF assay plate with the candidate proteins: yes |
| G. Adjustment by tau measures that do not share the CSF assay plate | matched subset for tau-PET SUVR (n=192) \| adjusted for tau-PET SUVR | FABP3 | N+ vs N- | non-tau | 0.306 |  |  |  |  | 0.0223 | 192 | unadjusted log2FC 0.342; attenuation -11%; covariate correlation with Elecsys p-tau181 (log scale) r=0.45; shares the CSF assay plate with the candidate proteins: no |
| G. Adjustment by tau measures that do not share the CSF assay plate | matched subset for tau-PET SUVR (n=192) \| adjusted for CSF NULISA pTau-217 | VSNL1 | N+ vs N- | non-tau | 0.119 |  |  |  |  | 0.708 | 192 | unadjusted log2FC 0.324; attenuation -63%; covariate correlation with Elecsys p-tau181 (log scale) r=0.47; shares the CSF assay plate with the candidate proteins: yes |
| G. Adjustment by tau measures that do not share the CSF assay plate | matched subset for tau-PET SUVR (n=192) \| adjusted for tau-PET SUVR | VSNL1 | N+ vs N- | non-tau | 0.326 |  |  |  |  | 0.0217 | 192 | unadjusted log2FC 0.324; attenuation +1%; covariate correlation with Elecsys p-tau181 (log scale) r=0.45; shares the CSF assay plate with the candidate proteins: no |
| G. Adjustment by tau measures that do not share the CSF assay plate | matched subset for tau-PET SUVR (n=183) \| adjusted for CSF NULISA pTau-217 | pTau-217 | T+N+ vs T-N- | tau-family |  |  |  |  |  |  | 183 | N/A: this analyte is the adjustment covariate, so the model is a self-adjustment and yields no interpretable estimate |
| G. Adjustment by tau measures that do not share the CSF assay plate | matched subset for tau-PET SUVR (n=183) \| adjusted for tau-PET SUVR | pTau-217 | T+N+ vs T-N- | tau-family | 0.611 |  |  |  |  | 0.05 | 183 | unadjusted log2FC 0.745; attenuation -18%; covariate correlation with Elecsys p-tau181 (log scale) r=0.46; shares the CSF assay plate with the candidate proteins: no |
| G. Adjustment by tau measures that do not share the CSF assay plate | matched subset for tau-PET SUVR (n=183) \| adjusted for CSF NULISA pTau-217 | pTau-231 | T+N+ vs T-N- | tau-family | -0.022 |  |  |  |  | 0.842 | 183 | unadjusted log2FC 0.669; attenuation -103%; covariate correlation with Elecsys p-tau181 (log scale) r=0.47; shares the CSF assay plate with the candidate proteins: yes |
| G. Adjustment by tau measures that do not share the CSF assay plate | matched subset for tau-PET SUVR (n=183) \| adjusted for tau-PET SUVR | pTau-231 | T+N+ vs T-N- | tau-family | 0.538 |  |  |  |  | 0.0554 | 183 | unadjusted log2FC 0.669; attenuation -20%; covariate correlation with Elecsys p-tau181 (log scale) r=0.46; shares the CSF assay plate with the candidate proteins: no |
| G. Adjustment by tau measures that do not share the CSF assay plate | matched subset for tau-PET SUVR (n=183) \| adjusted for CSF NULISA pTau-217 | pTau-181 | T+N+ vs T-N- | tau-family | 0.025 |  |  |  |  | 0.855 | 183 | unadjusted log2FC 0.665; attenuation -96%; covariate correlation with Elecsys p-tau181 (log scale) r=0.47; shares the CSF assay plate with the candidate proteins: yes |
| G. Adjustment by tau measures that do not share the CSF assay plate | matched subset for tau-PET SUVR (n=183) \| adjusted for tau-PET SUVR | pTau-181 | T+N+ vs T-N- | tau-family | 0.515 |  |  |  |  | 0.0549 | 183 | unadjusted log2FC 0.665; attenuation -22%; covariate correlation with Elecsys p-tau181 (log scale) r=0.46; shares the CSF assay plate with the candidate proteins: no |
| G. Adjustment by tau measures that do not share the CSF assay plate | matched subset for tau-PET SUVR (n=183) \| adjusted for CSF NULISA pTau-217 | MAPT | T+N+ vs T-N- | tau-family | 0.09 |  |  |  |  | 0.842 | 183 | unadjusted log2FC 0.566; attenuation -84%; covariate correlation with Elecsys p-tau181 (log scale) r=0.47; shares the CSF assay plate with the candidate proteins: yes |
| G. Adjustment by tau measures that do not share the CSF assay plate | matched subset for tau-PET SUVR (n=183) \| adjusted for tau-PET SUVR | MAPT | T+N+ vs T-N- | tau-family | 0.482 |  |  |  |  | 0.0287 | 183 | unadjusted log2FC 0.566; attenuation -15%; covariate correlation with Elecsys p-tau181 (log scale) r=0.46; shares the CSF assay plate with the candidate proteins: no |
| G. Adjustment by tau measures that do not share the CSF assay plate | matched subset for tau-PET SUVR (n=183) \| adjusted for CSF NULISA pTau-217 | SMOC1 | T+N+ vs T-N- | non-tau | 0.142 |  |  |  |  | 0.842 | 183 | unadjusted log2FC 0.500; attenuation -72%; covariate correlation with Elecsys p-tau181 (log scale) r=0.47; shares the CSF assay plate with the candidate proteins: yes |
| G. Adjustment by tau measures that do not share the CSF assay plate | matched subset for tau-PET SUVR (n=183) \| adjusted for tau-PET SUVR | SMOC1 | T+N+ vs T-N- | non-tau | 0.493 |  |  |  |  | 0.0287 | 183 | unadjusted log2FC 0.500; attenuation -1%; covariate correlation with Elecsys p-tau181 (log scale) r=0.46; shares the CSF assay plate with the candidate proteins: no |
| G. Adjustment by tau measures that do not share the CSF assay plate | matched subset for tau-PET SUVR (n=183) \| adjusted for CSF NULISA pTau-217 | MDH1 | T+N+ vs T-N- | non-tau | 0.207 |  |  |  |  | 0.842 | 183 | unadjusted log2FC 0.464; attenuation -55%; covariate correlation with Elecsys p-tau181 (log scale) r=0.47; shares the CSF assay plate with the candidate proteins: yes |
| G. Adjustment by tau measures that do not share the CSF assay plate | matched subset for tau-PET SUVR (n=183) \| adjusted for tau-PET SUVR | MDH1 | T+N+ vs T-N- | non-tau | 0.455 |  |  |  |  | 0.0367 | 183 | unadjusted log2FC 0.464; attenuation -2%; covariate correlation with Elecsys p-tau181 (log scale) r=0.46; shares the CSF assay plate with the candidate proteins: no |
| G. Adjustment by tau measures that do not share the CSF assay plate | matched subset for tau-PET SUVR (n=183) \| adjusted for CSF NULISA pTau-217 | FABP3 | T+N+ vs T-N- | non-tau | 0.105 |  |  |  |  | 0.842 | 183 | unadjusted log2FC 0.359; attenuation -71%; covariate correlation with Elecsys p-tau181 (log scale) r=0.47; shares the CSF assay plate with the candidate proteins: yes |
| G. Adjustment by tau measures that do not share the CSF assay plate | matched subset for tau-PET SUVR (n=183) \| adjusted for tau-PET SUVR | FABP3 | T+N+ vs T-N- | non-tau | 0.318 |  |  |  |  | 0.0367 | 183 | unadjusted log2FC 0.359; attenuation -11%; covariate correlation with Elecsys p-tau181 (log scale) r=0.46; shares the CSF assay plate with the candidate proteins: no |
| G. Adjustment by tau measures that do not share the CSF assay plate | matched subset for tau-PET SUVR (n=183) \| adjusted for CSF NULISA pTau-217 | VSNL1 | T+N+ vs T-N- | non-tau | 0.102 |  |  |  |  | 0.842 | 183 | unadjusted log2FC 0.329; attenuation -69%; covariate correlation with Elecsys p-tau181 (log scale) r=0.47; shares the CSF assay plate with the candidate proteins: yes |
| G. Adjustment by tau measures that do not share the CSF assay plate | matched subset for tau-PET SUVR (n=183) \| adjusted for tau-PET SUVR | VSNL1 | T+N+ vs T-N- | non-tau | 0.33 |  |  |  |  | 0.0367 | 183 | unadjusted log2FC 0.329; attenuation +0%; covariate correlation with Elecsys p-tau181 (log scale) r=0.46; shares the CSF assay plate with the candidate proteins: no |
