# Supplementary material for: Cross‐cohort NULISAseq identifies tau‐anchored CSF and plasma‐specific signatures across the amyloid‐positive Alzheimer's disease
Source: Alzheimers Dement. 2026 Oct 12;22(10):e71915. doi: 10.1002/alz.71915 (PMC13656197; doi:10.1002/alz.71915)
Supplement: Supplementary file 3 [file ALZ-22-e71915-s003.docx]

**Supplementary Table S7. Full limma differential-expression results for the prespecified ATN contrasts in Beijing CSF, ADNI CSF, and ADNI plasma.**

**Legend**

| **Field** | **Description** |
| --- | --- |
| Title | Supplementary Table S7. Full limma differential-expression results for the pre-specified ATN contrasts in Beijing CSF, ADNI CSF, and ADNI plasma. |
| Cohort | Beijing (n=60 A⁺ AD, Fujirebio INNOTEST ATN classification) or ADNI (n=865 A⁺ baseline with CSF NULISA SampleQC=passed; n=793 A⁺ for plasma; Roche Elecsys ATN classification). |
| Fluid | CSF (cerebrospinal fluid) or plasma. Both fluids were profiled on the NULISAseq CNS Disease Panel (127 protein targets). |
| Analyte family | APOE and CRP had no finite NPQ values in the ADNI CSF export because reverse-curve quantification was considered unreliable for this matrix. Although NPQ values were available in Beijing CSF, both analytes were excluded from both CSF cohorts to maintain an identical cross-cohort testing family. The APOE4 panel target was excluded from all differential-abundance families because the data-provider guidance specifies interpreting it as a binary carrier/non-carrier readout rather than as a routine continuous differential-abundance endpoint, even though an NPQ value is provided. The resulting testing families comprised 124 CSF analytes in each cohort and 126 plasma analytes. Genotype-derived APOE ε4 carrier status remained a model covariate throughout. |
| Contrast | Three pre-specified ATN contrasts: "T+ vs T-", "N+ vs N-", and "A+T+N+ vs A+T-N-" (extreme contrast). |
| Protein | Analyte on the NULISAseq CNS Disease Panel. |
| log₂FC | log₂ fold-change of NULISA NPQ between the two contrast groups (positive = higher in the first listed group). |
| AveExpr | Average expression (mean NPQ on the log₂ scale, across all samples in the contrast). |
| t | Moderated t-statistic from limma empirical Bayes. |
| P.Value | Nominal two-sided p-value from the moderated t-statistic. |
| FDR | Benjamini-Hochberg false discovery rate computed within each cohort × fluid × contrast combination, over the full analyte family for that fluid (124 in CSF, 126 in plasma). |
| B | Log-odds of differential expression from limma empirical Bayes. |
| N_total | Total number of participants in the contrast. |
| N_group1 | Number in the first group (e.g. T⁺ for "T+ vs T-"). |
| N_group2 | Number in the second group. |
| Pass_FDR | TRUE if FDR < 0.05. |
| Pass_both_thresholds | TRUE if FDR < 0.05 AND \|log₂FC\| ≥ 0.5. |
| Direction | "up" if log₂FC > 0, "down" if log₂FC < 0. |
| Model | limma with empirical Bayes moderation. Beijing CSF: age + sex + APOE ε4 carrier status + sample batch (all 60 Beijing samples were assayed on a single NULISA plate; the batch term is the 0/1 grouping variable recorded in the sample information file). ADNI CSF and ADNI plasma: age + sex + APOE ε4 carrier status + NULISA plate. |
| Notes | Each row is one analyte in one cohort × fluid × contrast combination. Filter Pass_both_thresholds = TRUE for the threshold-passing set used in the cross-cohort consistency framework. |

*Numeric values are displayed to eight significant digits; analyses used unrounded values.*

**Beijing | CSF | T+ vs T-**

| **Cohort** | **Fluid** | **Contrast** | **Protein** | **log₂FC** | **AveExpr** | **t** | **P.Value** | **FDR** | **B** | **N_total** | **N_group1** | **N_group2** | **Pass_FDR** | **Pass_both_thresholds** | **Direction** |
| --- | --- | --- | --- | --- | --- | --- | --- | --- | --- | --- | --- | --- | --- | --- | --- |
| Beijing | CSF | T+ vs T- | pTau-181 | 0.94245928 | 18.13809 | 4.9631499 | 6.8478854e-06 | 0.00064560069 | 3.6609424 | 60 | 44 | 16 | TRUE | TRUE | up |
| Beijing | CSF | T+ vs T- | pTau-217 | 1.0896785 | 17.149338 | 4.8453653 | 1.0412914e-05 | 0.00064560069 | 3.2665507 | 60 | 44 | 16 | TRUE | TRUE | up |
| Beijing | CSF | T+ vs T- | pTau-231 | 0.97558492 | 18.450377 | 4.5351615 | 3.0868643e-05 | 0.0012759039 | 2.2461203 | 60 | 44 | 16 | TRUE | TRUE | up |
| Beijing | CSF | T+ vs T- | MDH1 | 0.50372045 | 10.990934 | 3.5135665 | 0.00088329704 | 0.027020164 | -0.8710062 | 60 | 44 | 16 | TRUE | TRUE | up |
| Beijing | CSF | T+ vs T- | SMOC1 | 0.53306454 | 13.448316 | 3.4450468 | 0.0010895228 | 0.027020164 | -1.0635289 | 60 | 44 | 16 | TRUE | TRUE | up |
| Beijing | CSF | T+ vs T- | PSEN1 | 0.28637464 | 11.144936 | 3.3208902 | 0.0015845815 | 0.032748017 | -1.4061509 | 60 | 44 | 16 | TRUE | FALSE | up |
| Beijing | CSF | T+ vs T- | NRGN | 0.32986275 | 9.163614 | 3.1743975 | 0.0024414239 | 0.043248081 | -1.7996513 | 60 | 44 | 16 | TRUE | FALSE | up |
| Beijing | CSF | T+ vs T- | MAPT | 0.47798636 | 16.851448 | 2.9094933 | 0.0051860668 | 0.080384035 | -2.4797232 | 60 | 44 | 16 | FALSE | FALSE | up |
| Beijing | CSF | T+ vs T- | VSNL1 | 0.33756186 | 13.60885 | 2.8490149 | 0.0061265066 | 0.084409647 | -2.6289918 | 60 | 44 | 16 | FALSE | FALSE | up |
| Beijing | CSF | T+ vs T- | BACE1 | 0.29087643 | 14.665497 | 2.7154637 | 0.0087869359 | 0.10317875 | -2.9503292 | 60 | 44 | 16 | FALSE | FALSE | up |
| Beijing | CSF | T+ vs T- | FABP3 | 0.34025169 | 12.027746 | 2.6828399 | 0.0095810975 | 0.10317875 | -3.0270522 | 60 | 44 | 16 | FALSE | FALSE | up |
| Beijing | CSF | T+ vs T- | NPTX2 | 0.5360211 | 14.3925 | 2.6671858 | 0.0099850401 | 0.10317875 | -3.063615 | 60 | 44 | 16 | FALSE | FALSE | up |
| Beijing | CSF | T+ vs T- | GOT1 | 0.24533351 | 11.001013 | 2.5870615 | 0.012307062 | 0.10900541 | -3.2481712 | 60 | 44 | 16 | FALSE | FALSE | up |
| Beijing | CSF | T+ vs T- | NPTXR | 0.3779639 | 16.90649 | 2.592003 | 0.012150737 | 0.10900541 | -3.2369155 | 60 | 44 | 16 | FALSE | FALSE | up |
| Beijing | CSF | T+ vs T- | SNCB | 0.24195202 | 12.536073 | 2.4871069 | 0.015887979 | 0.13134062 | -3.4722214 | 60 | 44 | 16 | FALSE | FALSE | up |
| Beijing | CSF | T+ vs T- | DDC | 0.41288178 | 6.4644941 | 2.3511695 | 0.022263574 | 0.1725427 | -3.7655898 | 60 | 44 | 16 | FALSE | FALSE | up |
| Beijing | CSF | T+ vs T- | SOD1 | 0.32099685 | 13.477222 | 2.2881418 | 0.025930266 | 0.18913841 | -3.8970589 | 60 | 44 | 16 | FALSE | FALSE | up |
| Beijing | CSF | T+ vs T- | CX3CL1 | 0.23710445 | 10.477216 | 2.2343744 | 0.029472137 | 0.20303028 | -4.006883 | 60 | 44 | 16 | FALSE | FALSE | up |
| Beijing | CSF | T+ vs T- | TIMP3 | 0.32036628 | 8.526493 | 2.0776628 | 0.04234266 | 0.27634157 | -4.3144715 | 60 | 44 | 16 | FALSE | FALSE | up |
| Beijing | CSF | T+ vs T- | SLIT2 | 0.1667083 | 11.231703 | 2.0514507 | 0.044916564 | 0.27848269 | -4.3640674 | 60 | 44 | 16 | FALSE | FALSE | up |
| Beijing | CSF | T+ vs T- | Abeta40 | 0.56769419 | 17.208252 | 2.0267744 | 0.047462403 | 0.28025419 | -4.4102648 | 60 | 44 | 16 | FALSE | FALSE | up |
| Beijing | CSF | T+ vs T- | ENO2 | 0.28283139 | 9.386125 | 2.0007315 | 0.050283411 | 0.28341559 | -4.4584992 | 60 | 44 | 16 | FALSE | FALSE | up |
| Beijing | CSF | T+ vs T- | BASP1 | 0.087109651 | 9.9938904 | 1.9707782 | 0.053705327 | 0.28541728 | -4.5133102 | 60 | 44 | 16 | FALSE | FALSE | up |
| Beijing | CSF | T+ vs T- | pSNCA-129 | 0.23202244 | 8.4037929 | 1.9578568 | 0.055242054 | 0.28541728 | -4.536734 | 60 | 44 | 16 | FALSE | FALSE | up |
| Beijing | CSF | T+ vs T- | AGRN | 0.21951925 | 13.790963 | 1.9320139 | 0.058428635 | 0.28980603 | -4.5831796 | 60 | 44 | 16 | FALSE | FALSE | up |
| Beijing | CSF | T+ vs T- | TAFA5 | 0.30738935 | 20.025517 | 1.9117478 | 0.061036065 | 0.29109508 | -4.6192263 | 60 | 44 | 16 | FALSE | FALSE | up |
| Beijing | CSF | T+ vs T- | Abeta38 | 0.44769434 | 20.038683 | 1.8496845 | 0.069644477 | 0.29779018 | -4.7275435 | 60 | 44 | 16 | FALSE | FALSE | up |
| Beijing | CSF | T+ vs T- | IL12p70 | 0.2650854 | 9.1754639 | 1.8813169 | 0.065136829 | 0.29779018 | -4.6727284 | 60 | 44 | 16 | FALSE | FALSE | up |
| Beijing | CSF | T+ vs T- | KLK6 | 0.1323347 | 18.683566 | 1.8558944 | 0.068739369 | 0.29779018 | -4.716847 | 60 | 44 | 16 | FALSE | FALSE | up |
| Beijing | CSF | T+ vs T- | CALB2 | 0.29419657 | 15.679569 | 1.7798499 | 0.080532612 | 0.32213045 | -4.8456482 | 60 | 44 | 16 | FALSE | FALSE | up |
| Beijing | CSF | T+ vs T- | NPY | 0.29674888 | 11.430528 | 1.7802344 | 0.080468979 | 0.32213045 | -4.845009 | 60 | 44 | 16 | FALSE | FALSE | up |
| Beijing | CSF | T+ vs T- | FGF2 | 0.64145883 | 9.8206192 | 1.7441806 | 0.086620669 | 0.32548373 | -4.9044125 | 60 | 44 | 16 | FALSE | FALSE | up |
| Beijing | CSF | T+ vs T- | UCHL1 | 0.14674591 | 12.167293 | 1.7491941 | 0.08574256 | 0.32548373 | -4.896217 | 60 | 44 | 16 | FALSE | FALSE | up |
| Beijing | CSF | T+ vs T- | IL15 | 0.14580858 | 13.200736 | 1.7172725 | 0.091461858 | 0.33356678 | -4.9480391 | 60 | 44 | 16 | FALSE | FALSE | up |
| Beijing | CSF | T+ vs T- | VEGFD | 0.13692354 | 10.639803 | 1.6803843 | 0.09845944 | 0.34882773 | -5.0068566 | 60 | 44 | 16 | FALSE | FALSE | up |
| Beijing | CSF | T+ vs T- | ACHE | 0.3491848 | 12.648374 | 1.6530802 | 0.10391692 | 0.35793606 | -5.049651 | 60 | 44 | 16 | FALSE | FALSE | up |
| Beijing | CSF | T+ vs T- | CD63 | 0.14026016 | 9.7160327 | 1.6106245 | 0.1128908 | 0.37000213 | -5.1149317 | 60 | 44 | 16 | FALSE | FALSE | up |
| Beijing | CSF | T+ vs T- | IGF1R | 0.14107464 | 13.248154 | 1.6083546 | 0.11338775 | 0.37000213 | -5.1183786 | 60 | 44 | 16 | FALSE | FALSE | up |
| Beijing | CSF | T+ vs T- | PARK7 | 0.24059197 | 8.7899905 | 1.5794877 | 0.11986301 | 0.38110289 | -5.1618269 | 60 | 44 | 16 | FALSE | FALSE | up |
| Beijing | CSF | T+ vs T- | IL4 | -0.16930065 | 9.7496377 | -1.5479826 | 0.12726605 | 0.38826449 | -5.2084262 | 60 | 44 | 16 | FALSE | FALSE | down |
| Beijing | CSF | T+ vs T- | SFRP1 | 0.77019464 | 15.725117 | 1.5305817 | 0.13150894 | 0.38826449 | -5.2337955 | 60 | 44 | 16 | FALSE | FALSE | up |
| Beijing | CSF | T+ vs T- | SNCA | 0.4119134 | 6.7272227 | 1.5421465 | 0.12867673 | 0.38826449 | -5.2169642 | 60 | 44 | 16 | FALSE | FALSE | up |
| Beijing | CSF | T+ vs T- | CNTN2 | 0.24546346 | 18.485956 | 1.4167662 | 0.16209671 | 0.43695634 | -5.3932062 | 60 | 44 | 16 | FALSE | FALSE | up |
| Beijing | CSF | T+ vs T- | CST3 | 0.21463361 | 13.793429 | 1.4467071 | 0.15355972 | 0.43695634 | -5.3523739 | 60 | 44 | 16 | FALSE | FALSE | up |
| Beijing | CSF | T+ vs T- | IL17A | 0.083651638 | 8.6633345 | 1.4339024 | 0.15716676 | 0.43695634 | -5.3699334 | 60 | 44 | 16 | FALSE | FALSE | up |
| Beijing | CSF | T+ vs T- | Oligo-SNCA | 0.17891882 | 8.1936524 | 1.4236637 | 0.16009813 | 0.43695634 | -5.3838699 | 60 | 44 | 16 | FALSE | FALSE | up |
| Beijing | CSF | T+ vs T- | CRH | 0.24753 | 12.948292 | 1.3003272 | 0.19882103 | 0.52454911 | -5.5444239 | 60 | 44 | 16 | FALSE | FALSE | up |
| Beijing | CSF | T+ vs T- | CCL22 | -0.34545203 | 5.0531413 | -1.2082296 | 0.23204217 | 0.54289112 | -5.6553913 | 60 | 44 | 16 | FALSE | FALSE | down |
| Beijing | CSF | T+ vs T- | MSLN | 0.20950278 | 8.6360746 | 1.2346945 | 0.22210576 | 0.54289112 | -5.624292 | 60 | 44 | 16 | FALSE | FALSE | up |
| Beijing | CSF | T+ vs T- | PDGFRB | 0.2968451 | 7.0759348 | 1.2125451 | 0.23040013 | 0.54289112 | -5.6503636 | 60 | 44 | 16 | FALSE | FALSE | up |
| Beijing | CSF | T+ vs T- | PGK1 | 0.16764422 | 3.7118072 | 1.234788 | 0.22207125 | 0.54289112 | -5.6241811 | 60 | 44 | 16 | FALSE | FALSE | up |
| Beijing | CSF | T+ vs T- | SAA1 | -0.41271884 | 0.59637182 | -1.2628513 | 0.21188162 | 0.54289112 | -5.5905057 | 60 | 44 | 16 | FALSE | FALSE | down |
| Beijing | CSF | T+ vs T- | TEK | 0.16404016 | 6.0853717 | 1.2166056 | 0.22886287 | 0.54289112 | -5.6456175 | 60 | 44 | 16 | FALSE | FALSE | up |
| Beijing | CSF | T+ vs T- | CXCL10 | -0.28633275 | 11.099771 | -1.1443682 | 0.25734424 | 0.55213428 | -5.7278003 | 60 | 44 | 16 | FALSE | FALSE | down |
| Beijing | CSF | T+ vs T- | FLT1 | 0.21306464 | 11.601569 | 1.1407816 | 0.25882147 | 0.55213428 | -5.731756 | 60 | 44 | 16 | FALSE | FALSE | up |
| Beijing | CSF | T+ vs T- | NPTX1 | 0.21178702 | 15.5617 | 1.1500474 | 0.25501744 | 0.55213428 | -5.7215125 | 60 | 44 | 16 | FALSE | FALSE | up |
| Beijing | CSF | T+ vs T- | PDLIM5 | 0.063926756 | 7.4308726 | 1.1208003 | 0.26716175 | 0.55213428 | -5.7535768 | 60 | 44 | 16 | FALSE | FALSE | up |
| Beijing | CSF | T+ vs T- | PGF | -0.1699518 | 14.466913 | -1.1301154 | 0.26325028 | 0.55213428 | -5.74345 | 60 | 44 | 16 | FALSE | FALSE | down |
| Beijing | CSF | T+ vs T- | PRDX6 | 0.51433725 | 7.5458417 | 1.1729815 | 0.24577407 | 0.55213428 | -5.6958192 | 60 | 44 | 16 | FALSE | FALSE | up |
| Beijing | CSF | T+ vs T- | SNAP25 | 0.089648479 | 13.164438 | 1.1414372 | 0.25855103 | 0.55213428 | -5.7310339 | 60 | 44 | 16 | FALSE | FALSE | up |
| Beijing | CSF | T+ vs T- | HBA1 | 1.5463905 | 3.582214 | 1.0740144 | 0.28742749 | 0.57583855 | -5.8032274 | 60 | 44 | 16 | FALSE | FALSE | up |
| Beijing | CSF | T+ vs T- | IL13 | -0.26665037 | 8.8546879 | -1.0420947 | 0.30185085 | 0.57583855 | -5.835937 | 60 | 44 | 16 | FALSE | FALSE | down |
| Beijing | CSF | T+ vs T- | IL33 | 0.31956832 | 9.6851193 | 1.0426725 | 0.30158547 | 0.57583855 | -5.8353534 | 60 | 44 | 16 | FALSE | FALSE | up |
| Beijing | CSF | T+ vs T- | SQSTM1 | 0.13304995 | 10.84027 | 1.0482879 | 0.29901436 | 0.57583855 | -5.8296646 | 60 | 44 | 16 | FALSE | FALSE | up |
| Beijing | CSF | T+ vs T- | TARDBP | 0.38821689 | 5.4452168 | 1.0477286 | 0.29926977 | 0.57583855 | -5.8302325 | 60 | 44 | 16 | FALSE | FALSE | up |
| Beijing | CSF | T+ vs T- | pTDP43-409 | 0.058063514 | 9.9081331 | 0.97745773 | 0.3325511 | 0.62002092 | -5.8992643 | 60 | 44 | 16 | FALSE | FALSE | up |
| Beijing | CSF | T+ vs T- | VEGFA | 0.092766432 | 14.637162 | 0.97245388 | 0.33501131 | 0.62002092 | -5.9040037 | 60 | 44 | 16 | FALSE | FALSE | up |
| Beijing | CSF | T+ vs T- | RUVBL2 | 0.14101161 | 9.4233337 | 0.92473784 | 0.35907454 | 0.65478298 | -5.9480162 | 60 | 44 | 16 | FALSE | FALSE | up |
| Beijing | CSF | T+ vs T- | HTT | 0.21932797 | 7.3937432 | 0.80909392 | 0.42188931 | 0.75817789 | -6.0457571 | 60 | 44 | 16 | FALSE | FALSE | up |
| Beijing | CSF | T+ vs T- | CCL4 | -0.22352311 | 9.6808155 | -0.77670307 | 0.44060611 | 0.75984155 | -6.0708534 | 60 | 44 | 16 | FALSE | FALSE | down |
| Beijing | CSF | T+ vs T- | CXCL8 | -0.08013392 | 13.909918 | -0.77171657 | 0.44353036 | 0.75984155 | -6.0746279 | 60 | 44 | 16 | FALSE | FALSE | down |
| Beijing | CSF | T+ vs T- | KDR | 0.73110817 | 5.4327837 | 0.77005225 | 0.44450891 | 0.75984155 | -6.0758824 | 60 | 44 | 16 | FALSE | FALSE | up |
| Beijing | CSF | T+ vs T- | NGF | 0.076869207 | 6.4075521 | 0.75696834 | 0.45224557 | 0.75984155 | -6.0856522 | 60 | 44 | 16 | FALSE | FALSE | up |
| Beijing | CSF | T+ vs T- | TREM2 | 0.080838767 | 12.054522 | 0.74468148 | 0.45958158 | 0.75984155 | -6.0946775 | 60 | 44 | 16 | FALSE | FALSE | up |
| Beijing | CSF | T+ vs T- | YWHAG | 0.043100763 | 11.917281 | 0.74545789 | 0.459116 | 0.75984155 | -6.0941115 | 60 | 44 | 16 | FALSE | FALSE | up |
| Beijing | CSF | T+ vs T- | S100B | 0.088651006 | 15.635169 | 0.70273448 | 0.48513561 | 0.79153705 | -6.1243985 | 60 | 44 | 16 | FALSE | FALSE | up |
| Beijing | CSF | T+ vs T- | CHI3L1 | -0.079069005 | 14.701673 | -0.68351635 | 0.49710227 | 0.80052833 | -6.1374502 | 60 | 44 | 16 | FALSE | FALSE | down |
| Beijing | CSF | T+ vs T- | VGF | -0.060757956 | 9.6395887 | -0.66574546 | 0.50830996 | 0.80808249 | -6.1492024 | 60 | 44 | 16 | FALSE | FALSE | down |
| Beijing | CSF | T+ vs T- | IL1B | 0.075996343 | 10.762551 | 0.62385923 | 0.53525599 | 0.84014864 | -6.1756967 | 60 | 44 | 16 | FALSE | FALSE | up |
| Beijing | CSF | T+ vs T- | ANXA5 | 0.14376379 | 6.6320687 | 0.58327045 | 0.56205561 | 0.84818835 | -6.199751 | 60 | 44 | 16 | FALSE | FALSE | up |
| Beijing | CSF | T+ vs T- | ARSA | 0.10748908 | 6.2430866 | 0.55454066 | 0.58141943 | 0.84818835 | -6.2158115 | 60 | 44 | 16 | FALSE | FALSE | up |
| Beijing | CSF | T+ vs T- | GDI1 | 0.064263313 | 9.8884739 | 0.55507647 | 0.58105538 | 0.84818835 | -6.2155193 | 60 | 44 | 16 | FALSE | FALSE | up |
| Beijing | CSF | T+ vs T- | IFNG | 0.1678533 | 7.8260158 | 0.55916682 | 0.57827985 | 0.84818835 | -6.2132796 | 60 | 44 | 16 | FALSE | FALSE | up |
| Beijing | CSF | T+ vs T- | IL16 | 0.086335424 | 7.6480456 | 0.59440158 | 0.55464018 | 0.84818835 | -6.1933132 | 60 | 44 | 16 | FALSE | FALSE | up |
| Beijing | CSF | T+ vs T- | NEFL | -0.13267863 | 17.107732 | -0.58697153 | 0.55958454 | 0.84818835 | -6.1976238 | 60 | 44 | 16 | FALSE | FALSE | down |
| Beijing | CSF | T+ vs T- | IL5 | 0.04838518 | 13.233528 | 0.52299879 | 0.60304069 | 0.85950627 | -6.2325202 | 60 | 44 | 16 | FALSE | FALSE | up |
| Beijing | CSF | T+ vs T- | POSTN | -0.14344727 | 5.6536085 | -0.52712755 | 0.60018946 | 0.85950627 | -6.2303882 | 60 | 44 | 16 | FALSE | FALSE | down |
| Beijing | CSF | T+ vs T- | IL9 | 0.087909911 | 11.399257 | 0.5129592 | 0.60999979 | 0.85954516 | -6.2376354 | 60 | 44 | 16 | FALSE | FALSE | up |
| Beijing | CSF | T+ vs T- | CHIT1 | -0.69113385 | 5.0467107 | -0.47782061 | 0.63463988 | 0.87836552 | -6.2547647 | 60 | 44 | 16 | FALSE | FALSE | down |
| Beijing | CSF | T+ vs T- | IL18 | 0.1046653 | 6.6752287 | 0.4737482 | 0.63752336 | 0.87836552 | -6.256672 | 60 | 44 | 16 | FALSE | FALSE | up |
| Beijing | CSF | T+ vs T- | ICAM1 | -0.18326896 | 5.0158133 | -0.422764 | 0.67408827 | 0.89368662 | -6.2791792 | 60 | 44 | 16 | FALSE | FALSE | down |
| Beijing | CSF | T+ vs T- | IL10 | -0.093787623 | 9.5248731 | -0.41810364 | 0.67747211 | 0.89368662 | -6.2811097 | 60 | 44 | 16 | FALSE | FALSE | down |
| Beijing | CSF | T+ vs T- | NEFH | -0.097469469 | 21.18115 | -0.42407765 | 0.67313566 | 0.89368662 | -6.2786312 | 60 | 44 | 16 | FALSE | FALSE | down |
| Beijing | CSF | T+ vs T- | PTN | -0.090342417 | 13.660102 | -0.42032568 | 0.67585787 | 0.89368662 | -6.2801919 | 60 | 44 | 16 | FALSE | FALSE | down |
| Beijing | CSF | T+ vs T- | CCL3 | -0.05237833 | 10.157944 | -0.3946667 | 0.69458971 | 0.90662235 | -6.2904958 | 60 | 44 | 16 | FALSE | FALSE | down |
| Beijing | CSF | T+ vs T- | IGFBP7 | -0.027190575 | 13.987251 | -0.37967791 | 0.70562221 | 0.91142868 | -6.2962164 | 60 | 44 | 16 | FALSE | FALSE | down |
| Beijing | CSF | T+ vs T- | S100A12 | -0.17104932 | 4.2013375 | -0.34383533 | 0.73225926 | 0.936084 | -6.3090025 | 60 | 44 | 16 | FALSE | FALSE | down |
| Beijing | CSF | T+ vs T- | FCN2 | 0.14246325 | 1.5792139 | 0.30216606 | 0.76364615 | 0.94705483 | -6.322281 | 60 | 44 | 16 | FALSE | FALSE | up |
| Beijing | CSF | T+ vs T- | IL6 | -0.065219231 | 12.316498 | -0.26197367 | 0.79430405 | 0.94705483 | -6.333471 | 60 | 44 | 16 | FALSE | FALSE | down |
| Beijing | CSF | T+ vs T- | IL6R | -0.067904121 | 6.7402073 | -0.29419486 | 0.76969796 | 0.94705483 | -6.3246266 | 60 | 44 | 16 | FALSE | FALSE | down |
| Beijing | CSF | T+ vs T- | MME | 0.067760301 | 0.47404207 | 0.26266979 | 0.79377012 | 0.94705483 | -6.3332907 | 60 | 44 | 16 | FALSE | FALSE | up |
| Beijing | CSF | T+ vs T- | TNF | -0.049462029 | 9.1767678 | -0.27000322 | 0.78815142 | 0.94705483 | -6.3313626 | 60 | 44 | 16 | FALSE | FALSE | down |
| Beijing | CSF | T+ vs T- | UBB | 0.014806088 | 11.875585 | 0.27453256 | 0.78468676 | 0.94705483 | -6.3301452 | 60 | 44 | 16 | FALSE | FALSE | up |
| Beijing | CSF | T+ vs T- | YWHAZ | -0.019461167 | 11.001806 | -0.28866203 | 0.77390705 | 0.94705483 | -6.326218 | 60 | 44 | 16 | FALSE | FALSE | down |
| Beijing | CSF | T+ vs T- | CCL17 | 0.069668616 | 5.6371718 | 0.24688443 | 0.80590151 | 0.95173131 | -6.3372614 | 60 | 44 | 16 | FALSE | FALSE | up |
| Beijing | CSF | T+ vs T- | Abeta42 | 0.065707017 | 16.530256 | 0.1868879 | 0.85242471 | 0.97870985 | -6.3501125 | 60 | 44 | 16 | FALSE | FALSE | up |
| Beijing | CSF | T+ vs T- | CCL11 | 0.028715085 | 9.026989 | 0.18901392 | 0.85076604 | 0.97870985 | -6.3497178 | 60 | 44 | 16 | FALSE | FALSE | up |
| Beijing | CSF | T+ vs T- | GDNF | 0.010768784 | 9.3229102 | 0.18861858 | 0.85107443 | 0.97870985 | -6.3497915 | 60 | 44 | 16 | FALSE | FALSE | up |
| Beijing | CSF | T+ vs T- | CCL2 | 0.01839406 | 11.617946 | 0.16565879 | 0.86902276 | 0.98804538 | -6.3538094 | 60 | 44 | 16 | FALSE | FALSE | up |
| Beijing | CSF | T+ vs T- | CXCL1 | 0.02172509 | 10.457518 | 0.15613101 | 0.87649187 | 0.98804538 | -6.355324 | 60 | 44 | 16 | FALSE | FALSE | up |
| Beijing | CSF | T+ vs T- | BDNF | 0.0068010827 | 1.5611245 | 0.010742348 | 0.99146723 | 0.99146723 | -6.3673123 | 60 | 44 | 16 | FALSE | FALSE | up |
| Beijing | CSF | T+ vs T- | CCL13 | -0.038866649 | 5.1846608 | -0.10907692 | 0.91353184 | 0.99146723 | -6.3614896 | 60 | 44 | 16 | FALSE | FALSE | down |
| Beijing | CSF | T+ vs T- | CCL26 | -0.0062264653 | 8.3339029 | -0.024501371 | 0.98053989 | 0.99146723 | -6.3670726 | 60 | 44 | 16 | FALSE | FALSE | down |
| Beijing | CSF | T+ vs T- | CD40LG | 0.013679238 | 5.4008421 | 0.03287076 | 0.97389466 | 0.99146723 | -6.3668353 | 60 | 44 | 16 | FALSE | FALSE | up |
| Beijing | CSF | T+ vs T- | CSF2 | -0.0094598473 | 12.05746 | -0.050208072 | 0.96013543 | 0.99146723 | -6.3661234 | 60 | 44 | 16 | FALSE | FALSE | down |
| Beijing | CSF | T+ vs T- | FOLR1 | 0.0012540511 | 12.253085 | 0.015084379 | 0.98801854 | 0.99146723 | -6.3672568 | 60 | 44 | 16 | FALSE | FALSE | up |
| Beijing | CSF | T+ vs T- | GDF15 | -0.085720887 | 6.5810566 | -0.096388538 | 0.92355651 | 0.99146723 | -6.3627778 | 60 | 44 | 16 | FALSE | FALSE | down |
| Beijing | CSF | T+ vs T- | GFAP | -0.020767525 | 19.001968 | -0.1320746 | 0.89539921 | 0.99146723 | -6.3587493 | 60 | 44 | 16 | FALSE | FALSE | down |
| Beijing | CSF | T+ vs T- | IL2 | 0.013563922 | 8.0504759 | 0.076472871 | 0.93931576 | 0.99146723 | -6.3644791 | 60 | 44 | 16 | FALSE | FALSE | up |
| Beijing | CSF | T+ vs T- | IL7 | 0.015806044 | 10.393783 | 0.085342439 | 0.93229388 | 0.99146723 | -6.3637698 | 60 | 44 | 16 | FALSE | FALSE | up |
| Beijing | CSF | T+ vs T- | REST | 0.008998025 | 7.5395625 | 0.041618345 | 0.96695115 | 0.99146723 | -6.3665132 | 60 | 44 | 16 | FALSE | FALSE | up |
| Beijing | CSF | T+ vs T- | SFTPD | 0.013157355 | 8.0591899 | 0.043275995 | 0.96563564 | 0.99146723 | -6.3664437 | 60 | 44 | 16 | FALSE | FALSE | up |
| Beijing | CSF | T+ vs T- | TREM1 | 0.01174527 | 7.9143354 | 0.053078789 | 0.95785824 | 0.99146723 | -6.3659769 | 60 | 44 | 16 | FALSE | FALSE | up |
| Beijing | CSF | T+ vs T- | VCAM1 | 0.0094259054 | 8.8746874 | 0.067144536 | 0.94670606 | 0.99146723 | -6.3651412 | 60 | 44 | 16 | FALSE | FALSE | up |

**Beijing | CSF | N+ vs N-**

| **Cohort** | **Fluid** | **Contrast** | **Protein** | **log₂FC** | **AveExpr** | **t** | **P.Value** | **FDR** | **B** | **N_total** | **N_group1** | **N_group2** | **Pass_FDR** | **Pass_both_thresholds** | **Direction** |
| --- | --- | --- | --- | --- | --- | --- | --- | --- | --- | --- | --- | --- | --- | --- | --- |
| Beijing | CSF | N+ vs N- | SNCB | 0.39453592 | 12.536073 | 4.803646 | 1.2068679e-05 | 0.0014965162 | 3.1381381 | 60 | 40 | 20 | TRUE | FALSE | up |
| Beijing | CSF | N+ vs N- | MAPT | 0.63099741 | 16.851448 | 4.3496053 | 5.8352739e-05 | 0.0035694118 | 1.6644979 | 60 | 40 | 20 | TRUE | TRUE | up |
| Beijing | CSF | N+ vs N- | pTau-217 | 0.93986369 | 17.149338 | 4.233772 | 8.6356736e-05 | 0.0035694118 | 1.2993193 | 60 | 40 | 20 | TRUE | TRUE | up |
| Beijing | CSF | N+ vs N- | pTau-181 | 0.7640815 | 18.13809 | 3.9921239 | 0.00019272412 | 0.0047795582 | 0.55363947 | 60 | 40 | 20 | TRUE | TRUE | up |
| Beijing | CSF | N+ vs N- | pTau-231 | 0.85439502 | 18.450377 | 4.059035 | 0.00015463869 | 0.0047795582 | 0.75782663 | 60 | 40 | 20 | TRUE | TRUE | up |
| Beijing | CSF | N+ vs N- | NRGN | 0.34666215 | 9.163614 | 3.5748961 | 0.00073069815 | 0.015101095 | -0.67592732 | 60 | 40 | 20 | TRUE | FALSE | up |
| Beijing | CSF | N+ vs N- | FABP3 | 0.4003009 | 12.027746 | 3.433731 | 0.0011276771 | 0.019975994 | -1.0733394 | 60 | 40 | 20 | TRUE | FALSE | up |
| Beijing | CSF | N+ vs N- | VSNL1 | 0.37163303 | 13.60885 | 3.3781421 | 0.0013344054 | 0.020683283 | -1.2270375 | 60 | 40 | 20 | TRUE | FALSE | up |
| Beijing | CSF | N+ vs N- | UCHL1 | 0.24698513 | 12.167293 | 3.2887079 | 0.0017439941 | 0.024028364 | -1.4708754 | 60 | 40 | 20 | TRUE | FALSE | up |
| Beijing | CSF | N+ vs N- | GDI1 | 0.25498401 | 9.8884739 | 2.4251481 | 0.018555597 | 0.2300894 | -3.5798276 | 60 | 40 | 20 | FALSE | FALSE | up |
| Beijing | CSF | N+ vs N- | SNCA | 0.57531686 | 6.7272227 | 2.3201348 | 0.024006732 | 0.27062134 | -3.8024304 | 60 | 40 | 20 | FALSE | FALSE | up |
| Beijing | CSF | N+ vs N- | SOD1 | 0.29692601 | 13.477222 | 2.217386 | 0.030676683 | 0.31699239 | -4.0123967 | 60 | 40 | 20 | FALSE | FALSE | up |
| Beijing | CSF | N+ vs N- | PDLIM5 | 0.11443948 | 7.4308726 | 2.1689085 | 0.0343563 | 0.32770625 | -4.1087111 | 60 | 40 | 20 | FALSE | FALSE | up |
| Beijing | CSF | N+ vs N- | GOT1 | 0.19346132 | 11.001013 | 2.1034359 | 0.039937572 | 0.35373278 | -4.2359449 | 60 | 40 | 20 | FALSE | FALSE | up |
| Beijing | CSF | N+ vs N- | FLT1 | 0.34418591 | 11.601569 | 1.9794979 | 0.052688949 | 0.40970579 | -4.4676727 | 60 | 40 | 20 | FALSE | FALSE | up |
| Beijing | CSF | N+ vs N- | NEFH | 0.41907274 | 21.18115 | 1.9779747 | 0.052865264 | 0.40970579 | -4.4704453 | 60 | 40 | 20 | FALSE | FALSE | up |
| Beijing | CSF | N+ vs N- | PGK1 | 0.24677721 | 3.7118072 | 1.9464334 | 0.056631601 | 0.41307756 | -4.5274425 | 60 | 40 | 20 | FALSE | FALSE | up |
| Beijing | CSF | N+ vs N- | DDC | 0.30254681 | 6.4644941 | 1.7743456 | 0.081447904 | 0.43529503 | -4.8242428 | 60 | 40 | 20 | FALSE | FALSE | up |
| Beijing | CSF | N+ vs N- | ENO2 | 0.24136961 | 9.386125 | 1.7809738 | 0.080346506 | 0.43529503 | -4.8132607 | 60 | 40 | 20 | FALSE | FALSE | up |
| Beijing | CSF | N+ vs N- | IL17A | 0.098799404 | 8.6633345 | 1.7957956 | 0.077928497 | 0.43529503 | -4.7885712 | 60 | 40 | 20 | FALSE | FALSE | up |
| Beijing | CSF | N+ vs N- | IL2 | 0.28895993 | 8.0504759 | 1.7578093 | 0.084250652 | 0.43529503 | -4.8514822 | 60 | 40 | 20 | FALSE | FALSE | up |
| Beijing | CSF | N+ vs N- | MDH1 | 0.26728693 | 10.990934 | 1.8244688 | 0.073423391 | 0.43529503 | -4.7402941 | 60 | 40 | 20 | FALSE | FALSE | up |
| Beijing | CSF | N+ vs N- | Oligo-SNCA | 0.21519544 | 8.1936524 | 1.8182633 | 0.074379396 | 0.43529503 | -4.7507997 | 60 | 40 | 20 | FALSE | FALSE | up |
| Beijing | CSF | N+ vs N- | SLIT2 | 0.14482856 | 11.231703 | 1.8599187 | 0.068157916 | 0.43529503 | -4.6796736 | 60 | 40 | 20 | FALSE | FALSE | up |
| Beijing | CSF | N+ vs N- | ICAM1 | 0.66240297 | 5.0158133 | 1.6406996 | 0.10647139 | 0.52405191 | -5.0378368 | 60 | 40 | 20 | FALSE | FALSE | up |
| Beijing | CSF | N+ vs N- | PRDX6 | 0.66308214 | 7.5458417 | 1.6050775 | 0.11410808 | 0.52405191 | -5.092219 | 60 | 40 | 20 | FALSE | FALSE | up |
| Beijing | CSF | N+ vs N- | PSEN1 | 0.14120636 | 11.144936 | 1.6076505 | 0.11354202 | 0.52405191 | -5.0883272 | 60 | 40 | 20 | FALSE | FALSE | up |
| Beijing | CSF | N+ vs N- | CHIT1 | -1.9811508 | 5.0467107 | -1.4631192 | 0.14903119 | 0.52799622 | -5.2980822 | 60 | 40 | 20 | FALSE | FALSE | down |
| Beijing | CSF | N+ vs N- | HTT | 0.37630909 | 7.3937432 | 1.4778023 | 0.14506928 | 0.52799622 | -5.2776015 | 60 | 40 | 20 | FALSE | FALSE | up |
| Beijing | CSF | N+ vs N- | IL4 | -0.15558831 | 9.7496377 | -1.491984 | 0.14132177 | 0.52799622 | -5.2576411 | 60 | 40 | 20 | FALSE | FALSE | down |
| Beijing | CSF | N+ vs N- | IL6R | -0.31597054 | 6.7402073 | -1.4641585 | 0.14874801 | 0.52799622 | -5.2966388 | 60 | 40 | 20 | FALSE | FALSE | down |
| Beijing | CSF | N+ vs N- | SFTPD | -0.44245371 | 8.0591899 | -1.561654 | 0.12400963 | 0.52799622 | -5.1570413 | 60 | 40 | 20 | FALSE | FALSE | down |
| Beijing | CSF | N+ vs N- | SMOC1 | 0.23704181 | 13.448316 | 1.4902879 | 0.1417659 | 0.52799622 | -5.2600376 | 60 | 40 | 20 | FALSE | FALSE | up |
| Beijing | CSF | N+ vs N- | SQSTM1 | 0.18789273 | 10.84027 | 1.5735818 | 0.12122356 | 0.52799622 | -5.1393971 | 60 | 40 | 20 | FALSE | FALSE | up |
| Beijing | CSF | N+ vs N- | UBB | 0.075780742 | 11.875585 | 1.5034565 | 0.13834641 | 0.52799622 | -5.2413654 | 60 | 40 | 20 | FALSE | FALSE | up |
| Beijing | CSF | N+ vs N- | PARK7 | 0.20524863 | 8.7899905 | 1.4091795 | 0.16431704 | 0.56598093 | -5.3716938 | 60 | 40 | 20 | FALSE | FALSE | up |
| Beijing | CSF | N+ vs N- | CCL13 | 0.46415017 | 5.1846608 | 1.3916863 | 0.16952676 | 0.56814374 | -5.3950154 | 60 | 40 | 20 | FALSE | FALSE | up |
| Beijing | CSF | N+ vs N- | IL16 | 0.18634101 | 7.6480456 | 1.3655195 | 0.17755582 | 0.57939267 | -5.4293939 | 60 | 40 | 20 | FALSE | FALSE | up |
| Beijing | CSF | N+ vs N- | CST3 | -0.18889675 | 13.793429 | -1.3337593 | 0.18768802 | 0.58077073 | -5.470302 | 60 | 40 | 20 | FALSE | FALSE | down |
| Beijing | CSF | N+ vs N- | FGF2 | 0.4675012 | 9.8206192 | 1.3205496 | 0.19202903 | 0.58077073 | -5.4870511 | 60 | 40 | 20 | FALSE | FALSE | up |
| Beijing | CSF | N+ vs N- | GDNF | 0.071568849 | 9.3229102 | 1.3363846 | 0.18683422 | 0.58077073 | -5.4669547 | 60 | 40 | 20 | FALSE | FALSE | up |
| Beijing | CSF | N+ vs N- | BACE1 | 0.13813491 | 14.665497 | 1.2919697 | 0.20167982 | 0.59543566 | -5.5227531 | 60 | 40 | 20 | FALSE | FALSE | up |
| Beijing | CSF | N+ vs N- | PTN | -0.25524859 | 13.660102 | -1.2631328 | 0.21178098 | 0.61071723 | -5.5580314 | 60 | 40 | 20 | FALSE | FALSE | down |
| Beijing | CSF | N+ vs N- | VGF | 0.10464417 | 9.6395887 | 1.2151852 | 0.22939954 | 0.64648962 | -5.6150247 | 60 | 40 | 20 | FALSE | FALSE | up |
| Beijing | CSF | N+ vs N- | Abeta42 | -0.37331204 | 16.530256 | -1.1276352 | 0.26428752 | 0.68274277 | -5.713689 | 60 | 40 | 20 | FALSE | FALSE | down |
| Beijing | CSF | N+ vs N- | CCL17 | 0.30165936 | 5.6371718 | 1.1351679 | 0.26114553 | 0.68274277 | -5.7054761 | 60 | 40 | 20 | FALSE | FALSE | up |
| Beijing | CSF | N+ vs N- | NEFL | 0.24264716 | 17.107732 | 1.1370804 | 0.26035203 | 0.68274277 | -5.7033826 | 60 | 40 | 20 | FALSE | FALSE | up |
| Beijing | CSF | N+ vs N- | pSNCA-129 | 0.13175846 | 8.4037929 | 1.1428141 | 0.25798342 | 0.68274277 | -5.6970861 | 60 | 40 | 20 | FALSE | FALSE | up |
| Beijing | CSF | N+ vs N- | IL12p70 | 0.14603788 | 9.1754639 | 1.0664365 | 0.29080752 | 0.73592108 | -5.7784758 | 60 | 40 | 20 | FALSE | FALSE | up |
| Beijing | CSF | N+ vs N- | Abeta38 | 0.19903006 | 20.038683 | 0.8438492 | 0.40234789 | 0.73626429 | -5.9846772 | 60 | 40 | 20 | FALSE | FALSE | up |
| Beijing | CSF | N+ vs N- | AGRN | 0.093395777 | 13.790963 | 0.84118006 | 0.40382858 | 0.73626429 | -5.9868668 | 60 | 40 | 20 | FALSE | FALSE | up |
| Beijing | CSF | N+ vs N- | CALB2 | 0.15235525 | 15.679569 | 0.94944505 | 0.3464783 | 0.73626429 | -5.8926493 | 60 | 40 | 20 | FALSE | FALSE | up |
| Beijing | CSF | N+ vs N- | CCL26 | -0.22493786 | 8.3339029 | -0.93705746 | 0.35275713 | 0.73626429 | -5.9039892 | 60 | 40 | 20 | FALSE | FALSE | down |
| Beijing | CSF | N+ vs N- | CRH | -0.16102551 | 12.948292 | -0.88148099 | 0.38182942 | 0.73626429 | -5.953088 | 60 | 40 | 20 | FALSE | FALSE | down |
| Beijing | CSF | N+ vs N- | CX3CL1 | 0.087790431 | 10.477216 | 0.83776171 | 0.40572978 | 0.73626429 | -5.9896612 | 60 | 40 | 20 | FALSE | FALSE | up |
| Beijing | CSF | N+ vs N- | CXCL10 | -0.2430181 | 11.099771 | -1.0178126 | 0.31314899 | 0.73626429 | -5.8274781 | 60 | 40 | 20 | FALSE | FALSE | down |
| Beijing | CSF | N+ vs N- | GDF15 | -0.74522321 | 6.5810566 | -0.88635126 | 0.37922294 | 0.73626429 | -5.9489018 | 60 | 40 | 20 | FALSE | FALSE | down |
| Beijing | CSF | N+ vs N- | HBA1 | 1.4321577 | 3.582214 | 1.0443145 | 0.30083189 | 0.73626429 | -5.8010422 | 60 | 40 | 20 | FALSE | FALSE | up |
| Beijing | CSF | N+ vs N- | IGFBP7 | -0.061461122 | 13.987251 | -0.90659613 | 0.36850913 | 0.73626429 | -5.9312607 | 60 | 40 | 20 | FALSE | FALSE | down |
| Beijing | CSF | N+ vs N- | IL18 | 0.20154591 | 6.6752287 | 0.96427206 | 0.33905971 | 0.73626429 | -5.8788869 | 60 | 40 | 20 | FALSE | FALSE | up |
| Beijing | CSF | N+ vs N- | IL9 | -0.1494193 | 11.399257 | -0.92056725 | 0.36122941 | 0.73626429 | -5.918861 | 60 | 40 | 20 | FALSE | FALSE | down |
| Beijing | CSF | N+ vs N- | KLK6 | -0.06793257 | 18.683566 | -0.97924109 | 0.33167702 | 0.73626429 | -5.8647837 | 60 | 40 | 20 | FALSE | FALSE | down |
| Beijing | CSF | N+ vs N- | MME | -0.24462061 | 0.47404207 | -1.004424 | 0.3194994 | 0.73626429 | -5.8405846 | 60 | 40 | 20 | FALSE | FALSE | down |
| Beijing | CSF | N+ vs N- | NPTX2 | 0.18006629 | 14.3925 | 0.89279 | 0.37579433 | 0.73626429 | -5.9433331 | 60 | 40 | 20 | FALSE | FALSE | up |
| Beijing | CSF | N+ vs N- | NPTXR | 0.12303275 | 16.90649 | 0.84268587 | 0.40299283 | 0.73626429 | -5.9856324 | 60 | 40 | 20 | FALSE | FALSE | up |
| Beijing | CSF | N+ vs N- | RUVBL2 | 0.14944582 | 9.4233337 | 1.0313191 | 0.30682966 | 0.73626429 | -5.8140869 | 60 | 40 | 20 | FALSE | FALSE | up |
| Beijing | CSF | N+ vs N- | TARDBP | 0.31907457 | 5.4452168 | 0.90233375 | 0.37074861 | 0.73626429 | -5.935007 | 60 | 40 | 20 | FALSE | FALSE | up |
| Beijing | CSF | N+ vs N- | TNF | 0.14987929 | 9.1767678 | 0.86456966 | 0.39096742 | 0.73626429 | -5.9674497 | 60 | 40 | 20 | FALSE | FALSE | up |
| Beijing | CSF | N+ vs N- | YWHAZ | 0.053049427 | 11.001806 | 0.83066291 | 0.40969545 | 0.73626429 | -5.9954287 | 60 | 40 | 20 | FALSE | FALSE | up |
| Beijing | CSF | N+ vs N- | BASP1 | 0.032183266 | 9.9938904 | 0.74250122 | 0.46089032 | 0.80466986 | -6.0630598 | 60 | 40 | 20 | FALSE | FALSE | up |
| Beijing | CSF | N+ vs N- | KDR | -0.66191235 | 5.4327837 | -0.73199453 | 0.46722766 | 0.80466986 | -6.0706249 | 60 | 40 | 20 | FALSE | FALSE | down |
| Beijing | CSF | N+ vs N- | TEK | -0.095916547 | 6.0853717 | -0.74111102 | 0.461726 | 0.80466986 | -6.0640668 | 60 | 40 | 20 | FALSE | FALSE | down |
| Beijing | CSF | N+ vs N- | IL13 | 0.16359718 | 8.8546879 | 0.66782943 | 0.50698852 | 0.83822102 | -6.1145277 | 60 | 40 | 20 | FALSE | FALSE | up |
| Beijing | CSF | N+ vs N- | PGF | -0.0993318 | 14.466913 | -0.68891707 | 0.49372302 | 0.83822102 | -6.1005354 | 60 | 40 | 20 | FALSE | FALSE | down |
| Beijing | CSF | N+ vs N- | VCAM1 | 0.090098612 | 8.8746874 | 0.67686429 | 0.50128156 | 0.83822102 | -6.1085852 | 60 | 40 | 20 | FALSE | FALSE | up |
| Beijing | CSF | N+ vs N- | Abeta40 | 0.14041702 | 17.208252 | 0.50945559 | 0.61243689 | 0.87520031 | -6.2058936 | 60 | 40 | 20 | FALSE | FALSE | up |
| Beijing | CSF | N+ vs N- | ANXA5 | 0.12211795 | 6.6320687 | 0.52012203 | 0.60503093 | 0.87520031 | -6.2005035 | 60 | 40 | 20 | FALSE | FALSE | up |
| Beijing | CSF | N+ vs N- | CCL11 | 0.074834469 | 9.026989 | 0.51848585 | 0.60616428 | 0.87520031 | -6.2013375 | 60 | 40 | 20 | FALSE | FALSE | up |
| Beijing | CSF | N+ vs N- | CNTN2 | -0.096020511 | 18.485956 | -0.57365814 | 0.56849848 | 0.87520031 | -6.1717813 | 60 | 40 | 20 | FALSE | FALSE | down |
| Beijing | CSF | N+ vs N- | CSF2 | 0.10250867 | 12.05746 | 0.57315886 | 0.56883412 | 0.87520031 | -6.172062 | 60 | 40 | 20 | FALSE | FALSE | up |
| Beijing | CSF | N+ vs N- | CXCL1 | -0.078197515 | 10.457518 | -0.59200246 | 0.55623419 | 0.87520031 | -6.1613005 | 60 | 40 | 20 | FALSE | FALSE | down |
| Beijing | CSF | N+ vs N- | IFNG | 0.14791114 | 7.8260158 | 0.51738991 | 0.60692397 | 0.87520031 | -6.2018946 | 60 | 40 | 20 | FALSE | FALSE | up |
| Beijing | CSF | N+ vs N- | NPY | 0.095339314 | 11.430528 | 0.58624744 | 0.56006747 | 0.87520031 | -6.1646237 | 60 | 40 | 20 | FALSE | FALSE | up |
| Beijing | CSF | N+ vs N- | PDGFRB | 0.11770141 | 7.0759348 | 0.49963999 | 0.61928822 | 0.87520031 | -6.2107559 | 60 | 40 | 20 | FALSE | FALSE | up |
| Beijing | CSF | N+ vs N- | REST | 0.10207809 | 7.5395625 | 0.49703839 | 0.6211099 | 0.87520031 | -6.212029 | 60 | 40 | 20 | FALSE | FALSE | up |
| Beijing | CSF | N+ vs N- | SAA1 | -0.17032493 | 0.59637182 | -0.54123649 | 0.59049364 | 0.87520031 | -6.1895077 | 60 | 40 | 20 | FALSE | FALSE | down |
| Beijing | CSF | N+ vs N- | TIMP3 | -0.078629786 | 8.526493 | -0.51732417 | 0.60696956 | 0.87520031 | -6.201928 | 60 | 40 | 20 | FALSE | FALSE | down |
| Beijing | CSF | N+ vs N- | VEGFD | 0.04612636 | 10.639803 | 0.58177018 | 0.56305873 | 0.87520031 | -6.1671868 | 60 | 40 | 20 | FALSE | FALSE | up |
| Beijing | CSF | N+ vs N- | BDNF | 0.27670785 | 1.5611245 | 0.45999092 | 0.64730569 | 0.88710404 | -6.2294425 | 60 | 40 | 20 | FALSE | FALSE | up |
| Beijing | CSF | N+ vs N- | IL15 | 0.03920814 | 13.200736 | 0.47359968 | 0.63762855 | 0.88710404 | -6.2232013 | 60 | 40 | 20 | FALSE | FALSE | up |
| Beijing | CSF | N+ vs N- | IL33 | 0.13372912 | 9.6851193 | 0.4547907 | 0.6510199 | 0.88710404 | -6.2317797 | 60 | 40 | 20 | FALSE | FALSE | up |
| Beijing | CSF | N+ vs N- | GFAP | 0.058160609 | 19.001968 | 0.38898622 | 0.6987631 | 0.94181114 | -6.2590748 | 60 | 40 | 20 | FALSE | FALSE | up |
| Beijing | CSF | N+ vs N- | CCL22 | -0.10399989 | 5.0531413 | -0.37768679 | 0.70709257 | 0.94279009 | -6.2633359 | 60 | 40 | 20 | FALSE | FALSE | down |
| Beijing | CSF | N+ vs N- | CHI3L1 | 0.036065159 | 14.701673 | 0.32640585 | 0.74533539 | 0.96316321 | -6.2811033 | 60 | 40 | 20 | FALSE | FALSE | up |
| Beijing | CSF | N+ vs N- | IGF1R | 0.021948727 | 13.248154 | 0.25707245 | 0.79806607 | 0.96316321 | -6.3010242 | 60 | 40 | 20 | FALSE | FALSE | up |
| Beijing | CSF | N+ vs N- | IL10 | -0.050094394 | 9.5248731 | -0.23433337 | 0.81558175 | 0.96316321 | -6.3065293 | 60 | 40 | 20 | FALSE | FALSE | down |
| Beijing | CSF | N+ vs N- | IL6 | 0.069577226 | 12.316498 | 0.29362416 | 0.77013176 | 0.96316321 | -6.2911104 | 60 | 40 | 20 | FALSE | FALSE | up |
| Beijing | CSF | N+ vs N- | IL7 | -0.059191714 | 10.393783 | -0.33602745 | 0.73810734 | 0.96316321 | -6.2779661 | 60 | 40 | 20 | FALSE | FALSE | down |
| Beijing | CSF | N+ vs N- | NGF | 0.030889787 | 6.4075521 | 0.31814101 | 0.75156266 | 0.96316321 | -6.2837257 | 60 | 40 | 20 | FALSE | FALSE | up |
| Beijing | CSF | N+ vs N- | POSTN | 0.074080139 | 5.6536085 | 0.28545809 | 0.77634753 | 0.96316321 | -6.2934391 | 60 | 40 | 20 | FALSE | FALSE | up |
| Beijing | CSF | N+ vs N- | S100A12 | 0.11227681 | 4.2013375 | 0.23695421 | 0.81355792 | 0.96316321 | -6.3059207 | 60 | 40 | 20 | FALSE | FALSE | up |
| Beijing | CSF | N+ vs N- | SNAP25 | 0.02000395 | 13.164438 | 0.26456861 | 0.79231418 | 0.96316321 | -6.2990981 | 60 | 40 | 20 | FALSE | FALSE | up |
| Beijing | CSF | N+ vs N- | TREM2 | -0.024894566 | 12.054522 | -0.23979908 | 0.81136255 | 0.96316321 | -6.3052525 | 60 | 40 | 20 | FALSE | FALSE | down |
| Beijing | CSF | N+ vs N- | VEGFA | -0.024712997 | 14.637162 | -0.2699819 | 0.78816769 | 0.96316321 | -6.2976727 | 60 | 40 | 20 | FALSE | FALSE | down |
| Beijing | CSF | N+ vs N- | YWHAG | 0.015228753 | 11.917281 | 0.27533605 | 0.78407256 | 0.96316321 | -6.2962347 | 60 | 40 | 20 | FALSE | FALSE | up |
| Beijing | CSF | N+ vs N- | ARSA | -0.024749794 | 6.2430866 | -0.13377793 | 0.89405831 | 0.97620435 | -6.3247736 | 60 | 40 | 20 | FALSE | FALSE | down |
| Beijing | CSF | N+ vs N- | CCL2 | -0.012801765 | 11.617946 | -0.12107961 | 0.90406185 | 0.97620435 | -6.3263693 | 60 | 40 | 20 | FALSE | FALSE | down |
| Beijing | CSF | N+ vs N- | CD40LG | -0.064600344 | 5.4008421 | -0.16310449 | 0.87102398 | 0.97620435 | -6.3204812 | 60 | 40 | 20 | FALSE | FALSE | down |
| Beijing | CSF | N+ vs N- | CD63 | -0.011668549 | 9.7160327 | -0.13758734 | 0.8910606 | 0.97620435 | -6.3242639 | 60 | 40 | 20 | FALSE | FALSE | down |
| Beijing | CSF | N+ vs N- | FOLR1 | -0.014254211 | 12.253085 | -0.18010878 | 0.85771805 | 0.97620435 | -6.3176043 | 60 | 40 | 20 | FALSE | FALSE | down |
| Beijing | CSF | N+ vs N- | IL1B | -0.019313327 | 10.762551 | -0.16598903 | 0.86876406 | 0.97620435 | -6.3200132 | 60 | 40 | 20 | FALSE | FALSE | down |
| Beijing | CSF | N+ vs N- | IL5 | -0.013173928 | 13.233528 | -0.14921477 | 0.88192082 | 0.97620435 | -6.3226198 | 60 | 40 | 20 | FALSE | FALSE | down |
| Beijing | CSF | N+ vs N- | MSLN | 0.017920487 | 8.6360746 | 0.10946754 | 0.91322342 | 0.97620435 | -6.3276893 | 60 | 40 | 20 | FALSE | FALSE | up |
| Beijing | CSF | N+ vs N- | NPTX1 | 0.032065842 | 15.5617 | 0.18083149 | 0.85715341 | 0.97620435 | -6.3174757 | 60 | 40 | 20 | FALSE | FALSE | up |
| Beijing | CSF | N+ vs N- | S100B | 0.013231226 | 15.635169 | 0.10969256 | 0.91304577 | 0.97620435 | -6.327665 | 60 | 40 | 20 | FALSE | FALSE | up |
| Beijing | CSF | N+ vs N- | SFRP1 | 0.087198443 | 15.725117 | 0.17838789 | 0.85906283 | 0.97620435 | -6.3179084 | 60 | 40 | 20 | FALSE | FALSE | up |
| Beijing | CSF | N+ vs N- | ACHE | -0.012141519 | 12.648374 | -0.058957294 | 0.95319624 | 0.9768292 | -6.3318846 | 60 | 40 | 20 | FALSE | FALSE | down |
| Beijing | CSF | N+ vs N- | CCL3 | 0.0093580454 | 10.157944 | 0.073963614 | 0.94130319 | 0.9768292 | -6.3309009 | 60 | 40 | 20 | FALSE | FALSE | up |
| Beijing | CSF | N+ vs N- | CXCL8 | -0.0064984182 | 13.909918 | -0.065383763 | 0.94810156 | 0.9768292 | -6.3314905 | 60 | 40 | 20 | FALSE | FALSE | down |
| Beijing | CSF | N+ vs N- | pTDP43-409 | -0.0033828683 | 9.9081331 | -0.059285064 | 0.95293634 | 0.9768292 | -6.3318655 | 60 | 40 | 20 | FALSE | FALSE | down |
| Beijing | CSF | N+ vs N- | TREM1 | 0.019918939 | 7.9143354 | 0.0945624 | 0.92500033 | 0.9768292 | -6.3291889 | 60 | 40 | 20 | FALSE | FALSE | up |
| Beijing | CSF | N+ vs N- | CCL4 | -0.0092637647 | 9.6808155 | -0.03363362 | 0.97328904 | 0.98120196 | -6.333041 | 60 | 40 | 20 | FALSE | FALSE | down |
| Beijing | CSF | N+ vs N- | FCN2 | -0.016883876 | 1.5792139 | -0.037588216 | 0.97014982 | 0.98120196 | -6.3329021 | 60 | 40 | 20 | FALSE | FALSE | down |
| Beijing | CSF | N+ vs N- | TAFA5 | -0.0033947528 | 20.025517 | -0.021486413 | 0.9829341 | 0.9829341 | -6.3333712 | 60 | 40 | 20 | FALSE | FALSE | down |

**Beijing | CSF | A+T+N+ vs A+T-N-**

| **Cohort** | **Fluid** | **Contrast** | **Protein** | **log₂FC** | **AveExpr** | **t** | **P.Value** | **FDR** | **B** | **N_total** | **N_group1** | **N_group2** | **Pass_FDR** | **Pass_both_thresholds** | **Direction** |
| --- | --- | --- | --- | --- | --- | --- | --- | --- | --- | --- | --- | --- | --- | --- | --- |
| Beijing | CSF | A+T+N+ vs A+T-N- | pTau-181 | 1.2075184 | 18.215873 | 5.3515898 | 3.0240882e-06 | 0.00018749347 | 4.4637067 | 48 | 36 | 12 | TRUE | TRUE | up |
| Beijing | CSF | A+T+N+ vs A+T-N- | pTau-217 | 1.460201 | 17.205887 | 5.4375836 | 2.2685306e-06 | 0.00018749347 | 4.7376126 | 48 | 36 | 12 | TRUE | TRUE | up |
| Beijing | CSF | A+T+N+ vs A+T-N- | pTau-231 | 1.2978823 | 18.509591 | 5.0602238 | 7.9645624e-06 | 0.00032920191 | 3.5419914 | 48 | 36 | 12 | TRUE | TRUE | up |
| Beijing | CSF | A+T+N+ vs A+T-N- | SNCB | 0.46182353 | 12.568067 | 4.5749624 | 3.8928668e-05 | 0.0012067887 | 2.0362672 | 48 | 36 | 12 | TRUE | FALSE | up |
| Beijing | CSF | A+T+N+ vs A+T-N- | FABP3 | 0.56819976 | 12.062005 | 4.225519 | 0.00011870725 | 0.0029439399 | 0.98321834 | 48 | 36 | 12 | TRUE | TRUE | up |
| Beijing | CSF | A+T+N+ vs A+T-N- | MAPT | 0.77308706 | 16.894836 | 4.0905215 | 0.00018119854 | 0.0037447697 | 0.58521237 | 48 | 36 | 12 | TRUE | TRUE | up |
| Beijing | CSF | A+T+N+ vs A+T-N- | VSNL1 | 0.51192198 | 13.63791 | 3.7579108 | 0.00050254637 | 0.00890225 | -0.37051769 | 48 | 36 | 12 | TRUE | TRUE | up |
| Beijing | CSF | A+T+N+ vs A+T-N- | NRGN | 0.42935424 | 9.1905295 | 3.4919194 | 0.0011073228 | 0.017163504 | -1.1055 | 48 | 36 | 12 | TRUE | FALSE | up |
| Beijing | CSF | A+T+N+ vs A+T-N- | MDH1 | 0.54887988 | 11.086176 | 2.9369917 | 0.005264001 | 0.072526235 | -2.5366563 | 48 | 36 | 12 | FALSE | FALSE | up |
| Beijing | CSF | A+T+N+ vs A+T-N- | PSEN1 | 0.28398105 | 11.178628 | 2.8602183 | 0.006460817 | 0.080114131 | -2.7221922 | 48 | 36 | 12 | FALSE | FALSE | up |
| Beijing | CSF | A+T+N+ vs A+T-N- | GOT1 | 0.30808057 | 11.056756 | 2.7312413 | 0.009055707 | 0.090505804 | -3.0263185 | 48 | 36 | 12 | FALSE | FALSE | up |
| Beijing | CSF | A+T+N+ vs A+T-N- | SMOC1 | 0.53794937 | 13.476569 | 2.7445875 | 0.0087481774 | 0.090505804 | -2.9952994 | 48 | 36 | 12 | FALSE | FALSE | up |
| Beijing | CSF | A+T+N+ vs A+T-N- | UCHL1 | 0.26465042 | 12.188307 | 2.7131504 | 0.0094885117 | 0.090505804 | -3.0681955 | 48 | 36 | 12 | FALSE | FALSE | up |
| Beijing | CSF | A+T+N+ vs A+T-N- | SOD1 | 0.4275946 | 13.56548 | 2.4808629 | 0.01701669 | 0.15071926 | -3.5879953 | 48 | 36 | 12 | FALSE | FALSE | up |
| Beijing | CSF | A+T+N+ vs A+T-N- | BACE1 | 0.28135593 | 14.738289 | 2.3543019 | 0.023103204 | 0.19098649 | -3.8565885 | 48 | 36 | 12 | FALSE | FALSE | up |
| Beijing | CSF | A+T+N+ vs A+T-N- | DDC | 0.43685272 | 6.4551109 | 2.2252448 | 0.031257718 | 0.24224731 | -4.1192646 | 48 | 36 | 12 | FALSE | FALSE | up |
| Beijing | CSF | A+T+N+ vs A+T-N- | NPTXR | 0.33753552 | 16.971971 | 2.1858932 | 0.034208614 | 0.24266639 | -4.1970344 | 48 | 36 | 12 | FALSE | FALSE | up |
| Beijing | CSF | A+T+N+ vs A+T-N- | SNCA | 0.72324647 | 6.7596891 | 2.1730237 | 0.035225766 | 0.24266639 | -4.2222281 | 48 | 36 | 12 | FALSE | FALSE | up |
| Beijing | CSF | A+T+N+ vs A+T-N- | NPTX2 | 0.46762288 | 14.481615 | 2.0555157 | 0.04581597 | 0.29900949 | -4.446697 | 48 | 36 | 12 | FALSE | FALSE | up |
| Beijing | CSF | A+T+N+ vs A+T-N- | ENO2 | 0.32559855 | 9.4355011 | 2.0149789 | 0.050065227 | 0.30377457 | -4.5217653 | 48 | 36 | 12 | FALSE | FALSE | up |
| Beijing | CSF | A+T+N+ vs A+T-N- | NPY | 0.38126124 | 11.447445 | 2.002452 | 0.051445694 | 0.30377457 | -4.5447136 | 48 | 36 | 12 | FALSE | FALSE | up |
| Beijing | CSF | A+T+N+ vs A+T-N- | SLIT2 | 0.18089353 | 11.263452 | 1.9682996 | 0.055377854 | 0.31212972 | -4.6066749 | 48 | 36 | 12 | FALSE | FALSE | up |
| Beijing | CSF | A+T+N+ vs A+T-N- | IL4 | -0.24530188 | 9.7433547 | -1.935157 | 0.059438771 | 0.3204525 | -4.6659538 | 48 | 36 | 12 | FALSE | FALSE | down |
| Beijing | CSF | A+T+N+ vs A+T-N- | FLT1 | 0.38081263 | 11.64528 | 1.7748994 | 0.082858322 | 0.40278569 | -4.9405326 | 48 | 36 | 12 | FALSE | FALSE | up |
| Beijing | CSF | A+T+N+ vs A+T-N- | Oligo-SNCA | 0.25784272 | 8.1537343 | 1.7742925 | 0.082959975 | 0.40278569 | -4.9415339 | 48 | 36 | 12 | FALSE | FALSE | up |
| Beijing | CSF | A+T+N+ vs A+T-N- | PDLIM5 | 0.12131349 | 7.4315376 | 1.7654377 | 0.084455063 | 0.40278569 | -4.9561083 | 48 | 36 | 12 | FALSE | FALSE | up |
| Beijing | CSF | A+T+N+ vs A+T-N- | CALB2 | 0.29103852 | 15.757081 | 1.6597216 | 0.10410854 | 0.42325792 | -5.1252048 | 48 | 36 | 12 | FALSE | FALSE | up |
| Beijing | CSF | A+T+N+ vs A+T-N- | PGK1 | 0.27774907 | 3.7458455 | 1.6513471 | 0.10581448 | 0.42325792 | -5.1382094 | 48 | 36 | 12 | FALSE | FALSE | up |
| Beijing | CSF | A+T+N+ vs A+T-N- | PRDX6 | 0.90874594 | 7.5917047 | 1.7131086 | 0.093757569 | 0.42325792 | -5.0409474 | 48 | 36 | 12 | FALSE | FALSE | up |
| Beijing | CSF | A+T+N+ vs A+T-N- | pSNCA-129 | 0.25189039 | 8.4436533 | 1.6641264 | 0.10322036 | 0.42325792 | -5.1183415 | 48 | 36 | 12 | FALSE | FALSE | up |
| Beijing | CSF | A+T+N+ vs A+T-N- | SQSTM1 | 0.23583876 | 10.895583 | 1.6518632 | 0.10570869 | 0.42325792 | -5.1374097 | 48 | 36 | 12 | FALSE | FALSE | up |
| Beijing | CSF | A+T+N+ vs A+T-N- | CX3CL1 | 0.20268363 | 10.528404 | 1.5619635 | 0.12548758 | 0.44894725 | -5.2733828 | 48 | 36 | 12 | FALSE | FALSE | up |
| Beijing | CSF | A+T+N+ vs A+T-N- | FGF2 | 0.7121934 | 9.8388999 | 1.5589463 | 0.1262 | 0.44894725 | -5.2778291 | 48 | 36 | 12 | FALSE | FALSE | up |
| Beijing | CSF | A+T+N+ vs A+T-N- | GDI1 | 0.19198628 | 9.8892172 | 1.5974408 | 0.11735169 | 0.44894725 | -5.2205291 | 48 | 36 | 12 | FALSE | FALSE | up |
| Beijing | CSF | A+T+N+ vs A+T-N- | IL12p70 | 0.24434803 | 9.1468309 | 1.556757 | 0.12671898 | 0.44894725 | -5.2810505 | 48 | 36 | 12 | FALSE | FALSE | up |
| Beijing | CSF | A+T+N+ vs A+T-N- | Abeta38 | 0.35795478 | 20.123538 | 1.4386659 | 0.15734702 | 0.50790452 | -5.4487757 | 48 | 36 | 12 | FALSE | FALSE | up |
| Beijing | CSF | A+T+N+ vs A+T-N- | AGRN | 0.18008213 | 13.847667 | 1.4261144 | 0.16091819 | 0.50790452 | -5.4658993 | 48 | 36 | 12 | FALSE | FALSE | up |
| Beijing | CSF | A+T+N+ vs A+T-N- | BASP1 | 0.077927672 | 9.9848384 | 1.4341921 | 0.15861274 | 0.50790452 | -5.4548948 | 48 | 36 | 12 | FALSE | FALSE | up |
| Beijing | CSF | A+T+N+ vs A+T-N- | CXCL10 | -0.40743966 | 11.096081 | -1.460904 | 0.1511727 | 0.50790452 | -5.418103 | 48 | 36 | 12 | FALSE | FALSE | down |
| Beijing | CSF | A+T+N+ vs A+T-N- | IL15 | 0.14788119 | 13.222645 | 1.4160056 | 0.16384017 | 0.50790452 | -5.4795912 | 48 | 36 | 12 | FALSE | FALSE | up |
| Beijing | CSF | A+T+N+ vs A+T-N- | PARK7 | 0.22784642 | 8.8776117 | 1.3933284 | 0.17054541 | 0.51579587 | -5.5099835 | 48 | 36 | 12 | FALSE | FALSE | up |
| Beijing | CSF | A+T+N+ vs A+T-N- | Abeta40 | 0.439683 | 17.237613 | 1.3676688 | 0.17838678 | 0.52666572 | -5.5438321 | 48 | 36 | 12 | FALSE | FALSE | up |
| Beijing | CSF | A+T+N+ vs A+T-N- | CHIT1 | -2.2438916 | 5.1992229 | -1.3266098 | 0.19150737 | 0.55225382 | -5.5967936 | 48 | 36 | 12 | FALSE | FALSE | down |
| Beijing | CSF | A+T+N+ vs A+T-N- | HBA1 | 2.2155738 | 3.51062 | 1.2916086 | 0.20326138 | 0.56009802 | -5.6407665 | 48 | 36 | 12 | FALSE | FALSE | up |
| Beijing | CSF | A+T+N+ vs A+T-N- | PTN | -0.32611511 | 13.711228 | -1.2961723 | 0.20169867 | 0.56009802 | -5.6350945 | 48 | 36 | 12 | FALSE | FALSE | down |
| Beijing | CSF | A+T+N+ vs A+T-N- | CD63 | 0.11714905 | 9.7415666 | 1.135402 | 0.26237663 | 0.56535928 | -5.8236505 | 48 | 36 | 12 | FALSE | FALSE | up |
| Beijing | CSF | A+T+N+ vs A+T-N- | HTT | 0.39796683 | 7.4493198 | 1.1777907 | 0.24523459 | 0.56535928 | -5.7761993 | 48 | 36 | 12 | FALSE | FALSE | up |
| Beijing | CSF | A+T+N+ vs A+T-N- | ICAM1 | 0.54626042 | 5.2362811 | 1.1702827 | 0.24821014 | 0.56535928 | -5.7847229 | 48 | 36 | 12 | FALSE | FALSE | up |
| Beijing | CSF | A+T+N+ vs A+T-N- | IGF1R | 0.11592571 | 13.286578 | 1.1677323 | 0.24922686 | 0.56535928 | -5.7876068 | 48 | 36 | 12 | FALSE | FALSE | up |
| Beijing | CSF | A+T+N+ vs A+T-N- | IL17A | 0.082156845 | 8.6663454 | 1.2252207 | 0.22703446 | 0.56535928 | -5.7211755 | 48 | 36 | 12 | FALSE | FALSE | up |
| Beijing | CSF | A+T+N+ vs A+T-N- | IL2 | 0.25508258 | 8.0820327 | 1.1491589 | 0.25672184 | 0.56535928 | -5.8084298 | 48 | 36 | 12 | FALSE | FALSE | up |
| Beijing | CSF | A+T+N+ vs A+T-N- | IL6R | -0.30097132 | 6.823957 | -1.121485 | 0.2681872 | 0.56535928 | -5.8388727 | 48 | 36 | 12 | FALSE | FALSE | down |
| Beijing | CSF | A+T+N+ vs A+T-N- | NEFH | 0.2920395 | 21.199904 | 1.2717436 | 0.21016985 | 0.56535928 | -5.6652396 | 48 | 36 | 12 | FALSE | FALSE | up |
| Beijing | CSF | A+T+N+ vs A+T-N- | PDGFRB | 0.32565134 | 7.1368711 | 1.1351108 | 0.26249727 | 0.56535928 | -5.8239708 | 48 | 36 | 12 | FALSE | FALSE | up |
| Beijing | CSF | A+T+N+ vs A+T-N- | PGF | -0.21961241 | 14.461618 | -1.1719851 | 0.24753319 | 0.56535928 | -5.7827948 | 48 | 36 | 12 | FALSE | FALSE | down |
| Beijing | CSF | A+T+N+ vs A+T-N- | TAFA5 | 0.1762227 | 20.098989 | 1.1243523 | 0.26698264 | 0.56535928 | -5.835751 | 48 | 36 | 12 | FALSE | FALSE | up |
| Beijing | CSF | A+T+N+ vs A+T-N- | TIMP3 | 0.20294517 | 8.5937153 | 1.1272653 | 0.26576282 | 0.56535928 | -5.8325718 | 48 | 36 | 12 | FALSE | FALSE | up |
| Beijing | CSF | A+T+N+ vs A+T-N- | TREM2 | 0.12749933 | 12.054985 | 1.1195517 | 0.26900159 | 0.56535928 | -5.8409734 | 48 | 36 | 12 | FALSE | FALSE | up |
| Beijing | CSF | A+T+N+ vs A+T-N- | VEGFD | 0.13239461 | 10.640535 | 1.258164 | 0.21499267 | 0.56535928 | -5.6817669 | 48 | 36 | 12 | FALSE | FALSE | up |
| Beijing | CSF | A+T+N+ vs A+T-N- | RUVBL2 | 0.21617033 | 9.4390576 | 1.0950123 | 0.27949112 | 0.57761498 | -5.8673391 | 48 | 36 | 12 | FALSE | FALSE | up |
| Beijing | CSF | A+T+N+ vs A+T-N- | IL18 | 0.26882371 | 6.683338 | 1.0318408 | 0.307806 | 0.62570399 | -5.9326644 | 48 | 36 | 12 | FALSE | FALSE | up |
| Beijing | CSF | A+T+N+ vs A+T-N- | IL16 | 0.18465237 | 7.6883368 | 0.99438449 | 0.325493 | 0.650986 | -5.9696536 | 48 | 36 | 12 | FALSE | FALSE | up |
| Beijing | CSF | A+T+N+ vs A+T-N- | SFRP1 | 0.55697468 | 15.774763 | 0.96590448 | 0.3393906 | 0.66099865 | -5.9969043 | 48 | 36 | 12 | FALSE | FALSE | up |
| Beijing | CSF | A+T+N+ vs A+T-N- | TARDBP | 0.45741541 | 5.5029341 | 0.96233341 | 0.34116059 | 0.66099865 | -6.0002677 | 48 | 36 | 12 | FALSE | FALSE | up |
| Beijing | CSF | A+T+N+ vs A+T-N- | SAA1 | -0.34744717 | 0.58673615 | -0.93953256 | 0.35260565 | 0.67266308 | -6.0214616 | 48 | 36 | 12 | FALSE | FALSE | down |
| Beijing | CSF | A+T+N+ vs A+T-N- | SNAP25 | 0.084359581 | 13.203434 | 0.90149387 | 0.37225219 | 0.6993829 | -6.0557327 | 48 | 36 | 12 | FALSE | FALSE | up |
| Beijing | CSF | A+T+N+ vs A+T-N- | CCL22 | -0.31720577 | 5.0509361 | -0.88227867 | 0.38243854 | 0.7077967 | -6.0725264 | 48 | 36 | 12 | FALSE | FALSE | down |
| Beijing | CSF | A+T+N+ vs A+T-N- | SFTPD | -0.31800562 | 8.0124041 | -0.85988368 | 0.39453139 | 0.71943959 | -6.0916587 | 48 | 36 | 12 | FALSE | FALSE | down |
| Beijing | CSF | A+T+N+ vs A+T-N- | ACHE | 0.19621242 | 12.704604 | 0.84552123 | 0.40241138 | 0.72317408 | -6.1036786 | 48 | 36 | 12 | FALSE | FALSE | up |
| Beijing | CSF | A+T+N+ vs A+T-N- | NPTX1 | 0.14260001 | 15.665552 | 0.81555484 | 0.419164 | 0.74251909 | -6.1281263 | 48 | 36 | 12 | FALSE | FALSE | up |
| Beijing | CSF | A+T+N+ vs A+T-N- | ANXA5 | 0.24132315 | 6.6579196 | 0.79979266 | 0.42814369 | 0.74774392 | -6.1406426 | 48 | 36 | 12 | FALSE | FALSE | up |
| Beijing | CSF | A+T+N+ vs A+T-N- | IGFBP7 | -0.069708401 | 13.993427 | -0.77558733 | 0.44215677 | 0.75106081 | -6.1594014 | 48 | 36 | 12 | FALSE | FALSE | down |
| Beijing | CSF | A+T+N+ vs A+T-N- | UBB | 0.048506954 | 11.886397 | 0.77768643 | 0.44093089 | 0.75106081 | -6.1577968 | 48 | 36 | 12 | FALSE | FALSE | up |
| Beijing | CSF | A+T+N+ vs A+T-N- | IL33 | 0.29901398 | 9.7068814 | 0.75782734 | 0.45260907 | 0.75842601 | -6.1728086 | 48 | 36 | 12 | FALSE | FALSE | up |
| Beijing | CSF | A+T+N+ vs A+T-N- | NGF | 0.092869294 | 6.3860388 | 0.74354501 | 0.46111846 | 0.76238252 | -6.1833712 | 48 | 36 | 12 | FALSE | FALSE | up |
| Beijing | CSF | A+T+N+ vs A+T-N- | GDF15 | -0.6364478 | 6.8851658 | -0.68937457 | 0.49422061 | 0.80635994 | -6.2216503 | 48 | 36 | 12 | FALSE | FALSE | down |
| Beijing | CSF | A+T+N+ vs A+T-N- | MSLN | 0.13084835 | 8.6989442 | 0.66976393 | 0.506521 | 0.81569615 | -6.2348103 | 48 | 36 | 12 | FALSE | FALSE | up |
| Beijing | CSF | A+T+N+ vs A+T-N- | VCAM1 | 0.10290121 | 8.9034183 | 0.62762549 | 0.53350514 | 0.84813638 | -6.2618285 | 48 | 36 | 12 | FALSE | FALSE | up |
| Beijing | CSF | A+T+N+ vs A+T-N- | CCL4 | -0.19375179 | 9.6236661 | -0.60264304 | 0.54985115 | 0.8630575 | -6.2770331 | 48 | 36 | 12 | FALSE | FALSE | down |
| Beijing | CSF | A+T+N+ vs A+T-N- | Abeta42 | -0.23593571 | 16.558428 | -0.56054306 | 0.57796286 | 0.8664743 | -6.3012808 | 48 | 36 | 12 | FALSE | FALSE | down |
| Beijing | CSF | A+T+N+ vs A+T-N- | CCL17 | 0.20035997 | 5.6668187 | 0.5624597 | 0.57666798 | 0.8664743 | -6.3002144 | 48 | 36 | 12 | FALSE | FALSE | up |
| Beijing | CSF | A+T+N+ vs A+T-N- | GDNF | 0.036962798 | 9.3241319 | 0.54727336 | 0.58696646 | 0.8664743 | -6.3085651 | 48 | 36 | 12 | FALSE | FALSE | up |
| Beijing | CSF | A+T+N+ vs A+T-N- | IL13 | -0.16163552 | 9.0102707 | -0.58288389 | 0.56295803 | 0.8664743 | -6.2886286 | 48 | 36 | 12 | FALSE | FALSE | down |
| Beijing | CSF | A+T+N+ vs A+T-N- | NEFL | 0.11204653 | 17.105107 | 0.55646018 | 0.58072596 | 0.8664743 | -6.3035404 | 48 | 36 | 12 | FALSE | FALSE | up |
| Beijing | CSF | A+T+N+ vs A+T-N- | IL9 | -0.094669861 | 11.446234 | -0.52131583 | 0.60477058 | 0.88225356 | -6.3223165 | 48 | 36 | 12 | FALSE | FALSE | down |
| Beijing | CSF | A+T+N+ vs A+T-N- | CNTN2 | 0.083348293 | 18.603381 | 0.47037785 | 0.64041701 | 0.91277826 | -6.3473827 | 48 | 36 | 12 | FALSE | FALSE | up |
| Beijing | CSF | A+T+N+ vs A+T-N- | TNF | 0.10449586 | 9.1752238 | 0.47421815 | 0.637698 | 0.91277826 | -6.3455817 | 48 | 36 | 12 | FALSE | FALSE | up |
| Beijing | CSF | A+T+N+ vs A+T-N- | ARSA | 0.078416133 | 6.2257164 | 0.32356265 | 0.74780774 | 0.92193493 | -6.4053351 | 48 | 36 | 12 | FALSE | FALSE | up |
| Beijing | CSF | A+T+N+ vs A+T-N- | CCL11 | 0.068788764 | 8.9978053 | 0.36422727 | 0.7174393 | 0.92193493 | -6.3914153 | 48 | 36 | 12 | FALSE | FALSE | up |
| Beijing | CSF | A+T+N+ vs A+T-N- | CCL13 | 0.16651395 | 5.135022 | 0.38241141 | 0.70400402 | 0.92193493 | -6.3846615 | 48 | 36 | 12 | FALSE | FALSE | up |
| Beijing | CSF | A+T+N+ vs A+T-N- | CCL2 | -0.040348872 | 11.634328 | -0.31418861 | 0.75486818 | 0.92193493 | -6.4083117 | 48 | 36 | 12 | FALSE | FALSE | down |
| Beijing | CSF | A+T+N+ vs A+T-N- | CCL26 | -0.12180407 | 8.3083813 | -0.41803799 | 0.67796006 | 0.92193493 | -6.3704825 | 48 | 36 | 12 | FALSE | FALSE | down |
| Beijing | CSF | A+T+N+ vs A+T-N- | CHI3L1 | -0.052142251 | 14.729985 | -0.36172258 | 0.7192971 | 0.92193493 | -6.39232 | 48 | 36 | 12 | FALSE | FALSE | down |
| Beijing | CSF | A+T+N+ vs A+T-N- | CRH | 0.09082709 | 12.993722 | 0.41390302 | 0.68096324 | 0.92193493 | -6.3721924 | 48 | 36 | 12 | FALSE | FALSE | up |
| Beijing | CSF | A+T+N+ vs A+T-N- | CST3 | 0.012140502 | 13.858078 | 0.29934187 | 0.76609386 | 0.92193493 | -6.4128477 | 48 | 36 | 12 | FALSE | FALSE | up |
| Beijing | CSF | A+T+N+ vs A+T-N- | IFNG | 0.086406872 | 7.8242037 | 0.26070522 | 0.79554063 | 0.92193493 | -6.4236269 | 48 | 36 | 12 | FALSE | FALSE | up |
| Beijing | CSF | A+T+N+ vs A+T-N- | IL10 | -0.077294081 | 9.5280139 | -0.27094802 | 0.78770275 | 0.92193493 | -6.4209137 | 48 | 36 | 12 | FALSE | FALSE | down |
| Beijing | CSF | A+T+N+ vs A+T-N- | IL1B | 0.063339487 | 10.762509 | 0.4057774 | 0.68688 | 0.92193493 | -6.3755034 | 48 | 36 | 12 | FALSE | FALSE | up |
| Beijing | CSF | A+T+N+ vs A+T-N- | IL5 | 0.030586493 | 13.248817 | 0.26533397 | 0.79199597 | 0.92193493 | -6.4224137 | 48 | 36 | 12 | FALSE | FALSE | up |
| Beijing | CSF | A+T+N+ vs A+T-N- | KLK6 | 0.025739439 | 18.722235 | 0.3340873 | 0.7399067 | 0.92193493 | -6.4018895 | 48 | 36 | 12 | FALSE | FALSE | up |
| Beijing | CSF | A+T+N+ vs A+T-N- | MME | -0.1175597 | 0.41935196 | -0.39441999 | 0.69518333 | 0.92193493 | -6.3800222 | 48 | 36 | 12 | FALSE | FALSE | down |
| Beijing | CSF | A+T+N+ vs A+T-N- | pTDP43-409 | 0.029221744 | 9.9102005 | 0.38822745 | 0.69972671 | 0.92193493 | -6.3824323 | 48 | 36 | 12 | FALSE | FALSE | up |
| Beijing | CSF | A+T+N+ vs A+T-N- | S100B | 0.050186183 | 15.683473 | 0.31730479 | 0.75251871 | 0.92193493 | -6.4073319 | 48 | 36 | 12 | FALSE | FALSE | up |
| Beijing | CSF | A+T+N+ vs A+T-N- | TREM1 | 0.068090293 | 7.8478477 | 0.26239616 | 0.79424521 | 0.92193493 | -6.4231862 | 48 | 36 | 12 | FALSE | FALSE | up |
| Beijing | CSF | A+T+N+ vs A+T-N- | VEGFA | 0.035765032 | 14.688986 | 0.35393103 | 0.72508726 | 0.92193493 | -6.3950946 | 48 | 36 | 12 | FALSE | FALSE | up |
| Beijing | CSF | A+T+N+ vs A+T-N- | YWHAG | 0.020662977 | 11.928375 | 0.28364056 | 0.77802117 | 0.92193493 | -6.4174069 | 48 | 36 | 12 | FALSE | FALSE | up |
| Beijing | CSF | A+T+N+ vs A+T-N- | YWHAZ | 0.029285268 | 11.002938 | 0.37585954 | 0.70883411 | 0.92193493 | -6.3871326 | 48 | 36 | 12 | FALSE | FALSE | up |
| Beijing | CSF | A+T+N+ vs A+T-N- | CD40LG | -0.12072493 | 5.4660301 | -0.23747157 | 0.81339733 | 0.92426666 | -6.4293951 | 48 | 36 | 12 | FALSE | FALSE | down |
| Beijing | CSF | A+T+N+ vs A+T-N- | CXCL8 | -0.029220016 | 13.92458 | -0.2411069 | 0.81059643 | 0.92426666 | -6.4285279 | 48 | 36 | 12 | FALSE | FALSE | down |
| Beijing | CSF | A+T+N+ vs A+T-N- | IL6 | 0.067280586 | 12.24345 | 0.22902583 | 0.81991397 | 0.92426666 | -6.4313589 | 48 | 36 | 12 | FALSE | FALSE | up |
| Beijing | CSF | A+T+N+ vs A+T-N- | FOLR1 | -0.020968206 | 12.262039 | -0.20891986 | 0.83547857 | 0.93332741 | -6.4357484 | 48 | 36 | 12 | FALSE | FALSE | down |
| Beijing | CSF | A+T+N+ vs A+T-N- | IL7 | -0.041487264 | 10.349176 | -0.18236206 | 0.85613931 | 0.93948031 | -6.4409299 | 48 | 36 | 12 | FALSE | FALSE | down |
| Beijing | CSF | A+T+N+ vs A+T-N- | VGF | 0.020445643 | 9.6412838 | 0.1855958 | 0.85361784 | 0.93948031 | -6.4403365 | 48 | 36 | 12 | FALSE | FALSE | up |
| Beijing | CSF | A+T+N+ vs A+T-N- | BDNF | 0.10558133 | 1.7252544 | 0.13388148 | 0.89410904 | 0.95995726 | -6.4485769 | 48 | 36 | 12 | FALSE | FALSE | up |
| Beijing | CSF | A+T+N+ vs A+T-N- | CSF2 | 0.025489417 | 12.030372 | 0.12108224 | 0.90417919 | 0.95995726 | -6.450205 | 48 | 36 | 12 | FALSE | FALSE | up |
| Beijing | CSF | A+T+N+ vs A+T-N- | CXCL1 | -0.019675997 | 10.47559 | -0.11510094 | 0.90889072 | 0.95995726 | -6.4509098 | 48 | 36 | 12 | FALSE | FALSE | down |
| Beijing | CSF | A+T+N+ vs A+T-N- | GFAP | 0.017261778 | 19.041699 | 0.088468177 | 0.9299075 | 0.95995726 | -6.4536149 | 48 | 36 | 12 | FALSE | FALSE | up |
| Beijing | CSF | A+T+N+ vs A+T-N- | KDR | -0.11959534 | 5.3702855 | -0.10180771 | 0.91937356 | 0.95995726 | -6.4523484 | 48 | 36 | 12 | FALSE | FALSE | down |
| Beijing | CSF | A+T+N+ vs A+T-N- | POSTN | -0.02508193 | 5.6127842 | -0.079834168 | 0.93673249 | 0.95995726 | -6.45434 | 48 | 36 | 12 | FALSE | FALSE | down |
| Beijing | CSF | A+T+N+ vs A+T-N- | S100A12 | 0.070291128 | 4.1138976 | 0.11721509 | 0.907225 | 0.95995726 | -6.4506647 | 48 | 36 | 12 | FALSE | FALSE | up |
| Beijing | CSF | A+T+N+ vs A+T-N- | TEK | 0.01217604 | 6.1457445 | 0.091805069 | 0.92727116 | 0.95995726 | -6.4533147 | 48 | 36 | 12 | FALSE | FALSE | up |
| Beijing | CSF | A+T+N+ vs A+T-N- | REST | 0.018219202 | 7.5219474 | 0.067938747 | 0.94614333 | 0.96165388 | -6.4552171 | 48 | 36 | 12 | FALSE | FALSE | up |
| Beijing | CSF | A+T+N+ vs A+T-N- | FCN2 | -0.023195608 | 1.7487597 | -0.039215745 | 0.96889643 | 0.97677364 | -6.4567531 | 48 | 36 | 12 | FALSE | FALSE | down |
| Beijing | CSF | A+T+N+ vs A+T-N- | CCL3 | -0.003737835 | 10.117533 | -0.024636774 | 0.98045649 | 0.98045649 | -6.4572176 | 48 | 36 | 12 | FALSE | FALSE | down |

**ADNI | CSF | T+ vs T-**

| **Cohort** | **Fluid** | **Contrast** | **Protein** | **log₂FC** | **AveExpr** | **t** | **P.Value** | **FDR** | **B** | **N_total** | **N_group1** | **N_group2** | **Pass_FDR** | **Pass_both_thresholds** | **Direction** |
| --- | --- | --- | --- | --- | --- | --- | --- | --- | --- | --- | --- | --- | --- | --- | --- |
| ADNI | CSF | T+ vs T- | MAPT | 1.0791669 | 15.421986 | 18.233844 | 6.9196652e-63 | 8.5803848e-61 | 132.30105 | 865 | 585 | 280 | TRUE | TRUE | up |
| ADNI | CSF | T+ vs T- | pTau-181 | 1.2750754 | 17.463133 | 17.450264 | 1.8306921e-58 | 1.1350291e-56 | 122.16683 | 865 | 585 | 280 | TRUE | TRUE | up |
| ADNI | CSF | T+ vs T- | pTau-231 | 1.294179 | 17.931362 | 16.838131 | 4.5138335e-55 | 1.8657179e-53 | 114.39574 | 865 | 585 | 280 | TRUE | TRUE | up |
| ADNI | CSF | T+ vs T- | pTau-217 | 1.3941459 | 15.999841 | 16.607207 | 8.294615e-54 | 2.5713306e-52 | 111.49966 | 865 | 585 | 280 | TRUE | TRUE | up |
| ADNI | CSF | T+ vs T- | VSNL1 | 0.60097716 | 12.958872 | 13.500815 | 9.4268818e-38 | 2.3378667e-36 | 74.746845 | 865 | 585 | 280 | TRUE | TRUE | up |
| ADNI | CSF | T+ vs T- | SMOC1 | 0.82749358 | 11.946752 | 13.40578 | 2.7170666e-37 | 5.6152711e-36 | 73.695458 | 865 | 585 | 280 | TRUE | TRUE | up |
| ADNI | CSF | T+ vs T- | FABP3 | 0.58270671 | 11.870522 | 13.214526 | 2.2541098e-36 | 3.9929944e-35 | 71.594257 | 865 | 585 | 280 | TRUE | TRUE | up |
| ADNI | CSF | T+ vs T- | NRGN | 0.59645913 | 7.443744 | 12.864098 | 1.0334747e-34 | 1.6018858e-33 | 67.796094 | 865 | 585 | 280 | TRUE | TRUE | up |
| ADNI | CSF | T+ vs T- | BACE1 | 0.40312846 | 14.800397 | 11.88878 | 3.0149698e-30 | 4.1539583e-29 | 57.594151 | 865 | 585 | 280 | TRUE | FALSE | up |
| ADNI | CSF | T+ vs T- | MDH1 | 0.73870623 | 10.808306 | 11.860206 | 4.04045e-30 | 5.010158e-29 | 57.303784 | 865 | 585 | 280 | TRUE | TRUE | up |
| ADNI | CSF | T+ vs T- | SOD1 | 0.4649039 | 13.817817 | 11.555316 | 8.9060109e-29 | 1.0039503e-27 | 54.236746 | 865 | 585 | 280 | TRUE | FALSE | up |
| ADNI | CSF | T+ vs T- | ENO2 | 0.4627044 | 10.798748 | 11.515491 | 1.3283219e-28 | 1.3725993e-27 | 53.8404 | 865 | 585 | 280 | TRUE | FALSE | up |
| ADNI | CSF | T+ vs T- | GOT1 | 0.37487376 | 10.956185 | 11.313641 | 9.9239146e-28 | 9.4658878e-27 | 51.846915 | 865 | 585 | 280 | TRUE | FALSE | up |
| ADNI | CSF | T+ vs T- | GDI1 | 0.36252358 | 8.8736365 | 11.19713 | 3.1313912e-27 | 2.773518e-26 | 50.708054 | 865 | 585 | 280 | TRUE | FALSE | up |
| ADNI | CSF | T+ vs T- | IGF1R | 0.27081504 | 13.081985 | 10.93487 | 4.0298218e-26 | 3.3313193e-25 | 48.17666 | 865 | 585 | 280 | TRUE | FALSE | up |
| ADNI | CSF | T+ vs T- | SNCB | 0.26625639 | 11.166338 | 10.649397 | 6.1795925e-25 | 4.7891842e-24 | 45.472636 | 865 | 585 | 280 | TRUE | FALSE | up |
| ADNI | CSF | T+ vs T- | AGRN | 0.40979759 | 14.214683 | 10.35887 | 9.4083212e-24 | 6.8625402e-23 | 42.776921 | 865 | 585 | 280 | TRUE | FALSE | up |
| ADNI | CSF | T+ vs T- | TAFA5 | 0.58269627 | 19.390467 | 9.897812 | 6.2951914e-22 | 4.3366874e-21 | 38.618212 | 865 | 585 | 280 | TRUE | TRUE | up |
| ADNI | CSF | T+ vs T- | SQSTM1 | 0.41269311 | 10.451519 | 9.4904354 | 2.2819805e-20 | 1.4892925e-19 | 35.068861 | 865 | 585 | 280 | TRUE | FALSE | up |
| ADNI | CSF | T+ vs T- | YWHAG | 0.15631362 | 11.519674 | 9.3947275 | 5.214257e-20 | 3.2328394e-19 | 34.252396 | 865 | 585 | 280 | TRUE | FALSE | up |
| ADNI | CSF | T+ vs T- | CHI3L1 | 0.36501958 | 15.108736 | 9.2331329 | 2.0729982e-19 | 1.2240561e-18 | 32.889119 | 865 | 585 | 280 | TRUE | FALSE | up |
| ADNI | CSF | T+ vs T- | KLK6 | 0.24765911 | 18.923171 | 9.186547 | 3.0751621e-19 | 1.7332732e-18 | 32.499683 | 865 | 585 | 280 | TRUE | FALSE | up |
| ADNI | CSF | T+ vs T- | NPTXR | 0.41154878 | 16.940387 | 9.0991232 | 6.4181506e-19 | 3.4602203e-18 | 31.773217 | 865 | 585 | 280 | TRUE | FALSE | up |
| ADNI | CSF | T+ vs T- | SLIT2 | 0.2492539 | 10.214377 | 9.0738204 | 7.9330257e-19 | 4.09873e-18 | 31.564022 | 865 | 585 | 280 | TRUE | FALSE | up |
| ADNI | CSF | T+ vs T- | ACHE | 0.48156153 | 12.949521 | 9.0090727 | 1.361442e-18 | 6.7527524e-18 | 31.030891 | 865 | 585 | 280 | TRUE | FALSE | up |
| ADNI | CSF | T+ vs T- | TIMP3 | 0.40643704 | 7.0810649 | 8.6900902 | 1.8612539e-17 | 8.8767495e-17 | 28.450608 | 865 | 585 | 280 | TRUE | FALSE | up |
| ADNI | CSF | T+ vs T- | UCHL1 | 0.10784029 | 11.741851 | 8.6258057 | 3.1238534e-17 | 1.4346586e-16 | 27.939991 | 865 | 585 | 280 | TRUE | FALSE | up |
| ADNI | CSF | T+ vs T- | NPTX1 | 0.36344124 | 14.78841 | 8.5986814 | 3.8830429e-17 | 1.7196333e-16 | 27.725493 | 865 | 585 | 280 | TRUE | FALSE | up |
| ADNI | CSF | T+ vs T- | CALB2 | 0.3668552 | 14.003327 | 8.4246623 | 1.5472138e-16 | 6.6156727e-16 | 26.362876 | 865 | 585 | 280 | TRUE | FALSE | up |
| ADNI | CSF | T+ vs T- | IL15 | 0.24529194 | 13.178702 | 8.2561416 | 5.7725171e-16 | 2.3859738e-15 | 25.065771 | 865 | 585 | 280 | TRUE | FALSE | up |
| ADNI | CSF | T+ vs T- | FLT1 | 0.3348988 | 11.585478 | 8.1628888 | 1.1849377e-15 | 4.7397506e-15 | 24.357582 | 865 | 585 | 280 | TRUE | FALSE | up |
| ADNI | CSF | T+ vs T- | PSEN1 | 0.23640156 | 11.312148 | 8.0601444 | 2.5966888e-15 | 1.0062169e-14 | 23.585267 | 865 | 585 | 280 | TRUE | FALSE | up |
| ADNI | CSF | T+ vs T- | VEGFA | 0.26164867 | 14.24775 | 8.0010361 | 4.062712e-15 | 1.5265948e-14 | 23.144756 | 865 | 585 | 280 | TRUE | FALSE | up |
| ADNI | CSF | T+ vs T- | CX3CL1 | 0.27512527 | 10.495984 | 7.9257691 | 7.1554106e-15 | 2.6096203e-14 | 22.587855 | 865 | 585 | 280 | TRUE | FALSE | up |
| ADNI | CSF | T+ vs T- | Abeta38 | 0.71331975 | 17.392873 | 7.8243543 | 1.5233154e-14 | 5.3968887e-14 | 21.844663 | 865 | 585 | 280 | TRUE | TRUE | up |
| ADNI | CSF | T+ vs T- | GFAP | 0.50363972 | 17.178517 | 7.7430999 | 2.7743674e-14 | 9.5561543e-14 | 21.255183 | 865 | 585 | 280 | TRUE | TRUE | up |
| ADNI | CSF | T+ vs T- | SNAP25 | 0.12339081 | 12.219968 | 7.2979273 | 6.7461253e-13 | 2.2608636e-12 | 18.120953 | 865 | 585 | 280 | TRUE | FALSE | up |
| ADNI | CSF | T+ vs T- | NPTX2 | 0.35623501 | 14.412663 | 6.9571246 | 6.9651147e-12 | 2.2728269e-11 | 15.832216 | 865 | 585 | 280 | TRUE | FALSE | up |
| ADNI | CSF | T+ vs T- | CD63 | 0.160117 | 9.8053129 | 6.813782 | 1.8071305e-11 | 5.7457482e-11 | 14.898692 | 865 | 585 | 280 | TRUE | FALSE | up |
| ADNI | CSF | T+ vs T- | CNTN2 | 0.33921253 | 19.147286 | 6.5886829 | 7.8031307e-11 | 2.4189705e-10 | 13.467934 | 865 | 585 | 280 | TRUE | FALSE | up |
| ADNI | CSF | T+ vs T- | NEFL | 0.34091129 | 15.968484 | 6.5503791 | 9.9664932e-11 | 3.0142565e-10 | 13.228781 | 865 | 585 | 280 | TRUE | FALSE | up |
| ADNI | CSF | T+ vs T- | VCAM1 | 0.28869793 | 8.8470681 | 6.3923538 | 2.6997716e-10 | 7.9707543e-10 | 12.255478 | 865 | 585 | 280 | TRUE | FALSE | up |
| ADNI | CSF | T+ vs T- | TREM2 | 0.36266531 | 11.304917 | 6.381619 | 2.8866585e-10 | 8.3243176e-10 | 12.190142 | 865 | 585 | 280 | TRUE | FALSE | up |
| ADNI | CSF | T+ vs T- | CRH | 0.28933536 | 10.340378 | 6.3673249 | 3.1552683e-10 | 8.8921199e-10 | 12.103298 | 865 | 585 | 280 | TRUE | FALSE | up |
| ADNI | CSF | T+ vs T- | Abeta40 | 0.64665174 | 14.923961 | 6.3526054 | 3.457395e-10 | 9.527044e-10 | 12.014053 | 865 | 585 | 280 | TRUE | TRUE | up |
| ADNI | CSF | T+ vs T- | IL9 | 0.28625232 | 11.430261 | 6.2334267 | 7.2004156e-10 | 1.9409816e-09 | 11.298399 | 865 | 585 | 280 | TRUE | FALSE | up |
| ADNI | CSF | T+ vs T- | IL5 | 0.27871521 | 13.069152 | 6.0600575 | 2.048744e-09 | 5.4051968e-09 | 10.279435 | 865 | 585 | 280 | TRUE | FALSE | up |
| ADNI | CSF | T+ vs T- | NPY | 0.38617693 | 9.7785854 | 5.928196 | 4.4610032e-09 | 1.1524258e-08 | 9.5220762 | 865 | 585 | 280 | TRUE | FALSE | up |
| ADNI | CSF | T+ vs T- | SNCA | 0.38637491 | 5.649085 | 5.8578093 | 6.7170042e-09 | 1.6998133e-08 | 9.1240771 | 865 | 585 | 280 | TRUE | FALSE | up |
| ADNI | CSF | T+ vs T- | SFRP1 | 0.5200456 | 13.945303 | 5.8065626 | 9.0244743e-09 | 2.2380696e-08 | 8.8370619 | 865 | 585 | 280 | TRUE | TRUE | up |
| ADNI | CSF | T+ vs T- | MSLN | 0.32696299 | 9.011218 | 5.7728681 | 1.0944815e-08 | 2.6610923e-08 | 8.6496185 | 865 | 585 | 280 | TRUE | FALSE | up |
| ADNI | CSF | T+ vs T- | ANXA5 | 0.41553758 | 6.5454661 | 5.7144717 | 1.5255005e-08 | 3.6377319e-08 | 8.327147 | 865 | 585 | 280 | TRUE | FALSE | up |
| ADNI | CSF | T+ vs T- | S100B | 0.23525148 | 15.569714 | 5.565858 | 3.5044686e-08 | 8.1991342e-08 | 7.5201918 | 865 | 585 | 280 | TRUE | FALSE | up |
| ADNI | CSF | T+ vs T- | TEK | 0.22357494 | 6.014131 | 5.4353147 | 7.1621664e-08 | 1.6446456e-07 | 6.8276657 | 865 | 585 | 280 | TRUE | FALSE | up |
| ADNI | CSF | T+ vs T- | TREM1 | 0.2669106 | 7.421262 | 5.1192176 | 3.8013685e-07 | 8.5703581e-07 | 5.2144522 | 865 | 585 | 280 | TRUE | FALSE | up |
| ADNI | CSF | T+ vs T- | PTN | 0.28005916 | 10.258457 | 5.0036389 | 6.8467946e-07 | 1.5160759e-06 | 4.6472489 | 865 | 585 | 280 | TRUE | FALSE | up |
| ADNI | CSF | T+ vs T- | IL12p70 | 0.21509635 | 9.0801764 | 4.3846241 | 1.3085091e-05 | 2.8465812e-05 | 1.8184911 | 865 | 585 | 280 | TRUE | FALSE | up |
| ADNI | CSF | T+ vs T- | CCL3 | 0.19233496 | 9.3597907 | 4.3546948 | 1.4961513e-05 | 3.1986682e-05 | 1.690727 | 865 | 585 | 280 | TRUE | FALSE | up |
| ADNI | CSF | T+ vs T- | PGK1 | 0.21205718 | 4.6726958 | 4.329801 | 1.6715449e-05 | 3.5130775e-05 | 1.5850957 | 865 | 585 | 280 | TRUE | FALSE | up |
| ADNI | CSF | T+ vs T- | NGF | 0.082483006 | 6.0745741 | 4.0387148 | 5.8639311e-05 | 0.00012118791 | 0.39304155 | 865 | 585 | 280 | TRUE | FALSE | up |
| ADNI | CSF | T+ vs T- | PDGFRB | 0.2258751 | 7.4625476 | 3.9018753 | 0.00010304596 | 0.00020947047 | -0.13977793 | 865 | 585 | 280 | TRUE | FALSE | up |
| ADNI | CSF | T+ vs T- | IL16 | 0.17560536 | 6.9396995 | 3.8092362 | 0.00014950053 | 0.00029900106 | -0.49043566 | 865 | 585 | 280 | TRUE | FALSE | up |
| ADNI | CSF | T+ vs T- | CHIT1 | 0.59412776 | 9.8484586 | 3.5035213 | 0.00048328522 | 0.00095122805 | -1.5897395 | 865 | 585 | 280 | TRUE | TRUE | up |
| ADNI | CSF | T+ vs T- | DDC | 0.13327701 | 6.4746458 | 3.4512018 | 0.00058580219 | 0.0011349918 | -1.7689335 | 865 | 585 | 280 | TRUE | FALSE | up |
| ADNI | CSF | T+ vs T- | ICAM1 | 0.17102175 | 5.7154497 | 3.3386938 | 0.00087859745 | 0.0016760936 | -2.1453884 | 865 | 585 | 280 | TRUE | FALSE | up |
| ADNI | CSF | T+ vs T- | IL6R | 0.23026936 | 6.9156142 | 3.1439826 | 0.0017249345 | 0.0031924161 | -2.7681583 | 865 | 585 | 280 | TRUE | FALSE | up |
| ADNI | CSF | T+ vs T- | POSTN | 0.2431141 | 3.8755617 | 3.1476024 | 0.0017039656 | 0.0031924161 | -2.7569137 | 865 | 585 | 280 | TRUE | FALSE | up |
| ADNI | CSF | T+ vs T- | TNF | 0.098514759 | 8.5166926 | 2.7289259 | 0.0064862455 | 0.011827859 | -3.9734329 | 865 | 585 | 280 | TRUE | FALSE | up |
| ADNI | CSF | T+ vs T- | VEGFD | 0.072356659 | 10.518116 | 2.6822032 | 0.0074568152 | 0.013400653 | -4.0986395 | 865 | 585 | 280 | TRUE | FALSE | up |
| ADNI | CSF | T+ vs T- | CSF2 | 0.09092843 | 11.926858 | 2.2936601 | 0.022054891 | 0.039068664 | -5.0573309 | 865 | 585 | 280 | TRUE | FALSE | up |
| ADNI | CSF | T+ vs T- | CXCL1 | 0.091258 | 9.8111605 | 2.2808747 | 0.022804229 | 0.039827105 | -5.0863674 | 865 | 585 | 280 | TRUE | FALSE | up |
| ADNI | CSF | T+ vs T- | CCL2 | 0.08561116 | 11.470998 | 2.2096947 | 0.027394036 | 0.047178618 | -5.2450894 | 865 | 585 | 280 | TRUE | FALSE | up |
| ADNI | CSF | T+ vs T- | RUVBL2 | -0.058158919 | 10.057577 | -2.1989568 | 0.028151546 | 0.047819064 | -5.2686018 | 865 | 585 | 280 | TRUE | FALSE | down |
| ADNI | CSF | T+ vs T- | MME | -0.050955348 | 0.22139954 | -2.0852437 | 0.037347053 | 0.062581548 | -5.5106429 | 865 | 585 | 280 | FALSE | FALSE | down |
| ADNI | CSF | T+ vs T- | CXCL8 | 0.075205231 | 12.99566 | 2.0314073 | 0.042526472 | 0.070310434 | -5.6207995 | 865 | 585 | 280 | FALSE | FALSE | up |
| ADNI | CSF | T+ vs T- | IL2 | 0.078091255 | 6.1375557 | 1.8599243 | 0.063243849 | 0.10213946 | -5.9526466 | 865 | 585 | 280 | FALSE | FALSE | up |
| ADNI | CSF | T+ vs T- | S100A12 | -0.1851512 | 4.0366743 | -1.8586449 | 0.063425313 | 0.10213946 | -5.9550135 | 865 | 585 | 280 | FALSE | FALSE | down |
| ADNI | CSF | T+ vs T- | SAA1 | -0.10214756 | 0.21416277 | -1.8248534 | 0.068376403 | 0.10870095 | -6.0169422 | 865 | 585 | 280 | FALSE | FALSE | down |
| ADNI | CSF | T+ vs T- | CCL11 | 0.06469871 | 8.8768174 | 1.7262707 | 0.084664568 | 0.13289122 | -6.1911681 | 865 | 585 | 280 | FALSE | FALSE | up |
| ADNI | CSF | T+ vs T- | SFTPD | 0.15827796 | 7.612795 | 1.7051902 | 0.08852682 | 0.13721657 | -6.2271774 | 865 | 585 | 280 | FALSE | FALSE | up |
| ADNI | CSF | T+ vs T- | FCN2 | -0.11149118 | 1.1916955 | -1.546116 | 0.12245131 | 0.18745632 | -6.4847287 | 865 | 585 | 280 | FALSE | FALSE | down |
| ADNI | CSF | T+ vs T- | UBB | 0.0116582 | 11.108963 | 1.4247886 | 0.15458786 | 0.23376701 | -6.6643175 | 865 | 585 | 280 | FALSE | FALSE | up |
| ADNI | CSF | T+ vs T- | TARDBP | -0.073508294 | 5.8957293 | -1.3506732 | 0.17716206 | 0.26467585 | -6.7668405 | 865 | 585 | 280 | FALSE | FALSE | down |
| ADNI | CSF | T+ vs T- | PARK7 | 0.041656685 | 10.851027 | 1.2776964 | 0.20170771 | 0.29644089 | -6.8624609 | 865 | 585 | 280 | FALSE | FALSE | up |
| ADNI | CSF | T+ vs T- | YWHAZ | 0.014561217 | 11.639545 | 1.2734598 | 0.20320545 | 0.29644089 | -6.8678497 | 865 | 585 | 280 | FALSE | FALSE | up |
| ADNI | CSF | T+ vs T- | CD40LG | -0.10846016 | 4.9171758 | -1.238724 | 0.21579201 | 0.31114197 | -6.9113592 | 865 | 585 | 280 | FALSE | FALSE | down |
| ADNI | CSF | T+ vs T- | GDF15 | 0.15097055 | 5.7447086 | 1.2170087 | 0.22394098 | 0.31918025 | -6.9379505 | 865 | 585 | 280 | FALSE | FALSE | up |
| ADNI | CSF | T+ vs T- | CCL26 | 0.078691778 | 5.7571109 | 1.1779242 | 0.23915877 | 0.33699645 | -6.9846304 | 865 | 585 | 280 | FALSE | FALSE | up |
| ADNI | CSF | T+ vs T- | CXCL10 | 0.086171421 | 9.3540903 | 1.1385429 | 0.25521682 | 0.35558299 | -7.0301286 | 865 | 585 | 280 | FALSE | FALSE | up |
| ADNI | CSF | T+ vs T- | CCL4 | 0.065829777 | 8.4596226 | 1.120347 | 0.26288451 | 0.35821626 | -7.0506299 | 865 | 585 | 280 | FALSE | FALSE | up |
| ADNI | CSF | T+ vs T- | IL10 | 0.067635393 | 9.3832643 | 1.1236475 | 0.26148202 | 0.35821626 | -7.0469357 | 865 | 585 | 280 | FALSE | FALSE | up |
| ADNI | CSF | T+ vs T- | BDNF | -0.101021 | 0.81554271 | -1.0399266 | 0.29867172 | 0.40255754 | -7.1372947 | 865 | 585 | 280 | FALSE | FALSE | down |
| ADNI | CSF | T+ vs T- | HTT | 0.074152624 | 6.7923075 | 1.0248905 | 0.3057084 | 0.40327492 | -7.1527841 | 865 | 585 | 280 | FALSE | FALSE | up |
| ADNI | CSF | T+ vs T- | VGF | -0.022595127 | 9.981309 | -1.0277243 | 0.30437391 | 0.40327492 | -7.1498822 | 865 | 585 | 280 | FALSE | FALSE | down |
| ADNI | CSF | T+ vs T- | ARSA | -0.063364969 | 6.0160435 | -1.0095274 | 0.31301102 | 0.40856175 | -7.1683781 | 865 | 585 | 280 | FALSE | FALSE | down |
| ADNI | CSF | T+ vs T- | CCL22 | 0.095292342 | 2.8087159 | 0.99937424 | 0.31789978 | 0.41062056 | -7.1785549 | 865 | 585 | 280 | FALSE | FALSE | up |
| ADNI | CSF | T+ vs T- | IL13 | 0.039246788 | 9.6162048 | 0.98890552 | 0.3229927 | 0.41289789 | -7.1889405 | 865 | 585 | 280 | FALSE | FALSE | up |
| ADNI | CSF | T+ vs T- | pSNCA-129 | 0.030071024 | 8.657933 | 0.95379407 | 0.3404609 | 0.43078726 | -7.2229764 | 865 | 585 | 280 | FALSE | FALSE | up |
| ADNI | CSF | T+ vs T- | NEFH | 0.04642388 | 21.249659 | 0.93445586 | 0.35033599 | 0.43880467 | -7.241198 | 865 | 585 | 280 | FALSE | FALSE | up |
| ADNI | CSF | T+ vs T- | IL7 | -0.036009073 | 10.320323 | -0.89314462 | 0.37203425 | 0.46132247 | -7.278876 | 865 | 585 | 280 | FALSE | FALSE | down |
| ADNI | CSF | T+ vs T- | IL4 | -0.04514483 | 8.423937 | -0.86408848 | 0.38778472 | 0.47609213 | -7.3043584 | 865 | 585 | 280 | FALSE | FALSE | down |
| ADNI | CSF | T+ vs T- | CCL13 | 0.058550936 | 4.7364394 | 0.85420777 | 0.3932322 | 0.47804698 | -7.3128321 | 865 | 585 | 280 | FALSE | FALSE | up |
| ADNI | CSF | T+ vs T- | CCL17 | 0.049824806 | 4.2832576 | 0.83760141 | 0.40249166 | 0.48455306 | -7.3268547 | 865 | 585 | 280 | FALSE | FALSE | up |
| ADNI | CSF | T+ vs T- | CST3 | 0.0081355042 | 13.768563 | 0.7973841 | 0.4254522 | 0.50726993 | -7.3596757 | 865 | 585 | 280 | FALSE | FALSE | up |
| ADNI | CSF | T+ vs T- | IL6 | -0.02479683 | 12.344641 | -0.65793732 | 0.51075776 | 0.59979072 | -7.4609878 | 865 | 585 | 280 | FALSE | FALSE | down |
| ADNI | CSF | T+ vs T- | pTDP43-409 | -0.0080989804 | 9.6280056 | -0.65487856 | 0.51272433 | 0.59979072 | -7.4629928 | 865 | 585 | 280 | FALSE | FALSE | down |
| ADNI | CSF | T+ vs T- | IL1B | -0.011747912 | 10.307333 | -0.5893715 | 0.55576977 | 0.64406964 | -7.50369 | 865 | 585 | 280 | FALSE | FALSE | down |
| ADNI | CSF | T+ vs T- | KDR | 0.07731497 | 6.752261 | 0.5592012 | 0.57617272 | 0.66153164 | -7.5209937 | 865 | 585 | 280 | FALSE | FALSE | up |
| ADNI | CSF | T+ vs T- | Abeta42 | 0.049217556 | 14.296006 | 0.51210019 | 0.60871483 | 0.69248292 | -7.5461915 | 865 | 585 | 280 | FALSE | FALSE | up |
| ADNI | CSF | T+ vs T- | IL17A | 0.007443337 | 9.1512487 | 0.44074936 | 0.65950729 | 0.74344459 | -7.5801443 | 865 | 585 | 280 | FALSE | FALSE | up |
| ADNI | CSF | T+ vs T- | GDNF | 0.004140321 | 8.8452899 | 0.37743392 | 0.70594602 | 0.78147213 | -7.6060171 | 865 | 585 | 280 | FALSE | FALSE | up |
| ADNI | CSF | T+ vs T- | IL18 | -0.017195402 | 6.046538 | -0.36570753 | 0.71467494 | 0.78147213 | -7.6103695 | 865 | 585 | 280 | FALSE | FALSE | down |
| ADNI | CSF | T+ vs T- | PDLIM5 | -0.0094440194 | 9.3722644 | -0.37417114 | 0.70837095 | 0.78147213 | -7.6072419 | 865 | 585 | 280 | FALSE | FALSE | down |
| ADNI | CSF | T+ vs T- | PRDX6 | -0.036259465 | 8.1238801 | -0.36065153 | 0.71845018 | 0.78147213 | -7.6122038 | 865 | 585 | 280 | FALSE | FALSE | down |
| ADNI | CSF | T+ vs T- | HBA1 | -0.11014258 | 4.5355013 | -0.33348009 | 0.73885472 | 0.78305971 | -7.621624 | 865 | 585 | 280 | FALSE | FALSE | down |
| ADNI | CSF | T+ vs T- | IL33 | 0.013158934 | 9.300228 | 0.33980422 | 0.73408849 | 0.78305971 | -7.6194973 | 865 | 585 | 280 | FALSE | FALSE | up |
| ADNI | CSF | T+ vs T- | PGF | 0.014664346 | 14.686982 | 0.34054877 | 0.73352803 | 0.78305971 | -7.6192443 | 865 | 585 | 280 | FALSE | FALSE | up |
| ADNI | CSF | T+ vs T- | FGF2 | -0.020568602 | 9.8415851 | -0.26391338 | 0.79191107 | 0.83217773 | -7.642382 | 865 | 585 | 280 | FALSE | FALSE | down |
| ADNI | CSF | T+ vs T- | BASP1 | -0.0028807973 | 10.146421 | -0.22717546 | 0.82034231 | 0.85481048 | -7.6513938 | 865 | 585 | 280 | FALSE | FALSE | down |
| ADNI | CSF | T+ vs T- | FOLR1 | -0.0043361201 | 12.38248 | -0.18527521 | 0.85305768 | 0.88149294 | -7.6600263 | 865 | 585 | 280 | FALSE | FALSE | down |
| ADNI | CSF | T+ vs T- | IFNG | 0.0057651182 | 8.1147173 | 0.092796791 | 0.92608702 | 0.94561317 | -7.672872 | 865 | 585 | 280 | FALSE | FALSE | up |
| ADNI | CSF | T+ vs T- | Oligo-SNCA | -0.0060483061 | 5.5059386 | -0.087416298 | 0.93036134 | 0.94561317 | -7.6733563 | 865 | 585 | 280 | FALSE | FALSE | down |
| ADNI | CSF | T+ vs T- | IGFBP7 | -0.00055327519 | 13.9384 | -0.030639213 | 0.97556451 | 0.98349593 | -7.6767046 | 865 | 585 | 280 | FALSE | FALSE | down |
| ADNI | CSF | T+ vs T- | REST | 0.00016581592 | 8.0924338 | 0.0043532254 | 0.99652767 | 0.99652767 | -7.6771641 | 865 | 585 | 280 | FALSE | FALSE | up |

**ADNI | CSF | N+ vs N-**

| **Cohort** | **Fluid** | **Contrast** | **Protein** | **log₂FC** | **AveExpr** | **t** | **P.Value** | **FDR** | **B** | **N_total** | **N_group1** | **N_group2** | **Pass_FDR** | **Pass_both_thresholds** | **Direction** |
| --- | --- | --- | --- | --- | --- | --- | --- | --- | --- | --- | --- | --- | --- | --- | --- |
| ADNI | CSF | N+ vs N- | MAPT | 1.0387359 | 15.421986 | 17.878783 | 7.15861e-61 | 8.8766764e-59 | 127.69019 | 865 | 548 | 317 | TRUE | TRUE | up |
| ADNI | CSF | N+ vs N- | pTau-181 | 1.1960905 | 17.463133 | 16.53939 | 1.9429771e-53 | 1.2046458e-51 | 110.661 | 865 | 548 | 317 | TRUE | TRUE | up |
| ADNI | CSF | N+ vs N- | pTau-231 | 1.2085819 | 17.931362 | 15.881215 | 6.8429867e-50 | 2.8284345e-48 | 102.53863 | 865 | 548 | 317 | TRUE | TRUE | up |
| ADNI | CSF | N+ vs N- | pTau-217 | 1.290792 | 15.999841 | 15.496834 | 7.4329679e-48 | 2.30422e-46 | 97.877202 | 865 | 548 | 317 | TRUE | TRUE | up |
| ADNI | CSF | N+ vs N- | VSNL1 | 0.59125703 | 12.958872 | 13.627923 | 2.2714298e-38 | 5.6331458e-37 | 76.172278 | 865 | 548 | 317 | TRUE | TRUE | up |
| ADNI | CSF | N+ vs N- | NRGN | 0.60386321 | 7.443744 | 13.439795 | 1.8612789e-37 | 3.846643e-36 | 74.08321 | 865 | 548 | 317 | TRUE | TRUE | up |
| ADNI | CSF | N+ vs N- | FABP3 | 0.57341557 | 11.870522 | 13.34176 | 5.5289897e-37 | 9.7942103e-36 | 73.00202 | 865 | 548 | 317 | TRUE | TRUE | up |
| ADNI | CSF | N+ vs N- | SMOC1 | 0.80237672 | 11.946752 | 13.295425 | 9.2331667e-37 | 1.4311408e-35 | 72.492807 | 865 | 548 | 317 | TRUE | TRUE | up |
| ADNI | CSF | N+ vs N- | BACE1 | 0.42201156 | 14.800397 | 12.910911 | 6.2241463e-35 | 8.5754904e-34 | 68.312131 | 865 | 548 | 317 | TRUE | FALSE | up |
| ADNI | CSF | N+ vs N- | SOD1 | 0.48525774 | 13.817817 | 12.496375 | 5.318152e-33 | 6.5945085e-32 | 63.897572 | 865 | 548 | 317 | TRUE | FALSE | up |
| ADNI | CSF | N+ vs N- | MDH1 | 0.75263473 | 10.808306 | 12.470245 | 7.0162736e-33 | 7.9092538e-32 | 63.622595 | 865 | 548 | 317 | TRUE | TRUE | up |
| ADNI | CSF | N+ vs N- | GOT1 | 0.38320013 | 10.956185 | 11.933327 | 1.90829e-30 | 1.9718996e-29 | 58.061456 | 865 | 548 | 317 | TRUE | FALSE | up |
| ADNI | CSF | N+ vs N- | ENO2 | 0.45903527 | 10.798748 | 11.731761 | 1.4974249e-29 | 1.428313e-28 | 56.018509 | 865 | 548 | 317 | TRUE | FALSE | up |
| ADNI | CSF | N+ vs N- | SNCB | 0.27642526 | 11.166338 | 11.425656 | 3.2612967e-28 | 2.8885771e-27 | 52.96412 | 865 | 548 | 317 | TRUE | FALSE | up |
| ADNI | CSF | N+ vs N- | IGF1R | 0.27212791 | 13.081985 | 11.302523 | 1.1078476e-27 | 9.1582071e-27 | 51.752104 | 865 | 548 | 317 | TRUE | FALSE | up |
| ADNI | CSF | N+ vs N- | GDI1 | 0.34998443 | 8.8736365 | 11.054293 | 1.2660001e-26 | 9.8115006e-26 | 49.338292 | 865 | 548 | 317 | TRUE | FALSE | up |
| ADNI | CSF | N+ vs N- | AGRN | 0.42173835 | 14.214683 | 10.997677 | 2.1944651e-26 | 1.6006686e-25 | 48.793344 | 865 | 548 | 317 | TRUE | FALSE | up |
| ADNI | CSF | N+ vs N- | TAFA5 | 0.60589963 | 19.390467 | 10.625099 | 7.7769474e-25 | 5.3574527e-24 | 45.259872 | 865 | 548 | 317 | TRUE | TRUE | up |
| ADNI | CSF | N+ vs N- | KLK6 | 0.27007877 | 18.923171 | 10.391072 | 6.9769059e-24 | 4.5533491e-23 | 43.087966 | 865 | 548 | 317 | TRUE | FALSE | up |
| ADNI | CSF | N+ vs N- | YWHAG | 0.16257307 | 11.519674 | 10.079897 | 1.2180832e-22 | 7.5521159e-22 | 40.258236 | 865 | 548 | 317 | TRUE | FALSE | up |
| ADNI | CSF | N+ vs N- | SQSTM1 | 0.42495496 | 10.451519 | 10.070378 | 1.3280618e-22 | 7.841889e-22 | 40.172728 | 865 | 548 | 317 | TRUE | FALSE | up |
| ADNI | CSF | N+ vs N- | NPTXR | 0.43381162 | 16.940387 | 9.9051553 | 5.894428e-22 | 3.3223139e-21 | 38.698786 | 865 | 548 | 317 | TRUE | FALSE | up |
| ADNI | CSF | N+ vs N- | CHI3L1 | 0.37104697 | 15.108736 | 9.6546749 | 5.4426965e-21 | 2.9343233e-20 | 36.501191 | 865 | 548 | 317 | TRUE | FALSE | up |
| ADNI | CSF | N+ vs N- | SLIT2 | 0.24809937 | 10.214377 | 9.2691101 | 1.5270973e-19 | 7.8900026e-19 | 33.206995 | 865 | 548 | 317 | TRUE | FALSE | up |
| ADNI | CSF | N+ vs N- | PSEN1 | 0.25736657 | 11.312148 | 9.0735718 | 7.9496363e-19 | 3.9430196e-18 | 31.578145 | 865 | 548 | 317 | TRUE | FALSE | up |
| ADNI | CSF | N+ vs N- | NPTX1 | 0.37253598 | 14.78841 | 9.0686834 | 8.2813806e-19 | 3.9495815e-18 | 31.537789 | 865 | 548 | 317 | TRUE | FALSE | up |
| ADNI | CSF | N+ vs N- | UCHL1 | 0.10986917 | 11.741851 | 9.037594 | 1.0736092e-18 | 4.9306495e-18 | 31.281552 | 865 | 548 | 317 | TRUE | FALSE | up |
| ADNI | CSF | N+ vs N- | IL15 | 0.25981913 | 13.178702 | 9.0223289 | 1.2192441e-18 | 5.3995095e-18 | 31.156002 | 865 | 548 | 317 | TRUE | FALSE | up |
| ADNI | CSF | N+ vs N- | TIMP3 | 0.40108028 | 7.0810649 | 8.7923938 | 8.112138e-18 | 3.4686383e-17 | 29.286126 | 865 | 548 | 317 | TRUE | FALSE | up |
| ADNI | CSF | N+ vs N- | ACHE | 0.45687762 | 12.949521 | 8.7315726 | 1.3303756e-17 | 5.4988859e-17 | 28.798223 | 865 | 548 | 317 | TRUE | FALSE | up |
| ADNI | CSF | N+ vs N- | CALB2 | 0.36947767 | 14.003327 | 8.7145488 | 1.5271845e-17 | 6.108738e-17 | 28.662164 | 865 | 548 | 317 | TRUE | FALSE | up |
| ADNI | CSF | N+ vs N- | FLT1 | 0.33995751 | 11.585478 | 8.5147715 | 7.5844058e-17 | 2.9389573e-16 | 27.082123 | 865 | 548 | 317 | TRUE | FALSE | up |
| ADNI | CSF | N+ vs N- | VEGFA | 0.26751931 | 14.24775 | 8.4102181 | 1.7335497e-16 | 6.5139444e-16 | 26.267499 | 865 | 548 | 317 | TRUE | FALSE | up |
| ADNI | CSF | N+ vs N- | CX3CL1 | 0.2760368 | 10.495984 | 8.1622771 | 1.1905232e-15 | 4.3419082e-15 | 24.369803 | 865 | 548 | 317 | TRUE | FALSE | up |
| ADNI | CSF | N+ vs N- | Abeta38 | 0.69103323 | 17.392873 | 7.7596647 | 2.4562379e-14 | 8.7021001e-14 | 21.39204 | 865 | 548 | 317 | TRUE | TRUE | up |
| ADNI | CSF | N+ vs N- | GFAP | 0.49216974 | 17.178517 | 7.7510683 | 2.6165743e-14 | 9.0126448e-14 | 21.329878 | 865 | 548 | 317 | TRUE | FALSE | up |
| ADNI | CSF | N+ vs N- | SNAP25 | 0.12600356 | 12.219968 | 7.6564566 | 5.2275937e-14 | 1.7519503e-13 | 20.649675 | 865 | 548 | 317 | TRUE | FALSE | up |
| ADNI | CSF | N+ vs N- | NPTX2 | 0.36206408 | 14.412663 | 7.2601719 | 8.7781727e-13 | 2.8644563e-12 | 17.880066 | 865 | 548 | 317 | TRUE | FALSE | up |
| ADNI | CSF | N+ vs N- | CD63 | 0.16563479 | 9.8053129 | 7.2443859 | 9.7963706e-13 | 3.1147435e-12 | 17.772419 | 865 | 548 | 317 | TRUE | FALSE | up |
| ADNI | CSF | N+ vs N- | CNTN2 | 0.36167681 | 19.147286 | 7.2315733 | 1.0707405e-12 | 3.3192956e-12 | 17.6852 | 865 | 548 | 317 | TRUE | FALSE | up |
| ADNI | CSF | N+ vs N- | NEFL | 0.35677347 | 15.968484 | 7.0484193 | 3.761669e-12 | 1.1376755e-11 | 16.453365 | 865 | 548 | 317 | TRUE | FALSE | up |
| ADNI | CSF | N+ vs N- | VCAM1 | 0.29324165 | 8.8470681 | 6.6639892 | 4.8059654e-11 | 1.4189041e-10 | 13.959565 | 865 | 548 | 317 | TRUE | FALSE | up |
| ADNI | CSF | N+ vs N- | CRH | 0.29447818 | 10.340378 | 6.6517936 | 5.2000597e-11 | 1.4995521e-10 | 13.882505 | 865 | 548 | 317 | TRUE | FALSE | up |
| ADNI | CSF | N+ vs N- | IL5 | 0.29173929 | 13.069152 | 6.5185495 | 1.2202492e-10 | 3.4388841e-10 | 13.048875 | 865 | 548 | 317 | TRUE | FALSE | up |
| ADNI | CSF | N+ vs N- | TREM2 | 0.35635726 | 11.304917 | 6.4249382 | 2.202094e-10 | 6.0679925e-10 | 12.472327 | 865 | 548 | 317 | TRUE | FALSE | up |
| ADNI | CSF | N+ vs N- | MSLN | 0.34421416 | 9.011218 | 6.2451689 | 6.7019239e-10 | 1.8066056e-09 | 11.386376 | 865 | 548 | 317 | TRUE | FALSE | up |
| ADNI | CSF | N+ vs N- | Abeta40 | 0.61584533 | 14.923961 | 6.1897431 | 9.3935963e-10 | 2.4783105e-09 | 11.057221 | 865 | 548 | 317 | TRUE | TRUE | up |
| ADNI | CSF | N+ vs N- | IL9 | 0.27730158 | 11.430261 | 6.1829998 | 9.7857561e-10 | 2.527987e-09 | 11.017358 | 865 | 548 | 317 | TRUE | FALSE | up |
| ADNI | CSF | N+ vs N- | SNCA | 0.39440079 | 5.649085 | 6.1363535 | 1.2971946e-09 | 3.2826966e-09 | 10.742693 | 865 | 548 | 317 | TRUE | FALSE | up |
| ADNI | CSF | N+ vs N- | NPY | 0.38201351 | 9.7785854 | 6.0100521 | 2.7568113e-09 | 6.8368921e-09 | 10.008561 | 865 | 548 | 317 | TRUE | FALSE | up |
| ADNI | CSF | N+ vs N- | SFRP1 | 0.50080874 | 13.945303 | 5.7244934 | 1.4413075e-08 | 3.5043554e-08 | 8.4005511 | 865 | 548 | 317 | TRUE | TRUE | up |
| ADNI | CSF | N+ vs N- | ANXA5 | 0.39272884 | 6.5454661 | 5.5252665 | 4.3836507e-08 | 1.0453321e-07 | 7.3215913 | 865 | 548 | 317 | TRUE | FALSE | up |
| ADNI | CSF | N+ vs N- | TEK | 0.22055416 | 6.014131 | 5.4941448 | 5.1993437e-08 | 1.2164502e-07 | 7.156251 | 865 | 548 | 317 | TRUE | FALSE | up |
| ADNI | CSF | N+ vs N- | S100B | 0.22341313 | 15.569714 | 5.4088369 | 8.264594e-08 | 1.8977957e-07 | 6.7074978 | 865 | 548 | 317 | TRUE | FALSE | up |
| ADNI | CSF | N+ vs N- | PGK1 | 0.24255481 | 4.6726958 | 5.0939197 | 4.3282398e-07 | 9.7582133e-07 | 5.1078286 | 865 | 548 | 317 | TRUE | FALSE | up |
| ADNI | CSF | N+ vs N- | TREM1 | 0.25083747 | 7.421262 | 4.9221939 | 1.0291863e-06 | 2.2789125e-06 | 4.2735297 | 865 | 548 | 317 | TRUE | FALSE | up |
| ADNI | CSF | N+ vs N- | IL12p70 | 0.23397632 | 9.0801764 | 4.898817 | 1.1556595e-06 | 2.5140662e-06 | 4.1620439 | 865 | 548 | 317 | TRUE | FALSE | up |
| ADNI | CSF | N+ vs N- | PTN | 0.23858884 | 10.258457 | 4.3508892 | 1.5217773e-05 | 3.2534548e-05 | 1.6934059 | 865 | 548 | 317 | TRUE | FALSE | up |
| ADNI | CSF | N+ vs N- | IL16 | 0.19081593 | 6.9396995 | 4.2484539 | 2.391992e-05 | 5.0272374e-05 | 1.2628613 | 865 | 548 | 317 | TRUE | FALSE | up |
| ADNI | CSF | N+ vs N- | CCL3 | 0.17834701 | 9.3597907 | 4.131537 | 3.9623587e-05 | 8.1888746e-05 | 0.78346627 | 865 | 548 | 317 | TRUE | FALSE | up |
| ADNI | CSF | N+ vs N- | NGF | 0.079978599 | 6.0745741 | 4.01076 | 6.5887128e-05 | 0.00013393449 | 0.30174026 | 865 | 548 | 317 | TRUE | FALSE | up |
| ADNI | CSF | N+ vs N- | PDGFRB | 0.22299526 | 7.4625476 | 3.9464944 | 8.5900898e-05 | 0.0001718018 | 0.051020214 | 865 | 548 | 317 | TRUE | FALSE | up |
| ADNI | CSF | N+ vs N- | CHIT1 | 0.58800506 | 9.8484586 | 3.5523403 | 0.00040297839 | 0.00078269492 | -1.401042 | 865 | 548 | 317 | TRUE | TRUE | up |
| ADNI | CSF | N+ vs N- | ICAM1 | 0.17746552 | 5.7154497 | 3.5516829 | 0.00040397157 | 0.00078269492 | -1.4033405 | 865 | 548 | 317 | TRUE | FALSE | up |
| ADNI | CSF | N+ vs N- | TNF | 0.12190061 | 8.5166926 | 3.4680496 | 0.00055076216 | 0.0010506847 | -1.6923594 | 865 | 548 | 317 | TRUE | FALSE | up |
| ADNI | CSF | N+ vs N- | DDC | 0.12847635 | 6.4746458 | 3.4071045 | 0.00068760394 | 0.0012918619 | -1.8987564 | 865 | 548 | 317 | TRUE | FALSE | up |
| ADNI | CSF | N+ vs N- | POSTN | 0.25038267 | 3.8755617 | 3.3226419 | 0.00093004342 | 0.0017212744 | -2.1789103 | 865 | 548 | 317 | TRUE | FALSE | up |
| ADNI | CSF | N+ vs N- | VEGFD | 0.084299513 | 10.518116 | 3.206648 | 0.0013934605 | 0.0025410162 | -2.5524803 | 865 | 548 | 317 | TRUE | FALSE | up |
| ADNI | CSF | N+ vs N- | IL6R | 0.21825302 | 6.9156142 | 3.0512658 | 0.0023500943 | 0.0042233579 | -3.032592 | 865 | 548 | 317 | TRUE | FALSE | up |
| ADNI | CSF | N+ vs N- | CSF2 | 0.11731844 | 11.926858 | 3.0383093 | 0.0024523788 | 0.0043442138 | -3.0715729 | 865 | 548 | 317 | TRUE | FALSE | up |
| ADNI | CSF | N+ vs N- | SFTPD | 0.22579567 | 7.612795 | 2.4965499 | 0.012730419 | 0.022233407 | -4.5557243 | 865 | 548 | 317 | TRUE | FALSE | up |
| ADNI | CSF | N+ vs N- | CCL2 | 0.087488309 | 11.470998 | 2.3136412 | 0.02092679 | 0.036040583 | -4.9921932 | 865 | 548 | 317 | TRUE | FALSE | up |
| ADNI | CSF | N+ vs N- | CXCL1 | 0.085043804 | 9.8111605 | 2.1765969 | 0.029787141 | 0.050597335 | -5.2977343 | 865 | 548 | 317 | FALSE | FALSE | up |
| ADNI | CSF | N+ vs N- | IL2 | 0.087518423 | 6.1375557 | 2.1364796 | 0.032927287 | 0.055175455 | -5.3836872 | 865 | 548 | 317 | FALSE | FALSE | up |
| ADNI | CSF | N+ vs N- | SAA1 | -0.11490547 | 0.21416277 | -2.1039912 | 0.035674422 | 0.05898171 | -5.4521358 | 865 | 548 | 317 | FALSE | FALSE | down |
| ADNI | CSF | N+ vs N- | UBB | 0.015260572 | 11.108963 | 1.9124512 | 0.056156488 | 0.091623743 | -5.8345685 | 865 | 548 | 317 | FALSE | FALSE | up |
| ADNI | CSF | N+ vs N- | CXCL8 | 0.065214484 | 12.99566 | 1.803405 | 0.071681056 | 0.11536011 | -6.0361379 | 865 | 548 | 317 | FALSE | FALSE | up |
| ADNI | CSF | N+ vs N- | YWHAZ | 0.020048819 | 11.639545 | 1.7978043 | 0.072565233 | 0.11536011 | -6.0461738 | 865 | 548 | 317 | FALSE | FALSE | up |
| ADNI | CSF | N+ vs N- | RUVBL2 | -0.045998344 | 10.057577 | -1.7796827 | 0.075487548 | 0.11848678 | -6.0784335 | 865 | 548 | 317 | FALSE | FALSE | down |
| ADNI | CSF | N+ vs N- | PARK7 | 0.05551573 | 10.851027 | 1.7456088 | 0.081242734 | 0.12592624 | -6.1382128 | 865 | 548 | 317 | FALSE | FALSE | up |
| ADNI | CSF | N+ vs N- | CCL13 | 0.11324193 | 4.7364394 | 1.694371 | 0.090563612 | 0.13864059 | -6.2259444 | 865 | 548 | 317 | FALSE | FALSE | up |
| ADNI | CSF | N+ vs N- | MME | -0.038353269 | 0.22139954 | -1.6059956 | 0.10864882 | 0.16429821 | -6.3711635 | 865 | 548 | 317 | FALSE | FALSE | down |
| ADNI | CSF | N+ vs N- | CCL4 | 0.089920159 | 8.4596226 | 1.5686243 | 0.11711022 | 0.17067733 | -6.4302471 | 865 | 548 | 317 | FALSE | FALSE | up |
| ADNI | CSF | N+ vs N- | CXCL10 | 0.11653928 | 9.3540903 | 1.5782904 | 0.11487351 | 0.17067733 | -6.4150976 | 865 | 548 | 317 | FALSE | FALSE | up |
| ADNI | CSF | N+ vs N- | HTT | 0.11032977 | 6.7923075 | 1.5632311 | 0.11837299 | 0.17067733 | -6.4386593 | 865 | 548 | 317 | FALSE | FALSE | up |
| ADNI | CSF | N+ vs N- | VGF | -0.033741385 | 9.981309 | -1.573326 | 0.11601801 | 0.17067733 | -6.4228897 | 865 | 548 | 317 | FALSE | FALSE | down |
| ADNI | CSF | N+ vs N- | GDF15 | 0.1878719 | 5.7447086 | 1.5521111 | 0.12101042 | 0.17247462 | -6.4559136 | 865 | 548 | 317 | FALSE | FALSE | up |
| ADNI | CSF | N+ vs N- | pSNCA-129 | 0.044732953 | 8.657933 | 1.4543494 | 0.14622112 | 0.20603884 | -6.6023308 | 865 | 548 | 317 | FALSE | FALSE | up |
| ADNI | CSF | N+ vs N- | S100A12 | -0.13705958 | 4.0366743 | -1.4080674 | 0.15947923 | 0.22219577 | -6.6683428 | 865 | 548 | 317 | FALSE | FALSE | down |
| ADNI | CSF | N+ vs N- | NEFH | 0.06638034 | 21.249659 | 1.3694209 | 0.17123189 | 0.23591949 | -6.7218364 | 865 | 548 | 317 | FALSE | FALSE | up |
| ADNI | CSF | N+ vs N- | IL17A | 0.021932397 | 9.1512487 | 1.3315315 | 0.18337358 | 0.24970138 | -6.7728432 | 865 | 548 | 317 | FALSE | FALSE | up |
| ADNI | CSF | N+ vs N- | KDR | 0.17880645 | 6.752261 | 1.3258068 | 0.18526231 | 0.24970138 | -6.7804259 | 865 | 548 | 317 | FALSE | FALSE | up |
| ADNI | CSF | N+ vs N- | IL13 | 0.048295825 | 9.6162048 | 1.2469016 | 0.2127794 | 0.28370587 | -6.8816249 | 865 | 548 | 317 | FALSE | FALSE | up |
| ADNI | CSF | N+ vs N- | IL10 | 0.072810439 | 9.3832643 | 1.2392019 | 0.21561511 | 0.28442845 | -6.8911689 | 865 | 548 | 317 | FALSE | FALSE | up |
| ADNI | CSF | N+ vs N- | IL33 | 0.045264601 | 9.300228 | 1.1982017 | 0.23117478 | 0.30174393 | -6.9409978 | 865 | 548 | 317 | FALSE | FALSE | up |
| ADNI | CSF | N+ vs N- | IL6 | -0.041393806 | 12.344641 | -1.1255429 | 0.26067891 | 0.33671026 | -7.025198 | 865 | 548 | 317 | FALSE | FALSE | down |
| ADNI | CSF | N+ vs N- | Abeta42 | 0.10250038 | 14.296006 | 1.0930035 | 0.27470405 | 0.35116807 | -7.0612042 | 865 | 548 | 317 | FALSE | FALSE | up |
| ADNI | CSF | N+ vs N- | TARDBP | -0.056711495 | 5.8957293 | -1.0669189 | 0.28631347 | 0.36227418 | -7.0893073 | 865 | 548 | 317 | FALSE | FALSE | down |
| ADNI | CSF | N+ vs N- | CCL11 | 0.038405779 | 8.8768174 | 1.0484588 | 0.29472734 | 0.36915344 | -7.1087868 | 865 | 548 | 317 | FALSE | FALSE | up |
| ADNI | CSF | N+ vs N- | PDLIM5 | -0.024618589 | 9.3722644 | -0.99959735 | 0.31779182 | 0.39406186 | -7.1587098 | 865 | 548 | 317 | FALSE | FALSE | down |
| ADNI | CSF | N+ vs N- | pTDP43-409 | -0.011182691 | 9.6280056 | -0.92647901 | 0.35446187 | 0.43518091 | -7.2289772 | 865 | 548 | 317 | FALSE | FALSE | down |
| ADNI | CSF | N+ vs N- | FCN2 | -0.063474289 | 1.1916955 | -0.90077417 | 0.36796529 | 0.44733035 | -7.252415 | 865 | 548 | 317 | FALSE | FALSE | down |
| ADNI | CSF | N+ vs N- | CCL26 | 0.057939364 | 5.7571109 | 0.88803461 | 0.37477506 | 0.45118551 | -7.263787 | 865 | 548 | 317 | FALSE | FALSE | up |
| ADNI | CSF | N+ vs N- | BDNF | -0.080712924 | 0.81554271 | -0.85087114 | 0.3950822 | 0.47105954 | -7.2960375 | 865 | 548 | 317 | FALSE | FALSE | down |
| ADNI | CSF | N+ vs N- | HBA1 | -0.26314173 | 4.5355013 | -0.81633471 | 0.414539 | 0.48955082 | -7.3247745 | 865 | 548 | 317 | FALSE | FALSE | down |
| ADNI | CSF | N+ vs N- | IL7 | -0.031692032 | 10.320323 | -0.80509138 | 0.42099362 | 0.49248311 | -7.3338733 | 865 | 548 | 317 | FALSE | FALSE | down |
| ADNI | CSF | N+ vs N- | FOLR1 | 0.014834648 | 12.38248 | 0.64941393 | 0.5162475 | 0.59272861 | -7.4469045 | 865 | 548 | 317 | FALSE | FALSE | up |
| ADNI | CSF | N+ vs N- | IL1B | -0.012672506 | 10.307333 | -0.65124186 | 0.51506759 | 0.59272861 | -7.4457175 | 865 | 548 | 317 | FALSE | FALSE | down |
| ADNI | CSF | N+ vs N- | ARSA | -0.038426245 | 6.0160435 | -0.62684196 | 0.53093231 | 0.60399639 | -7.4612868 | 865 | 548 | 317 | FALSE | FALSE | down |
| ADNI | CSF | N+ vs N- | IFNG | 0.03597832 | 8.1147173 | 0.59330242 | 0.55313769 | 0.62353703 | -7.4817187 | 865 | 548 | 317 | FALSE | FALSE | up |
| ADNI | CSF | N+ vs N- | CCL17 | 0.033458005 | 4.2832576 | 0.57599546 | 0.56477176 | 0.6309162 | -7.491823 | 865 | 548 | 317 | FALSE | FALSE | up |
| ADNI | CSF | N+ vs N- | IL4 | -0.028618704 | 8.423937 | -0.56093398 | 0.57499139 | 0.63659761 | -7.500373 | 865 | 548 | 317 | FALSE | FALSE | down |
| ADNI | CSF | N+ vs N- | IL18 | 0.022048768 | 6.046538 | 0.48034563 | 0.63110609 | 0.69254119 | -7.5422747 | 865 | 548 | 317 | FALSE | FALSE | up |
| ADNI | CSF | N+ vs N- | CD40LG | -0.036642088 | 4.9171758 | -0.42831085 | 0.66853407 | 0.72717741 | -7.565886 | 865 | 548 | 317 | FALSE | FALSE | down |
| ADNI | CSF | N+ vs N- | Oligo-SNCA | -0.024152486 | 5.5059386 | -0.35758014 | 0.72074691 | 0.77715319 | -7.5936462 | 865 | 548 | 317 | FALSE | FALSE | down |
| ADNI | CSF | N+ vs N- | BASP1 | -0.0039646669 | 10.146421 | -0.32026954 | 0.74884326 | 0.80048763 | -7.6062773 | 865 | 548 | 317 | FALSE | FALSE | down |
| ADNI | CSF | N+ vs N- | IGFBP7 | -0.003311025 | 13.9384 | -0.18781991 | 0.85106296 | 0.89543148 | -7.6398888 | 865 | 548 | 317 | FALSE | FALSE | down |
| ADNI | CSF | N+ vs N- | REST | 0.0069349158 | 8.0924338 | 0.1864915 | 0.85210415 | 0.89543148 | -7.6401371 | 865 | 548 | 317 | FALSE | FALSE | up |
| ADNI | CSF | N+ vs N- | PGF | 0.0074245599 | 14.686982 | 0.17659927 | 0.85986552 | 0.89599433 | -7.6419312 | 865 | 548 | 317 | FALSE | FALSE | up |
| ADNI | CSF | N+ vs N- | CCL22 | 0.01067335 | 2.8087159 | 0.11458799 | 0.90879895 | 0.93909225 | -7.6509503 | 865 | 548 | 317 | FALSE | FALSE | up |
| ADNI | CSF | N+ vs N- | FGF2 | 0.0048830678 | 9.8415851 | 0.064173294 | 0.94884743 | 0.96440231 | -7.6554518 | 865 | 548 | 317 | FALSE | FALSE | up |
| ADNI | CSF | N+ vs N- | GDNF | -0.00073440686 | 8.8452899 | -0.068574723 | 0.94534438 | 0.96440231 | -7.65516 | 865 | 548 | 317 | FALSE | FALSE | down |
| ADNI | CSF | N+ vs N- | CST3 | -0.0003866551 | 13.768563 | -0.038806547 | 0.9690538 | 0.9690538 | -7.6567566 | 865 | 548 | 317 | FALSE | FALSE | down |
| ADNI | CSF | N+ vs N- | PRDX6 | -0.0045456823 | 8.1238801 | -0.046307792 | 0.96307588 | 0.9690538 | -7.6564377 | 865 | 548 | 317 | FALSE | FALSE | down |

**ADNI | CSF | A+T+N+ vs A+T-N-**

| **Cohort** | **Fluid** | **Contrast** | **Protein** | **log₂FC** | **AveExpr** | **t** | **P.Value** | **FDR** | **B** | **N_total** | **N_group1** | **N_group2** | **Pass_FDR** | **Pass_both_thresholds** | **Direction** |
| --- | --- | --- | --- | --- | --- | --- | --- | --- | --- | --- | --- | --- | --- | --- | --- |
| ADNI | CSF | A+T+N+ vs A+T-N- | MAPT | 1.1262239 | 15.427501 | 18.41724 | 2.2010591e-63 | 2.7293133e-61 | 133.44896 | 818 | 543 | 275 | TRUE | TRUE | up |
| ADNI | CSF | A+T+N+ vs A+T-N- | pTau-181 | 1.314871 | 17.464308 | 17.340592 | 2.1254556e-57 | 1.3177825e-55 | 119.73351 | 818 | 543 | 275 | TRUE | TRUE | up |
| ADNI | CSF | A+T+N+ vs A+T-N- | pTau-231 | 1.333167 | 17.930766 | 16.677578 | 8.5724961e-54 | 3.5432984e-52 | 111.47241 | 818 | 543 | 275 | TRUE | TRUE | up |
| ADNI | CSF | A+T+N+ vs A+T-N- | pTau-217 | 1.4311194 | 15.994456 | 16.38782 | 3.0761225e-52 | 9.5359798e-51 | 107.91047 | 818 | 543 | 275 | TRUE | TRUE | up |
| ADNI | CSF | A+T+N+ vs A+T-N- | VSNL1 | 0.6364948 | 12.966714 | 13.889225 | 1.9453284e-39 | 4.8244144e-38 | 78.603521 | 818 | 543 | 275 | TRUE | TRUE | up |
| ADNI | CSF | A+T+N+ vs A+T-N- | SMOC1 | 0.87267234 | 11.946587 | 13.728294 | 1.1799311e-38 | 2.4385243e-37 | 76.812533 | 818 | 543 | 275 | TRUE | TRUE | up |
| ADNI | CSF | A+T+N+ vs A+T-N- | FABP3 | 0.61552486 | 11.878117 | 13.548652 | 8.6931434e-38 | 1.5399283e-36 | 74.828561 | 818 | 543 | 275 | TRUE | TRUE | up |
| ADNI | CSF | A+T+N+ vs A+T-N- | NRGN | 0.63449715 | 7.4552128 | 13.357662 | 7.1376618e-37 | 1.1063376e-35 | 72.737219 | 818 | 543 | 275 | TRUE | TRUE | up |
| ADNI | CSF | A+T+N+ vs A+T-N- | BACE1 | 0.43802575 | 14.811429 | 12.628903 | 1.8479759e-33 | 2.5461001e-32 | 64.93357 | 818 | 543 | 275 | TRUE | FALSE | up |
| ADNI | CSF | A+T+N+ vs A+T-N- | MDH1 | 0.79145752 | 10.824454 | 12.298953 | 5.9006334e-32 | 7.3167854e-31 | 61.496014 | 818 | 543 | 275 | TRUE | TRUE | up |
| ADNI | CSF | A+T+N+ vs A+T-N- | SOD1 | 0.50249576 | 13.82975 | 12.16414 | 2.3869668e-31 | 2.6907626e-30 | 60.109264 | 818 | 543 | 275 | TRUE | TRUE | up |
| ADNI | CSF | A+T+N+ vs A+T-N- | ENO2 | 0.49178117 | 10.802922 | 11.837286 | 6.7692659e-30 | 6.9949081e-29 | 56.790944 | 818 | 543 | 275 | TRUE | FALSE | up |
| ADNI | CSF | A+T+N+ vs A+T-N- | GOT1 | 0.40109962 | 10.964545 | 11.759921 | 1.4805231e-29 | 1.4121912e-28 | 56.014757 | 818 | 543 | 275 | TRUE | FALSE | up |
| ADNI | CSF | A+T+N+ vs A+T-N- | IGF1R | 0.28957801 | 13.085856 | 11.355457 | 8.35586e-28 | 7.4009045e-27 | 52.015703 | 818 | 543 | 275 | TRUE | FALSE | up |
| ADNI | CSF | A+T+N+ vs A+T-N- | GDI1 | 0.37672974 | 8.8759208 | 11.303059 | 1.3987709e-27 | 1.1563173e-26 | 51.504985 | 818 | 543 | 275 | TRUE | FALSE | up |
| ADNI | CSF | A+T+N+ vs A+T-N- | SNCB | 0.28801435 | 11.169141 | 11.237987 | 2.6461115e-27 | 2.0507364e-26 | 50.873101 | 818 | 543 | 275 | TRUE | FALSE | up |
| ADNI | CSF | A+T+N+ vs A+T-N- | AGRN | 0.44129046 | 14.224782 | 10.87108 | 9.1666335e-26 | 6.6862503e-25 | 47.360258 | 818 | 543 | 275 | TRUE | FALSE | up |
| ADNI | CSF | A+T+N+ vs A+T-N- | TAFA5 | 0.63203756 | 19.40309 | 10.500438 | 3.0175623e-24 | 2.0787651e-23 | 43.8998 | 818 | 543 | 275 | TRUE | TRUE | up |
| ADNI | CSF | A+T+N+ vs A+T-N- | SQSTM1 | 0.45044982 | 10.458739 | 10.122576 | 9.6915477e-23 | 6.3250101e-22 | 40.465819 | 818 | 543 | 275 | TRUE | FALSE | up |
| ADNI | CSF | A+T+N+ vs A+T-N- | KLK6 | 0.27568263 | 18.931174 | 10.078607 | 1.4422077e-22 | 8.941688e-22 | 40.072514 | 818 | 543 | 275 | TRUE | FALSE | up |
| ADNI | CSF | A+T+N+ vs A+T-N- | YWHAG | 0.16862962 | 11.523362 | 9.9090572 | 6.5977092e-22 | 3.8957902e-21 | 38.568329 | 818 | 543 | 275 | TRUE | FALSE | up |
| ADNI | CSF | A+T+N+ vs A+T-N- | CHI3L1 | 0.39175563 | 15.118684 | 9.6807255 | 4.9565628e-21 | 2.793699e-20 | 36.574214 | 818 | 543 | 275 | TRUE | FALSE | up |
| ADNI | CSF | A+T+N+ vs A+T-N- | NPTXR | 0.44778642 | 16.94762 | 9.6421764 | 6.9421217e-21 | 3.7427091e-20 | 36.24116 | 818 | 543 | 275 | TRUE | FALSE | up |
| ADNI | CSF | A+T+N+ vs A+T-N- | SLIT2 | 0.26445121 | 10.217538 | 9.3479922 | 8.7729089e-20 | 4.5326696e-19 | 33.734319 | 818 | 543 | 275 | TRUE | FALSE | up |
| ADNI | CSF | A+T+N+ vs A+T-N- | ACHE | 0.5054087 | 12.951106 | 9.2484303 | 2.0414973e-19 | 1.0125827e-18 | 32.900008 | 818 | 543 | 275 | TRUE | TRUE | up |
| ADNI | CSF | A+T+N+ vs A+T-N- | TIMP3 | 0.43388818 | 7.0848296 | 9.0320577 | 1.2486715e-18 | 5.9552027e-18 | 31.111757 | 818 | 543 | 275 | TRUE | FALSE | up |
| ADNI | CSF | A+T+N+ vs A+T-N- | NPTX1 | 0.39243097 | 14.7909 | 9.0142809 | 1.4468111e-18 | 6.6446141e-18 | 30.966366 | 818 | 543 | 275 | TRUE | FALSE | up |
| ADNI | CSF | A+T+N+ vs A+T-N- | UCHL1 | 0.11526192 | 11.74218 | 8.9807594 | 1.9087798e-18 | 8.4531678e-18 | 30.692838 | 818 | 543 | 275 | TRUE | FALSE | up |
| ADNI | CSF | A+T+N+ vs A+T-N- | CALB2 | 0.39733344 | 14.003267 | 8.9011366 | 3.6743349e-18 | 1.5710949e-17 | 30.046472 | 818 | 543 | 275 | TRUE | FALSE | up |
| ADNI | CSF | A+T+N+ vs A+T-N- | IL15 | 0.26705311 | 13.188067 | 8.8281319 | 6.6709713e-18 | 2.7573348e-17 | 29.457976 | 818 | 543 | 275 | TRUE | FALSE | up |
| ADNI | CSF | A+T+N+ vs A+T-N- | PSEN1 | 0.26163619 | 11.319678 | 8.7624057 | 1.137417e-17 | 4.5496679e-17 | 28.931559 | 818 | 543 | 275 | TRUE | FALSE | up |
| ADNI | CSF | A+T+N+ vs A+T-N- | VEGFA | 0.28441464 | 14.250946 | 8.5226992 | 7.7493071e-17 | 3.0028565e-16 | 27.039281 | 818 | 543 | 275 | TRUE | FALSE | up |
| ADNI | CSF | A+T+N+ vs A+T-N- | FLT1 | 0.35652892 | 11.594353 | 8.3939702 | 2.1334722e-16 | 8.0166836e-16 | 26.041112 | 818 | 543 | 275 | TRUE | FALSE | up |
| ADNI | CSF | A+T+N+ vs A+T-N- | CX3CL1 | 0.2933471 | 10.501577 | 8.2873013 | 4.8912689e-16 | 1.7838745e-15 | 25.223641 | 818 | 543 | 275 | TRUE | FALSE | up |
| ADNI | CSF | A+T+N+ vs A+T-N- | Abeta38 | 0.75788412 | 17.392251 | 8.042617 | 3.1750541e-15 | 1.1248763e-14 | 23.381815 | 818 | 543 | 275 | TRUE | TRUE | up |
| ADNI | CSF | A+T+N+ vs A+T-N- | GFAP | 0.53566289 | 17.184875 | 8.0331649 | 3.409817e-15 | 1.1744925e-14 | 23.311604 | 818 | 543 | 275 | TRUE | TRUE | up |
| ADNI | CSF | A+T+N+ vs A+T-N- | SNAP25 | 0.13395514 | 12.219855 | 7.762271 | 2.5575115e-14 | 8.5711197e-14 | 21.329366 | 818 | 543 | 275 | TRUE | FALSE | up |
| ADNI | CSF | A+T+N+ vs A+T-N- | CD63 | 0.17600861 | 9.8093839 | 7.3351884 | 5.4529699e-13 | 1.7793902e-12 | 18.323525 | 818 | 543 | 275 | TRUE | FALSE | up |
| ADNI | CSF | A+T+N+ vs A+T-N- | NPTX2 | 0.383691 | 14.414413 | 7.2837092 | 7.8081677e-13 | 2.4825969e-12 | 17.971208 | 818 | 543 | 275 | TRUE | FALSE | up |
| ADNI | CSF | A+T+N+ vs A+T-N- | NEFL | 0.3754547 | 15.977558 | 7.0584861 | 3.6626454e-12 | 1.1354201e-11 | 16.45543 | 818 | 543 | 275 | TRUE | FALSE | up |
| ADNI | CSF | A+T+N+ vs A+T-N- | CNTN2 | 0.370662 | 19.154891 | 7.0503438 | 3.870154e-12 | 1.1704856e-11 | 16.401416 | 818 | 543 | 275 | TRUE | FALSE | up |
| ADNI | CSF | A+T+N+ vs A+T-N- | VCAM1 | 0.31281949 | 8.8525887 | 6.84482 | 1.5278355e-11 | 4.5107525e-11 | 15.056315 | 818 | 543 | 275 | TRUE | FALSE | up |
| ADNI | CSF | A+T+N+ vs A+T-N- | TREM2 | 0.38735827 | 11.304029 | 6.6378391 | 5.8810957e-11 | 1.6959439e-10 | 13.737477 | 818 | 543 | 275 | TRUE | FALSE | up |
| ADNI | CSF | A+T+N+ vs A+T-N- | CRH | 0.30498403 | 10.345824 | 6.5477549 | 1.0457667e-10 | 2.9471607e-10 | 13.174797 | 818 | 543 | 275 | TRUE | FALSE | up |
| ADNI | CSF | A+T+N+ vs A+T-N- | Abeta40 | 0.68283386 | 14.920652 | 6.4990584 | 1.4234612e-10 | 3.9224265e-10 | 12.873505 | 818 | 543 | 275 | TRUE | TRUE | up |
| ADNI | CSF | A+T+N+ vs A+T-N- | IL9 | 0.30598863 | 11.427883 | 6.4681317 | 1.7296031e-10 | 4.6624084e-10 | 12.683207 | 818 | 543 | 275 | TRUE | FALSE | up |
| ADNI | CSF | A+T+N+ vs A+T-N- | IL5 | 0.30443868 | 13.076876 | 6.4003358 | 2.6435777e-10 | 6.9745454e-10 | 12.268908 | 818 | 543 | 275 | TRUE | FALSE | up |
| ADNI | CSF | A+T+N+ vs A+T-N- | NPY | 0.41175607 | 9.7875832 | 6.1723686 | 1.0702028e-09 | 2.7646906e-09 | 10.904772 | 818 | 543 | 275 | TRUE | FALSE | up |
| ADNI | CSF | A+T+N+ vs A+T-N- | MSLN | 0.35244503 | 9.0220702 | 6.1166877 | 1.495914e-09 | 3.7855784e-09 | 10.578406 | 818 | 543 | 275 | TRUE | FALSE | up |
| ADNI | CSF | A+T+N+ vs A+T-N- | SNCA | 0.41486231 | 5.659623 | 6.0514524 | 2.2072704e-09 | 5.4740306e-09 | 10.199465 | 818 | 543 | 275 | TRUE | FALSE | up |
| ADNI | CSF | A+T+N+ vs A+T-N- | SFRP1 | 0.54883229 | 13.948871 | 5.9779723 | 3.406288e-09 | 8.2819552e-09 | 9.7770706 | 818 | 543 | 275 | TRUE | TRUE | up |
| ADNI | CSF | A+T+N+ vs A+T-N- | ANXA5 | 0.42947448 | 6.5614466 | 5.769302 | 1.138926e-08 | 2.7159005e-08 | 8.6033272 | 818 | 543 | 275 | TRUE | FALSE | up |
| ADNI | CSF | A+T+N+ vs A+T-N- | S100B | 0.24513318 | 15.570143 | 5.6912077 | 1.7722464e-08 | 4.1463878e-08 | 8.1739146 | 818 | 543 | 275 | TRUE | FALSE | up |
| ADNI | CSF | A+T+N+ vs A+T-N- | TEK | 0.23949503 | 6.014503 | 5.6425156 | 2.3286501e-08 | 5.3472705e-08 | 7.9089046 | 818 | 543 | 275 | TRUE | FALSE | up |
| ADNI | CSF | A+T+N+ vs A+T-N- | TREM1 | 0.28603778 | 7.4232887 | 5.3777693 | 9.9153809e-08 | 2.2354677e-07 | 6.5049098 | 818 | 543 | 275 | TRUE | FALSE | up |
| ADNI | CSF | A+T+N+ vs A+T-N- | PTN | 0.28313536 | 10.246109 | 5.0410633 | 5.7328323e-07 | 1.2694129e-06 | 4.8101599 | 818 | 543 | 275 | TRUE | FALSE | up |
| ADNI | CSF | A+T+N+ vs A+T-N- | IL12p70 | 0.24056564 | 9.0853142 | 4.7366314 | 2.5720411e-06 | 5.5953176e-06 | 3.3664583 | 818 | 543 | 275 | TRUE | FALSE | up |
| ADNI | CSF | A+T+N+ vs A+T-N- | CCL3 | 0.20866727 | 9.3602959 | 4.6855318 | 3.2826747e-06 | 7.0181322e-06 | 3.1324476 | 818 | 543 | 275 | TRUE | FALSE | up |
| ADNI | CSF | A+T+N+ vs A+T-N- | PGK1 | 0.23709903 | 4.6833512 | 4.6359779 | 4.1496921e-06 | 8.7213867e-06 | 2.9078105 | 818 | 543 | 275 | TRUE | FALSE | up |
| ADNI | CSF | A+T+N+ vs A+T-N- | IL16 | 0.1976533 | 6.9484299 | 4.1814835 | 3.2171626e-05 | 6.6488027e-05 | 0.9535592 | 818 | 543 | 275 | TRUE | FALSE | up |
| ADNI | CSF | A+T+N+ vs A+T-N- | PDGFRB | 0.24621014 | 7.4619737 | 4.1291808 | 4.0244728e-05 | 8.1808955e-05 | 0.74101365 | 818 | 543 | 275 | TRUE | FALSE | up |
| ADNI | CSF | A+T+N+ vs A+T-N- | NGF | 0.085826412 | 6.0750331 | 4.0930961 | 4.6900351e-05 | 9.3800701e-05 | 0.59586835 | 818 | 543 | 275 | TRUE | FALSE | up |
| ADNI | CSF | A+T+N+ vs A+T-N- | CHIT1 | 0.61653067 | 9.8671255 | 3.5400733 | 0.00042320415 | 0.00083297325 | -1.4749698 | 818 | 543 | 275 | TRUE | TRUE | up |
| ADNI | CSF | A+T+N+ vs A+T-N- | ICAM1 | 0.18549005 | 5.718056 | 3.5105571 | 0.00047227528 | 0.00091503336 | -1.5773401 | 818 | 543 | 275 | TRUE | FALSE | up |
| ADNI | CSF | A+T+N+ vs A+T-N- | DDC | 0.13505453 | 6.4781118 | 3.4531975 | 0.00058319687 | 0.0011125602 | -1.7739021 | 818 | 543 | 275 | TRUE | FALSE | up |
| ADNI | CSF | A+T+N+ vs A+T-N- | POSTN | 0.26893385 | 3.8865019 | 3.413548 | 0.00067359323 | 0.0012655388 | -1.9079371 | 818 | 543 | 275 | TRUE | FALSE | up |
| ADNI | CSF | A+T+N+ vs A+T-N- | IL6R | 0.24687354 | 6.9196807 | 3.2749097 | 0.0011025785 | 0.002040593 | -2.3647747 | 818 | 543 | 275 | TRUE | FALSE | up |
| ADNI | CSF | A+T+N+ vs A+T-N- | TNF | 0.1176857 | 8.524919 | 3.1992365 | 0.0014324055 | 0.0026120335 | -2.6063537 | 818 | 543 | 275 | TRUE | FALSE | up |
| ADNI | CSF | A+T+N+ vs A+T-N- | VEGFD | 0.083076843 | 10.520108 | 2.9917824 | 0.0028590356 | 0.0051379771 | -3.2403649 | 818 | 543 | 275 | TRUE | FALSE | up |
| ADNI | CSF | A+T+N+ vs A+T-N- | CSF2 | 0.11051879 | 11.934631 | 2.6980557 | 0.0071220325 | 0.012616172 | -4.0668859 | 818 | 543 | 275 | TRUE | FALSE | up |
| ADNI | CSF | A+T+N+ vs A+T-N- | CCL2 | 0.098831921 | 11.469945 | 2.4942647 | 0.012823307 | 0.022395634 | -4.5910836 | 818 | 543 | 275 | TRUE | FALSE | up |
| ADNI | CSF | A+T+N+ vs A+T-N- | CXCL1 | 0.099854697 | 9.8123122 | 2.431807 | 0.015242946 | 0.026251741 | -4.7436311 | 818 | 543 | 275 | TRUE | FALSE | up |
| ADNI | CSF | A+T+N+ vs A+T-N- | SFTPD | 0.21237854 | 7.6277759 | 2.2390966 | 0.025424719 | 0.043187194 | -5.1902651 | 818 | 543 | 275 | TRUE | FALSE | up |
| ADNI | CSF | A+T+N+ vs A+T-N- | RUVBL2 | -0.057097493 | 10.058469 | -2.0553064 | 0.040174689 | 0.06731975 | -5.582304 | 818 | 543 | 275 | FALSE | FALSE | down |
| ADNI | CSF | A+T+N+ vs A+T-N- | CXCL8 | 0.076937095 | 12.995217 | 2.0216374 | 0.043547455 | 0.071051111 | -5.6505253 | 818 | 543 | 275 | FALSE | FALSE | up |
| ADNI | CSF | A+T+N+ vs A+T-N- | MME | -0.050799167 | 0.22254375 | -2.0222207 | 0.043487045 | 0.071051111 | -5.6493529 | 818 | 543 | 275 | FALSE | FALSE | down |
| ADNI | CSF | A+T+N+ vs A+T-N- | SAA1 | -0.11485591 | 0.21289391 | -1.9832094 | 0.047686774 | 0.076794285 | -5.7270264 | 818 | 543 | 275 | FALSE | FALSE | down |
| ADNI | CSF | A+T+N+ vs A+T-N- | IL2 | 0.082513354 | 6.1441176 | 1.9000674 | 0.057784834 | 0.091863069 | -5.8875663 | 818 | 543 | 275 | FALSE | FALSE | up |
| ADNI | CSF | A+T+N+ vs A+T-N- | YWHAZ | 0.021150152 | 11.640563 | 1.8248894 | 0.068391793 | 0.10734914 | -6.0268626 | 818 | 543 | 275 | FALSE | FALSE | up |
| ADNI | CSF | A+T+N+ vs A+T-N- | S100A12 | -0.17624085 | 4.0463005 | -1.7076049 | 0.088099012 | 0.13655347 | -6.2330389 | 818 | 543 | 275 | FALSE | FALSE | down |
| ADNI | CSF | A+T+N+ vs A+T-N- | UBB | 0.014031341 | 11.110319 | 1.67045 | 0.095222863 | 0.14577327 | -6.2955195 | 818 | 543 | 275 | FALSE | FALSE | up |
| ADNI | CSF | A+T+N+ vs A+T-N- | CXCL10 | 0.11850507 | 9.3581092 | 1.5586539 | 0.11947517 | 0.18066977 | -6.4752842 | 818 | 543 | 275 | FALSE | FALSE | up |
| ADNI | CSF | A+T+N+ vs A+T-N- | PARK7 | 0.051126441 | 10.856021 | 1.4972525 | 0.13472337 | 0.20127346 | -6.5687523 | 818 | 543 | 275 | FALSE | FALSE | up |
| ADNI | CSF | A+T+N+ vs A+T-N- | GDF15 | 0.18417299 | 5.75341 | 1.4532579 | 0.14654567 | 0.21632932 | -6.633426 | 818 | 543 | 275 | FALSE | FALSE | up |
| ADNI | CSF | A+T+N+ vs A+T-N- | CCL4 | 0.084143153 | 8.4661978 | 1.4315874 | 0.15265384 | 0.22269501 | -6.6645772 | 818 | 543 | 275 | FALSE | FALSE | up |
| ADNI | CSF | A+T+N+ vs A+T-N- | CCL11 | 0.054409397 | 8.8800567 | 1.402578 | 0.161132 | 0.23232986 | -6.7055492 | 818 | 543 | 275 | FALSE | FALSE | up |
| ADNI | CSF | A+T+N+ vs A+T-N- | FCN2 | -0.10024958 | 1.2015225 | -1.3589217 | 0.17455572 | 0.24879205 | -6.7656348 | 818 | 543 | 275 | FALSE | FALSE | down |
| ADNI | CSF | A+T+N+ vs A+T-N- | IL10 | 0.080345433 | 9.3888438 | 1.3061968 | 0.19186235 | 0.2703515 | -6.8356805 | 818 | 543 | 275 | FALSE | FALSE | up |
| ADNI | CSF | A+T+N+ vs A+T-N- | HTT | 0.095948611 | 6.8008624 | 1.2839443 | 0.19953445 | 0.27800306 | -6.8644151 | 818 | 543 | 275 | FALSE | FALSE | up |
| ADNI | CSF | A+T+N+ vs A+T-N- | CCL13 | 0.088214522 | 4.7494364 | 1.2758568 | 0.20237773 | 0.27883154 | -6.8747367 | 818 | 543 | 275 | FALSE | FALSE | up |
| ADNI | CSF | A+T+N+ vs A+T-N- | IL13 | 0.05095321 | 9.6176008 | 1.2607499 | 0.2077678 | 0.28051528 | -6.8938426 | 818 | 543 | 275 | FALSE | FALSE | up |
| ADNI | CSF | A+T+N+ vs A+T-N- | TARDBP | -0.071447859 | 5.9015382 | -1.259761 | 0.20812424 | 0.28051528 | -6.8950854 | 818 | 543 | 275 | FALSE | FALSE | down |
| ADNI | CSF | A+T+N+ vs A+T-N- | VGF | -0.028312105 | 9.9789349 | -1.231665 | 0.2184374 | 0.29124987 | -6.9299885 | 818 | 543 | 275 | FALSE | FALSE | down |
| ADNI | CSF | A+T+N+ vs A+T-N- | NEFH | 0.062796616 | 21.255294 | 1.2250282 | 0.22092642 | 0.29143486 | -6.9381189 | 818 | 543 | 275 | FALSE | FALSE | up |
| ADNI | CSF | A+T+N+ vs A+T-N- | CCL26 | 0.082675154 | 5.7561971 | 1.2025656 | 0.22950149 | 0.29955984 | -6.9653114 | 818 | 543 | 275 | FALSE | FALSE | up |
| ADNI | CSF | A+T+N+ vs A+T-N- | pSNCA-129 | 0.035664896 | 8.6622333 | 1.0847652 | 0.27835357 | 0.35954002 | -7.0997049 | 818 | 543 | 275 | FALSE | FALSE | up |
| ADNI | CSF | A+T+N+ vs A+T-N- | BDNF | -0.101219 | 0.81613132 | -1.0076351 | 0.3139354 | 0.40131949 | -7.1802222 | 818 | 543 | 275 | FALSE | FALSE | down |
| ADNI | CSF | A+T+N+ vs A+T-N- | KDR | 0.13740735 | 6.7683888 | 0.97798722 | 0.32837731 | 0.41549782 | -7.2095967 | 818 | 543 | 275 | FALSE | FALSE | up |
| ADNI | CSF | A+T+N+ vs A+T-N- | ARSA | -0.059630484 | 6.0197095 | -0.91502373 | 0.36045584 | 0.45148004 | -7.2690766 | 818 | 543 | 275 | FALSE | FALSE | down |
| ADNI | CSF | A+T+N+ vs A+T-N- | CCL17 | 0.054499998 | 4.2766913 | 0.90545554 | 0.36549765 | 0.45321709 | -7.2777698 | 818 | 543 | 275 | FALSE | FALSE | up |
| ADNI | CSF | A+T+N+ vs A+T-N- | IL7 | -0.035334754 | 10.323315 | -0.8453173 | 0.39818732 | 0.48886364 | -7.3303207 | 818 | 543 | 275 | FALSE | FALSE | down |
| ADNI | CSF | A+T+N+ vs A+T-N- | CCL22 | 0.079906999 | 2.7979158 | 0.81274427 | 0.41660733 | 0.50154668 | -7.3572796 | 818 | 543 | 275 | FALSE | FALSE | up |
| ADNI | CSF | A+T+N+ vs A+T-N- | IL6 | -0.031781037 | 12.344746 | -0.81744301 | 0.41391953 | 0.50154668 | -7.353456 | 818 | 543 | 275 | FALSE | FALSE | down |
| ADNI | CSF | A+T+N+ vs A+T-N- | Abeta42 | 0.079815853 | 14.307892 | 0.80338464 | 0.4219919 | 0.50314419 | -7.3648304 | 818 | 543 | 275 | FALSE | FALSE | up |
| ADNI | CSF | A+T+N+ vs A+T-N- | CD40LG | -0.066866764 | 4.926071 | -0.74796966 | 0.45469903 | 0.53191207 | -7.4077472 | 818 | 543 | 275 | FALSE | FALSE | down |
| ADNI | CSF | A+T+N+ vs A+T-N- | IL33 | 0.0299864 | 9.3065525 | 0.75350767 | 0.45136737 | 0.53191207 | -7.403596 | 818 | 543 | 275 | FALSE | FALSE | up |
| ADNI | CSF | A+T+N+ vs A+T-N- | IL17A | 0.012779683 | 9.1561453 | 0.73037433 | 0.46537597 | 0.53850254 | -7.4207338 | 818 | 543 | 275 | FALSE | FALSE | up |
| ADNI | CSF | A+T+N+ vs A+T-N- | IL4 | -0.038915868 | 8.424355 | -0.72442345 | 0.46901834 | 0.53850254 | -7.425056 | 818 | 543 | 275 | FALSE | FALSE | down |
| ADNI | CSF | A+T+N+ vs A+T-N- | pTDP43-409 | -0.0090124382 | 9.6256322 | -0.71009953 | 0.47785007 | 0.54360925 | -7.4353151 | 818 | 543 | 275 | FALSE | FALSE | down |
| ADNI | CSF | A+T+N+ vs A+T-N- | PDLIM5 | -0.017886747 | 9.3684439 | -0.69199915 | 0.48913935 | 0.55139344 | -7.4479862 | 818 | 543 | 275 | FALSE | FALSE | down |
| ADNI | CSF | A+T+N+ vs A+T-N- | CST3 | 0.0062385156 | 13.767921 | 0.59059819 | 0.55495706 | 0.61995203 | -7.5129281 | 818 | 543 | 275 | FALSE | FALSE | up |
| ADNI | CSF | A+T+N+ vs A+T-N- | IL1B | -0.010638967 | 10.304916 | -0.52348328 | 0.60078346 | 0.66515311 | -7.5502689 | 818 | 543 | 275 | FALSE | FALSE | down |
| ADNI | CSF | A+T+N+ vs A+T-N- | HBA1 | -0.15071706 | 4.4833782 | -0.44448077 | 0.65681566 | 0.72075347 | -7.5884624 | 818 | 543 | 275 | FALSE | FALSE | down |
| ADNI | CSF | A+T+N+ vs A+T-N- | IFNG | 0.025426912 | 8.1222694 | 0.39675461 | 0.69165461 | 0.75232606 | -7.6085161 | 818 | 543 | 275 | FALSE | FALSE | up |
| ADNI | CSF | A+T+N+ vs A+T-N- | GDNF | 0.0039521893 | 8.8453549 | 0.35269019 | 0.72441394 | 0.7811072 | -7.6250117 | 818 | 543 | 275 | FALSE | FALSE | up |
| ADNI | CSF | A+T+N+ vs A+T-N- | Oligo-SNCA | -0.01499968 | 5.5069397 | -0.20791815 | 0.83534598 | 0.88532394 | -7.6655548 | 818 | 543 | 275 | FALSE | FALSE | down |
| ADNI | CSF | A+T+N+ vs A+T-N- | PGF | 0.0091971305 | 14.68933 | 0.20901235 | 0.83449199 | 0.88532394 | -7.6653269 | 818 | 543 | 275 | FALSE | FALSE | up |
| ADNI | CSF | A+T+N+ vs A+T-N- | PRDX6 | -0.019443648 | 8.128311 | -0.18644718 | 0.85214153 | 0.89547076 | -7.6697848 | 818 | 543 | 275 | FALSE | FALSE | down |
| ADNI | CSF | A+T+N+ vs A+T-N- | FOLR1 | 0.0037828661 | 12.386612 | 0.15731236 | 0.87503851 | 0.91180484 | -7.6747883 | 818 | 543 | 275 | FALSE | FALSE | up |
| ADNI | CSF | A+T+N+ vs A+T-N- | BASP1 | -0.0016975796 | 10.146599 | -0.13011607 | 0.89650742 | 0.926391 | -7.6786935 | 818 | 543 | 275 | FALSE | FALSE | down |
| ADNI | CSF | A+T+N+ vs A+T-N- | REST | 0.0040380892 | 8.09634 | 0.10284198 | 0.91811425 | 0.94087741 | -7.6818677 | 818 | 543 | 275 | FALSE | FALSE | up |
| ADNI | CSF | A+T+N+ vs A+T-N- | FGF2 | -0.0058835424 | 9.8491393 | -0.073668964 | 0.94129227 | 0.94820044 | -7.6844403 | 818 | 543 | 275 | FALSE | FALSE | down |
| ADNI | CSF | A+T+N+ vs A+T-N- | IGFBP7 | -0.0011853141 | 13.937726 | -0.064987261 | 0.94820044 | 0.94820044 | -7.6850417 | 818 | 543 | 275 | FALSE | FALSE | down |
| ADNI | CSF | A+T+N+ vs A+T-N- | IL18 | 0.0033699402 | 6.0557595 | 0.069618243 | 0.94451497 | 0.94820044 | -7.6847303 | 818 | 543 | 275 | FALSE | FALSE | up |

**ADNI | plasma | T+ vs T-**

| **Cohort** | **Fluid** | **Contrast** | **Protein** | **log₂FC** | **AveExpr** | **t** | **P.Value** | **FDR** | **B** | **N_total** | **N_group1** | **N_group2** | **Pass_FDR** | **Pass_both_thresholds** | **Direction** |
| --- | --- | --- | --- | --- | --- | --- | --- | --- | --- | --- | --- | --- | --- | --- | --- |
| ADNI | plasma | T+ vs T- | pTau-217 | 0.63398885 | 11.899171 | 10.787604 | 2.2713353e-25 | 2.8618825e-23 | 46.773277 | 793 | 531 | 262 | TRUE | TRUE | up |
| ADNI | plasma | T+ vs T- | pTau-231 | 0.42131823 | 13.812086 | 8.116288 | 1.889888e-15 | 1.1906295e-13 | 24.307159 | 793 | 531 | 262 | TRUE | FALSE | up |
| ADNI | plasma | T+ vs T- | pTau-181 | 0.34559519 | 13.532756 | 6.8300998 | 1.718693e-11 | 7.2185105e-10 | 15.394799 | 793 | 531 | 262 | TRUE | FALSE | up |
| ADNI | plasma | T+ vs T- | GFAP | 0.26714672 | 13.336909 | 5.1056169 | 4.1551647e-07 | 1.0471015e-05 | 5.6160459 | 793 | 531 | 262 | TRUE | FALSE | up |
| ADNI | plasma | T+ vs T- | MAPT | 0.222673 | 12.164537 | 5.1393721 | 3.4944334e-07 | 1.0471015e-05 | 5.7824266 | 793 | 531 | 262 | TRUE | FALSE | up |
| ADNI | plasma | T+ vs T- | ACHE | 0.24055226 | 13.408239 | 3.7295614 | 0.00020583463 | 0.0043225272 | -0.27976672 | 793 | 531 | 262 | TRUE | FALSE | up |
| ADNI | plasma | T+ vs T- | NGF | 0.10647367 | 8.7824569 | 3.0189012 | 0.0026203446 | 0.047166202 | -2.6307475 | 793 | 531 | 262 | TRUE | FALSE | up |
| ADNI | plasma | T+ vs T- | BACE1 | 0.055415203 | 13.485286 | 2.3476613 | 0.019142969 | 0.30150176 | -4.4061672 | 793 | 531 | 262 | FALSE | FALSE | up |
| ADNI | plasma | T+ vs T- | IGF1R | 0.038927304 | 12.240122 | 2.301576 | 0.021624201 | 0.30273882 | -4.5120292 | 793 | 531 | 262 | FALSE | FALSE | up |
| ADNI | plasma | T+ vs T- | VSNL1 | 0.069139839 | 12.281453 | 2.159875 | 0.031089154 | 0.39172334 | -4.824553 | 793 | 531 | 262 | FALSE | FALSE | up |
| ADNI | plasma | T+ vs T- | NEFL | 0.087203827 | 11.679451 | 2.0792814 | 0.037920634 | 0.43436363 | -4.9935533 | 793 | 531 | 262 | FALSE | FALSE | up |
| ADNI | plasma | T+ vs T- | CCL11 | 0.080950998 | 13.718093 | 1.8313326 | 0.067435654 | 0.55003753 | -5.4735998 | 793 | 531 | 262 | FALSE | FALSE | up |
| ADNI | plasma | T+ vs T- | CNTN2 | -0.18474006 | 7.8125734 | -1.8154248 | 0.069846035 | 0.55003753 | -5.5023396 | 793 | 531 | 262 | FALSE | FALSE | down |
| ADNI | plasma | T+ vs T- | NPTX2 | 0.05392342 | 13.347348 | 1.8681813 | 0.062114934 | 0.55003753 | -5.4060728 | 793 | 531 | 262 | FALSE | FALSE | up |
| ADNI | plasma | T+ vs T- | NPTXR | -0.071481434 | 10.989891 | -1.8408462 | 0.066027201 | 0.55003753 | -5.4562934 | 793 | 531 | 262 | FALSE | FALSE | down |
| ADNI | plasma | T+ vs T- | VGF | -0.28785798 | 12.611345 | -1.8847848 | 0.059833834 | 0.55003753 | -5.3752105 | 793 | 531 | 262 | FALSE | FALSE | down |
| ADNI | plasma | T+ vs T- | TREM1 | 0.058736258 | 11.39001 | 1.6119851 | 0.10737339 | 0.79582629 | -5.8479444 | 793 | 531 | 262 | FALSE | FALSE | up |
| ADNI | plasma | T+ vs T- | APOE | -0.065300162 | 13.225898 | -1.451513 | 0.14704279 | 0.88225675 | -6.0917984 | 793 | 531 | 262 | FALSE | FALSE | down |
| ADNI | plasma | T+ vs T- | CXCL10 | 0.084303732 | 11.271722 | 1.4630555 | 0.14385868 | 0.88225675 | -6.0751061 | 793 | 531 | 262 | FALSE | FALSE | up |
| ADNI | plasma | T+ vs T- | KLK6 | -0.051229174 | 12.216801 | -1.4627524 | 0.14394162 | 0.88225675 | -6.0755462 | 793 | 531 | 262 | FALSE | FALSE | down |
| ADNI | plasma | T+ vs T- | VCAM1 | 0.035217905 | 13.447396 | 1.4809986 | 0.13901445 | 0.88225675 | -6.0488966 | 793 | 531 | 262 | FALSE | FALSE | up |
| ADNI | plasma | T+ vs T- | Abeta42 | -0.061330978 | 12.416167 | -1.3154714 | 0.18874156 | 0.88375141 | -6.2786238 | 793 | 531 | 262 | FALSE | FALSE | down |
| ADNI | plasma | T+ vs T- | BASP1 | -0.19480845 | 11.893332 | -1.2607808 | 0.20776825 | 0.88375141 | -6.3485766 | 793 | 531 | 262 | FALSE | FALSE | down |
| ADNI | plasma | T+ vs T- | CCL13 | 0.062711065 | 13.170055 | 1.149979 | 0.2505082 | 0.88375141 | -6.4812304 | 793 | 531 | 262 | FALSE | FALSE | up |
| ADNI | plasma | T+ vs T- | CD63 | -0.042726873 | 12.307859 | -1.149342 | 0.25077048 | 0.88375141 | -6.4819578 | 793 | 531 | 262 | FALSE | FALSE | down |
| ADNI | plasma | T+ vs T- | CHI3L1 | 0.099023216 | 13.307362 | 1.2593047 | 0.20830043 | 0.88375141 | -6.3504237 | 793 | 531 | 262 | FALSE | FALSE | up |
| ADNI | plasma | T+ vs T- | CHIT1 | 0.2648266 | 13.689934 | 1.3409695 | 0.18032427 | 0.88375141 | -6.2449997 | 793 | 531 | 262 | FALSE | FALSE | up |
| ADNI | plasma | T+ vs T- | CSF2 | -0.088049901 | 14.748262 | -1.3115911 | 0.19004755 | 0.88375141 | -6.2836844 | 793 | 531 | 262 | FALSE | FALSE | down |
| ADNI | plasma | T+ vs T- | GOT1 | 0.044959108 | 11.648811 | 1.1451526 | 0.2525004 | 0.88375141 | -6.4867325 | 793 | 531 | 262 | FALSE | FALSE | up |
| ADNI | plasma | T+ vs T- | IL10 | 0.092140837 | 12.311109 | 1.3637785 | 0.17303429 | 0.88375141 | -6.2143771 | 793 | 531 | 262 | FALSE | FALSE | up |
| ADNI | plasma | T+ vs T- | IL6 | -0.068446372 | 12.894965 | -1.4127985 | 0.1581175 | 0.88375141 | -6.1468256 | 793 | 531 | 262 | FALSE | FALSE | down |
| ADNI | plasma | T+ vs T- | IL9 | 0.084768309 | 10.509838 | 1.202249 | 0.22963524 | 0.88375141 | -6.4201655 | 793 | 531 | 262 | FALSE | FALSE | up |
| ADNI | plasma | T+ vs T- | PDGFRB | 0.063356477 | 12.915791 | 1.2048107 | 0.22864514 | 0.88375141 | -6.4171033 | 793 | 531 | 262 | FALSE | FALSE | up |
| ADNI | plasma | T+ vs T- | SFTPD | 0.075658934 | 13.313642 | 1.1774392 | 0.23938265 | 0.88375141 | -6.4494868 | 793 | 531 | 262 | FALSE | FALSE | up |
| ADNI | plasma | T+ vs T- | UBB | 0.15331349 | 12.305531 | 1.2194329 | 0.22305188 | 0.88375141 | -6.3994997 | 793 | 531 | 262 | FALSE | FALSE | up |
| ADNI | plasma | T+ vs T- | YWHAG | -0.037040865 | 11.87825 | -1.218207 | 0.223517 | 0.88375141 | -6.4009836 | 793 | 531 | 262 | FALSE | FALSE | down |
| ADNI | plasma | T+ vs T- | CCL4 | 0.086708987 | 11.815711 | 1.1129327 | 0.2660833 | 0.89881727 | -6.5228721 | 793 | 531 | 262 | FALSE | FALSE | up |
| ADNI | plasma | T+ vs T- | IL2 | 0.050515901 | 10.651703 | 1.0851233 | 0.27820534 | 0.89881727 | -6.5532382 | 793 | 531 | 262 | FALSE | FALSE | up |
| ADNI | plasma | T+ vs T- | S100A12 | 0.12099604 | 12.59537 | 1.0888332 | 0.2765668 | 0.89881727 | -6.5492316 | 793 | 531 | 262 | FALSE | FALSE | up |
| ADNI | plasma | T+ vs T- | CRP | -0.11645335 | 11.451022 | -1.0351396 | 0.30092745 | 0.90350202 | -6.6058913 | 793 | 531 | 262 | FALSE | FALSE | down |
| ADNI | plasma | T+ vs T- | IL15 | 0.026374707 | 13.022508 | 1.0417851 | 0.29783703 | 0.90350202 | -6.5990336 | 793 | 531 | 262 | FALSE | FALSE | up |
| ADNI | plasma | T+ vs T- | PGF | 0.034556949 | 13.649756 | 1.0346257 | 0.30116734 | 0.90350202 | -6.6064199 | 793 | 531 | 262 | FALSE | FALSE | up |
| ADNI | plasma | T+ vs T- | CCL2 | 0.048714781 | 10.207694 | 0.89113167 | 0.37313583 | 0.90413682 | -6.7437448 | 793 | 531 | 262 | FALSE | FALSE | up |
| ADNI | plasma | T+ vs T- | CX3CL1 | -0.026467272 | 13.376408 | -0.94382637 | 0.34555329 | 0.90413682 | -6.6956877 | 793 | 531 | 262 | FALSE | FALSE | down |
| ADNI | plasma | T+ vs T- | FOLR1 | -0.032883366 | 10.309816 | -0.99972835 | 0.31775484 | 0.90413682 | -6.6416955 | 793 | 531 | 262 | FALSE | FALSE | down |
| ADNI | plasma | T+ vs T- | ICAM1 | 0.028937068 | 12.878742 | 0.9691485 | 0.33277424 | 0.90413682 | -6.6716145 | 793 | 531 | 262 | FALSE | FALSE | up |
| ADNI | plasma | T+ vs T- | PARK7 | -0.1141461 | 13.180652 | -0.97449062 | 0.33011785 | 0.90413682 | -6.6664547 | 793 | 531 | 262 | FALSE | FALSE | down |
| ADNI | plasma | T+ vs T- | PTN | 0.061985769 | 6.868528 | 0.93853677 | 0.3482618 | 0.90413682 | -6.7006361 | 793 | 531 | 262 | FALSE | FALSE | up |
| ADNI | plasma | T+ vs T- | SFRP1 | 0.073102877 | 15.219506 | 0.90489056 | 0.36580532 | 0.90413682 | -6.7314625 | 793 | 531 | 262 | FALSE | FALSE | up |
| ADNI | plasma | T+ vs T- | TEK | -0.023812077 | 12.806769 | -0.92006772 | 0.35782441 | 0.90413682 | -6.7176963 | 793 | 531 | 262 | FALSE | FALSE | down |
| ADNI | plasma | T+ vs T- | VEGFD | -0.066062889 | 12.264268 | -0.90340237 | 0.36659383 | 0.90413682 | -6.7328001 | 793 | 531 | 262 | FALSE | FALSE | down |
| ADNI | plasma | T+ vs T- | YWHAZ | 0.029051485 | 11.811358 | 0.99979201 | 0.31772404 | 0.90413682 | -6.6416322 | 793 | 531 | 262 | FALSE | FALSE | up |
| ADNI | plasma | T+ vs T- | AGRN | 0.022199902 | 13.205262 | 0.84561933 | 0.39802682 | 0.91184327 | -6.7830348 | 793 | 531 | 262 | FALSE | FALSE | up |
| ADNI | plasma | T+ vs T- | CD40LG | -0.11416949 | 12.846874 | -0.86102393 | 0.38949186 | 0.91184327 | -6.7699664 | 793 | 531 | 262 | FALSE | FALSE | down |
| ADNI | plasma | T+ vs T- | IL1B | -0.044687224 | 10.691047 | -0.85807235 | 0.3911185 | 0.91184327 | -6.7724886 | 793 | 531 | 262 | FALSE | FALSE | down |
| ADNI | plasma | T+ vs T- | CRH | 0.063577018 | 12.22954 | 0.80434402 | 0.42144563 | 0.94447116 | -6.8168896 | 793 | 531 | 262 | FALSE | FALSE | up |
| ADNI | plasma | T+ vs T- | FABP3 | -0.02944264 | 13.154646 | -0.77628905 | 0.4378158 | 0.94447116 | -6.8389356 | 793 | 531 | 262 | FALSE | FALSE | down |
| ADNI | plasma | T+ vs T- | FLT1 | 0.02538197 | 13.463483 | 0.76879075 | 0.44225237 | 0.94447116 | -6.8446955 | 793 | 531 | 262 | FALSE | FALSE | up |
| ADNI | plasma | T+ vs T- | SNCB | -0.020455306 | 10.836801 | -0.7782066 | 0.43668537 | 0.94447116 | -6.8374536 | 793 | 531 | 262 | FALSE | FALSE | down |
| ADNI | plasma | T+ vs T- | MSLN | -0.050973914 | 13.507774 | -0.73328626 | 0.46360618 | 0.95895736 | -6.8712108 | 793 | 531 | 262 | FALSE | FALSE | down |
| ADNI | plasma | T+ vs T- | SMOC1 | 0.072698741 | 12.760329 | 0.73221855 | 0.46425714 | 0.95895736 | -6.8719889 | 793 | 531 | 262 | FALSE | FALSE | up |
| ADNI | plasma | T+ vs T- | NPY | 0.073365416 | 14.99324 | 0.70833667 | 0.47894976 | 0.97334951 | -6.8890948 | 793 | 531 | 262 | FALSE | FALSE | up |
| ADNI | plasma | T+ vs T- | ARSA | -0.065069306 | 11.224533 | -0.56632568 | 0.57133679 | 0.97483637 | -6.979112 | 793 | 531 | 262 | FALSE | FALSE | down |
| ADNI | plasma | T+ vs T- | GDF15 | 0.10818508 | 12.19832 | 0.5791995 | 0.56262331 | 0.97483637 | -6.9717775 | 793 | 531 | 262 | FALSE | FALSE | up |
| ADNI | plasma | T+ vs T- | HBA1 | 0.091761229 | 6.9467986 | 0.61080346 | 0.54150926 | 0.97483637 | -6.9530737 | 793 | 531 | 262 | FALSE | FALSE | up |
| ADNI | plasma | T+ vs T- | IL12p70 | -0.044665002 | 13.465644 | -0.64253605 | 0.52071572 | 0.97483637 | -6.9332952 | 793 | 531 | 262 | FALSE | FALSE | down |
| ADNI | plasma | T+ vs T- | IL4 | 0.040444971 | 10.919791 | 0.61428357 | 0.53920871 | 0.97483637 | -6.9509535 | 793 | 531 | 262 | FALSE | FALSE | up |
| ADNI | plasma | T+ vs T- | IL6R | -0.022842125 | 12.068669 | -0.61109268 | 0.54131788 | 0.97483637 | -6.952898 | 793 | 531 | 262 | FALSE | FALSE | down |
| ADNI | plasma | T+ vs T- | KDR | 0.094334967 | 12.299125 | 0.6674997 | 0.50465223 | 0.97483637 | -6.9170326 | 793 | 531 | 262 | FALSE | FALSE | up |
| ADNI | plasma | T+ vs T- | SLIT2 | -0.020466265 | 13.230064 | -0.56458047 | 0.57252295 | 0.97483637 | -6.9800936 | 793 | 531 | 262 | FALSE | FALSE | down |
| ADNI | plasma | T+ vs T- | SQSTM1 | 0.037184899 | 11.626986 | 0.57910755 | 0.56268533 | 0.97483637 | -6.9718305 | 793 | 531 | 262 | FALSE | FALSE | up |
| ADNI | plasma | T+ vs T- | TIMP3 | 0.069048975 | 14.730706 | 0.5685028 | 0.56985872 | 0.97483637 | -6.9778832 | 793 | 531 | 262 | FALSE | FALSE | up |
| ADNI | plasma | T+ vs T- | TNF | 0.022415116 | 13.354033 | 0.62075726 | 0.53494233 | 0.97483637 | -6.9469773 | 793 | 531 | 262 | FALSE | FALSE | up |
| ADNI | plasma | T+ vs T- | VEGFA | -0.019741412 | 13.730398 | -0.6607113 | 0.50899433 | 0.97483637 | -6.9215162 | 793 | 531 | 262 | FALSE | FALSE | down |
| ADNI | plasma | T+ vs T- | Abeta38 | 0.034268262 | 9.5709062 | 0.52854435 | 0.59727329 | 0.99021625 | -6.9996856 | 793 | 531 | 262 | FALSE | FALSE | up |
| ADNI | plasma | T+ vs T- | CXCL8 | 0.034279421 | 10.260772 | 0.53765066 | 0.590973 | 0.99021625 | -6.9948566 | 793 | 531 | 262 | FALSE | FALSE | up |
| ADNI | plasma | T+ vs T- | Abeta40 | 0.010284217 | 10.912733 | 0.1710879 | 0.86419942 | 0.99276472 | -7.1240932 | 793 | 531 | 262 | FALSE | FALSE | up |
| ADNI | plasma | T+ vs T- | BDNF | 0.0062427636 | 13.70788 | 0.054562261 | 0.9565013 | 0.99276472 | -7.1371756 | 793 | 531 | 262 | FALSE | FALSE | up |
| ADNI | plasma | T+ vs T- | CCL17 | 0.037657566 | 13.55906 | 0.26087781 | 0.79425623 | 0.99276472 | -7.1047961 | 793 | 531 | 262 | FALSE | FALSE | up |
| ADNI | plasma | T+ vs T- | CCL22 | -0.0087585563 | 12.839852 | -0.20126223 | 0.84054648 | 0.99276472 | -7.1185032 | 793 | 531 | 262 | FALSE | FALSE | down |
| ADNI | plasma | T+ vs T- | CCL26 | 0.025147555 | 11.46364 | 0.33809676 | 0.73538208 | 0.99276472 | -7.0817859 | 793 | 531 | 262 | FALSE | FALSE | up |
| ADNI | plasma | T+ vs T- | CCL3 | -0.019424038 | 11.789254 | -0.29985217 | 0.76437053 | 0.99276472 | -7.0939236 | 793 | 531 | 262 | FALSE | FALSE | down |
| ADNI | plasma | T+ vs T- | CST3 | -0.0014022766 | 13.312248 | -0.073223533 | 0.94164718 | 0.99276472 | -7.1359891 | 793 | 531 | 262 | FALSE | FALSE | down |
| ADNI | plasma | T+ vs T- | CXCL1 | 0.033804465 | 12.008193 | 0.34414904 | 0.7308278 | 0.99276472 | -7.0797317 | 793 | 531 | 262 | FALSE | FALSE | up |
| ADNI | plasma | T+ vs T- | DDC | 0.0036166633 | 11.436432 | 0.074329903 | 0.94076712 | 0.99276472 | -7.1359079 | 793 | 531 | 262 | FALSE | FALSE | up |
| ADNI | plasma | T+ vs T- | ENO2 | -0.00368937 | 12.823457 | -0.048602816 | 0.96124838 | 0.99276472 | -7.1374815 | 793 | 531 | 262 | FALSE | FALSE | down |
| ADNI | plasma | T+ vs T- | FCN2 | -0.0057363573 | 12.455908 | -0.11309196 | 0.90998701 | 0.99276472 | -7.1322933 | 793 | 531 | 262 | FALSE | FALSE | down |
| ADNI | plasma | T+ vs T- | FGF2 | -0.030124542 | 13.541512 | -0.2123907 | 0.83185824 | 0.99276472 | -7.1162129 | 793 | 531 | 262 | FALSE | FALSE | down |
| ADNI | plasma | T+ vs T- | GDI1 | -0.029244203 | 8.8744837 | -0.45717046 | 0.64767699 | 0.99276472 | -7.0346796 | 793 | 531 | 262 | FALSE | FALSE | down |
| ADNI | plasma | T+ vs T- | GDNF | -0.02730733 | 11.262178 | -0.17639872 | 0.86002691 | 0.99276472 | -7.123175 | 793 | 531 | 262 | FALSE | FALSE | down |
| ADNI | plasma | T+ vs T- | HTT | -0.03951376 | 13.850602 | -0.30017779 | 0.76412225 | 0.99276472 | -7.0938264 | 793 | 531 | 262 | FALSE | FALSE | down |
| ADNI | plasma | T+ vs T- | IFNG | -0.010283 | 12.569214 | -0.12513429 | 0.90044977 | 0.99276472 | -7.1308659 | 793 | 531 | 262 | FALSE | FALSE | down |
| ADNI | plasma | T+ vs T- | IGFBP7 | 0.0060385528 | 13.112446 | 0.29381133 | 0.76898095 | 0.99276472 | -7.0957078 | 793 | 531 | 262 | FALSE | FALSE | up |
| ADNI | plasma | T+ vs T- | IL13 | -0.028937497 | 13.107169 | -0.2609491 | 0.79420126 | 0.99276472 | -7.1047776 | 793 | 531 | 262 | FALSE | FALSE | down |
| ADNI | plasma | T+ vs T- | IL16 | -0.013132456 | 12.787441 | -0.3047302 | 0.76065367 | 0.99276472 | -7.0924564 | 793 | 531 | 262 | FALSE | FALSE | down |
| ADNI | plasma | T+ vs T- | IL17A | -0.017649496 | 11.220003 | -0.15953238 | 0.87329112 | 0.99276472 | -7.125994 | 793 | 531 | 262 | FALSE | FALSE | down |
| ADNI | plasma | T+ vs T- | IL18 | -0.002271803 | 13.050907 | -0.050767421 | 0.95952397 | 0.99276472 | -7.1373745 | 793 | 531 | 262 | FALSE | FALSE | down |
| ADNI | plasma | T+ vs T- | IL33 | 0.0089074494 | 12.151655 | 0.22524443 | 0.82184866 | 0.99276472 | -7.1134142 | 793 | 531 | 262 | FALSE | FALSE | up |
| ADNI | plasma | T+ vs T- | IL5 | -0.037907478 | 13.121425 | -0.42727987 | 0.66929449 | 0.99276472 | -7.04783 | 793 | 531 | 262 | FALSE | FALSE | down |
| ADNI | plasma | T+ vs T- | IL7 | 0.036099148 | 13.085417 | 0.45093725 | 0.65216124 | 0.99276472 | -7.0374952 | 793 | 531 | 262 | FALSE | FALSE | up |
| ADNI | plasma | T+ vs T- | MDH1 | -0.041136959 | 12.418893 | -0.42594613 | 0.67026567 | 0.99276472 | -7.0483961 | 793 | 531 | 262 | FALSE | FALSE | down |
| ADNI | plasma | T+ vs T- | MME | -0.044072679 | 9.3380354 | -0.32318931 | 0.74663928 | 0.99276472 | -7.0866901 | 793 | 531 | 262 | FALSE | FALSE | down |
| ADNI | plasma | T+ vs T- | NEFH | 0.035144226 | 18.51843 | 0.2005834 | 0.8410771 | 0.99276472 | -7.1186389 | 793 | 531 | 262 | FALSE | FALSE | up |
| ADNI | plasma | T+ vs T- | NPTX1 | -0.016989908 | 11.552505 | -0.49829883 | 0.61841518 | 0.99276472 | -7.0151331 | 793 | 531 | 262 | FALSE | FALSE | down |
| ADNI | plasma | T+ vs T- | NRGN | 0.034666389 | 16.30272 | 0.19395213 | 0.84626432 | 0.99276472 | -7.1199406 | 793 | 531 | 262 | FALSE | FALSE | up |
| ADNI | plasma | T+ vs T- | Oligo-SNCA | 0.052962434 | 12.054404 | 0.20498555 | 0.83763738 | 0.99276472 | -7.1177506 | 793 | 531 | 262 | FALSE | FALSE | up |
| ADNI | plasma | T+ vs T- | PDLIM5 | 0.05219735 | 10.598409 | 0.32389455 | 0.74610549 | 0.99276472 | -7.0864631 | 793 | 531 | 262 | FALSE | FALSE | up |
| ADNI | plasma | T+ vs T- | PGK1 | -0.056715219 | 10.726245 | -0.33057687 | 0.7410537 | 0.99276472 | -7.0842874 | 793 | 531 | 262 | FALSE | FALSE | down |
| ADNI | plasma | T+ vs T- | POSTN | 0.023802298 | 13.455809 | 0.45165189 | 0.65164648 | 0.99276472 | -7.0371744 | 793 | 531 | 262 | FALSE | FALSE | up |
| ADNI | plasma | T+ vs T- | PRDX6 | -0.0076446554 | 12.0455 | -0.068986411 | 0.94501828 | 0.99276472 | -7.1362889 | 793 | 531 | 262 | FALSE | FALSE | down |
| ADNI | plasma | T+ vs T- | PSEN1 | 0.0062790383 | 12.171903 | 0.093166756 | 0.92579523 | 0.99276472 | -7.1343381 | 793 | 531 | 262 | FALSE | FALSE | up |
| ADNI | plasma | T+ vs T- | RUVBL2 | 0.020909382 | 11.186997 | 0.40419301 | 0.6861826 | 0.99276472 | -7.057379 | 793 | 531 | 262 | FALSE | FALSE | up |
| ADNI | plasma | T+ vs T- | S100B | 0.0059304779 | 11.94047 | 0.10891401 | 0.91329896 | 0.99276472 | -7.1327548 | 793 | 531 | 262 | FALSE | FALSE | up |
| ADNI | plasma | T+ vs T- | SAA1 | 0.025431497 | 10.745706 | 0.18022204 | 0.85702549 | 0.99276472 | -7.1224966 | 793 | 531 | 262 | FALSE | FALSE | up |
| ADNI | plasma | T+ vs T- | SNAP25 | -0.0016548535 | 12.817951 | -0.21744643 | 0.82791784 | 0.99276472 | -7.1151317 | 793 | 531 | 262 | FALSE | FALSE | down |
| ADNI | plasma | T+ vs T- | SNCA | -0.029997157 | 13.647318 | -0.33156992 | 0.74030391 | 0.99276472 | -7.0839603 | 793 | 531 | 262 | FALSE | FALSE | down |
| ADNI | plasma | T+ vs T- | SOD1 | -0.015053889 | 12.081226 | -0.17045986 | 0.86469311 | 0.99276472 | -7.1241999 | 793 | 531 | 262 | FALSE | FALSE | down |
| ADNI | plasma | T+ vs T- | TAFA5 | -0.0043663096 | 11.051552 | -0.078489428 | 0.93745905 | 0.99276472 | -7.1355916 | 793 | 531 | 262 | FALSE | FALSE | down |
| ADNI | plasma | T+ vs T- | TARDBP | -0.036718016 | 13.37081 | -0.28191692 | 0.77808267 | 0.99276472 | -7.0991146 | 793 | 531 | 262 | FALSE | FALSE | down |
| ADNI | plasma | T+ vs T- | TREM2 | -0.010153712 | 12.867275 | -0.21246447 | 0.83180071 | 0.99276472 | -7.1161973 | 793 | 531 | 262 | FALSE | FALSE | down |
| ADNI | plasma | T+ vs T- | UCHL1 | 0.0013549958 | 11.587985 | 0.13498084 | 0.89266217 | 0.99276472 | -7.1295916 | 793 | 531 | 262 | FALSE | FALSE | up |
| ADNI | plasma | T+ vs T- | pTDP43-409 | 0.017398357 | 10.73662 | 0.38470753 | 0.70055993 | 0.99276472 | -7.0650259 | 793 | 531 | 262 | FALSE | FALSE | up |
| ADNI | plasma | T+ vs T- | ANXA5 | -0.0066093253 | 12.32062 | -0.02976838 | 0.97625946 | 0.99719698 | -7.1382159 | 793 | 531 | 262 | FALSE | FALSE | down |
| ADNI | plasma | T+ vs T- | REST | 0.0011737571 | 12.947845 | 0.023360859 | 0.98136846 | 0.99719698 | -7.1383853 | 793 | 531 | 262 | FALSE | FALSE | up |
| ADNI | plasma | T+ vs T- | CALB2 | 4.0687062e-05 | 10.044539 | 0.00090552185 | 0.99927773 | 0.99927773 | -7.1386564 | 793 | 531 | 262 | FALSE | FALSE | up |
| ADNI | plasma | T+ vs T- | pSNCA-129 | -0.00078136999 | 13.760962 | -0.006393924 | 0.99490007 | 0.99927773 | -7.1386365 | 793 | 531 | 262 | FALSE | FALSE | down |

**ADNI | plasma | N+ vs N-**

| **Cohort** | **Fluid** | **Contrast** | **Protein** | **log₂FC** | **AveExpr** | **t** | **P.Value** | **FDR** | **B** | **N_total** | **N_group1** | **N_group2** | **Pass_FDR** | **Pass_both_thresholds** | **Direction** |
| --- | --- | --- | --- | --- | --- | --- | --- | --- | --- | --- | --- | --- | --- | --- | --- |
| ADNI | plasma | N+ vs N- | pTau-217 | 0.58815669 | 11.899171 | 10.291658 | 2.2831699e-23 | 2.8767941e-21 | 42.244403 | 793 | 497 | 296 | TRUE | TRUE | up |
| ADNI | plasma | N+ vs N- | pTau-231 | 0.3959688 | 13.812086 | 7.8725153 | 1.1748923e-14 | 7.4018216e-13 | 22.545073 | 793 | 497 | 296 | TRUE | FALSE | up |
| ADNI | plasma | N+ vs N- | pTau-181 | 0.34008104 | 13.532756 | 6.9603964 | 7.2478364e-12 | 3.0440913e-10 | 16.26899 | 793 | 497 | 296 | TRUE | FALSE | up |
| ADNI | plasma | N+ vs N- | GFAP | 0.27431456 | 13.336909 | 5.4350757 | 7.3474234e-08 | 2.3144384e-06 | 7.3216061 | 793 | 497 | 296 | TRUE | FALSE | up |
| ADNI | plasma | N+ vs N- | MAPT | 0.22321397 | 12.164537 | 5.3363005 | 1.2474301e-07 | 3.1435239e-06 | 6.8118258 | 793 | 497 | 296 | TRUE | FALSE | up |
| ADNI | plasma | N+ vs N- | ACHE | 0.24583152 | 13.408239 | 3.9469582 | 8.6363764e-05 | 0.0018136391 | 0.57725056 | 793 | 497 | 296 | TRUE | FALSE | up |
| ADNI | plasma | N+ vs N- | NGF | 0.11661681 | 8.7824569 | 3.4261973 | 0.00064442514 | 0.011599653 | -1.2986418 | 793 | 497 | 296 | TRUE | FALSE | up |
| ADNI | plasma | N+ vs N- | BACE1 | 0.066221884 | 13.485286 | 2.9076685 | 0.0037456077 | 0.052438508 | -2.9105575 | 793 | 497 | 296 | FALSE | FALSE | up |
| ADNI | plasma | N+ vs N- | CCL11 | 0.12478869 | 13.718093 | 2.9301888 | 0.0034873475 | 0.052438508 | -2.8459015 | 793 | 497 | 296 | FALSE | FALSE | up |
| ADNI | plasma | N+ vs N- | VSNL1 | 0.085311119 | 12.281453 | 2.7621619 | 0.0058783349 | 0.07406702 | -3.3165347 | 793 | 497 | 296 | FALSE | FALSE | up |
| ADNI | plasma | N+ vs N- | NEFL | 0.10955981 | 11.679451 | 2.7075994 | 0.0069268763 | 0.07934422 | -3.4635037 | 793 | 497 | 296 | FALSE | FALSE | up |
| ADNI | plasma | N+ vs N- | IGF1R | 0.040087122 | 12.240122 | 2.4529862 | 0.014387457 | 0.15106829 | -4.1112356 | 793 | 497 | 296 | FALSE | FALSE | up |
| ADNI | plasma | N+ vs N- | IL10 | 0.14490716 | 12.311109 | 2.2231062 | 0.026497095 | 0.25681799 | -4.6419612 | 793 | 497 | 296 | FALSE | FALSE | up |
| ADNI | plasma | N+ vs N- | PGF | 0.068565412 | 13.649756 | 2.1283263 | 0.033625753 | 0.30263177 | -4.8457837 | 793 | 497 | 296 | FALSE | FALSE | up |
| ADNI | plasma | N+ vs N- | NPTX2 | 0.055991984 | 13.347348 | 2.007407 | 0.045053984 | 0.37845347 | -5.0930747 | 793 | 497 | 296 | FALSE | FALSE | up |
| ADNI | plasma | N+ vs N- | CHI3L1 | 0.14790608 | 13.307362 | 1.9485671 | 0.051708645 | 0.39532113 | -5.2082322 | 793 | 497 | 296 | FALSE | FALSE | up |
| ADNI | plasma | N+ vs N- | CXCL10 | 0.10629944 | 11.271722 | 1.9102218 | 0.056474448 | 0.39532113 | -5.2814533 | 793 | 497 | 296 | FALSE | FALSE | up |
| ADNI | plasma | N+ vs N- | IL1B | -0.09717891 | 10.691047 | -1.9340637 | 0.053470002 | 0.39532113 | -5.2360961 | 793 | 497 | 296 | FALSE | FALSE | down |
| ADNI | plasma | N+ vs N- | AGRN | 0.045402076 | 13.205262 | 1.7919101 | 0.073538396 | 0.46329189 | -5.4982827 | 793 | 497 | 296 | FALSE | FALSE | up |
| ADNI | plasma | N+ vs N- | IL15 | 0.044156726 | 13.022508 | 1.8068214 | 0.071178875 | 0.46329189 | -5.4717114 | 793 | 497 | 296 | FALSE | FALSE | up |
| ADNI | plasma | N+ vs N- | CD40LG | -0.22127037 | 12.846874 | -1.72876 | 0.084251368 | 0.50550821 | -5.6083921 | 793 | 497 | 296 | FALSE | FALSE | down |
| ADNI | plasma | N+ vs N- | S100A12 | 0.1792254 | 12.59537 | 1.6701507 | 0.095294402 | 0.52204759 | -5.7070766 | 793 | 497 | 296 | FALSE | FALSE | up |
| ADNI | plasma | N+ vs N- | TREM1 | 0.059302557 | 11.39001 | 1.683881 | 0.092608418 | 0.52204759 | -5.684261 | 793 | 497 | 296 | FALSE | FALSE | up |
| ADNI | plasma | N+ vs N- | PDGFRB | 0.08271686 | 12.915791 | 1.6284513 | 0.10383669 | 0.52333694 | -5.7752316 | 793 | 497 | 296 | FALSE | FALSE | up |
| ADNI | plasma | N+ vs N- | SFTPD | 0.1018862 | 13.313642 | 1.641636 | 0.10107222 | 0.52333694 | -5.753867 | 793 | 497 | 296 | FALSE | FALSE | up |
| ADNI | plasma | N+ vs N- | APOE | -0.067942554 | 13.225898 | -1.5626406 | 0.11854645 | 0.56587869 | -5.8793128 | 793 | 497 | 296 | FALSE | FALSE | down |
| ADNI | plasma | N+ vs N- | PARK7 | -0.17548109 | 13.180652 | -1.551214 | 0.12125972 | 0.56587869 | -5.8969496 | 793 | 497 | 296 | FALSE | FALSE | down |
| ADNI | plasma | N+ vs N- | CNTN2 | -0.14673262 | 7.8125734 | -1.4905953 | 0.13647572 | 0.61414075 | -5.9883622 | 793 | 497 | 296 | FALSE | FALSE | down |
| ADNI | plasma | N+ vs N- | GOT1 | 0.054343887 | 11.648811 | 1.4325866 | 0.15238029 | 0.63999723 | -6.0724468 | 793 | 497 | 296 | FALSE | FALSE | up |
| ADNI | plasma | N+ vs N- | VGF | -0.21244857 | 12.611345 | -1.4375995 | 0.1509524 | 0.63999723 | -6.0653115 | 793 | 497 | 296 | FALSE | FALSE | down |
| ADNI | plasma | N+ vs N- | ARSA | -0.13871954 | 11.224533 | -1.2499498 | 0.21169617 | 0.74346792 | -6.315492 | 793 | 497 | 296 | FALSE | FALSE | down |
| ADNI | plasma | N+ vs N- | CCL4 | 0.08633906 | 11.815711 | 1.1464362 | 0.25196947 | 0.74346792 | -6.4386084 | 793 | 497 | 296 | FALSE | FALSE | up |
| ADNI | plasma | N+ vs N- | CHIT1 | 0.22855412 | 13.689934 | 1.1969068 | 0.23170986 | 0.74346792 | -6.3799039 | 793 | 497 | 296 | FALSE | FALSE | up |
| ADNI | plasma | N+ vs N- | GDI1 | -0.071217954 | 8.8744837 | -1.1525469 | 0.24945272 | 0.74346792 | -6.4316349 | 793 | 497 | 296 | FALSE | FALSE | down |
| ADNI | plasma | N+ vs N- | HTT | -0.16096606 | 13.850602 | -1.2662097 | 0.20581947 | 0.74346792 | -6.2951902 | 793 | 497 | 296 | FALSE | FALSE | down |
| ADNI | plasma | N+ vs N- | IL2 | 0.0512852 | 10.651703 | 1.1397086 | 0.25476076 | 0.74346792 | -6.4462433 | 793 | 497 | 296 | FALSE | FALSE | up |
| ADNI | plasma | N+ vs N- | IL4 | 0.0779522 | 10.919791 | 1.2256457 | 0.22070532 | 0.74346792 | -6.3453506 | 793 | 497 | 296 | FALSE | FALSE | up |
| ADNI | plasma | N+ vs N- | MDH1 | -0.12022524 | 12.418893 | -1.2889912 | 0.19778675 | 0.74346792 | -6.266306 | 793 | 497 | 296 | FALSE | FALSE | down |
| ADNI | plasma | N+ vs N- | PGK1 | -0.19677778 | 10.726245 | -1.1874913 | 0.23539865 | 0.74346792 | -6.3910465 | 793 | 497 | 296 | FALSE | FALSE | down |
| ADNI | plasma | N+ vs N- | SNCA | -0.11418696 | 13.647318 | -1.3070029 | 0.1916004 | 0.74346792 | -6.2431064 | 793 | 497 | 296 | FALSE | FALSE | down |
| ADNI | plasma | N+ vs N- | SOD1 | -0.10526876 | 12.081226 | -1.2342661 | 0.21747886 | 0.74346792 | -6.3348269 | 793 | 497 | 296 | FALSE | FALSE | down |
| ADNI | plasma | N+ vs N- | TARDBP | -0.14192468 | 13.37081 | -1.1281095 | 0.25962372 | 0.74346792 | -6.4593014 | 793 | 497 | 296 | FALSE | FALSE | down |
| ADNI | plasma | N+ vs N- | VCAM1 | 0.026724126 | 13.447396 | 1.1619187 | 0.24562712 | 0.74346792 | -6.420868 | 793 | 497 | 296 | FALSE | FALSE | up |
| ADNI | plasma | N+ vs N- | YWHAG | -0.034352476 | 11.87825 | -1.1686432 | 0.2429077 | 0.74346792 | -6.4130889 | 793 | 497 | 296 | FALSE | FALSE | down |
| ADNI | plasma | N+ vs N- | CRP | -0.11619912 | 11.451022 | -1.0685282 | 0.2856159 | 0.77517398 | -6.5242786 | 793 | 497 | 296 | FALSE | FALSE | down |
| ADNI | plasma | N+ vs N- | IGFBP7 | 0.021059707 | 13.112446 | 1.0607118 | 0.2891522 | 0.77517398 | -6.532542 | 793 | 497 | 296 | FALSE | FALSE | up |
| ADNI | plasma | N+ vs N- | NPY | 0.10738654 | 14.99324 | 1.0729932 | 0.28360903 | 0.77517398 | -6.519531 | 793 | 497 | 296 | FALSE | FALSE | up |
| ADNI | plasma | N+ vs N- | Abeta42 | -0.042875313 | 12.416167 | -0.95081011 | 0.34199797 | 0.80095849 | -6.6423238 | 793 | 497 | 296 | FALSE | FALSE | down |
| ADNI | plasma | N+ vs N- | BASP1 | -0.13304571 | 11.893332 | -0.89027794 | 0.37359367 | 0.80095849 | -6.6976786 | 793 | 497 | 296 | FALSE | FALSE | down |
| ADNI | plasma | N+ vs N- | CCL3 | -0.061001657 | 11.789254 | -0.97469191 | 0.33001803 | 0.80095849 | -6.6194856 | 793 | 497 | 296 | FALSE | FALSE | down |
| ADNI | plasma | N+ vs N- | FABP3 | -0.033413926 | 13.154646 | -0.91149675 | 0.36231787 | 0.80095849 | -6.6786881 | 793 | 497 | 296 | FALSE | FALSE | down |
| ADNI | plasma | N+ vs N- | FGF2 | -0.13612248 | 13.541512 | -0.99340362 | 0.32082416 | 0.80095849 | -6.6011967 | 793 | 497 | 296 | FALSE | FALSE | down |
| ADNI | plasma | N+ vs N- | GDF15 | 0.15765568 | 12.19832 | 0.8733873 | 0.38272326 | 0.80095849 | -6.7124764 | 793 | 497 | 296 | FALSE | FALSE | up |
| ADNI | plasma | N+ vs N- | ICAM1 | 0.024942588 | 12.878742 | 0.86405297 | 0.38782684 | 0.80095849 | -6.7205328 | 793 | 497 | 296 | FALSE | FALSE | up |
| ADNI | plasma | N+ vs N- | KDR | 0.12071713 | 12.299125 | 0.88380807 | 0.37707463 | 0.80095849 | -6.7033803 | 793 | 497 | 296 | FALSE | FALSE | up |
| ADNI | plasma | N+ vs N- | NPTXR | -0.032694201 | 10.989891 | -0.86950924 | 0.38483857 | 0.80095849 | -6.7158341 | 793 | 497 | 296 | FALSE | FALSE | down |
| ADNI | plasma | N+ vs N- | POSTN | 0.049962324 | 13.455809 | 0.9811993 | 0.32680147 | 0.80095849 | -6.6131646 | 793 | 497 | 296 | FALSE | FALSE | up |
| ADNI | plasma | N+ vs N- | REST | 0.044322322 | 12.947845 | 0.91302706 | 0.36151299 | 0.80095849 | -6.6773013 | 793 | 497 | 296 | FALSE | FALSE | up |
| ADNI | plasma | N+ vs N- | SFRP1 | 0.073287097 | 15.219506 | 0.93847569 | 0.34829315 | 0.80095849 | -6.6538979 | 793 | 497 | 296 | FALSE | FALSE | up |
| ADNI | plasma | N+ vs N- | TEK | -0.021333397 | 12.806769 | -0.85264117 | 0.39412243 | 0.80095849 | -6.7302649 | 793 | 497 | 296 | FALSE | FALSE | down |
| ADNI | plasma | N+ vs N- | TNF | 0.035156477 | 13.354033 | 1.0075807 | 0.3139711 | 0.80095849 | -6.5871091 | 793 | 497 | 296 | FALSE | FALSE | up |
| ADNI | plasma | N+ vs N- | pSNCA-129 | -0.11414129 | 13.760962 | -0.96679063 | 0.33395109 | 0.80095849 | -6.6271042 | 793 | 497 | 296 | FALSE | FALSE | down |
| ADNI | plasma | N+ vs N- | ANXA5 | -0.17226153 | 12.32062 | -0.80294099 | 0.42225566 | 0.8017523 | -6.7711434 | 793 | 497 | 296 | FALSE | FALSE | down |
| ADNI | plasma | N+ vs N- | BDNF | -0.0879944 | 13.70788 | -0.79591016 | 0.42632861 | 0.8017523 | -6.7767285 | 793 | 497 | 296 | FALSE | FALSE | down |
| ADNI | plasma | N+ vs N- | NPTX1 | 0.027264259 | 11.552505 | 0.82743138 | 0.40824797 | 0.8017523 | -6.7513062 | 793 | 497 | 296 | FALSE | FALSE | up |
| ADNI | plasma | N+ vs N- | Oligo-SNCA | -0.20802936 | 12.054404 | -0.83325815 | 0.40495655 | 0.8017523 | -6.7464989 | 793 | 497 | 296 | FALSE | FALSE | down |
| ADNI | plasma | N+ vs N- | PRDX6 | -0.086869135 | 12.0455 | -0.81128065 | 0.41745425 | 0.8017523 | -6.7644551 | 793 | 497 | 296 | FALSE | FALSE | down |
| ADNI | plasma | N+ vs N- | CXCL1 | -0.070617628 | 12.008193 | -0.74391666 | 0.45715297 | 0.84707756 | -6.8165081 | 793 | 497 | 296 | FALSE | FALSE | down |
| ADNI | plasma | N+ vs N- | CCL2 | 0.035595574 | 10.207694 | 0.67343885 | 0.50086944 | 0.86151941 | -6.8661468 | 793 | 497 | 296 | FALSE | FALSE | up |
| ADNI | plasma | N+ vs N- | CSF2 | -0.045899532 | 14.748262 | -0.70672748 | 0.47994883 | 0.86151941 | -6.8433154 | 793 | 497 | 296 | FALSE | FALSE | down |
| ADNI | plasma | N+ vs N- | FLT1 | 0.021149187 | 13.463483 | 0.66259906 | 0.50778489 | 0.86151941 | -6.873344 | 793 | 497 | 296 | FALSE | FALSE | up |
| ADNI | plasma | N+ vs N- | IL12p70 | -0.046523304 | 13.465644 | -0.69236633 | 0.48891524 | 0.86151941 | -6.8533001 | 793 | 497 | 296 | FALSE | FALSE | down |
| ADNI | plasma | N+ vs N- | IL16 | 0.025958194 | 12.787441 | 0.62322344 | 0.53332154 | 0.86151941 | -6.8985062 | 793 | 497 | 296 | FALSE | FALSE | up |
| ADNI | plasma | N+ vs N- | IL9 | 0.045251115 | 10.509838 | 0.66347523 | 0.50722406 | 0.86151941 | -6.8727666 | 793 | 497 | 296 | FALSE | FALSE | up |
| ADNI | plasma | N+ vs N- | MSLN | -0.042237735 | 13.507774 | -0.62849626 | 0.52986456 | 0.86151941 | -6.895226 | 793 | 497 | 296 | FALSE | FALSE | down |
| ADNI | plasma | N+ vs N- | NRGN | -0.11153941 | 16.30272 | -0.64570965 | 0.51865904 | 0.86151941 | -6.8843254 | 793 | 497 | 296 | FALSE | FALSE | down |
| ADNI | plasma | N+ vs N- | SMOC1 | 0.063187727 | 12.760329 | 0.65831573 | 0.5105313 | 0.86151941 | -6.8761558 | 793 | 497 | 296 | FALSE | FALSE | up |
| ADNI | plasma | N+ vs N- | SNAP25 | 0.0046078707 | 12.817951 | 0.62649324 | 0.53117644 | 0.86151941 | -6.8964753 | 793 | 497 | 296 | FALSE | FALSE | up |
| ADNI | plasma | N+ vs N- | CCL13 | 0.029859684 | 13.170055 | 0.56606511 | 0.57151381 | 0.86209775 | -6.9322925 | 793 | 497 | 296 | FALSE | FALSE | up |
| ADNI | plasma | N+ vs N- | CRH | 0.044353224 | 12.22954 | 0.58035849 | 0.56184204 | 0.86209775 | -6.9241479 | 793 | 497 | 296 | FALSE | FALSE | up |
| ADNI | plasma | N+ vs N- | ENO2 | -0.041182107 | 12.823457 | -0.56133507 | 0.57473183 | 0.86209775 | -6.9349431 | 793 | 497 | 296 | FALSE | FALSE | down |
| ADNI | plasma | N+ vs N- | GDNF | -0.084642092 | 11.262178 | -0.56571829 | 0.57174948 | 0.86209775 | -6.9324876 | 793 | 497 | 296 | FALSE | FALSE | down |
| ADNI | plasma | N+ vs N- | UBB | 0.074238898 | 12.305531 | 0.61039768 | 0.54177782 | 0.86209775 | -6.9063699 | 793 | 497 | 296 | FALSE | FALSE | up |
| ADNI | plasma | N+ vs N- | YWHAZ | 0.016014658 | 11.811358 | 0.56988432 | 0.56892175 | 0.86209775 | -6.9301361 | 793 | 497 | 296 | FALSE | FALSE | up |
| ADNI | plasma | N+ vs N- | PDLIM5 | 0.084780336 | 10.598409 | 0.54427769 | 0.58640736 | 0.86926268 | -6.9443169 | 793 | 497 | 296 | FALSE | FALSE | up |
| ADNI | plasma | N+ vs N- | IL6 | -0.024593567 | 12.894965 | -0.52454805 | 0.6000478 | 0.8791398 | -6.9547988 | 793 | 497 | 296 | FALSE | FALSE | down |
| ADNI | plasma | N+ vs N- | TREM2 | -0.022246628 | 12.867275 | -0.48160928 | 0.63021985 | 0.9127322 | -6.9762744 | 793 | 497 | 296 | FALSE | FALSE | down |
| ADNI | plasma | N+ vs N- | Abeta38 | 0.027584308 | 9.5709062 | 0.44009252 | 0.65999313 | 0.91718942 | -6.995297 | 793 | 497 | 296 | FALSE | FALSE | up |
| ADNI | plasma | N+ vs N- | CCL26 | 0.032921802 | 11.46364 | 0.45790163 | 0.64715181 | 0.91718942 | -6.9873468 | 793 | 497 | 296 | FALSE | FALSE | up |
| ADNI | plasma | N+ vs N- | FOLR1 | -0.014121529 | 10.309816 | -0.44389393 | 0.6572435 | 0.91718942 | -6.9936264 | 793 | 497 | 296 | FALSE | FALSE | down |
| ADNI | plasma | N+ vs N- | IL18 | -0.01889074 | 13.050907 | -0.43675008 | 0.66241458 | 0.91718942 | -6.9967539 | 793 | 497 | 296 | FALSE | FALSE | down |
| ADNI | plasma | N+ vs N- | CCL17 | -0.055384141 | 13.55906 | -0.39692818 | 0.69152999 | 0.92278845 | -7.0132582 | 793 | 497 | 296 | FALSE | FALSE | down |
| ADNI | plasma | N+ vs N- | DDC | 0.018398875 | 11.436432 | 0.39120544 | 0.6957532 | 0.92278845 | -7.0155005 | 793 | 497 | 296 | FALSE | FALSE | up |
| ADNI | plasma | N+ vs N- | NEFH | -0.068777168 | 18.51843 | -0.40610355 | 0.68477891 | 0.92278845 | -7.0095952 | 793 | 497 | 296 | FALSE | FALSE | down |
| ADNI | plasma | N+ vs N- | SLIT2 | 0.01420101 | 13.230064 | 0.40520983 | 0.6854354 | 0.92278845 | -7.0099557 | 793 | 497 | 296 | FALSE | FALSE | up |
| ADNI | plasma | N+ vs N- | VEGFD | -0.026749976 | 12.264268 | -0.37824627 | 0.7053515 | 0.92577385 | -7.0204578 | 793 | 497 | 296 | FALSE | FALSE | down |
| ADNI | plasma | N+ vs N- | CCL22 | -0.014092645 | 12.839852 | -0.33501109 | 0.73770761 | 0.92689849 | -7.0357894 | 793 | 497 | 296 | FALSE | FALSE | down |
| ADNI | plasma | N+ vs N- | CD63 | -0.01254577 | 12.307859 | -0.34883958 | 0.72730474 | 0.92689849 | -7.0310878 | 793 | 497 | 296 | FALSE | FALSE | down |
| ADNI | plasma | N+ vs N- | HBA1 | 0.043999617 | 6.9467986 | 0.30292048 | 0.76203197 | 0.92689849 | -7.0459675 | 793 | 497 | 296 | FALSE | FALSE | up |
| ADNI | plasma | N+ vs N- | IL33 | 0.011427973 | 12.151655 | 0.29894932 | 0.76505907 | 0.92689849 | -7.0471559 | 793 | 497 | 296 | FALSE | FALSE | up |
| ADNI | plasma | N+ vs N- | IL6R | -0.010892614 | 12.068669 | -0.30139789 | 0.76319217 | 0.92689849 | -7.046425 | 793 | 497 | 296 | FALSE | FALSE | down |
| ADNI | plasma | N+ vs N- | IL7 | -0.024838264 | 13.085417 | -0.32094363 | 0.74833986 | 0.92689849 | -7.0403772 | 793 | 497 | 296 | FALSE | FALSE | down |
| ADNI | plasma | N+ vs N- | KLK6 | -0.011055802 | 12.216801 | -0.32612963 | 0.74441456 | 0.92689849 | -7.0387088 | 793 | 497 | 296 | FALSE | FALSE | down |
| ADNI | plasma | N+ vs N- | UCHL1 | 0.0032795604 | 11.587985 | 0.33798618 | 0.73546537 | 0.92689849 | -7.0347939 | 793 | 497 | 296 | FALSE | FALSE | up |
| ADNI | plasma | N+ vs N- | CALB2 | 0.010384062 | 10.044539 | 0.23907969 | 0.81110716 | 0.95880148 | -7.0631716 | 793 | 497 | 296 | FALSE | FALSE | up |
| ADNI | plasma | N+ vs N- | TIMP3 | 0.027603993 | 14.730706 | 0.23506531 | 0.8142203 | 0.95880148 | -7.0641179 | 793 | 497 | 296 | FALSE | FALSE | up |
| ADNI | plasma | N+ vs N- | VEGFA | -0.0072960053 | 13.730398 | -0.25254052 | 0.80069043 | 0.95880148 | -7.0598812 | 793 | 497 | 296 | FALSE | FALSE | down |
| ADNI | plasma | N+ vs N- | Abeta40 | 0.0095688945 | 10.912733 | 0.1646743 | 0.8692434 | 0.96324726 | -7.0781088 | 793 | 497 | 296 | FALSE | FALSE | up |
| ADNI | plasma | N+ vs N- | CXCL8 | 0.010283236 | 10.260772 | 0.16681678 | 0.86755785 | 0.96324726 | -7.0777557 | 793 | 497 | 296 | FALSE | FALSE | up |
| ADNI | plasma | N+ vs N- | MME | 0.020050468 | 9.3380354 | 0.15209177 | 0.87915425 | 0.96324726 | -7.0800907 | 793 | 497 | 296 | FALSE | FALSE | up |
| ADNI | plasma | N+ vs N- | PSEN1 | -0.012285465 | 12.171903 | -0.18857476 | 0.85047562 | 0.96324726 | -7.0739108 | 793 | 497 | 296 | FALSE | FALSE | down |
| ADNI | plasma | N+ vs N- | PTN | 0.012309453 | 6.868528 | 0.19269825 | 0.84724591 | 0.96324726 | -7.0731291 | 793 | 497 | 296 | FALSE | FALSE | up |
| ADNI | plasma | N+ vs N- | S100B | 0.010811385 | 11.94047 | 0.20540007 | 0.83731364 | 0.96324726 | -7.0706149 | 793 | 497 | 296 | FALSE | FALSE | up |
| ADNI | plasma | N+ vs N- | SNCB | -0.0039403452 | 10.836801 | -0.15501623 | 0.87684899 | 0.96324726 | -7.0796441 | 793 | 497 | 296 | FALSE | FALSE | down |
| ADNI | plasma | N+ vs N- | SQSTM1 | 0.0115017 | 11.626986 | 0.18526215 | 0.85307202 | 0.96324726 | -7.0745266 | 793 | 497 | 296 | FALSE | FALSE | up |
| ADNI | plasma | N+ vs N- | IL17A | -0.015207941 | 11.220003 | -0.14220079 | 0.88695846 | 0.9634204 | -7.081538 | 793 | 497 | 296 | FALSE | FALSE | down |
| ADNI | plasma | N+ vs N- | FCN2 | 0.0053903777 | 12.455908 | 0.1099338 | 0.91249041 | 0.98268198 | -7.0855834 | 793 | 497 | 296 | FALSE | FALSE | up |
| ADNI | plasma | N+ vs N- | TAFA5 | -0.004975545 | 11.051552 | -0.092524046 | 0.92630566 | 0.98910605 | -7.0873361 | 793 | 497 | 296 | FALSE | FALSE | down |
| ADNI | plasma | N+ vs N- | IL5 | -0.007020615 | 13.121425 | -0.0818521 | 0.93478551 | 0.9897729 | -7.0882614 | 793 | 497 | 296 | FALSE | FALSE | down |
| ADNI | plasma | N+ vs N- | CST3 | -0.00093498508 | 13.312248 | -0.050505528 | 0.95973259 | 0.9898105 | -7.0903244 | 793 | 497 | 296 | FALSE | FALSE | down |
| ADNI | plasma | N+ vs N- | CX3CL1 | -0.0019066618 | 13.376408 | -0.070295103 | 0.94397696 | 0.9898105 | -7.0891357 | 793 | 497 | 296 | FALSE | FALSE | down |
| ADNI | plasma | N+ vs N- | IFNG | 0.0016097711 | 12.569214 | 0.020264361 | 0.98383772 | 0.9898105 | -7.0913886 | 793 | 497 | 296 | FALSE | FALSE | up |
| ADNI | plasma | N+ vs N- | IL13 | 0.001369534 | 13.107169 | 0.012775123 | 0.9898105 | 0.9898105 | -7.0915117 | 793 | 497 | 296 | FALSE | FALSE | up |
| ADNI | plasma | N+ vs N- | RUVBL2 | 0.0022563187 | 11.186997 | 0.045114838 | 0.96402743 | 0.9898105 | -7.0905808 | 793 | 497 | 296 | FALSE | FALSE | up |
| ADNI | plasma | N+ vs N- | SAA1 | 0.0030685393 | 10.745706 | 0.022494438 | 0.98205935 | 0.9898105 | -7.0913412 | 793 | 497 | 296 | FALSE | FALSE | up |
| ADNI | plasma | N+ vs N- | pTDP43-409 | -0.0017131034 | 10.73662 | -0.039181551 | 0.96875576 | 0.9898105 | -7.0908295 | 793 | 497 | 296 | FALSE | FALSE | down |

**ADNI | plasma | A+T+N+ vs A+T-N-**

| **Cohort** | **Fluid** | **Contrast** | **Protein** | **log₂FC** | **AveExpr** | **t** | **P.Value** | **FDR** | **B** | **N_total** | **N_group1** | **N_group2** | **Pass_FDR** | **Pass_both_thresholds** | **Direction** |
| --- | --- | --- | --- | --- | --- | --- | --- | --- | --- | --- | --- | --- | --- | --- | --- |
| ADNI | plasma | A+T+N+ vs A+T-N- | pTau-217 | 0.65881119 | 11.895343 | 10.938987 | 6.80449e-26 | 8.5736575e-24 | 47.962725 | 749 | 492 | 257 | TRUE | TRUE | up |
| ADNI | plasma | A+T+N+ vs A+T-N- | pTau-231 | 0.44163455 | 13.811655 | 8.3342161 | 3.8703969e-16 | 2.4383501e-14 | 25.857241 | 749 | 492 | 257 | TRUE | FALSE | up |
| ADNI | plasma | A+T+N+ vs A+T-N- | pTau-181 | 0.37226981 | 13.533615 | 7.250821 | 1.0596345e-12 | 4.450465e-11 | 18.1067 | 749 | 492 | 257 | TRUE | FALSE | up |
| ADNI | plasma | A+T+N+ vs A+T-N- | MAPT | 0.24354152 | 12.162691 | 5.5079904 | 5.0347902e-08 | 1.5859589e-06 | 7.6384265 | 749 | 492 | 257 | TRUE | FALSE | up |
| ADNI | plasma | A+T+N+ vs A+T-N- | GFAP | 0.28582996 | 13.342432 | 5.2804331 | 1.7027128e-07 | 4.2908362e-06 | 6.4638307 | 749 | 492 | 257 | TRUE | FALSE | up |
| ADNI | plasma | A+T+N+ vs A+T-N- | ACHE | 0.26102889 | 13.411113 | 3.9247391 | 9.5045473e-05 | 0.0019959549 | 0.43311917 | 749 | 492 | 257 | TRUE | FALSE | up |
| ADNI | plasma | A+T+N+ vs A+T-N- | NGF | 0.11866509 | 8.7856022 | 3.262803 | 0.0011545756 | 0.020782361 | -1.8928774 | 749 | 492 | 257 | TRUE | FALSE | up |
| ADNI | plasma | A+T+N+ vs A+T-N- | BACE1 | 0.062718167 | 13.486184 | 2.5875298 | 0.0098590692 | 0.15528034 | -3.8357475 | 749 | 492 | 257 | FALSE | FALSE | up |
| ADNI | plasma | A+T+N+ vs A+T-N- | VSNL1 | 0.082155812 | 12.284859 | 2.4877978 | 0.013076067 | 0.18306494 | -4.0854388 | 749 | 492 | 257 | FALSE | FALSE | up |
| ADNI | plasma | A+T+N+ vs A+T-N- | NEFL | 0.10506815 | 11.684959 | 2.4461789 | 0.014673252 | 0.18488297 | -4.1867841 | 749 | 492 | 257 | FALSE | FALSE | up |
| ADNI | plasma | A+T+N+ vs A+T-N- | CCL11 | 0.10586964 | 13.729938 | 2.3638041 | 0.018349714 | 0.21018763 | -4.3824129 | 749 | 492 | 257 | FALSE | FALSE | up |
| ADNI | plasma | A+T+N+ vs A+T-N- | IGF1R | 0.040353246 | 12.241314 | 2.3166093 | 0.020801386 | 0.21841455 | -4.491521 | 749 | 492 | 257 | FALSE | FALSE | up |
| ADNI | plasma | A+T+N+ vs A+T-N- | NPTX2 | 0.059297222 | 13.348694 | 1.9901507 | 0.046947423 | 0.45502887 | -5.1867717 | 749 | 492 | 257 | FALSE | FALSE | up |
| ADNI | plasma | A+T+N+ vs A+T-N- | VGF | -0.2886979 | 12.639086 | -1.8534921 | 0.064215693 | 0.57794124 | -5.4468583 | 749 | 492 | 257 | FALSE | FALSE | down |
| ADNI | plasma | A+T+N+ vs A+T-N- | CNTN2 | -0.1857465 | 7.8148797 | -1.7961028 | 0.072892129 | 0.61229388 | -5.5506189 | 749 | 492 | 257 | FALSE | FALSE | down |
| ADNI | plasma | A+T+N+ vs A+T-N- | IL10 | 0.1155395 | 12.325967 | 1.6528824 | 0.098785379 | 0.77793486 | -5.7954481 | 749 | 492 | 257 | FALSE | FALSE | up |
| ADNI | plasma | A+T+N+ vs A+T-N- | TREM1 | 0.060969751 | 11.392422 | 1.6224445 | 0.10514057 | 0.77927718 | -5.8448807 | 749 | 492 | 257 | FALSE | FALSE | up |
| ADNI | plasma | A+T+N+ vs A+T-N- | APOE | -0.073853386 | 13.227513 | -1.5869051 | 0.11296778 | 0.79077447 | -5.9014441 | 749 | 492 | 257 | FALSE | FALSE | down |
| ADNI | plasma | A+T+N+ vs A+T-N- | AGRN | 0.033385416 | 13.211731 | 1.2447817 | 0.21361205 | 0.79788734 | -6.3822387 | 749 | 492 | 257 | FALSE | FALSE | up |
| ADNI | plasma | A+T+N+ vs A+T-N- | CD40LG | -0.16916389 | 12.818503 | -1.238135 | 0.2160646 | 0.79788734 | -6.3904345 | 749 | 492 | 257 | FALSE | FALSE | down |
| ADNI | plasma | A+T+N+ vs A+T-N- | CHI3L1 | 0.11956497 | 13.332683 | 1.4736785 | 0.14099985 | 0.79788734 | -6.073353 | 749 | 492 | 257 | FALSE | FALSE | up |
| ADNI | plasma | A+T+N+ vs A+T-N- | CHIT1 | 0.26387109 | 13.683772 | 1.2913004 | 0.19700883 | 0.79788734 | -6.3236549 | 749 | 492 | 257 | FALSE | FALSE | up |
| ADNI | plasma | A+T+N+ vs A+T-N- | CXCL10 | 0.090411475 | 11.283589 | 1.5219552 | 0.12845394 | 0.79788734 | -6.0016012 | 749 | 492 | 257 | FALSE | FALSE | up |
| ADNI | plasma | A+T+N+ vs A+T-N- | GOT1 | 0.050297451 | 11.650887 | 1.2369765 | 0.21649415 | 0.79788734 | -6.3918586 | 749 | 492 | 257 | FALSE | FALSE | up |
| ADNI | plasma | A+T+N+ vs A+T-N- | IL15 | 0.035035637 | 13.027562 | 1.341124 | 0.18029789 | 0.79788734 | -6.2585368 | 749 | 492 | 257 | FALSE | FALSE | up |
| ADNI | plasma | A+T+N+ vs A+T-N- | IL1B | -0.070871658 | 10.679539 | -1.3259871 | 0.1852593 | 0.79788734 | -6.2785798 | 749 | 492 | 257 | FALSE | FALSE | down |
| ADNI | plasma | A+T+N+ vs A+T-N- | IL2 | 0.058444993 | 10.651011 | 1.2232373 | 0.22163537 | 0.79788734 | -6.4086458 | 749 | 492 | 257 | FALSE | FALSE | up |
| ADNI | plasma | A+T+N+ vs A+T-N- | NPTXR | -0.060309283 | 10.994954 | -1.5232838 | 0.12812138 | 0.79788734 | -5.9995941 | 749 | 492 | 257 | FALSE | FALSE | down |
| ADNI | plasma | A+T+N+ vs A+T-N- | PARK7 | -0.15813464 | 13.170901 | -1.3153055 | 0.18882081 | 0.79788734 | -6.2925872 | 749 | 492 | 257 | FALSE | FALSE | down |
| ADNI | plasma | A+T+N+ vs A+T-N- | PDGFRB | 0.078012366 | 12.914591 | 1.4423883 | 0.14962237 | 0.79788734 | -6.1186305 | 749 | 492 | 257 | FALSE | FALSE | up |
| ADNI | plasma | A+T+N+ vs A+T-N- | PGF | 0.048920459 | 13.65706 | 1.4236236 | 0.15498332 | 0.79788734 | -6.1453199 | 749 | 492 | 257 | FALSE | FALSE | up |
| ADNI | plasma | A+T+N+ vs A+T-N- | S100A12 | 0.15765662 | 12.610045 | 1.3812709 | 0.16761893 | 0.79788734 | -6.2042815 | 749 | 492 | 257 | FALSE | FALSE | up |
| ADNI | plasma | A+T+N+ vs A+T-N- | SFTPD | 0.084937827 | 13.32264 | 1.2869933 | 0.19850511 | 0.79788734 | -6.329169 | 749 | 492 | 257 | FALSE | FALSE | up |
| ADNI | plasma | A+T+N+ vs A+T-N- | VCAM1 | 0.031259303 | 13.449562 | 1.2823128 | 0.20014055 | 0.79788734 | -6.3351403 | 749 | 492 | 257 | FALSE | FALSE | up |
| ADNI | plasma | A+T+N+ vs A+T-N- | YWHAG | -0.040410147 | 11.879358 | -1.2834525 | 0.19974143 | 0.79788734 | -6.3336883 | 749 | 492 | 257 | FALSE | FALSE | down |
| ADNI | plasma | A+T+N+ vs A+T-N- | BASP1 | -0.19199805 | 11.909233 | -1.2043844 | 0.22883194 | 0.80091179 | -6.4313771 | 749 | 492 | 257 | FALSE | FALSE | down |
| ADNI | plasma | A+T+N+ vs A+T-N- | ARSA | -0.10766665 | 11.203102 | -0.91018453 | 0.36302605 | 0.85611185 | -6.7404997 | 749 | 492 | 257 | FALSE | FALSE | down |
| ADNI | plasma | A+T+N+ vs A+T-N- | Abeta42 | -0.054805882 | 12.416558 | -1.1327906 | 0.25767463 | 0.85611185 | -6.5144954 | 749 | 492 | 257 | FALSE | FALSE | down |
| ADNI | plasma | A+T+N+ vs A+T-N- | CCL13 | 0.044747096 | 13.171249 | 0.78846906 | 0.43067872 | 0.85611185 | -6.8432931 | 749 | 492 | 257 | FALSE | FALSE | up |
| ADNI | plasma | A+T+N+ vs A+T-N- | CCL2 | 0.050611195 | 10.208445 | 0.94614697 | 0.34438714 | 0.85611185 | -6.707315 | 749 | 492 | 257 | FALSE | FALSE | up |
| ADNI | plasma | A+T+N+ vs A+T-N- | CCL4 | 0.084372652 | 11.822666 | 1.1050299 | 0.2695112 | 0.85611185 | -6.5453596 | 749 | 492 | 257 | FALSE | FALSE | up |
| ADNI | plasma | A+T+N+ vs A+T-N- | CD63 | -0.035795378 | 12.314549 | -0.93401199 | 0.35060698 | 0.85611185 | -6.7186561 | 749 | 492 | 257 | FALSE | FALSE | down |
| ADNI | plasma | A+T+N+ vs A+T-N- | CRP | -0.12173878 | 11.458484 | -1.0539474 | 0.2922562 | 0.85611185 | -6.6001584 | 749 | 492 | 257 | FALSE | FALSE | down |
| ADNI | plasma | A+T+N+ vs A+T-N- | CSF2 | -0.076277154 | 14.755894 | -1.0923043 | 0.27506021 | 0.85611185 | -6.5592527 | 749 | 492 | 257 | FALSE | FALSE | down |
| ADNI | plasma | A+T+N+ vs A+T-N- | CX3CL1 | -0.023002187 | 13.383186 | -0.80008265 | 0.42392356 | 0.85611185 | -6.8341195 | 749 | 492 | 257 | FALSE | FALSE | down |
| ADNI | plasma | A+T+N+ vs A+T-N- | FABP3 | -0.036050468 | 13.157483 | -0.92205969 | 0.3568025 | 0.85611185 | -6.7296836 | 749 | 492 | 257 | FALSE | FALSE | down |
| ADNI | plasma | A+T+N+ vs A+T-N- | FOLR1 | -0.028609182 | 10.313709 | -0.84624615 | 0.39769314 | 0.85611185 | -6.7963311 | 749 | 492 | 257 | FALSE | FALSE | down |
| ADNI | plasma | A+T+N+ vs A+T-N- | GDI1 | -0.053961238 | 8.8631134 | -0.82537949 | 0.40942619 | 0.85611185 | -6.813674 | 749 | 492 | 257 | FALSE | FALSE | down |
| ADNI | plasma | A+T+N+ vs A+T-N- | ICAM1 | 0.026774603 | 12.880154 | 0.86305107 | 0.38839324 | 0.85611185 | -6.7820499 | 749 | 492 | 257 | FALSE | FALSE | up |
| ADNI | plasma | A+T+N+ vs A+T-N- | IL12p70 | -0.056609255 | 13.472706 | -0.78508014 | 0.43266165 | 0.85611185 | -6.8459447 | 749 | 492 | 257 | FALSE | FALSE | down |
| ADNI | plasma | A+T+N+ vs A+T-N- | IL4 | 0.058829982 | 10.925914 | 0.86785501 | 0.38575931 | 0.85611185 | -6.7779159 | 749 | 492 | 257 | FALSE | FALSE | up |
| ADNI | plasma | A+T+N+ vs A+T-N- | IL6 | -0.044401644 | 12.900478 | -0.89620886 | 0.37043708 | 0.85611185 | -6.7530497 | 749 | 492 | 257 | FALSE | FALSE | down |
| ADNI | plasma | A+T+N+ vs A+T-N- | IL9 | 0.076918639 | 10.50465 | 1.0543805 | 0.29205809 | 0.85611185 | -6.5997047 | 749 | 492 | 257 | FALSE | FALSE | up |
| ADNI | plasma | A+T+N+ vs A+T-N- | KDR | 0.11409554 | 12.319192 | 0.80934252 | 0.41858224 | 0.85611185 | -6.8267092 | 749 | 492 | 257 | FALSE | FALSE | up |
| ADNI | plasma | A+T+N+ vs A+T-N- | KLK6 | -0.037980751 | 12.223126 | -1.0573502 | 0.29070206 | 0.85611185 | -6.5965884 | 749 | 492 | 257 | FALSE | FALSE | down |
| ADNI | plasma | A+T+N+ vs A+T-N- | MDH1 | -0.083454277 | 12.398842 | -0.84305235 | 0.39947569 | 0.85611185 | -6.7990136 | 749 | 492 | 257 | FALSE | FALSE | down |
| ADNI | plasma | A+T+N+ vs A+T-N- | MSLN | -0.055301909 | 13.506658 | -0.7813498 | 0.43485047 | 0.85611185 | -6.8488504 | 749 | 492 | 257 | FALSE | FALSE | down |
| ADNI | plasma | A+T+N+ vs A+T-N- | NPY | 0.10367177 | 14.993106 | 0.97615368 | 0.32931213 | 0.85611185 | -6.6786446 | 749 | 492 | 257 | FALSE | FALSE | up |
| ADNI | plasma | A+T+N+ vs A+T-N- | SFRP1 | 0.072557371 | 15.225401 | 0.8680429 | 0.38565652 | 0.85611185 | -6.7777538 | 749 | 492 | 257 | FALSE | FALSE | up |
| ADNI | plasma | A+T+N+ vs A+T-N- | SNCA | -0.072798841 | 13.626088 | -0.78201516 | 0.43445959 | 0.85611185 | -6.8483331 | 749 | 492 | 257 | FALSE | FALSE | down |
| ADNI | plasma | A+T+N+ vs A+T-N- | TEK | -0.027909056 | 12.808911 | -1.0540778 | 0.29219654 | 0.85611185 | -6.6000218 | 749 | 492 | 257 | FALSE | FALSE | down |
| ADNI | plasma | A+T+N+ vs A+T-N- | UBB | 0.12495081 | 12.298305 | 0.97707699 | 0.32885517 | 0.85611185 | -6.6777482 | 749 | 492 | 257 | FALSE | FALSE | up |
| ADNI | plasma | A+T+N+ vs A+T-N- | VEGFD | -0.066586143 | 12.280984 | -0.88360476 | 0.37720092 | 0.85611185 | -6.7642019 | 749 | 492 | 257 | FALSE | FALSE | down |
| ADNI | plasma | A+T+N+ vs A+T-N- | YWHAZ | 0.023867874 | 11.80986 | 0.80434285 | 0.42146122 | 0.85611185 | -6.8307208 | 749 | 492 | 257 | FALSE | FALSE | up |
| ADNI | plasma | A+T+N+ vs A+T-N- | CCL3 | -0.047240389 | 11.788168 | -0.75030098 | 0.45331555 | 0.8614809 | -6.8724988 | 749 | 492 | 257 | FALSE | FALSE | down |
| ADNI | plasma | A+T+N+ vs A+T-N- | FLT1 | 0.023784803 | 13.463576 | 0.70520639 | 0.48090707 | 0.8614809 | -6.9051403 | 749 | 492 | 257 | FALSE | FALSE | up |
| ADNI | plasma | A+T+N+ vs A+T-N- | HTT | -0.095558627 | 13.817664 | -0.70802772 | 0.47915436 | 0.8614809 | -6.9031573 | 749 | 492 | 257 | FALSE | FALSE | down |
| ADNI | plasma | A+T+N+ vs A+T-N- | IL6R | -0.026162478 | 12.076902 | -0.68704194 | 0.4922748 | 0.8614809 | -6.9177179 | 749 | 492 | 257 | FALSE | FALSE | down |
| ADNI | plasma | A+T+N+ vs A+T-N- | PGK1 | -0.12696522 | 10.692758 | -0.72070252 | 0.4713236 | 0.8614809 | -6.8941512 | 749 | 492 | 257 | FALSE | FALSE | down |
| ADNI | plasma | A+T+N+ vs A+T-N- | POSTN | 0.039836441 | 13.457557 | 0.73316244 | 0.46369506 | 0.8614809 | -6.8851424 | 749 | 492 | 257 | FALSE | FALSE | up |
| ADNI | plasma | A+T+N+ vs A+T-N- | SMOC1 | 0.068618077 | 12.769667 | 0.69062769 | 0.49001937 | 0.8614809 | -6.9152609 | 749 | 492 | 257 | FALSE | FALSE | up |
| ADNI | plasma | A+T+N+ vs A+T-N- | TNF | 0.027846593 | 13.360808 | 0.74568216 | 0.45609981 | 0.8614809 | -6.8759349 | 749 | 492 | 257 | FALSE | FALSE | up |
| ADNI | plasma | A+T+N+ vs A+T-N- | PTN | 0.044577673 | 6.8668589 | 0.6707608 | 0.50258538 | 0.86747613 | -6.9287128 | 749 | 492 | 257 | FALSE | FALSE | up |
| ADNI | plasma | A+T+N+ vs A+T-N- | CRH | 0.052520949 | 12.228825 | 0.64266046 | 0.52064652 | 0.87172012 | -6.94707 | 749 | 492 | 257 | FALSE | FALSE | up |
| ADNI | plasma | A+T+N+ vs A+T-N- | GDF15 | 0.11975097 | 12.20702 | 0.62220579 | 0.53400112 | 0.87172012 | -6.9599391 | 749 | 492 | 257 | FALSE | FALSE | up |
| ADNI | plasma | A+T+N+ vs A+T-N- | SOD1 | -0.058349189 | 12.059307 | -0.64395993 | 0.51980397 | 0.87172012 | -6.9462384 | 749 | 492 | 257 | FALSE | FALSE | down |
| ADNI | plasma | A+T+N+ vs A+T-N- | TARDBP | -0.08343966 | 13.339959 | -0.62223699 | 0.53398062 | 0.87172012 | -6.9599197 | 749 | 492 | 257 | FALSE | FALSE | down |
| ADNI | plasma | A+T+N+ vs A+T-N- | VEGFA | -0.018942155 | 13.734833 | -0.61365273 | 0.53963626 | 0.87172012 | -6.9651969 | 749 | 492 | 257 | FALSE | FALSE | down |
| ADNI | plasma | A+T+N+ vs A+T-N- | Abeta38 | 0.037015916 | 9.5661426 | 0.54845701 | 0.58354606 | 0.92941379 | -7.0028857 | 749 | 492 | 257 | FALSE | FALSE | up |
| ADNI | plasma | A+T+N+ vs A+T-N- | IGFBP7 | 0.011184399 | 13.116618 | 0.52981637 | 0.59640057 | 0.92941379 | -7.0128849 | 749 | 492 | 257 | FALSE | FALSE | up |
| ADNI | plasma | A+T+N+ vs A+T-N- | SNCB | -0.01408556 | 10.836914 | -0.52825909 | 0.59748029 | 0.92941379 | -7.0137046 | 749 | 492 | 257 | FALSE | FALSE | down |
| ADNI | plasma | A+T+N+ vs A+T-N- | FGF2 | -0.07430665 | 13.507225 | -0.51076886 | 0.60966756 | 0.93680625 | -7.0227455 | 749 | 492 | 257 | FALSE | FALSE | down |
| ADNI | plasma | A+T+N+ vs A+T-N- | ANXA5 | -0.079596984 | 12.278259 | -0.34833952 | 0.72768579 | 0.96665716 | -7.0921754 | 749 | 492 | 257 | FALSE | FALSE | down |
| ADNI | plasma | A+T+N+ vs A+T-N- | Abeta40 | 0.011016904 | 10.912269 | 0.17681902 | 0.85969966 | 0.96665716 | -7.1369993 | 749 | 492 | 257 | FALSE | FALSE | up |
| ADNI | plasma | A+T+N+ vs A+T-N- | BDNF | -0.042072342 | 13.693862 | -0.3571656 | 0.72107121 | 0.96665716 | -7.089077 | 749 | 492 | 257 | FALSE | FALSE | down |
| ADNI | plasma | A+T+N+ vs A+T-N- | CALB2 | 0.0036084268 | 10.047998 | 0.078219787 | 0.93767469 | 0.96665716 | -7.1495139 | 749 | 492 | 257 | FALSE | FALSE | up |
| ADNI | plasma | A+T+N+ vs A+T-N- | CCL17 | -0.019880179 | 13.542329 | -0.13447159 | 0.89306679 | 0.96665716 | -7.1435596 | 749 | 492 | 257 | FALSE | FALSE | down |
| ADNI | plasma | A+T+N+ vs A+T-N- | CCL22 | -0.017336206 | 12.845235 | -0.38562814 | 0.6998846 | 0.96665716 | -7.0785571 | 749 | 492 | 257 | FALSE | FALSE | down |
| ADNI | plasma | A+T+N+ vs A+T-N- | CCL26 | 0.019898645 | 11.472891 | 0.25679013 | 0.79741324 | 0.96665716 | -7.1197428 | 749 | 492 | 257 | FALSE | FALSE | up |
| ADNI | plasma | A+T+N+ vs A+T-N- | CST3 | -0.0017328004 | 13.314189 | -0.087938478 | 0.92994973 | 0.96665716 | -7.1487102 | 749 | 492 | 257 | FALSE | FALSE | down |
| ADNI | plasma | A+T+N+ vs A+T-N- | CXCL8 | 0.019271507 | 10.264985 | 0.29038125 | 0.77160717 | 0.96665716 | -7.1105964 | 749 | 492 | 257 | FALSE | FALSE | up |
| ADNI | plasma | A+T+N+ vs A+T-N- | DDC | 0.0090012759 | 11.437847 | 0.18078903 | 0.85658342 | 0.96665716 | -7.1362928 | 749 | 492 | 257 | FALSE | FALSE | up |
| ADNI | plasma | A+T+N+ vs A+T-N- | ENO2 | -0.024197692 | 12.815746 | -0.30964455 | 0.75691977 | 0.96665716 | -7.1048447 | 749 | 492 | 257 | FALSE | FALSE | down |
| ADNI | plasma | A+T+N+ vs A+T-N- | FCN2 | -0.005958012 | 12.46047 | -0.11328327 | 0.90983717 | 0.96665716 | -7.1461721 | 749 | 492 | 257 | FALSE | FALSE | down |
| ADNI | plasma | A+T+N+ vs A+T-N- | GDNF | -0.065330469 | 11.259736 | -0.4155863 | 0.67783506 | 0.96665716 | -7.0666141 | 749 | 492 | 257 | FALSE | FALSE | down |
| ADNI | plasma | A+T+N+ vs A+T-N- | HBA1 | 0.063173406 | 6.9511714 | 0.41069157 | 0.68141939 | 0.96665716 | -7.0686264 | 749 | 492 | 257 | FALSE | FALSE | up |
| ADNI | plasma | A+T+N+ vs A+T-N- | IFNG | -0.015513284 | 12.582376 | -0.18302692 | 0.85482779 | 0.96665716 | -7.1358876 | 749 | 492 | 257 | FALSE | FALSE | down |
| ADNI | plasma | A+T+N+ vs A+T-N- | IL13 | -0.018136828 | 13.114115 | -0.16055072 | 0.87249175 | 0.96665716 | -7.1397306 | 749 | 492 | 257 | FALSE | FALSE | down |
| ADNI | plasma | A+T+N+ vs A+T-N- | IL16 | -0.0033982106 | 12.801434 | -0.076373294 | 0.93914307 | 0.96665716 | -7.149656 | 749 | 492 | 257 | FALSE | FALSE | down |
| ADNI | plasma | A+T+N+ vs A+T-N- | IL17A | -0.025271564 | 11.22086 | -0.22301002 | 0.82359018 | 0.96665716 | -7.1278084 | 749 | 492 | 257 | FALSE | FALSE | down |
| ADNI | plasma | A+T+N+ vs A+T-N- | IL18 | -0.015020373 | 13.05222 | -0.32391854 | 0.74609262 | 0.96665716 | -7.1003446 | 749 | 492 | 257 | FALSE | FALSE | down |
| ADNI | plasma | A+T+N+ vs A+T-N- | IL33 | 0.0080192398 | 12.153288 | 0.19635046 | 0.84439059 | 0.96665716 | -7.1333721 | 749 | 492 | 257 | FALSE | FALSE | up |
| ADNI | plasma | A+T+N+ vs A+T-N- | IL5 | -0.02924684 | 13.124978 | -0.32031396 | 0.74882213 | 0.96665716 | -7.1015001 | 749 | 492 | 257 | FALSE | FALSE | down |
| ADNI | plasma | A+T+N+ vs A+T-N- | IL7 | 0.007584285 | 13.074998 | 0.092433706 | 0.92637886 | 0.96665716 | -7.1483067 | 749 | 492 | 257 | FALSE | FALSE | up |
| ADNI | plasma | A+T+N+ vs A+T-N- | MME | -0.014435821 | 9.3394734 | -0.10301729 | 0.91797758 | 0.96665716 | -7.1472772 | 749 | 492 | 257 | FALSE | FALSE | down |
| ADNI | plasma | A+T+N+ vs A+T-N- | NRGN | -0.022475997 | 16.258818 | -0.12311736 | 0.9020481 | 0.96665716 | -7.1450152 | 749 | 492 | 257 | FALSE | FALSE | down |
| ADNI | plasma | A+T+N+ vs A+T-N- | Oligo-SNCA | -0.070591664 | 11.992173 | -0.26579043 | 0.79047576 | 0.96665716 | -7.1174023 | 749 | 492 | 257 | FALSE | FALSE | down |
| ADNI | plasma | A+T+N+ vs A+T-N- | PDLIM5 | 0.056144595 | 10.603971 | 0.33967294 | 0.73420068 | 0.96665716 | -7.0951424 | 749 | 492 | 257 | FALSE | FALSE | up |
| ADNI | plasma | A+T+N+ vs A+T-N- | PRDX6 | -0.048680145 | 12.026644 | -0.42517156 | 0.67083709 | 0.96665716 | -7.0626043 | 749 | 492 | 257 | FALSE | FALSE | down |
| ADNI | plasma | A+T+N+ vs A+T-N- | PSEN1 | -0.004914402 | 12.167326 | -0.070718094 | 0.94364152 | 0.96665716 | -7.1500699 | 749 | 492 | 257 | FALSE | FALSE | down |
| ADNI | plasma | A+T+N+ vs A+T-N- | REST | 0.01637074 | 12.958579 | 0.31457263 | 0.75317617 | 0.96665716 | -7.103314 | 749 | 492 | 257 | FALSE | FALSE | up |
| ADNI | plasma | A+T+N+ vs A+T-N- | RUVBL2 | 0.011334501 | 11.182762 | 0.21127373 | 0.83273273 | 0.96665716 | -7.1303449 | 749 | 492 | 257 | FALSE | FALSE | up |
| ADNI | plasma | A+T+N+ vs A+T-N- | S100B | 0.0049835166 | 11.944583 | 0.088456169 | 0.92953841 | 0.96665716 | -7.1486648 | 749 | 492 | 257 | FALSE | FALSE | up |
| ADNI | plasma | A+T+N+ vs A+T-N- | SAA1 | 0.015367606 | 10.754116 | 0.10594599 | 0.91565435 | 0.96665716 | -7.1469727 | 749 | 492 | 257 | FALSE | FALSE | up |
| ADNI | plasma | A+T+N+ vs A+T-N- | SLIT2 | -0.011285245 | 13.239655 | -0.30735093 | 0.75866406 | 0.96665716 | -7.1055489 | 749 | 492 | 257 | FALSE | FALSE | down |
| ADNI | plasma | A+T+N+ vs A+T-N- | SNAP25 | 0.00085385831 | 12.818574 | 0.10920377 | 0.91307092 | 0.96665716 | -7.1466238 | 749 | 492 | 257 | FALSE | FALSE | up |
| ADNI | plasma | A+T+N+ vs A+T-N- | SQSTM1 | 0.026357794 | 11.622467 | 0.39561886 | 0.69250198 | 0.96665716 | -7.0746735 | 749 | 492 | 257 | FALSE | FALSE | up |
| ADNI | plasma | A+T+N+ vs A+T-N- | TAFA5 | 0.0046901775 | 11.049246 | 0.082907246 | 0.93394805 | 0.96665716 | -7.149138 | 749 | 492 | 257 | FALSE | FALSE | up |
| ADNI | plasma | A+T+N+ vs A+T-N- | TIMP3 | 0.047830476 | 14.727755 | 0.38294679 | 0.70187086 | 0.96665716 | -7.0795826 | 749 | 492 | 257 | FALSE | FALSE | up |
| ADNI | plasma | A+T+N+ vs A+T-N- | TREM2 | -0.018514849 | 12.866937 | -0.37535694 | 0.70750422 | 0.96665716 | -7.0824463 | 749 | 492 | 257 | FALSE | FALSE | down |
| ADNI | plasma | A+T+N+ vs A+T-N- | UCHL1 | 0.0015515636 | 11.587707 | 0.15022791 | 0.88062636 | 0.96665716 | -7.1413272 | 749 | 492 | 257 | FALSE | FALSE | up |
| ADNI | plasma | A+T+N+ vs A+T-N- | pSNCA-129 | -0.057082851 | 13.735065 | -0.45370593 | 0.65017553 | 0.96665716 | -7.0501268 | 749 | 492 | 257 | FALSE | FALSE | down |
| ADNI | plasma | A+T+N+ vs A+T-N- | pTDP43-409 | 0.0069980154 | 10.729889 | 0.15047677 | 0.88043011 | 0.96665716 | -7.14129 | 749 | 492 | 257 | FALSE | FALSE | up |
| ADNI | plasma | A+T+N+ vs A+T-N- | NPTX1 | -0.0011772516 | 11.559819 | -0.033681478 | 0.97314037 | 0.98883618 | -7.1519943 | 749 | 492 | 257 | FALSE | FALSE | down |
| ADNI | plasma | A+T+N+ vs A+T-N- | CXCL1 | -0.00075981605 | 11.986626 | -0.0076368388 | 0.99390883 | 0.99891177 | -7.1525298 | 749 | 492 | 257 | FALSE | FALSE | down |
| ADNI | plasma | A+T+N+ vs A+T-N- | NEFH | -0.00024390738 | 18.51471 | -0.0013643666 | 0.99891177 | 0.99891177 | -7.1525579 | 749 | 492 | 257 | FALSE | FALSE | down |
