# Supplementary material for: Cross‐cohort NULISAseq identifies tau‐anchored CSF and plasma‐specific signatures across the amyloid‐positive Alzheimer's disease
Source: Alzheimers Dement. 2026 Oct 12;22(10):e71915. doi: 10.1002/alz.71915 (PMC13656197; doi:10.1002/alz.71915)
Supplement: Supplementary file 4 [file ALZ-22-e71915-s004.docx]

**Supplementary Material**

*Cross-cohort NULISAseq identifies tau-anchored CSF and plasma-specific signatures across the amyloid-positive Alzheimer’s disease*

Manuscript ID: ADJ-D-26-01660

**Contents**

Supplementary Table S1. ADNI A^+^ AD-only sensitivity analysis

Supplementary Table S2. Tau-adjustment sensitivity analyses, diagnosis-conditioned and diagnosis-omitted model performance, diagnosis-stratified protein–cognition correlations and rank-covariance decomposition, and CSF–plasma sampling intervals (provided as separate editable Word file)

Supplementary Table S3. Descriptive distributions across ATN groups

Supplementary Table S4. Exploratory longitudinal cognitive-slope associations

Supplementary Table S5. Protein-cognition correlation analyses (CSF and plasma)

Supplementary Table S6. CSF versus plasma pTau-217 Steiger tests

Supplementary Table S7. Full limma differential-expression results for the prespecified ATN contrasts in Beijing CSF, ADNI CSF, and ADNI plasma (provided as separate editable Word file)

Supplementary Figure S1. NULISA platform quality control

Supplementary Figure S2. Beijing NULISA-INNOTEST CSF tau concordance

Supplementary Figure S3. Non-tau proteins with cross-cohort FDR support but subthreshold Beijing effect sizes

*Note: Supplementary Tables S2 and S7 are provided as separate freestanding editable Word files, each with its own caption and legend.*

Supplementary Table S1. ADNI A+ AD-only sensitivity analysis for the eight proteins in the primary cross-cohort set. Diagnosis was anchored to the NULISA CSF index by exact VISCODE2 match when available and otherwise by nearest date. The QC-passed AD-only sample comprised n=221 for the T and N contrasts and n=210 for the concordant-extreme contrast. All displayed effects were directionally concordant with Beijing. Two-sided moderated-t 95% confidence intervals from limma are reported for every AD-only estimate and are not adjusted for multiplicity or feature selection. For MDH1, SMOC1, FABP3, and VSNL1 in their Beijing-nominated contrasts, every interval included 0.5; this subset therefore did not resolve whether the underlying effects exceeded the prespecified point-estimate magnitude threshold.

| **Contrast** | **Protein** | **Class** | **Beijing log₂FC** | **Beijing FDR** | **ADNI primary log₂FC** | **ADNI primary FDR** | **ADNI AD-only log₂FC** | **ADNI AD-only FDR** | **ADNI AD-only 95% CI lower** | **ADNI AD-only 95% CI upper** |
| --- | --- | --- | --- | --- | --- | --- | --- | --- | --- | --- |
| T+ vs T- | pTau-181 | tau-family | 0.942 | 0.000646 | 1.275 | 1.14e-56 | 0.594 | 0.025 | 0.197 | 0.992 |
| T+ vs T- | pTau-217 | tau-family | 1.090 | 0.000646 | 1.394 | 2.57e-52 | 0.530 | 0.0805 | 0.075 | 0.985 |
| T+ vs T- | pTau-231 | tau-family | 0.976 | 0.00128 | 1.294 | 1.87e-53 | 0.558 | 0.044 | 0.139 | 0.977 |
| T+ vs T- | MAPT | tau-family | 0.478 | 0.0804 | 1.079 | 8.58e-61 | 0.590 | 0.0184 | 0.251 | 0.929 |
| T+ vs T- | SMOC1 | non-tau | 0.533 | 0.027 | 0.827 | 5.62e-36 | 0.476 | 0.044 | 0.111 | 0.840 |
| T+ vs T- | MDH1 | non-tau | 0.504 | 0.027 | 0.739 | 5.01e-29 | 0.672 | 0.0184 | 0.329 | 1.016 |
| T+ vs T- | FABP3 | non-tau | 0.340 | 0.103 | 0.583 | 3.99e-35 | 0.441 | 0.0217 | 0.164 | 0.718 |
| T+ vs T- | VSNL1 | non-tau | 0.338 | 0.0844 | 0.601 | 2.34e-36 | 0.448 | 0.0184 | 0.179 | 0.717 |
| N+ vs N- | pTau-181 | tau-family | 0.764 | 0.00478 | 1.196 | 1.2e-51 | 0.597 | 0.00343 | 0.258 | 0.936 |
| N+ vs N- | pTau-217 | tau-family | 0.940 | 0.00357 | 1.291 | 2.3e-46 | 0.555 | 0.0167 | 0.166 | 0.944 |
| N+ vs N- | pTau-231 | tau-family | 0.854 | 0.00478 | 1.209 | 2.83e-48 | 0.584 | 0.006 | 0.227 | 0.941 |
| N+ vs N- | MAPT | tau-family | 0.631 | 0.00357 | 1.039 | 8.88e-59 | 0.645 | 0.000357 | 0.359 | 0.931 |
| N+ vs N- | SMOC1 | non-tau | 0.237 | 0.528 | 0.802 | 1.43e-35 | 0.544 | 0.00343 | 0.235 | 0.854 |
| N+ vs N- | MDH1 | non-tau | 0.267 | 0.435 | 0.753 | 7.91e-32 | 0.739 | 7.7e-05 | 0.450 | 1.027 |
| N+ vs N- | FABP3 | non-tau | 0.400 | 0.02 | 0.573 | 9.79e-36 | 0.469 | 0.00102 | 0.234 | 0.703 |
| N+ vs N- | VSNL1 | non-tau | 0.372 | 0.0207 | 0.591 | 5.63e-37 | 0.499 | 0.00047 | 0.272 | 0.726 |
| A+T+N+ vs A+T-N- | pTau-181 | tau-family | 1.208 | 0.000187 | 1.315 | 1.32e-55 | 0.621 | 0.0199 | 0.215 | 1.027 |
| A+T+N+ vs A+T-N- | pTau-217 | tau-family | 1.460 | 0.000187 | 1.431 | 9.54e-51 | 0.555 | 0.0694 | 0.090 | 1.020 |
| A+T+N+ vs A+T-N- | pTau-231 | tau-family | 1.298 | 0.000329 | 1.333 | 3.54e-52 | 0.585 | 0.0313 | 0.159 | 1.011 |
| A+T+N+ vs A+T-N- | MAPT | tau-family | 0.773 | 0.00374 | 1.126 | 2.73e-61 | 0.626 | 0.00911 | 0.286 | 0.967 |
| A+T+N+ vs A+T-N- | SMOC1 | non-tau | 0.538 | 0.0905 | 0.873 | 2.44e-37 | 0.500 | 0.0315 | 0.133 | 0.867 |
| A+T+N+ vs A+T-N- | MDH1 | non-tau | 0.549 | 0.0725 | 0.791 | 7.32e-31 | 0.712 | 0.00889 | 0.366 | 1.057 |
| A+T+N+ vs A+T-N- | FABP3 | non-tau | 0.568 | 0.00294 | 0.616 | 1.54e-36 | 0.465 | 0.0118 | 0.187 | 0.742 |
| A+T+N+ vs A+T-N- | VSNL1 | non-tau | 0.512 | 0.0089 | 0.636 | 4.82e-38 | 0.475 | 0.0103 | 0.205 | 0.745 |

**Supplementary Table S3.** Descriptive distributions across ATN groups for the eight strict cross-cohort consistent proteins within the ADNI A^+^ baseline cohort (n=865). Mean NULISA NPQ is shown across four ATN strata (A^+^T^-^N^-^ n=275, A^+^T^+^N^-^ n=42, A^+^T^-^N^+^ n=5, A^+^T^+^N^+^ n=543). The rightmost column indicates whether each protein showed a monotonic increase in group means across the ordered A^+^T^-^N^-^ → A^+^T^+^N^-^ → A^+^T^+^N^+^ sequence; the sparse A^+^T^-^N^+^ group is shown descriptively. These cross-sectional group distributions are descriptive and should not be interpreted as a temporal or disease-progression trajectory.

| **Protein** | **A+T−N− mean** | **A+T−N− n** | **A+T+N− mean** | **A+T+N− n** | **A+T−N+ mean** | **A+T−N+ n** | **A+T+N+ mean** | **A+T+N+ n** | **Δ: A+T+N+ − A+T−N−** | **Ordered increase** |
| --- | --- | --- | --- | --- | --- | --- | --- | --- | --- | --- |
| pTau-181 | 16.572 | 275 | 17.490 | 42 | 17.043 | 5 | 17.916 | 543 | 1.344 | Yes |
| pTau-217 | 15.006 | 275 | 16.160 | 42 | 15.534 | 5 | 16.495 | 543 | 1.489 | Yes |
| pTau-231 | 17.025 | 275 | 17.990 | 42 | 17.538 | 5 | 18.389 | 543 | 1.364 | Yes |
| MAPT | 14.658 | 275 | 15.360 | 42 | 15.039 | 5 | 15.817 | 543 | 1.159 | Yes |
| FABP3 | 11.469 | 275 | 11.754 | 42 | 11.610 | 5 | 12.085 | 543 | 0.616 | Yes |
| SMOC1 | 11.347 | 275 | 11.954 | 42 | 11.914 | 5 | 12.250 | 543 | 0.903 | Yes |
| MDH1 | 10.294 | 275 | 10.515 | 42 | 10.631 | 5 | 11.093 | 543 | 0.800 | Yes |
| VSNL1 | 12.551 | 275 | 12.797 | 42 | 13.034 | 5 | 13.177 | 543 | 0.626 | Yes |

Supplementary Table S4. Exploratory longitudinal cognitive-slope associations for the eight proteins in the primary cross-cohort set. Slopes used assessments from 0.25 years before the CSF draw onward, required at least three assessments and an observation span of at least one year, and were estimable for n=737 (CDR-SB) and n=724 (MMSE). Spearman correlations with baseline NPQ were unadjusted, two-sided, and not corrected for multiple comparisons.

| **Protein** | **CDR-SB n** | **Mean CDR-SB slope/y** | **ρ: NPQ vs CDR-SB slope** | **p** | **MMSE n** | **Mean MMSE slope/y** | **ρ: NPQ vs MMSE slope** | **p** |
| --- | --- | --- | --- | --- | --- | --- | --- | --- |
| pTau-181 | 737 | 0.863 | 0.295 | 2.67e-16 | 724 | -1.186 | -0.310 | 1.52e-17 |
| pTau-217 | 737 | 0.863 | 0.269 | 1.02e-13 | 724 | -1.186 | -0.284 | 6.67e-15 |
| pTau-231 | 737 | 0.863 | 0.258 | 1.11e-12 | 724 | -1.186 | -0.274 | 5.83e-14 |
| MAPT | 737 | 0.863 | 0.255 | 2.32e-12 | 724 | -1.186 | -0.261 | 1.07e-12 |
| FABP3 | 737 | 0.863 | 0.184 | 4.91e-07 | 724 | -1.186 | -0.162 | 1.24e-05 |
| SMOC1 | 737 | 0.863 | 0.108 | 0.00339 | 724 | -1.186 | -0.126 | 0.000689 |
| MDH1 | 737 | 0.863 | 0.134 | 0.000257 | 724 | -1.186 | -0.137 | 0.000217 |
| VSNL1 | 737 | 0.863 | 0.122 | 0.000863 | 724 | -1.186 | -0.127 | 0.000633 |

Supplementary Table S5. Protein–cognition correlations in ADNI A+ participants. Cognitive assessments were anchored to the NULISA CSF index by exact VISCODE2 match when available and otherwise by nearest date; under both routes the assessment was required to fall within ±90 days of the CSF index. Outcome-specific CSF n values were 781, 839, and 778 for MMSE, ADAS-Cog13, and CDR-SB; plasma n values were 712, 759, and 710. Spearman correlations are two-sided and descriptive.

| **Fluid** | **Protein** | **Cognitive measure** | **n** | **Spearman ρ** | **p** |
| --- | --- | --- | --- | --- | --- |
| CSF | Elecsys p-tau181 | MMSE | 781 | -0.320 | 5.09e-20 |
| CSF | Elecsys p-tau181 | ADAS_Cog13 | 839 | 0.394 | 1.3e-32 |
| CSF | Elecsys p-tau181 | CDR_SB | 778 | 0.344 | 5.51e-23 |
| CSF | pTau-181 | MMSE | 781 | -0.314 | 2.43e-19 |
| CSF | pTau-181 | ADAS_Cog13 | 839 | 0.352 | 8.01e-26 |
| CSF | pTau-181 | CDR_SB | 778 | 0.342 | 8.81e-23 |
| CSF | pTau-217 | MMSE | 781 | -0.295 | 4e-17 |
| CSF | pTau-217 | ADAS_Cog13 | 839 | 0.321 | 1.35e-21 |
| CSF | pTau-217 | CDR_SB | 778 | 0.316 | 1.51e-19 |
| CSF | pTau-231 | MMSE | 781 | -0.290 | 1.3e-16 |
| CSF | pTau-231 | ADAS_Cog13 | 839 | 0.319 | 2.75e-21 |
| CSF | pTau-231 | CDR_SB | 778 | 0.312 | 5.64e-19 |
| CSF | MAPT | MMSE | 781 | -0.277 | 3e-15 |
| CSF | MAPT | ADAS_Cog13 | 839 | 0.300 | 6.99e-19 |
| CSF | MAPT | CDR_SB | 778 | 0.304 | 4.01e-18 |
| CSF | FABP3 | MMSE | 781 | -0.215 | 1.22e-09 |
| CSF | FABP3 | ADAS_Cog13 | 839 | 0.254 | 7.62e-14 |
| CSF | FABP3 | CDR_SB | 778 | 0.241 | 9.39e-12 |
| CSF | SMOC1 | MMSE | 781 | -0.153 | 1.7e-05 |
| CSF | SMOC1 | ADAS_Cog13 | 839 | 0.159 | 3.48e-06 |
| CSF | SMOC1 | CDR_SB | 778 | 0.176 | 7.77e-07 |
| CSF | MDH1 | MMSE | 781 | -0.142 | 6.8e-05 |
| CSF | MDH1 | ADAS_Cog13 | 839 | 0.171 | 6.7e-07 |
| CSF | MDH1 | CDR_SB | 778 | 0.161 | 6.76e-06 |
| CSF | NRGN | MMSE | 781 | -0.122 | 0.000632 |
| CSF | NRGN | ADAS_Cog13 | 839 | 0.169 | 9.17e-07 |
| CSF | NRGN | CDR_SB | 778 | 0.173 | 1.28e-06 |
| CSF | UCHL1 | MMSE | 781 | -0.144 | 5.33e-05 |
| CSF | UCHL1 | ADAS_Cog13 | 839 | 0.168 | 9.72e-07 |
| CSF | UCHL1 | CDR_SB | 778 | 0.188 | 1.31e-07 |
| CSF | VSNL1 | MMSE | 781 | -0.128 | 0.000329 |
| CSF | VSNL1 | ADAS_Cog13 | 839 | 0.143 | 3.23e-05 |
| CSF | VSNL1 | CDR_SB | 778 | 0.149 | 3.09e-05 |
| CSF | SNCB | MMSE | 781 | -0.133 | 0.000197 |
| CSF | SNCB | ADAS_Cog13 | 839 | 0.139 | 5.2e-05 |
| CSF | SNCB | CDR_SB | 778 | 0.170 | 1.77e-06 |
| CSF | SNCA | MMSE | 781 | -0.123 | 0.000555 |
| CSF | SNCA | ADAS_Cog13 | 839 | 0.140 | 4.55e-05 |
| CSF | SNCA | CDR_SB | 778 | 0.132 | 0.000217 |
| CSF | CHIT1 | MMSE | 781 | -0.156 | 1.19e-05 |
| CSF | CHIT1 | ADAS_Cog13 | 839 | 0.143 | 3.12e-05 |
| CSF | CHIT1 | CDR_SB | 778 | 0.104 | 0.0036 |
| CSF | SOD1 | MMSE | 781 | -0.090 | 0.0122 |
| CSF | SOD1 | ADAS_Cog13 | 839 | 0.082 | 0.0176 |
| CSF | SOD1 | CDR_SB | 778 | 0.081 | 0.0233 |
| CSF | NPTXR | MMSE | 781 | 0.071 | 0.0462 |
| CSF | NPTXR | ADAS_Cog13 | 839 | -0.087 | 0.0118 |
| CSF | NPTXR | CDR_SB | 778 | -0.080 | 0.0259 |
| CSF | TAFA5 | MMSE | 781 | -0.049 | 0.173 |
| CSF | TAFA5 | ADAS_Cog13 | 839 | 0.067 | 0.0523 |
| CSF | TAFA5 | CDR_SB | 778 | 0.053 | 0.142 |
| CSF | BACE1 | MMSE | 781 | -0.031 | 0.385 |
| CSF | BACE1 | ADAS_Cog13 | 839 | 0.051 | 0.136 |
| CSF | BACE1 | CDR_SB | 778 | 0.049 | 0.171 |
| Plasma | pTau-217 | MMSE | 712 | -0.309 | 2.96e-17 |
| Plasma | pTau-217 | ADAS_Cog13 | 759 | 0.415 | 6.24e-33 |
| Plasma | pTau-217 | CDR_SB | 710 | 0.370 | 1.93e-24 |
| Plasma | pTau-181 | MMSE | 712 | -0.282 | 1.59e-14 |
| Plasma | pTau-181 | ADAS_Cog13 | 759 | 0.385 | 3.31e-28 |
| Plasma | pTau-181 | CDR_SB | 710 | 0.327 | 3.67e-19 |
| Plasma | pTau-231 | MMSE | 712 | -0.312 | 1.43e-17 |
| Plasma | pTau-231 | ADAS_Cog13 | 759 | 0.414 | 8.32e-33 |
| Plasma | pTau-231 | CDR_SB | 710 | 0.358 | 7.78e-23 |
| Plasma | MAPT | MMSE | 712 | -0.208 | 2.21e-08 |
| Plasma | MAPT | ADAS_Cog13 | 759 | 0.262 | 2.39e-13 |
| Plasma | MAPT | CDR_SB | 710 | 0.211 | 1.27e-08 |
| Plasma | NRGN | MMSE | 712 | -0.030 | 0.431 |
| Plasma | NRGN | ADAS_Cog13 | 759 | 0.020 | 0.576 |
| Plasma | NRGN | CDR_SB | 710 | -0.002 | 0.967 |
| Plasma | FABP3 | MMSE | 712 | -0.022 | 0.554 |
| Plasma | FABP3 | ADAS_Cog13 | 759 | 0.056 | 0.122 |
| Plasma | FABP3 | CDR_SB | 710 | 0.001 | 0.971 |
| Plasma | SMOC1 | MMSE | 712 | -0.077 | 0.0405 |
| Plasma | SMOC1 | ADAS_Cog13 | 759 | 0.108 | 0.00287 |
| Plasma | SMOC1 | CDR_SB | 710 | 0.090 | 0.0164 |
| Plasma | MDH1 | MMSE | 712 | -0.055 | 0.143 |
| Plasma | MDH1 | ADAS_Cog13 | 759 | 0.059 | 0.106 |
| Plasma | MDH1 | CDR_SB | 710 | 0.043 | 0.254 |
| Plasma | VSNL1 | MMSE | 712 | -0.097 | 0.00948 |
| Plasma | VSNL1 | ADAS_Cog13 | 759 | 0.107 | 0.00311 |
| Plasma | VSNL1 | CDR_SB | 710 | 0.163 | 1.33e-05 |
| Plasma | SOD1 | MMSE | 712 | -0.034 | 0.367 |
| Plasma | SOD1 | ADAS_Cog13 | 759 | 0.029 | 0.432 |
| Plasma | SOD1 | CDR_SB | 710 | 0.042 | 0.266 |
| Plasma | SNCB | MMSE | 712 | -0.031 | 0.404 |
| Plasma | SNCB | ADAS_Cog13 | 759 | 0.027 | 0.461 |
| Plasma | SNCB | CDR_SB | 710 | -0.026 | 0.492 |
| Plasma | UCHL1 | MMSE | 712 | 0.052 | 0.166 |
| Plasma | UCHL1 | ADAS_Cog13 | 759 | -0.031 | 0.401 |
| Plasma | UCHL1 | CDR_SB | 710 | -0.049 | 0.196 |
| Plasma | GFAP | MMSE | 712 | -0.218 | 4.11e-09 |
| Plasma | GFAP | ADAS_Cog13 | 759 | 0.293 | 1.52e-16 |
| Plasma | GFAP | CDR_SB | 710 | 0.243 | 5.66e-11 |
| Plasma | NEFL | MMSE | 712 | -0.269 | 2.92e-13 |
| Plasma | NEFL | ADAS_Cog13 | 759 | 0.287 | 7.24e-16 |
| Plasma | NEFL | CDR_SB | 710 | 0.229 | 6.47e-10 |
| Plasma | ACHE | MMSE | 712 | -0.315 | 6.46e-18 |
| Plasma | ACHE | ADAS_Cog13 | 759 | 0.367 | 1.37e-25 |
| Plasma | ACHE | CDR_SB | 710 | 0.394 | 7.83e-28 |
| Plasma | NGF | MMSE | 712 | -0.050 | 0.181 |
| Plasma | NGF | ADAS_Cog13 | 759 | 0.085 | 0.0186 |
| Plasma | NGF | CDR_SB | 710 | 0.074 | 0.0493 |

Supplementary Table S6. Steiger (1980) tests comparing dependent CSF and plasma pTau-217 correlations with cognition. Cognitive assessments were within ±90 days of the CSF draw. The test was applied to midranks and used the observed CSF–plasma pTau-217 correlation. Sample sizes are outcome-specific. Holm p values control all 15 outcome-by-window comparisons reported in Supplementary Table S2, Panel F. This table reports the all-pairs comparisons; same-day and interval-restricted sensitivity analyses are reported in Supplementary Table S2, Panel F.

| **Cognitive measure** | **n** | **CSF ρ** | **Plasma ρ** | **CSF–plasma ρ** | **Steiger z** | **p** | **Holm p** |
| --- | --- | --- | --- | --- | --- | --- | --- |
| MMSE | 712 | -0.262 | -0.309 | 0.373 | 1.192 | 0.233 | 0.878 |
| ADAS-Cog13 | 759 | 0.300 | 0.415 | 0.362 | -3.080 | 0.00207 | 0.0269 |
| CDR-SB | 710 | 0.299 | 0.370 | 0.372 | -1.820 | 0.0687 | 0.622 |

Supplementary Figure S1. NULISA platform quality control for the CNS Disease Panel in 60 Beijing A+ AD CSF samples. (A) Per-target detectability across 127 targets; 92/127 (72.4%) had NPQ above LoD in at least 90% of samples. (B) Intra-plate CV distribution from AQSC replicate controls after transforming NPQ to the linear 2ᴺᴾQ scale; median CV was 5.50%. (C) Principal component analysis of the NPQ matrix; PC1 and PC2 explained 31.0% and 9.8% of variance. (D) Per-sample Alamar QC metrics; 59/60 samples passed all criteria, and one sample had a marginal internal-control warning (−41.5%) but was retained.

**Supplementary Figure S2.** Beijing NULISA-INNOTEST CSF tau concordance (n=60 A^+^ AD). Spearman correlations between NULISAseq NPQ values and matched Fujirebio INNOTEST CSF ELISA measurements for the four NULISA tau-family analytes. (A) NULISA pTau-181 vs INNOTEST pTau-181 (same epitope, Thr181 phosphorylation; ρ=0.79, p=6.2×10^-14^). (B) NULISA pTau-217 vs INNOTEST pTau-181 (cross-epitope, Thr217 vs Thr181; ρ=0.79, p=1.0×10^-13^). (C) NULISA pTau-231 vs INNOTEST pTau-181 (cross-epitope, Thr231 vs Thr181; ρ=0.75, p=8.5×10^-12^). (D) NULISA MAPT vs INNOTEST total tau (same target; ρ=0.85, p=2.0×10^-17^). Red regression lines are shown with shaded 95% confidence bands.

**Supplementary Figure S3. Non-tau proteins with FDR support in both cohorts but effect sizes below the prespecified |log₂FC| ≥ 0.5 threshold in Beijing. These exploratory, lower-amplitude signals were not included in the cross-cohort consistent set displayed in Figure 3. Four non-tau proteins are shown at their strongest available contrast: NRGN and SNCB in A+T+N+ vs A+T-N-, PSEN1 in T+ vs T-, and UCHL1 in N+ vs N-. Beijing (blue) and ADNI (orange) effect sizes are shown side by side with 95% confidence intervals; the dotted vertical line marks the |log₂FC| = 0.5 threshold and the dashed vertical line marks zero. Contrast-specific group sizes were 36 versus 12 in Beijing and 543 versus 275 in ADNI for the concordant-extreme NRGN and SNCB estimates; 44 versus 16 and 585 versus 280 for the T-defined PSEN1 estimate; and 40 versus 20 and 548 versus 317 for the N-defined UCHL1 estimate. SNCB, PSEN1, and UCHL1 did not reach |log₂FC| ≥ 0.5 in either cohort. NRGN met both thresholds in ADNI (log₂FC 0.596–0.634 across the three contrasts) but not the Beijing effect-size threshold, and is shown here as a reference analyte.**
